# Accessing Self-Illuminated, Luminescent Lanthanide Probes by Enzymatic Radiophosphorylation

**DOI:** 10.1021/acs.inorgchem.5c04556

**Published:** 2025-12-12

**Authors:** Georgia G. Sands, Yichong Lao, M. Andrey Joaqui-Joaqui, Xuhui Huang, Eszter Boros

**Affiliations:** Department of Chemistry, 5228 University of Wisconsin-Madison, Madison, Wisconsin 53706, United States

## Abstract

Here, we describe the design and synthesis of Tb^3+^ and Eu^3+^ complexes appended to a kinase-substrate peptide, enabling the incorporation of ^32^P, a potent Cerenkov emitter, by enzymatic phosphorylation to form a metallacyclized peptide structure with a dual turn-on effect. The construct was optimized to accommodate one inner-sphere donor, identifying 8-coordinate, tricazamacrocycles as ideal to produce a selective turn-on response by displacement of an inner-sphere water molecule by phosphate. The optimization of the peptide sequence allowed for the maximization of PKCα kinase-induced luminescence enhancement. The resulting peptide gave a selective turn-on response of 15% upon displacement of inner-sphere waters. Sequence elongation or rigidification results in disruption of the O-coordination of phosphoserine, as evidenced by NMR spectroscopy and supported by Molecular dynamics (MD) simulations. The enzymatic incorporation of ^32^P to the lead peptide–chelate structure was successfully demonstrated with a 95% radiochemical yield and radiochemical purity. Subsequent optical imaging experiments demonstrate the highest probe sensitivity reported to date for a lanthanide probe employing conventional, optical imaging tools, with 0.2 nmol Tb^3+^ complex producing an optical signal above the limit of detection in the presence of 10 μCi ^32^P. This work validates enzymatic radiophosphorylation as a suitable strategy to synthesize self-illuminated lanthanide complexes.

## Graphical abstract

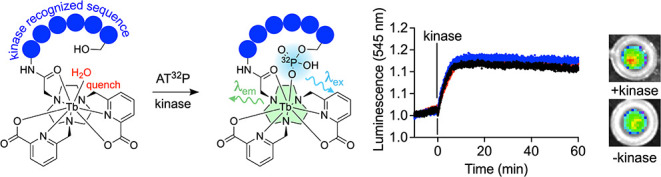

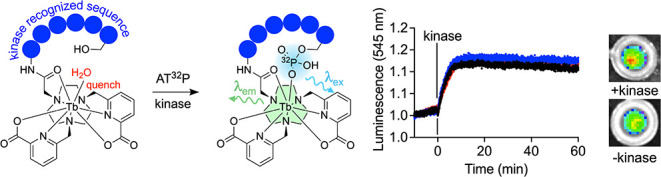

## Introduction

Discrete, lanthanide coordination complexes are an attractive compound class for sensing and imaging applications due to their water solubility, narrow emission profile, enhanced photostability, and long luminescence lifetimes.^1–6^ These optical properties arise from the de-excitation of excited 4f states, which are Laporte forbidden.^7^ Therefore, their sensitization is generally achieved indirectly by the excitation of a small organic chromophore with an energetically matched *S*_1_ and *T*_1_ state, which populates lanthanide excited states by energy transfer. This Dexter process is strongly distance-dependent, necessitating the incorporation of the sensitizer in the first coordination sphere of the lanthanide (Figure 1).^7–10^

**Figure 1:**
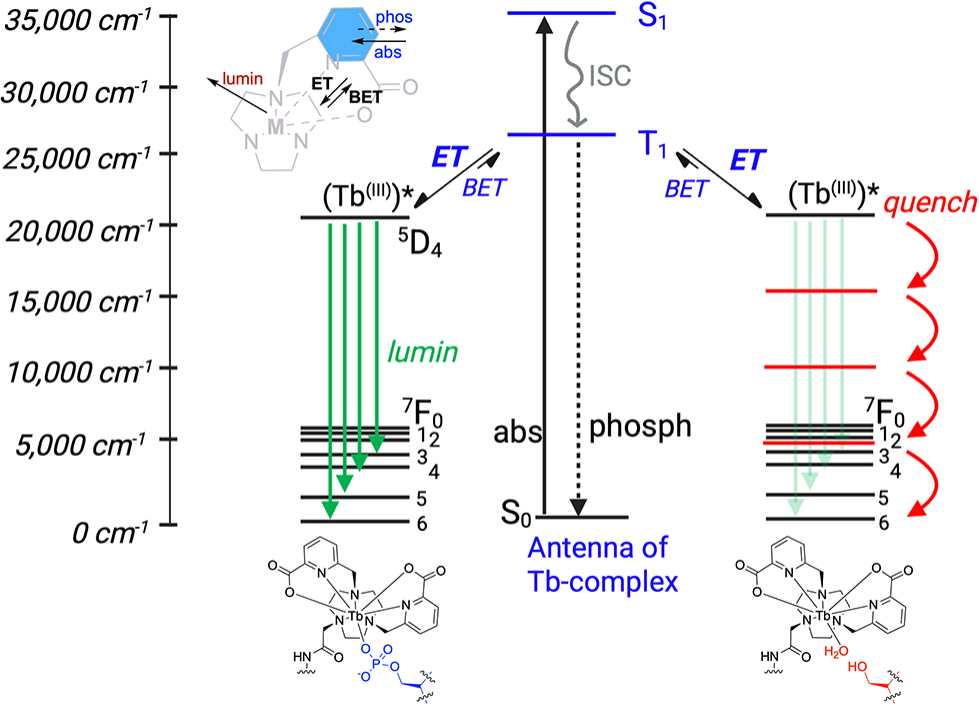
Jablonski diagram of Tb^3+^ luminescence with phosphorylated serine or water coordinated.

The optimal excitation wavelength for lanthanide complexes that have high quantum yields (Tb^3+^, Eu^3+^) generally falls into the UV region of the spectrum,^2,7,11–13^ which is not compatible with single photon excitation and conventional optical imaging of higher, nontransparent organisms.^14^ Additionally, lanthanide luminescence can be achieved through two-photon excitation^12^ or up-conversion systems;^15^ however, custom laser setups and elaborate complex structure that are not in vivo inert are required, respectively, limiting their application.

To overcome these barriers, our group, as well as Cao and co-workers^16^ and the Wang^17^ group, has demonstrated that Cerenkov luminescence, produced by charged particle emitting radioisotopes, can serve as an in situ excitation source.^18,19^ Cerenkov Radiation (CR) is produced when charged particles move through a dielectric medium faster than the speed of light.^20^ In our previous work, we demonstrated, that radioisotopes ^68^Ga (*t*_1/2_ = 68 min, β_avg_ = 0.89 MeV, 89% β^+^) and ^18^F (*t*_1/2_ = 109 min, β_avg_ = 0.25 MeV, 97% β^+^) can readily excite lanthanide complexes in situ through intermolecular excitation mechanisms, termed Cerenkov Radiation Energy Transfer (CRET).^21^ Cerenkov excitation allows for imaging with a standard small animal imager as opposed to techniques that exploit the long luminescence lifetimes of lanthanide metals, which require expensive, time-resolved equipment. While it is feasible to sensitize lanthanide particles with X-ray scintillation methods,^22^ the greater localized photon emission and lower radioactive dose represent a significant advantage for the use of Cerenkov radiation as an excitation source. While these provide the means to detect as little as 5 nmol lanthanide complex in solution, strategies that retain the Cerenkov emitter in immediate vicinity to the lanthanide by a covalent linkage can provide improved efficiency of CRET. Previous work by our group has investigated intermolecular CRET where a ^68^Ga-labeled prostate-specific membrane antigen (PSMA) targeted probe was coinjected with a ^nat^Eu PSMA-targeted complex.^21^ While CRET-mediated Eu^3+^ luminescence was achieved in vivo, both probes were designed to bind the same target tissue, making their accumulation competitive, limiting optical emission. By incorporating a radioisotope within the lanthanide complex, competitive binding can be circumvented.

To this end, two intramolecular, or “self-illuminated”, systems have been explored to date: one consists of the Cerenkov emitting ^89^Zr isotope complexed by a desferrioxamine linked to a luminescent Tb^3+^ complex.^19^ The second approach involves radio-iodination of a Tb^3+^ complex linked to a tyrosine using electrophilic aromatic substitution with ^124^I.^23^ Both approaches presented significant drawbacks, including poor synthetic accessibility for the heterobimetallic construct as well as radiochemical yield and solubility challenges for the radio-iodinated conjugate (Figure S1). These have prevented the implementation, evaluation, and advancement of intramolecular CRET probes to date.

An optimal, intramolecular CRET system must fulfill several requirements: (1) the construct should be synthetically readily accessible and the synthesis should be modular to allow incorporation of different linker structures and targeting vectors; (2) the lanthanide chelate must be inert and have high quantum yield; and (3) the radioactive isotope must be an efficient Cerenkov emitter, with radiochemical labeling proceeding with high yields to negate cumbersome purification.

Herein, we explore an approach that produces an intramolecular CRET system without significant change in water solubility and good synthetic accessibility, employing enzymatic phosphorylation with [γ-^32^P]-ATP. Inspired by previous work on kinase-sensing lanthanide probes, we identify a chelate–peptide combination that exhibits selective turn-on following the enzymatic phosphorylation of a serine. The corresponding radiophosphorylated peptide Tb^3+^ complex was probed using optical imaging experiments for the efficiency of self-illumination in vitro.

## Results and Discussion

### Molecular Design Considerations

In our previous work, we successfully explored high-energy positron emitters such as ^68^Ga, ^18^F, and ^89^Zr as CRET sources for lanthanide excitation. However, beta minus emitters can also readily produce Cerenkov radiation, and because of the lack of the antimatter annihilation event, they persist and produce more photons.^24^ Among these, ^32^P (*t*_1/2_ = 14.3 day, β_avg_ = 0.70 MeV, 100% β^–^) is particularly well-suited to generate high intensity Cerenkov radiance, making it an excellent candidate for CRET.^20^

The ^32^P isotope, generally in the form of AT^32^P, is extensively used for biochemical applications, such as the elucidation of metabolic pathways and quantification of protein kinase activity by selective phosphorylation of serine or tyrosine residues in peptides and proteins. As such, radiochemical labeling strategies are well-established and can be readily implemented upon recognition of a peptide substrate by the kinase.

In addition to the relative ease of incorporating the CR source, this approach also offers the exploration of a kinase-responsive lanthanide complex. Several groups have successfully pursued lanthanide-based phosphate and kinase sensors.^25,26^ Pazos and co-workers’ design encompassed a turn-off probe where phosphorylated serine bound to the metal center displaced the sensitizing antenna (Figure 2A).^27^ The Butler group synthesized a europium-based probe that selectively binds ADP over ATP^28^ (Figure 2B), and several other groups have invested lactate^29^ and phosphorylated amino acids^30^ through this analyte sensor approach.^28^ Zondlo and co-workers developed terbium-binding protein structures where affinity increased after phosphorylation, resulting in an increase in luminescence (Figure 2C).^31,32^ Of note, [Tb­(bispic)]^+^ has been reported by Maury, Girard, and Riobé^33^ to crystallize with proteins and has since been involved in QM-MM simulations.^34,35^

**Figure 2:**
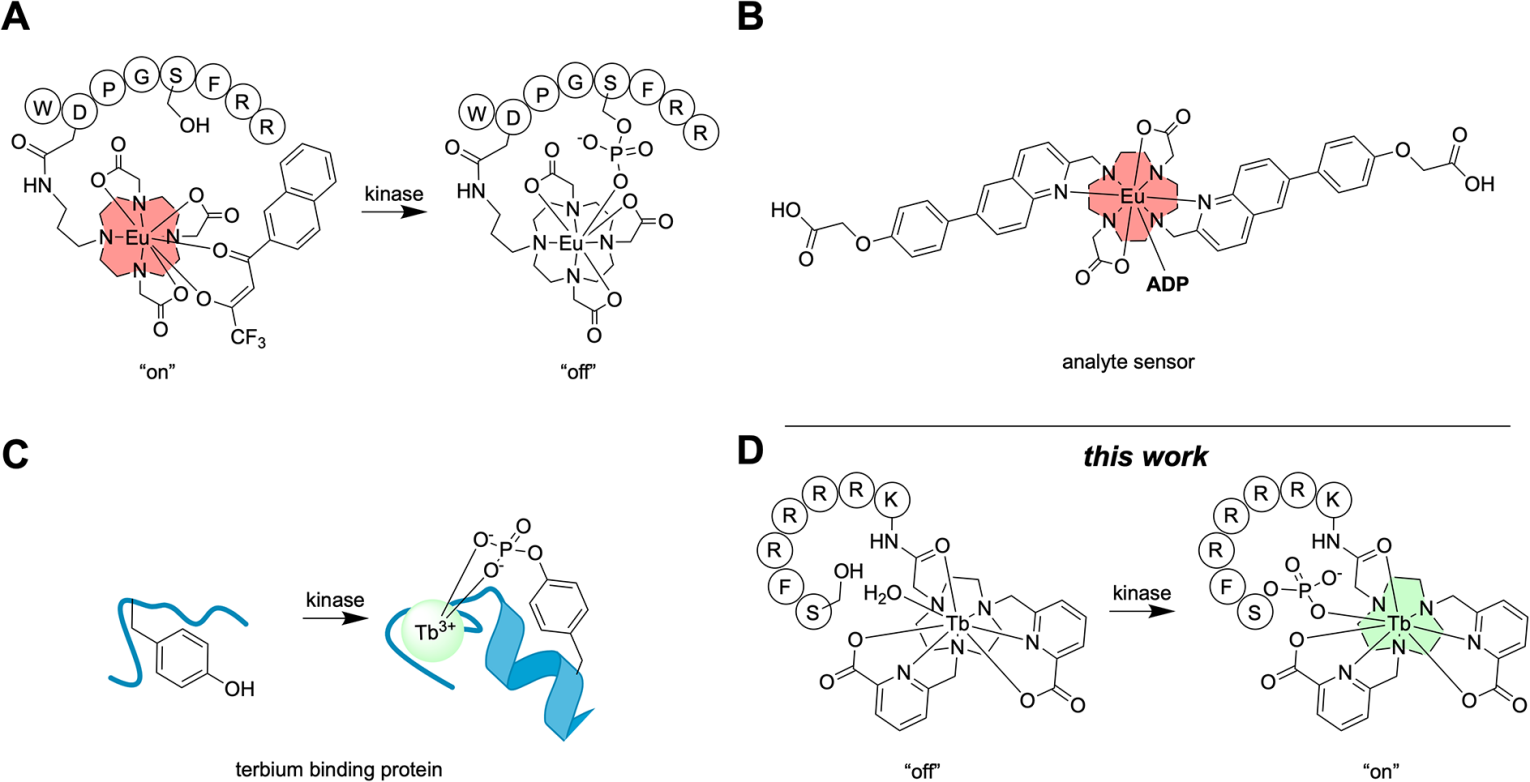
Luminescent lanthanide-based probes have been developed for application in phosphorylation sensing. (A) Europium probe where the sensitizing antenna is displaced after phosphorylation, turning off luminescence.^27^ (B) Europium probe that selectively binds ADP over ATP.^28^ (C) Terbium-binding protein increases emission after phosphorylation.^31^ (D) Present work: the displacement of an inner sphere water following phosphorylation turns on luminescence.

However, many kinase-sensing lanthanide complexes are incompatible with cellular and in vivo environments due to their sensitivity to transchelation by other competing metal ions in solution. A pM value of 13 or above is indicative of diminishing sensitivity to dechelation.^36^ We hypothesized that if a kinetically inert, 8-coordinate lanthanide complex can be appended to a kinase-recognized sequence and contains a sufficiently large ternary binding pocket to bind water and phosphorylated serine,^7^ the system would be in vivo compatible. For instance, the pM of **[Tb­(bispic-acetate)]** is 14.9,^38^ and derivatives of this complex have demonstrated high complex inertness.^38^

Furthermore, we proposed that kinase-mediated phosphorylation should result in displacement of the water and luminescence turn-on.^39^ This system could allow for efficient characterization and optimization of the enzymatic phosphorylation prior to the incorporation of ^32^P and subsequently facilitate the synthesis and characterization of the corresponding self-illuminated construct.

### Optimization of the Complex Structure

Previously, we and others have shown that Tb^3+^ and Eu^3+^ chelates with a 1,4,7-triazacyclononane (tacn) base are a versatile platform for the synthesis of water-soluble, thermodynamically stable lanthanide complexes ideal for in vivo applications.^37,38^ To determine if water could be displaced by phosphate and phosphorylated serine selectively, three model Eu^3+^ and Tb^3+^ complexes with inner-sphere hydration (denoted as *q*) from *q* = 0 to *q* = 2 were synthesized following previously established strategies (Figure 3).^37,38^ The complexes were characterized for their quantum yield (φ),^40^ luminescence lifetimes, and inner sphere hydration number (*q*), which matched literature results (Table 1).^37,38^ Inner sphere hydration number was determined by the empirically deduced Horrocks equation, which compares the luminescence lifetimes in D_2_O and H_2_O (eq 1).^41^

1 $$q=A\left[\frac{1}{\tau_{H_{2}O}}-\frac{1}{\tau_{D_{2}O}}\right]-B$$

where $\tau_{H_{2}O}$ is the luminescence lifetime in water, $\tau_{D_{2}O}$ is the luminescence lifetime in D_2_O, A is 1.2 and 5 ms for Eu^3+^ and Tb^3+^ respectively, and B is 0.25 ms^–1^ and 0.06 ms^–1^ for Eu^3+^ and Tb^3+^ respectively.

**Figure 3:**
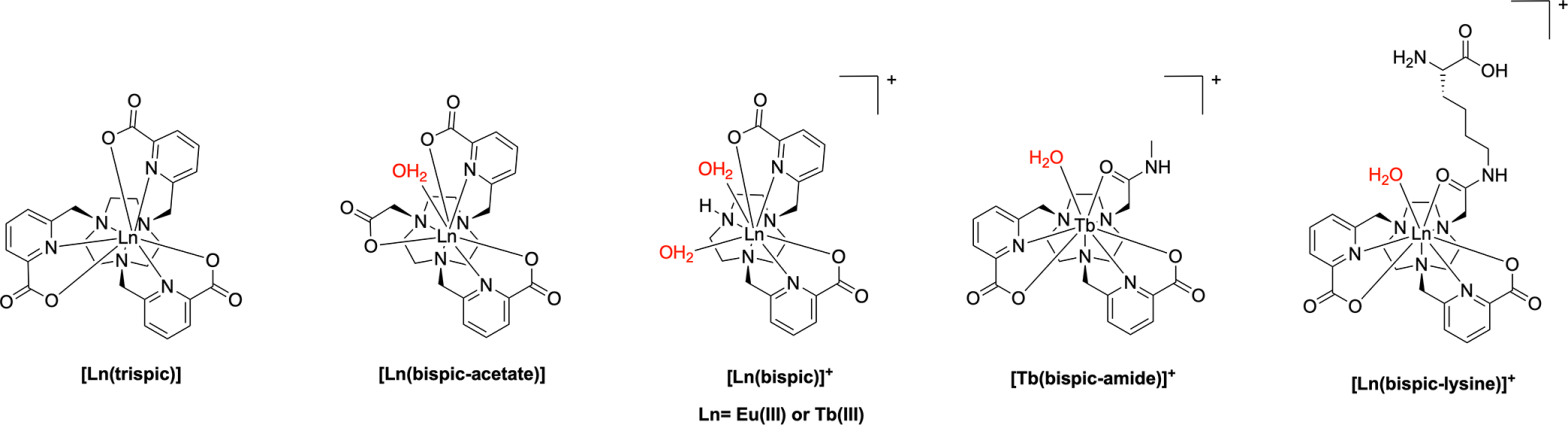
Structure of model complexes.

**Table 1:** Photophysical Characterization of Model Complexes^a^

Complex	φ [%]	τ(H_2_O) [ms]	τ(D_2_O) [ms]	*q*
**[Tb(trispic)]**	71.4 ± 0.7	2.017 ± 0.004	2.176 ± 0.001	–0.1
**[Tb** **(bispic-acetate)** **]**	52 ± 5	1.523 ± 0.002	2.425 ± 0.007	0.9
**[Tb(bispic)]** ^**+**^	30 ± 2	1.137 ± 0.002	2.406 ± 0.004	2.0
**[Tb** **(bispic-amide)** **]** ^**+**^	68 ± 3	1.466 ± 0.001	2.418 ± 0.005	1.0
**[Tb** **(bispic-lysine)** **]** ^**+**^	50 ± 1	1.477 ± 0.004	2.395 ± 0.005	1.0
**[Eu(trispic)]**	9 ± 1	1.083 ± 0.008	1.517 ± 0.006	0.0
**[Eu** **(bispic-acetate)** **]**	4.1 ± 0.1	0.536 ± 0.003	1.558 ± 0.001	1.2
**[Eu(bispic)]** ^**+**^	1.51 ± 0.4	0.611 ± 0.003	1.560 ± 0.001	0.9
**[Eu** **(bispic-lysine)** **]** ^**+**^	2.93 ± 0.07	0.526 ± 0.003	1.531 ± 0.005	1.2

a (ϕ:room temperature, pH 7.4 tris buffer, λ_ex_: 279 nm, reference compound: Ln­(dpa)_3_).^40^ τ­(H_2_O): room temperature, pH 7.4 water, λ_ex_: Tb (285 nm), Eu (274 nm). τ­(D_2_O): room temperature, D_2_O λ_ex_: Tb (285 nm), Eu (274 nm).

Following characterization, luminescence titrations of the Tb^3+^ complexes were performed with phosphate, phosphorylated serine, and serine. The *q* = 0 system, **[Tb­(trispic)]**, did not increase in emission when any of the titrants were added, indicating no change in inner-sphere hydration (Figure 4B). **[Tb­(bispic)]** ^**+**^ (*q* = 2) showed an increase in luminescence in the presence of all three titrants because of a lack of size-selectivity (Figure 4D). The *q* = 1 system, **[Tb­(bispic-acetate)]**, demonstrated a luminescence increase in the presence of phosphate and phosphorylated serine, but not serine. This selectivity identified **[Tb­(bispic-acetate)]** as the most promising scaffold for further evaluation (Figure 4C).

**Figure 4:**
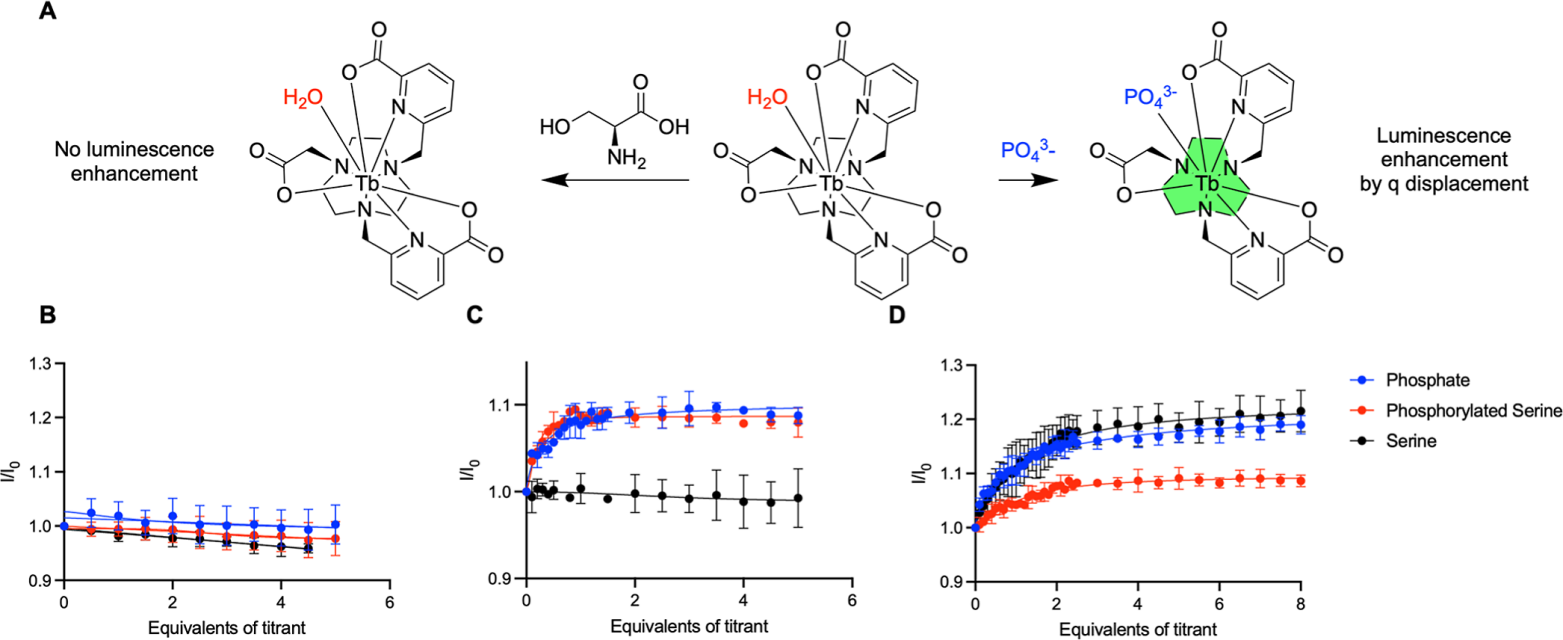
(A) An ideal complex selectively coordinates to phosphate and phosphorylated serine, turning on luminescence, but does not coordinate to serine. Luminescence titrations of model Tb^3+^ complexes (A) **[Tb­(trispic)]** (B), **[Tb­(bispic-acetate)]** (C), and **[Tb­(bispic)]** ^**+**^ (D) with the titrants phosphate, phosphorylated serine, and serine.

Quantum yield calculations were performed with **[Eu­(bispic-acetate)]** in the presence of 10 equiv of *N*-acetyl serine, phosphorylated serine, and phosphate. All three of the titrants resulted in a statistically significant increase in quantum yield, ranging from a 38% increase with phosphate and phosphorylated serine to 42% with *N*-acetyl serine. This lack of selectivity is attributed to the larger relative ionic radius of Eu^3+^ versus Tb^3+^, which increases the size of the ternary ligand binding site (Figure S33 and Table S5).

### Cerenkov Radiation Energy Transfer of Tb^3+^ Complexes

To determine whether the increase in luminescence observed with luminescence titrations and quantum yields after the addition of phosphate could be quantified with an in situ excitation source, phantom image experiments were conducted using ^68^Ga. The complexes were excited with 11 μCi, which is a typical quantity used for in vivo PET imaging. A dilution series ranging from 5 to 25 nmol complex were prepared in 100 mM pH 7.4 HEPES to validate that the model complexes could be efficiently excited and produce detectable emission intensity at relevant compound concentrations using a Cerenkov source. The emission signal intensity was in agreement with the quantum yield calculations where the highest emission signal was **[Tb­(trispic)]** and the lowest emission was from **[Tb­(bispic)]** ^**+**^. At as low as 5 nmol, both **[Tb­(trispic)]** and **[Tb­(bispic-acetate)]** complexes were emissive, motivating further investigation (Figure S27).

### Photophysical Characterization of Model Conjugate Complexes

With evidence that phosphate and phosphorylated serine can selectively displace water, a model-bifunctional chelate **[Ln­(bispic-lysine)]** ^**+**^ was synthesized on Wang resin (Figure 3 and Scheme S2).^42^ Several single amino acid chelators have been synthesized previously, including a tetra-aza-macrocyclic ligand by Sherry and co-workers^43^ as well as a tridentate, lysine-based chelator by Valliant and co-workers,^44^ underscoring the versatility of this approach.

All complexes were characterized for their maximum absorbance (λ_max_), molar extinction coefficients (ε), inner sphere hydration number (*q*), luminescence lifetimes (τ), and quantum yield measurements (ϕ) (Table 1). The ability for phosphate, phosphorylated serine, and serine to displace water was probed with luminescence titrations for **[Tb­(bispic-amide)]** ^**+**^ and quantum yield calculations with 10 equiv of the titrant for the **[Ln­(bispic-lysine)]** ^**+**^ complexes. **[Tb­(bispic-amide)]** ^**+**^ showed a luminescence increase with all three titrants. The **[Tb­(bispic-lysine)]** ^**+**^ quantum yields did not show a significant increase in the presence of any of the three titrants (Figure S32 and Table S4). **[Eu­(bispic-lysine)]** ^**+**^ showed a selective increase in quantum yield with phosphorylated serine and did not show a significant increase with *N*-methyl serine (3.3% and 2.9% respectively) (Figure S31 and Table S3). This increase observed with Eu^3+^ but not with Tb^3+^ is attributed to the lower energy, red emission of Eu^3+^ being more easily quenched than the green emission of Tb^3+^, which is generally considered less sensitive to the O–H oscillator quenching. The selectivity of **Eu­(bispic-lysine)** that is not seen with **Eu­(bispic-acetate)** is attributed to the larger arm size of lysine compared acetate. Additional quantum yield measurements were performed with 10 equiv of CN^–^, *O*-phospho-l-serine, *O*-phospho-d-serine, F^–^, and CO_3_^2–^; each of these anions resulted in a statistically significant increase in luminescence, except for CN^–^, indicating that the complex may also bind other coordinating anions within the ternary binding site (Figure S31 and Table S3).

### Synthesis and Optimization of a Kinase Recognition Sequence

The AcHN-SFRRRRK-CONH_2_ sequence was identified as compatible with phosphorylation by PKCα, a serine and threonine phosphokinase.^45^ Of note, the pendant lysine is not part of the recognition sequence and was incorporated to allow for the introduction of the previously characterized **[Ln­(bispic-lysine)]** ^**+**^ complex. To determine the impact of linker length and rigidity on kinase activity, two peptide sequences incorporating three additional glycine or proline residues, as well as the phosphorylated serine analogues of each peptide, were also synthesized (Figure 5).^27,46^

**Figure 5:**
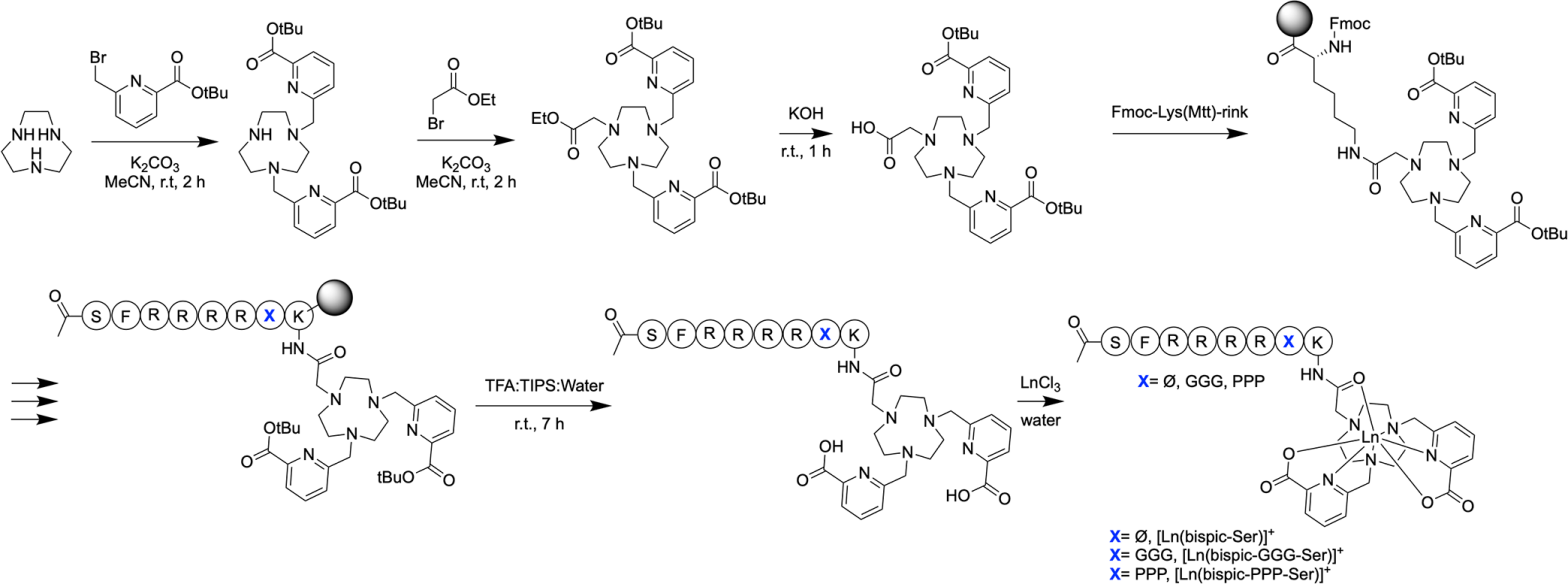
Schematic description of the chemical synthesis of peptide-linked complexes incorporating a kinase recognition sequence.

Photophysical characterization indicated that the quantum yield and luminescence lifetimes were in good agreement with those of the **[Ln­(bispic-lysine)]** ^**+**^ model complexes. The linker did not alter the quantum yields (Tb^3+^: 40–70%; Eu^3+^: 1.3–7%, Table 2).

**Table 2:** Photophysical Characterization of Peptide-Containing Complexes^a^

Complex	φ [%]	*q*
**[Tb** **(bispic-Ser)** **]** ^**+**^	55 ± 1	0.8
**[Tb** **(bispic-PSer)** **]** ^**–**^	70 ± 20	0.0
**[Eu** **(bispic-Ser)** **]** ^**+**^	3.2 ± 0.8	1.1
**[Eu** **(bispic-PSer)** **]** ^**–**^	3.9 ± 0.6	0.8
**[Tb** **(bispic-GGG-Ser)** **]** ^**+**^	44 ± 9	1.0
**[Tb** **(bispic-GGG-PSer)** **]-**	57.2 ± 0.9	0.4
**[Eu** **(bispic-GGG-Ser)** **]** ^**+**^	2 ± 1	1.1
**[Eu** **(bispic-GGG-PSer)** **]-**	2 ± 1	0.7
**[Tb** **(bispic-PPP-Ser)** **]** ^**+**^	40 ± 3	1.0
**[Tb** **(bispic-PPP-PSer)** **]** ^**–**^	50 ± 20	0.8
**[Eu** **(bispic-PPP-Ser)** **]** ^**+**^	1.3 ± 0.5	1.2
**[Eu** **(bispic-PPP-PSer)** **]** ^**–**^	7 ± 1	0.8

a (λ_ex_: 279 nm, reference compound for ϕ: Ln­(dpa)_3_).^17^

All serine-containing peptide complexes produced luminescence data consistent with one inner-sphere water (*q* = 1–1.2). While each phosphorylated peptide showed data consistent with a decrease in q, the greatest reduction in inner-sphere hydration was observed for the shortest peptide sequence chelate **[Tb­(bispic-PSer)]** ^**–**^ (*q* = 0), whereas the other phosphorylated derivatives showed a decrease of inner-sphere hydration by 0.2–0.6 (Table 2). The decrease in the hydration number correlated to an increase in the luminescence quantum yield (Table 2). Taken together, the decrease in inner-sphere hydration indicated the displacement of the inner sphere aqua ligand with the phosphate of the phosphorylated serine, forming an intramolecular metallacycle. An increase in linker length and rigidity resulted in smaller quantum yield and hydration number differences, indicating that the complex may exist as an equilibrium between the open/“linear” and “metallacyclized” state. This large difference in the q value is attributed to the longer and more rigid peptides holding the phosphate farther from the metal center, making them less likely to cyclize.

To further probe the solution structure of the three peptides, we conducted spectroscopic studies with Eu^3+^ complexes. Eu^3+^ is a known NMR shift reagent, where complexation results in large chemical shift changes and/or modification of the proton relaxation time, resulting in a disappearance of signals in the immediate vicinity of the paramagnetic center.^47^ NMR data of the phosphorylated ligands and corresponding Eu^3+^ complexes were compared: the HSQC plots of **[Eu­(bispic-Pser)]** ^**–**^ and **bispic-Pser** are shown in Figure 6A. Indeed, we observe the disappearance of the characteristic methylene signal 1 of the phosphorylated serine. In contrast, the overlay of **[Eu­(bispic-PPP-PSer)]** ^**–**^ and **bispic-PPP-PSer** (Figure 6B) retains signal 1, indicating that the Eu^3+^ center is not found in its immediate vicinity. For both constructs, signals 2, 3, and 4, corresponding to protons on the chelate, are readily suppressed, evidencing the short distance to the paramagnetic metal center. Additionally, Lu^3+^ complexes were synthesized, resulting in a shift of signals indicating proximity to the diamagnetic center (Figures S82 and S83).

**Figure 6:**
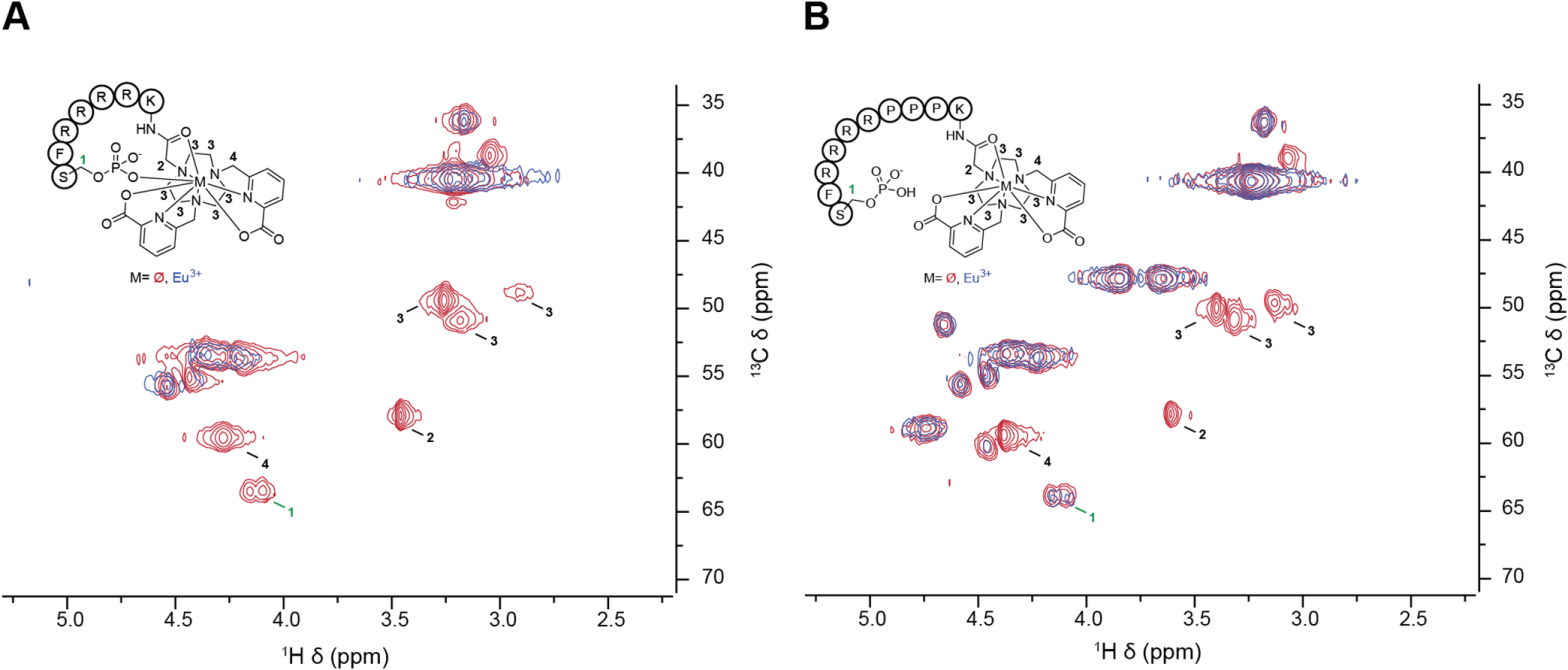
^1^H–^13^C HSQC NMR comparing (A) bispic-PSer (red) to **[Eu­(bispic-PSer)]** ^**–**^ (blue) and (B) bispic-PPP-PSer (red) to **[Eu­(bispic-PPP-PSer)]** ^**–**^ (blue).

### Computational Modeling of the Metallacyclized Structure

In the absence of our ability to obtain X-ray-quality crystals, we conducted in silico modeling and MD simulations to investigate the coordination environment of the proposed peptide–lanthanide complexes. Specifically, we modeled both the nonphosphorylated **([Tb­(bispic-GGG-Ser)]** ^**+**^) and phosphorylated **([Tb­(bispic-GGG-PSer)]** ^**–**^) systems. As no crystal structure was available for **[Tb­(bispic-amide)]** ^+^, we used the published structure of **[Tb­(trispic)]** as a template^37^ and introduced the necessary structural modifications to generate the bispic-amide scaffold, followed by assembly of the peptide sequences (Figure 5). In both systems, the terbium ion (Tb^3+^) forms eight coordinative bonds: seven with the bispic ligand and one with either the side-chain oxygen atom of serine (Ser) or the phosphate oxygen of phosphorylated serine (PSer).

To evaluate the stability of the Tb-Ser/PSer coordination, we performed MD simulations using the Li–Merz force field parameters,^48^ which employ a 12–6–4 Lennard-Jones potential specifically optimized for highly charged ions. Each system was simulated with 20 independent MD trajectories, each spanning 200 ns. To quantitatively compare the hydration environment, we computed the average number of water molecules coordinating Tb^3+^ (using a 5 Å cutoff between the water oxygen and the metal center) over the final 100 ns of all trajectories. As summarized in Table S6, the Ser system averages 0.90 ± 0.15 coordinating water molecules, whereas the PSer system averages only 0.10 ± 0.03, consistent with the experimental findings. These results collectively support that phosphorylation induces the formation of a stable, metallacyclized state by establishing a strong and persistent Tb-phosphate coordination bond, whereas the nonphosphorylated complex remains in a dynamic, noncyclized state due to the lack of a stable coordination interaction. Representative trajectories (Figure 7A–C) further illustrate this difference: in the phosphorylated PSer system, the Tb^3+^-phosphate coordination remains stable throughout the simulation, while in the Ser system, the Tb^3+^-Ser bond is rapidly lost, and a water molecule replaces Ser as the ligand in the metal’s coordination sphere (Figure 7D).

**Figure 7:**
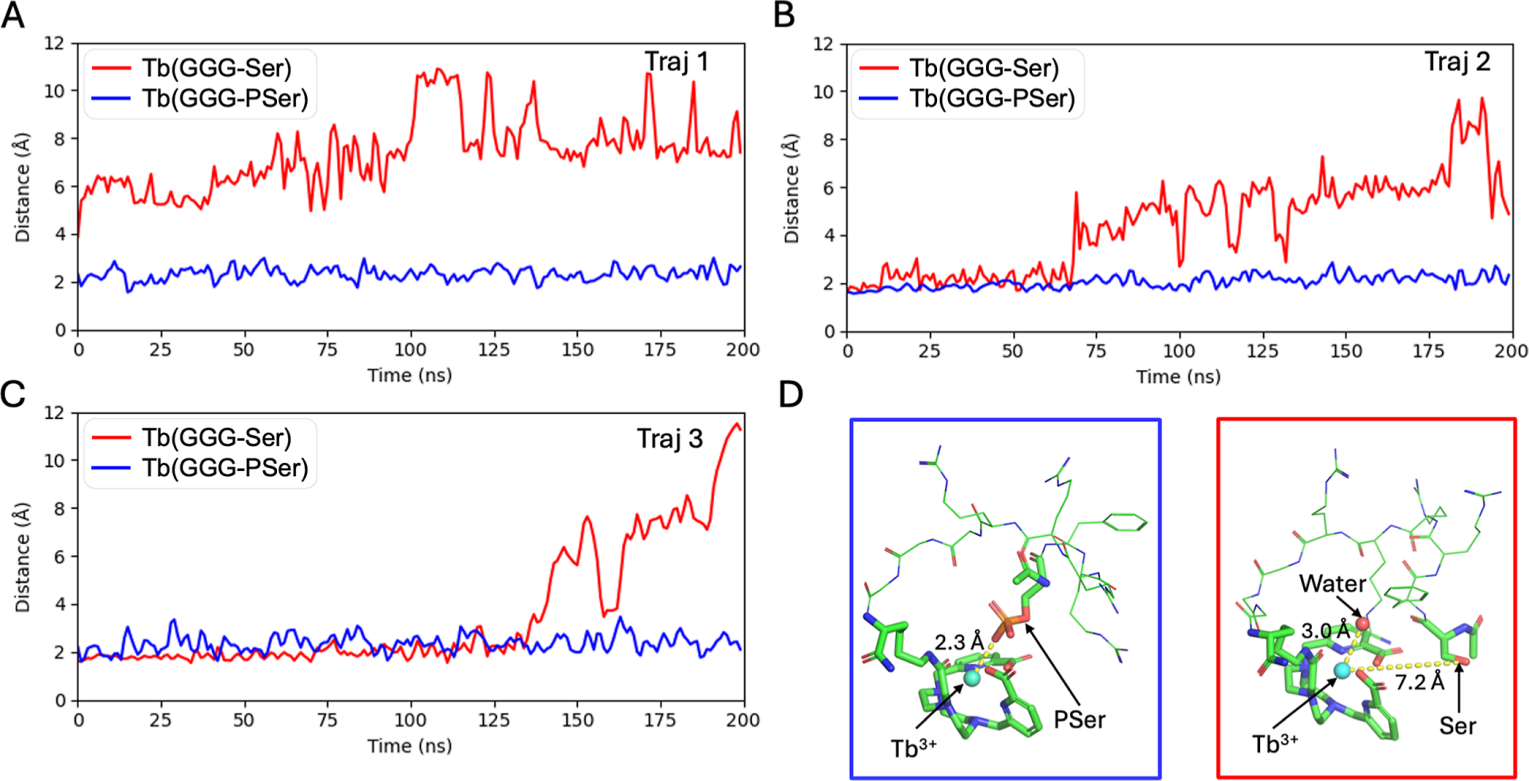
(A–C) Time evolution of the distance between the Tb^3+^ ion and the side-chain oxygen atom of Ser (red) or the phosphate oxygen of PSer (blue) for three representative MD trajectories out of a total of 20 MD trajectories (D) Representative final structures at 200 ns from Traj 1. In the PSer complex (left, blue box), the phosphate oxygen remains tightly coordinated to Tb^3+^. In the Ser complex (right, red box), the Ser side-chain oxygen is dissociated from the metal, and a water molecule occupies the coordination site.

To examine the impact of other linker sequences, we also performed MD simulations for **[Tb­(bispic-Ser/PSer)]** ^**+**^ and **[Tb­(bispic-PPP-Ser/PSer)]** ^**+**^. For each of these two linker sequences, we built both phosphorylated and nonphosphorylated complexes. Twenty independent 200 ns MD trajectories were performed for each complex, and the number of water molecules coordinating Tb^3+^ was averaged over the last 100 ns of all trajectories (Table S6). For the Ser peptides, the average number of coordinating water molecules was 0.88 ± 0.17 for **[Tb­(bispic-Ser)]** ^**+**^ and 0.87 ± 0.20 for **[Tb­(bispic-PPP-Ser)]** ^**+**^. In contrast, for the phosphorylated PSer systems, the numbers decreased substantially to 0.08 ± 0.02 for **[Tb­(bispic-PSer)]** ^**–**^ and 0.11 ± 0.03 for **[Tb­(bispic-PPP-PSer)]** ^**–**^. These results reveal trends comparable to those observed with the GGG linker, indicating that the phosphorylation of Ser enables the formation of a stable coordination structure with Tb^3+^ across all linker types.

To further assess the thermodynamic stability of these complexes, we performed MM-PBSA calculations to estimate the binding affinities between Tb^3+^ and Ser/PSer residues in each sequence. In the Ser systems, the binding affinities were weak across all three linkers (**[Tb­(bispic-Ser)]** ^**+**^: −93.22 ± 42.12 kJ/mol; **[Tb­(bispic-GGG-Ser)]** ^**+**^: −85.01 ± 45.37 kJ/mol; and **[Tb­(bispic-PPP-Ser)]** ^**+**^: −88.74 ± 31.75 kJ/mol). Upon phosphorylation, the binding affinity increased dramatically (**[Tb­(bispic-PSer)]** ^**–**^: −1218.92 ± 37.22 kJ/mol; **[Tb­(bispic-GGG-PSer)]** ^**–**^: −1109.83 ± 56.50 kJ/mol; and **[Tb­(bispic-PPP-PSer)]** ^–^: −1059.15 ± 68.79 kJ/mol), with the **[Tb­(bispic)-PSer]** ^**–**^ system showing the highest affinity. Notably, the flexible GGG linker and the rigid PPP linker both reduced the binding strength relative to the bispic-only sequence, indicating that linker dynamics influence the stability of the metallacyclized state.

Together, these modeling and simulation results provide support for the experimental observation that displacement of Ser for PSer promotes stable metallacycle formation through direct coordination of the phosphate to Tb^3+^.

### Enzymatic Phosphorylation of **[Ln­(bispic-pep)]** ^**+**^

We next probed if the enzymatic phosphorylation of the Ser-containing chelates resulted in an observable luminescence turn on.

To this end, luminescence was monitored using the highest intensity emission bands (Tb^3+^ = 545 nm, ^5^D_4_-^7^F_5_, Eu^3+^ = 616 nm, ^5^D_0_-^7^F_2_) for 10 min after the addition of all of the reagents, including adenosine trisphosphate (ATP) except the PKCα enzyme. Addition of all reagents resulted in no change of the luminescence intensity, indicating that ATP and phosphatidyl serine were not coordinating to the metal center. Subsequent addition of PKCα to the reaction mixture resulted in an increase of the observed emission for Eu^3+^ and Tb^3+^ complexes that plateaued within 10 min (Figures 8, S34, S36, and S38). Subsequent mass spectrometric analysis indicated that the conversion to the phosphorylated complex was complete (Figures S35, S37, and S39).

**Figure 8:**
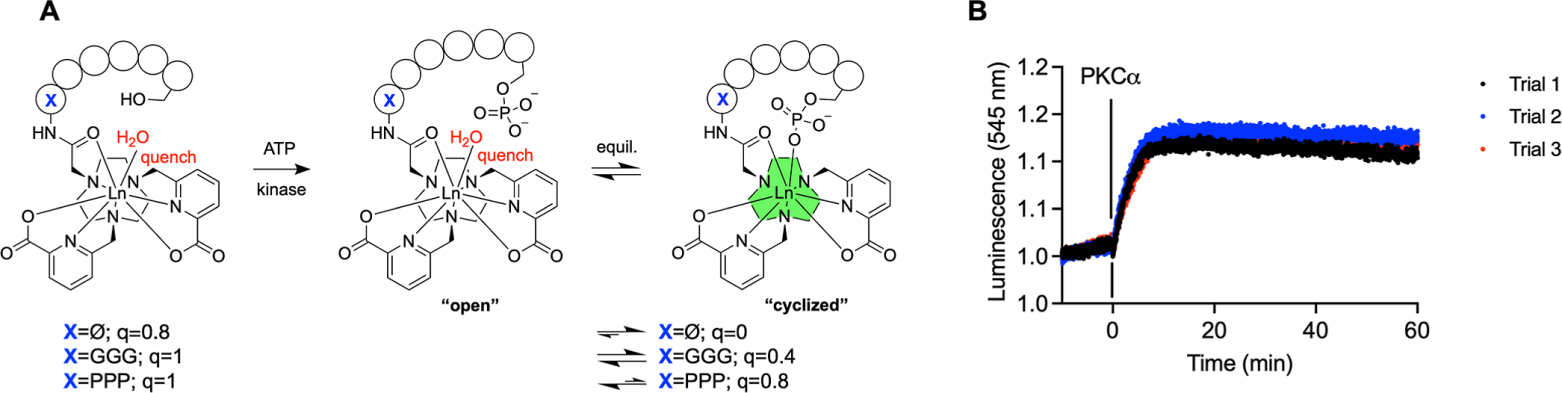
(A) Schematic of the enzymatic phosphorylation of the peptide-containing complexes with *q* values for Tb^3+^ complexes. (B) Luminescence at peak emission wavelength for **[Tb­(bispic-Ser)]** ^**+**^ (545 nm) tracking phosphorylation with the kinase, PKCα (200 μL total volume, 20 mM HEPES, 2.5 mM MgCl_2_, 0.225 mM CaCl2, 500 μM ATP, 1 mM DTT, 0.1 μg of phosphatidylserine, 0.02 μg of diacylglycerol, 20 mM peptide, pH 7.4, 0.136 μg of PKCa).

Of note, the luminescence intensity of the phosphorylation product of **[Ln­(bispic-Ser)]** ^**+**^ remained elevated, whereas time-dependent luminescence measurements of **[Tb­(bispic-GGG-PSer)]** ^**–**^ and **[Tb­(bispic-PPP-PSer)]** ^**–**^ showed a decrease of luminescence intensity over time, even though the mass spectrometric analysis demonstrated that the phosphorylated complex species was stable and did not hydrolyze or dephosphorylate under these conditions (Figures S37 and S39). These results further support the hypothesis that the GGG and PPP linked peptides exist in an equilibrium between the metallacyclized and linearized or open state.

### Enzymatic Radiolabeling and Imaging of **[Tb­(bispic-Ser)]** ^**+**^ with [γ-^32^P]-ATP

In order to construct the self-illuminated, enzymatically phosphorylated Tb chelate, we sought to incorporate the beta-emitting isotope ^32^P within the **[Tb­(bispic-Ser)]** ^**+**^ peptide sequence.

While [γ-^32^P]-ATP has traditionally been used to phosphorylate larger peptides and proteins, it has not been used to radiolabel and isolate ^32^P labeled substrates for further studies. The radiochemical labeling of **[Tb­(bispic-Ser)]** ^**+**^ with 50 μCi [γ-^32^P]-ATP was performed at 37 °C under identical reagent conditions to the enzymatic phosphorylation with nonradioactive ATP discussed above. The reaction was monitored by radio-HPLC and scintillation counting of fractions to reconstruct the corresponding radiochromatographic trace. Figure 9B shows comparative UV chromatograms of the [γ-^nat^P]-ATP and **[Tb­(bispic-** ^**nat**^ **PSer)]** ^**–**^ complex. Reconstruction of the radiochromatographic trace for **[Tb­(bispic-** ^32^ **PSer)]** ^**–**^ shows a good match of retention time with the desired, nonradioactive species; the delay of the radioactive signal by ∼0.5 min is due to the sequential setup of the UV detector and eluent collection at the end of the line. Quantitation of chromatographic results indicated that the enzymatic phosphorylation reaction produced a radiochemical yield and a purity of 95% (Figures 9A,B and S26), indicating that no additional purification was required prior to imaging experiments and underscoring the efficiency of enzymatic radiophosphorylation. Furthermore, the product remained hydrophilic and could be readily formulated for additional experiments, effectively overcoming the limitations of the previously employed, intramolecular CRET systems.

**Figure 9:**
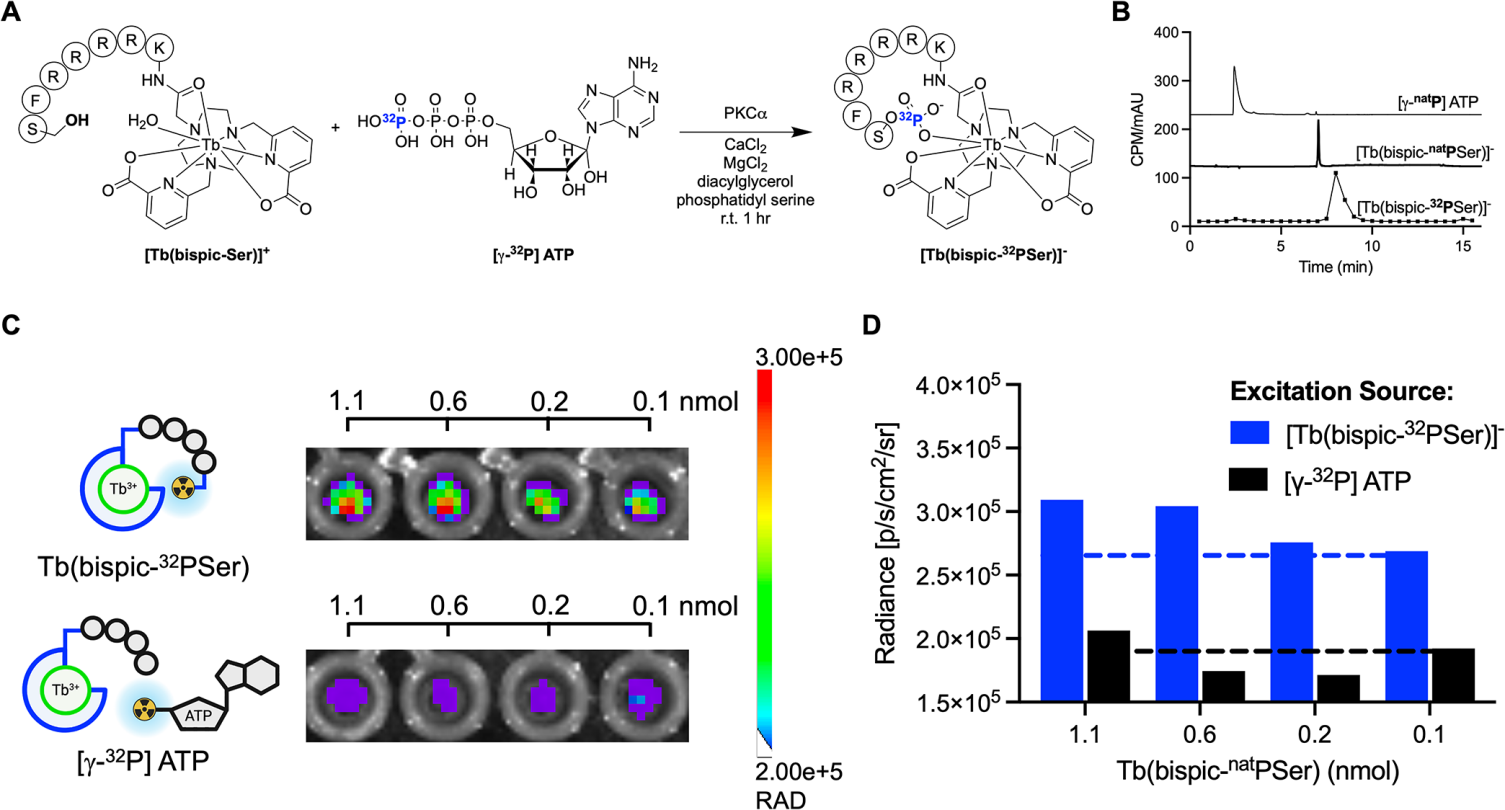
(A) Enzymatic radiolabeling with [γ-^32^P] ATP to form **[Tb­(bispic-^32^PSer)]** ^**–**^. (B) HPLC chromatograms of ATP, **[Tb­(bispic-** ^**nat**^ **PSer)]** ^**–**^, and **[Tb­(bispic-^32^PSer)]** ^**–**^. (C) Phantom image comparing intramolecular (**[Tb­(bispic-^32^PSer)]** ^**–**^ **)** and intermolecular ([γ-^32^P] ATP) Cerenkov excitation of **[Tb­(bispic-PSer)]** ^**–**^(10 μCi/well; 540 nm filter, 200 μL total volume). (D) ROI analysis comparing the excitation of **[Tb­(bispic-PSer)]** ^**–**^ with **[Tb­(bispic-^32^PSer)]** ^**–**^ and [γ-^32^P]-ATP (10 μCi/well; 540 nm filter, 200 μL total volume).

A phantom imaging experiment with a small animal imaging scanner was used to compare the signal intensity achieved by the intramolecular CRET system **[Tb­(bispic-^32^PSer)]** ^**–**^, when compared with the corresponding, intermolecular CRET system. The intermolecular CRET samples were composed of a mixture of nonradioactive **[Tb­(bispic-** ^**nat**^ **PSer)]** ^**–**^ excited by [γ-^32^P]-ATP, without establishing a covalent bond to the Tb-complex peptide. Two dilution series ranging from 0.2 to 1.1 nmol **[Tb­(bispic-** ^**nat**^ **PSer)]** ^**–**^ were prepared and doped with either [γ-^32^P]-ATP or **[Tb­(bispic-^32^PSer)]** ^**–**^. By investigating the emission of **[Tb­(bispic-** ^**nat**^ **PSer)]** ^**–**^ excited by either [γ-^32^P]-ATP (intermolecular) or **[Tb­(bispic-^32^PSer)]** ^**–**^ (intramolecular), a direct comparison can be made between intermolecular and intramolecular CRET. The samples were imaged using the 540 nm emission filter, centered on the Tb emission band, and the limit of detection (LOD) was determined by comparison to the background (0.1 nmol because of the molar activity of the radiolabeling). Image quantification indicated a limit of detection (LOD) of 1.1 nmol for intermolecular CRET, whereas the intramolecular, self-illuminated **[Tb­(bispic-^32^PSer)]** ^**–**^ system showed a limit of detection of 0.2 nmol, which is a 5.5-fold improvement compared to the intermolecular system and the lowest LOD for any CL-excited lanthanide system to date (Figure 9C,D).

## Conclusions

The present work introduces enzymatic radiophosphorylation as a viable strategy to produce self-illuminated lanthanide coordination complexes. In addition to producing an efficiently sensitized lanthanide complex, the kinase-responsive peptide forms a metallacyclized structure, resulting in an additional luminescence turn-on response following enzymatic phosphorylation. The enzymatic phosphorylation of the Tb^3+^ chelate was successfully reproduced using [γ-^32^P]-ATP, and subsequent optical imaging revealed the intramolecular **[Tb­(bispic-^32^PSer)]** ^**–**^ has a limit of detection as low as 0.2 nmol. A series of peptides were compared to determine the optimal length and rigidity for binding to Tb^3+^. Together, this work demonstrates that enzymatic radiophosphorylation represents a viable approach to synthesizing and evaluating intramolecular CRET probes for prospective in vivo applications.

## Experimental Methods

All starting materials were purchased from commercial sources and were not purified further. NMR spectra (^1^H, ^13^C) were collected on a Bruker Avance-500 MHz with a DCH cryoprobe or a Bruker Avance 400 MHz instrument in deuterated solvents at 298 K; chemical shifts (δ) in ppm relative to residual solvent resonances (CDCl_3_^1^H: δ7.26; CD_3_CN ^1^H: δ 1.96; MeOD ^1^H: δ 3.31); coupling constants (J) in Hz. Signal assignments are based on coupling constants, increment calculations, and/or 2D NMR experiments. Data were processed using TopSpin 4.1.4. Chemical shifts are reported as parts per million (ppm).

## Computational Methods

### Force Field Parameters for Lanthanide (Tb-bispic)

The force field parameters for lanthanide were derived based on the second generation of the General Amber Force Field^9^ (GAFF2). To obtain atomic charges, ligand geometry optimizations, and electrostatic potential (ESP) calculations were performed with Gaussian 16.^10^ Geometry optimizations were carried out using the B3LYP^11,12^ 11/17/25 1:25:00 PM functional with Grimme’s D3BJ dispersion correction,^13^ applying the 6-311G** basis set to nonmetal atoms, while Tb^III^ was treated with MWB54 ^14^ effective core potential (ECP). Implicit solvation was modeled using the polarizable continuum model^15^ (PCM) with water as the solvent. ESP calculations were performed at the Hartree–Fock (HF) level of theory using the MWB54 ECP for Tb^III^ and the 6-31G* basis set for all other atoms in the gas phase. Partial atomic charges were derived using the Sobtop^16^ program, with the charge of Tb restrained to +3 during the fitting procedure. The Tb ion was described with the Li–Merz 12–6–4 Lennard–Jones parameters,^17^ which have been optimized for highly charged metal ions.

### Molecular dynamics Simulations for Peptide–Lanthanide Complexes

Because of the lack of available crystal structures for the Tb­(bispic-amide) complex, the initial coordinates for modeling were generated based on the crystal structure of Tb­(trispic) (CCDC: 729999).^5^ The trispic ligand was mutated in silico to generate the bispic scaffold, after which peptide sequences (Ser or PSer variants, with GGG and PPP linkers as appropriate) were appended using PyMOL.^18^ The side chain conformation of serine or phosphorylated serine was manually adjusted to enable initial coordination with the terbium ion. All systems were subsequently subjected to energy minimization to relieve local steric clashes in a vacuum. The systems were then solvated in a cubic box of TIP3P^19^ water molecules with a minimum distance of 15 Å between the solute and the box edge. Na^+^ and Cl^–^ counterions were added to neutralize the systems to a total salt concentration of 0.15 M.

For each system, the following equilibration steps were performed. First, energy minimization was performed using the steepest descent algorithm and the conjugate gradient method. Second, a two-step equilibration simulation was carried out. The system was first heated from 0 to 300 K with the position restraints (with a force constant of 1000 kJmol^–1^ nm^–2^) over 1 ns in the *NVT* ensemble. Subsequently, the system was then equilibrated under NPT conditions at 1 bar for 1 ns with the same position restraints. Finally, 20 × 200 ns production simulations were conducted for each system within the *NPT* ensemble at 300 K and 1 atm using periodic boundary conditions. The temperature and pressure were maintained using a Langevin thermostat^20^ and a Monte Carlo barostat,^21^ respectively. Electrostatic interactions were calculated with a distance cutoff of 12 Å using the particle mesh Ewald (PME)^22^ method. The SHAKE^19^ algorithm was used to maintain all constraints for bonds involving hydrogens, and the time step was set to 2.0 fs. All simulations were performed using the Amber24 ^23^ suite, pmemd.cuda module, and Amber14sb^24^ force field.

## Supplementary Material



## Notes

Information on the experimental procedures for synthesis, radioactive studies, and luminescence studies and characterization methods including NMR, mass spectrometry, and HPLC characterization (PDF)
