# Supplementary material for: Accessing Self-Illuminated, Luminescent Lanthanide Probes by Enzymatic Radiophosphorylation
Source: Inorg Chem. 2025 Dec 12;64(51):25270–80. doi: 10.1021/acs.inorgchem.5c04556 (PMC12754790; doi:10.1021/acs.inorgchem.5c04556)
Supplement: Supplementary file 1 [file ic5c04556_si_001.pdf]

## Supporting Information

# Accessing Self-Illuminated, Luminescent Lanthanide Probes by Enzymatic Radiophosphorylation

Georgia G. Sands, Yichong Lao, M. Andrey Joaqui Joaqui, Xuhui Huang, and Eszter Boros*
Department of Chemistry, University of Wisconsin-Madison, 1101 University Avenue, Madison, WI 53706, USA
*Correspondence: eboros@wisc.edu

## Table of Contents

*Part I - Background* .......... 3

*Part II - Experimental* .......... 3

General .......... 3

Synthesis .......... 5
2.1 Synthesis of $[Tb(bispic\text{-}amide)]^+$ .......... 5
2.2 Synthesis of $[Ln(bispic\text{-}lysine)]^+$ .......... 9
2.3 Synthesis of [Ln(trispic)] .......... 15
2.4 Synthesis of [Ln(bispic-acetate)] .......... 16
2.5 Synthesis of $[Ln(bispic)]^+$ .......... 18
2.6 Synthesis of $[Ln(bispic\text{-}Ser)]^+$ .......... 19
2.7 Synthesis of Ln(bispic-peptides) .......... 24

Radioactive and Optical Imaging Experiments .......... 35
3.1 Radiochemical Synthesis Protocols .......... 35
3.2 Phantom Image Assays .......... 36

Luminescence Studies .......... 39
4.1 Luminescence Titrations .......... 39
4.2 Eu(bispic-lysine) Quantum Yield Comparisons .......... 39
4.3 Tb(bispic-lysine) Quantum Yield Comparisons .......... 40
4.4 Eu(bispic-acetate) Quantum Yield Comparisons .......... 40
4.5 Phosphorylation with PKCα of $[^{nat}Ln]$[(Ln(bispic-peptides)] .......... 41
4.6 Water Lifetime Experiments .......... 43
4.7 $D_2O$ Lifetime Experiments .......... 50

NMR Experiments .......... 57
5.1 Bispic-PSer .......... 58
5.2 Bispic-PPP-PSer .......... 59
5.3 Bispic-GGG-PSer .......... 60

Computational Methods .......... 61
6.1 Water coordination and binding affinities for $Tb^{3+}$ complexes with different linker peptides .......... 61

*Part III - Spectra* .......... 61

Synthesis of $[Ln(bispic\text{-}amide)]^+$ .......... 62
S1 .......... 62
S2 .......... 65
S3 .......... 69

Synthesis of $[Ln(bispic\text{-}lysine)]^+$ .......... 73
S4 .......... 73

**S5** .......... **77**
**S7** .......... **80**
**S8** .......... **84**
**[Tb(bispic-lysine)]$^+$** .......... **87**
**[Eu(bispic-lysine)]$^+$** .......... **88**
**[Eu(bispic)]$^+$** .......... **91**

**Synthesis of [Ln(bispic-Ser)]$^+$** .......... 93
**S10** .......... **93**
**S11** .......... **93**
**[Eu(bispic-Ser)]$^+$** .......... **95**
**[Tb(bispic-Ser)]$^+$** .......... **96**

**Synthesis of [Ln(bispic-Peptides)]$^-$** .......... 97
**S12** .......... **97**
**S13** .......... **101**
**S14** .......... **104**
**S16** .......... **108**
**S17** .......... **112**
**Synthesis of [Eu(bispic-PSer)]$^-$** .......... **113**
**Synthesis of [Tb(bispic-PSer)]$^-$** .......... **117**
**Synthesis of [Lu(bispic-PSer]$^-$** .......... **117**
**S18** .......... **122**
**S19** .......... **124**
**S20** .......... **125**
**Synthesis of [Tb(bispic-GGG-Ser)]$^+$** .......... **129**
**Synthesis of [Eu(bispic-GGG-Ser)]$^+$** .......... **129**
**Synthesis of [Tb(bispic-GGG-PSer)]$^-$** .......... **130**
**Synthesis of [Eu(bispic-GGG-PSer)]$^-$** .......... **131**
**Synthesis of [Tb(bispic-PPP-Ser)]$^+$** .......... **133**
**Synthesis of [Eu(bispic-PPP-Ser)]$^+$** .......... **134**
**Synthesis of [Tb(bispic-PPP-PSer)]$^-$** .......... **134**
**Synthesis of [Eu(bispic-PPP-PSer)]$^-$** .......... **135**

**References** .......... 139

## Part I - Background

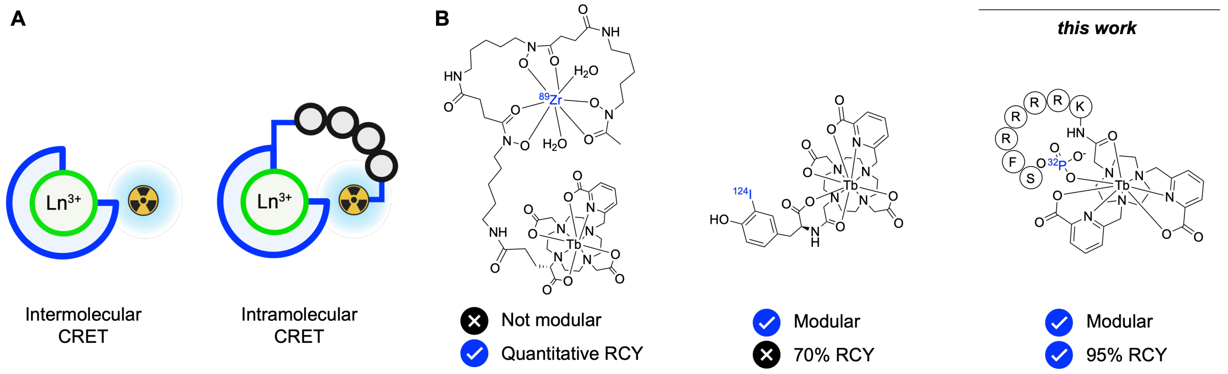


**Figure S1:** (A) intermolecular and intramolecular CRET. (B) Advantages of a series of intramolecular CRET systems. RCY= radiochemical yield.

## Part II - Experimental

### General

All starting materials were purchased from commercial sources and not purified further. NMR spectra ($^{1}$H, $^{13}$C) were collected on a Bruker Avance-500 MHz with a DCH cryoprobe or a Bruker Avance 400 MHz instrument in deuterated solvents at 298 K; chemical shifts (δ) in ppm relative to residual solvent resonances ($CDCl_3$ $^{1}$H: δ7.26; $CD_3CN$ $^{1}$H: δ 1.96; MeOD $^{1}$H: δ 3.31); coupling constants (J) in Hz. Signal assignments are based on coupling constants, increment calculations and/or 2D-NMR experiments. Data was processed using TopSpin 4.1.4. Chemical shifts are reported as parts per million (ppm).

**High resolution ESI mass spectrometry** was carried out at the University of Wisconsin-Madison Department of Chemistry Paul Bender Chemical Instrumentation Center (CIC) using a Thermo Scientific Q Exactive Focus Orbittrap MS system, or at the University of Wisconsin-Madison Biotechnology Center using a Thermo Scientific Oribitrap Elite, or at the University of Wisconsin-Madison School of Pharmacy Thermo Scientific Q Exactive Focus Orbittrap MS system.

**Low resolution liquid chromatography -mass spectrometry (LC-MS)** was carried out on a Phenomenex Luna 5 µm C18 column (150 mm × 3 mm, 100 Å, AXIA packed) at a flow rate of 0.8 mL/min using a single quadrupole Agilent 1200 Infinity II LC/MSD system equipped with a binary gradient pump, UV-vis detector, automatic injector, and an atmospheric pressure electrospray ionization (API-ES) source. UV absorption was recorded at 254 nm, both positive and negative mass spectra were collected. Purity of all intermediates and final products, including radiochemical species, was determined using analytical HPLC. All conjugates and complexes were ≥ 95% pure. LCMS analysis (Method I): **)**: binary solvent system (A: water + 0.1% FA; B: MeCN + 0.1% FA); gradient (0–3 min: 5% B; 3–10min: 5–95% B; 10–12 min: 95% B; 12–12.5 min: 95–5% B; 12.5–16 min 5% B); flow rate: 0.8 mL/min; column: Phenomenex Luna C18 column (5 µm, 150 mm × 3 mm, 100 Å, AXIA packed).

**UV-VIS** spectra were taken on a NanoDrop 1C instrument (AZY1706045). Spectra were recorded from 190-850 nm in a quartz cuvette with a 1 cm path length.

**Luminescence measurements** were collected on a Hitachi F-7100 FL spectrophotometer. Wavelength scans were collected by exciting at the appropriate wavelength for antenna-mediated excitation and minimization of scattering interference. Emission spectra were collected from 400 nm-800 nm with 1200 s scan time, 0.05 s response, and PMT voltage 400 or 700 V.

**ICP-OES analysis** was carried out using an Agilent 5110 inductively coupled plasma optical emission spectrometer. A 6-point standard curve (1-100 ppm) with respect to europium or terbium was used, and fits were found to be $R^2 > 0.99$.

**HPLC** purification and analytical methods were performed using a binary solvent system with solvent A (water + 0.1% FA) and solvent B (MeCN + 0.1% FA). Preparative HPLC was carried out on a Phenomenex Luna C18 column (250 mm × 21.2 mm, 100 Å, AXIA packed) at a flow rate of 30 mL/min using an Agilent 1260 Infinity II equipped with a binary gradient pump, UV-vis detector, and manual injector. UV absorption was recorded at 220 nm and 254 nm. Method A: Gradient: 0–3 min: 5% B; 3–17 min: 5–50% B; 17–21 min: 50–95% B; 21–25 min: 95% B; 25–27 min: 95–5% B; 27–30 min: 5% B. Flash chromatography was carried out using a Combi Flash Rf+ on a RediSep column (100 g HP C18 gold, CV: 87.7 mL, flow rate: 60 mL/min). Method B: Gradient: 1-2 min 10% B; 2-3 min: 10–20% B; 3–19 min: 20–25% B; 19 min: 25-100% B; 19-23 min: 100% B; 23 min: 100-10% B; 23-25 min: 10% B. Analytical HPLC was carried out on a Phenomenex Luna 5 µm C18 column (150 mm × 3 mm, 100 Å, AXIA packed) or a Restek C18 column (250 mm × 3 mm, 5 µm, Ultra AQ) at a flow rate of 0.8 mL/min using a Agilent 1260 Infinity II HPLC equipped with a binary gradient pump, UV-vis detector, autoinjector, and Laura radiodetector. UV absorption was recorded at 254 nm. Method C: Phenomenex Luna column, Gradient: 0–2 min: 5% B; 2–14 min: 5–95% B; 14-16 min: 95% B; 16-16.5 min: 95–5% B; 16.5– 20 min 5% B. Method D: Restek Ultra AQ column, Gradient: 0–3 min: 5% B; 2–8 min: 5–95% B; 8–11 min: 95% B; 11–13 min: 95–5% B; 13– 16 min 5% B. Method E: Phenomenex Luna column, Gradient: 0–3 min: 5% B; 2–8 min: 5–95% B; 8–11 min: 95% B; 11–13 min: 95–5% B; 13–16 min 5% B. Method F: Restek C18 column, Gradient: 0–2 min: 0% B; 2–14 min: 0–95% B; 14-16 min: 95% B; 16-16.5 min: 95–0% B; 16.5– 20 min 0% B. Method G: Restek C18 column, Gradient: 0-2.5 0% B; 2.5-27 min 0-50% B; 27-30 min 50-0% B; 30-33 min 0% B. Method H: Restek C18 column, Gradient: 0-2 min 0% B; 2-24 min 0-90% B; 14-16 min 90% B; 16-16.5 90-0% B; 16.5-20 0% B. Method I: Restek C18 column, Gradient: 0-25 0-50% B; 25-27 min 50-0% B; 27-30 min 0% B.

Analytical HPLC traces have been baseline corrected versus a blank measurement.

**Quantum Yield, Luminescence Lifetime, and Molar Absorptivity Measurements**

Quantum yields were determined with a relative method comparing to $Eu(dpa)_3$ ($\phi$= 13.5% at 279 nm in pH 7.4 0.1 mM tris buffer), and $Tb(dpa)_3$ ($\phi$= 26.5% at 279 nm in pH 7.4 0.1 mM tris buffer)[1] matching the lanthanide metal to the complex with unknown quantum yield[1]. One mL of pH 7.4 0.1 mM tris buffer was added to a quartz cuvette. The complex of interest was added to achieve an absorbance of 0.1 at 279 nm. An emission spectrum was taken with the parameters

described below. The solution was then diluted and the absorbance and emission spectrum repeated. This process was repeated from 0.1-0.03 ABS, and the relationship between absorbance and integrated area of the emission spectrum was determined using simple linear regression in Graph Pad Prism. The quantum yield was determined with the equation:

$$\phi_u = \phi_{st}(\frac{Grad_u}{Grad_{st}}) \qquad \text{Eq. 1}$$

where $\phi_{st}$ is the quantum yield of the reference complex, $Grad_u$ is the slope of the unknown, $Grad_{st}$ is the slope of the reference complex, and $\phi_u$ is the quantum yield of the unknown.

Lifetime values were determined by fitting the luminescent decay curves with the equation:

$$I_t = I_0 \times e^{-\frac{x}{t}} \qquad \text{Eq. 2}$$

where $I_t$ is the initial luminescent emission intensity, $I_0$s the intensity at time x = 0, and τ is the luminescence lifetime. Data was fit using GraphPad Prism. Lifetimes in the presence of phosphate were performed with 10 equivalents of $Na_3PO_4$. Hydration number (q) was calculated using Horrocks' method, eq. 3.

$$q = A[\frac{1}{\tau(H_2O)} - \frac{1}{\tau(D_2O)} - B] \qquad \text{Eq. 3}$$

Where A=1.2 $ms^{-1}$ and B=0.25 for $Eu^{3+}$ and A=5 $ms^{-1}$ and B=0.06 for $Tb^{3+}$.[2,3]

Molar absorptivity for ligands was performed with spectroscopic titrations with $Tb^{3+}$. The formation of the terbium-ligand complex was monitored at 280 nm in a 1 cm path length cuvette with a NanoDrop spectrophotometer. To a solution of HEPES buffer (pH 7.4, 20 mM, 0.449 mL) was added the ligand, bispic-amide (4.06 mM, 19.9 µL). The $Tb^{3+}$ was titrated (10 nmol/10 µL) as determined by ICP-OES. The titration endpoint was determined by the inflection point of the absorbance at 280 nm. The slopes were determined with a simple linear regression in Graph Pad Prism. Molar absorptivity was calculated from the slope of the standard curve usng the Beer-Lamber law.

## Synthesis

### 2.1 Synthesis of [Tb(bispic-amide)]$^+$

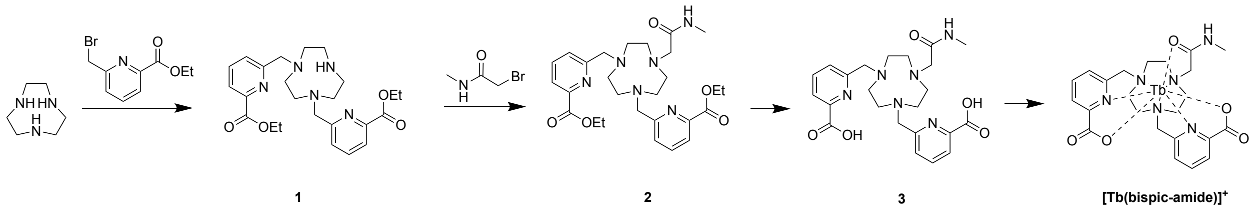


1 2 3 [Tb(bispic-amide)]$^+$

Scheme S1: Synthesis of [Tb(bispic-amide]$^+$.

**Synthesis of 1**

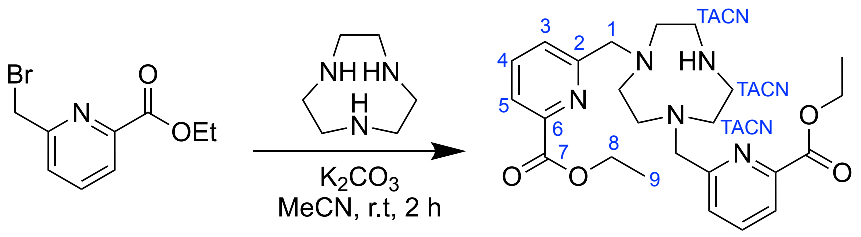


**6-(bromoethyl)-picolinate** **1**

The 6-(bromoethyl)-picolinate was synthesized according to literature procedure.[4] The triazacyclononane (tacn) (4.53 g, 35.08 mmol, 3 eq) was dissolved in 20 mL of dry acetonitrile. Potassium carbonate (4.85 g, 35.08 mmol, 3 eq) was added and the solution was stirred. The 6-(bromoethyl)-picolinate (2.85 g, 11.69 mmol, 1 eq) was dissolved in 5 mL of dry acetonitrile and added to solution dropwise. The solution was allowed to stir under nitrogen for two hours before it was filtered and solvent was removed. The brown oil was purified with flash chromatography (method B) and fractions containing the compound were lyophilized to give a brown oil (448.6 mg, 8.42%).

$^{1}$H NMR (500 MHz, $CD_3CN$) δ 7.85 (t, $J$ = 7.7 Hz, 2H, $H_4$), 7.79 (d, $J$ = 7.8 Hz, 2H, $H_5$), 7.44 (d, $J$ = 8.9 Hz, 2H, $H_3$), 4.48 (s, 4H, $H_1$), 4.28 (q, $J$ = 7.1 Hz, 4H, $H_8$), 3.55 (m, 12H, $H_{TACN}$), 1.32 (t, $J$ = 7.1 Hz, 6H, $H_9$).

$^{13}$C NMR (126 MHz, $CD_3CN$) δ 164.31($C_q$, 2 C, $C_7$), 155.90 ($C_q$, 2C, $C_6$), 146.38 ($C_q$, 2 C, $C_2$), 138.88 (CH, 2 C, $C_4$), 126.58 (CH, 2 C, $C_3$), 124.27 (CH, 2 C, $C_5$), 62.11 ($CH_2$, 2 C, $C_8$), 51.98 ($CH_2$, 2 C, $C_{TACN}$), 50.88 ($CH_2$, 2 C, $C_{TACN}$), 43.35 ($CH_2$, 2 C, $C_{TACN}$), 13.40 ($CH_3$, 2 C, $C_9$).

**HR-ESI-MS:** $[M+H]^+$ = calc. 456.2605 m/z, found: 456.2607 m/z.

**HPLC:** $R_f$ = 6.34 min (Method E)

**Synthesis of 2**

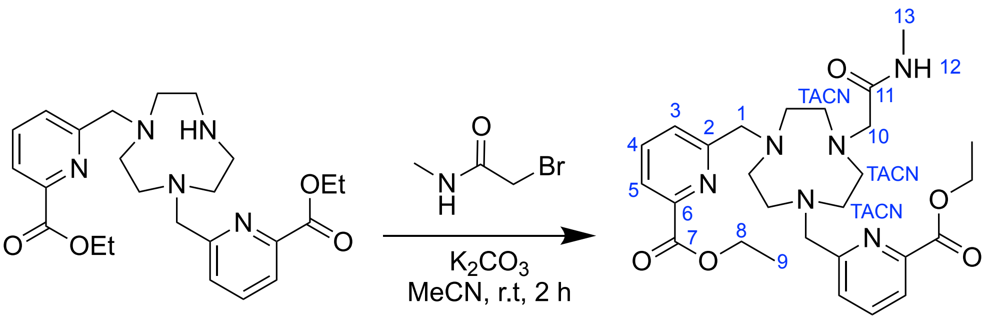


**1** **2**

**1** (90.9 mg, 200 µmol, 0.7 eq) was dissolved in 10 mL of dry acetonitrile. Potassium carbonate (118 mg, 855 µmol, 3 eq) was added and the solution was stirred. The 2-bromo-N-methylacetamide (43.3 mg, 285 µmol, 1 eq) was added to the solution and was allowed to stir under nitrogen for two hours. The reaction was filtered and solvent was removed. The brown oil

was purified with the preparative HPLC (method A) and fractions containing the compound were lyophilized to give a brown oil (40.5 mg, 27%).

$^{1}$H NMR (500 MHz, $CDCl_3$) δ 8.58 (s, 1H, $H_{12}$), 7.93 (d, $J$ = 8.9 Hz, 2H, $H_3$), 7.75 (t, $J$ = 7.7 Hz, 2H, $H_4$), 7.50 (d, $J$ = 7.5 Hz, 2H, $H_5$), 4.37 (q, $J$ = 7.1 Hz, 4H, $H_8$), 3.99 (s, 4H, $H_1$), 3.47 (s, 2H, $H_{10}$), 3.00 (s, 8H, $H_{TACN}$), 2.81 (s, 4H, $H_{TACN}$), 2.71 (d, $J$ = 4.7 Hz, 3H, $H_{13}$), 1.34 (t, $J$ = 7.2 Hz, 6H, $H_9$).

$^{13}$C NMR (126 MHz, $CDCl_3$) δ 170.37 ($C_q$, 1 C, $C_{11}$), 165.05 ($C_q$, 2 C, $C_7$), 159.23 ($C_q$, 2 C, $C_2$), 137.50 (CH, 2 C, $C_4$), 126.28 (CH, 2 C, $C_5$), 123.68 (CH, 2 C, $C_3$), 62.81 ($CH_2$, 1 C, $C_{10}$), 61.84 ($CH_2$, 2 C, $C_8$), 61.15 ($CH_2$, 2 C, $C_1$), 56.09 ($CH_2$, 2 C, $C_{TACN}$), 55.77 ($CH_2$, 2 C, $C_{TACN}$), 54.52 ($CH_2$, 2 C, $C_{TACN}$), 25.89 ($CH_3$, 1 C, $C_{13}$), 14.22 ($CH_3$, 1 C, $C_9$).

**HR-ESI-MS:** $[M+H]^+$ = calc. 527.2976, found: 527.2974.

**HPLC:** $R_f$ = 7.46 min (Method C)

**Synthesis of 3**

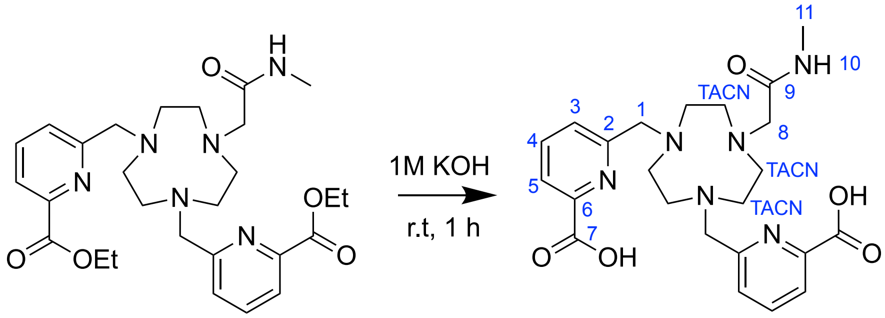


2 3

**2** (40.5 mg, 79.9 µmol, 1 eq) was dissolved in 2.4 mL of 8:2 THF:EtOH before adding 10 equivalents of 1M KOH (770 µL). The solution was stirred for one hour and was purified with a preparative HPLC (method A) to give compound **3** as a brown oil (36.2 mg, 77 µmol, 100%).

$^{1}$H NMR (400 MHz, $CD_3OD$_SPE) δ 8.04 (d, $J$ = 6.7 Hz, 2H, $H_5$), 7.93 (t, $J$ = 7.7 Hz, 2H, $H_4$), 7.62 (d, $J$ = 6.6 Hz, 2H, $H_3$), 4.26 (s, 4H, $H_1$), 3.45 (s, 1H, $H_8$), 3.18 (s, 6H, $H_{TACN}$), 2.94 (s, 6H, $H_{TACN}$), 2.61 (s, 3H, $H_{10}$).

$^{13}$C NMR (126 MHz, MeOD) δ 171.40 ($C_q$, 1 C, $C_7$), 167.82 ($C_q$, 1 C, $C_9$), 138.24 (CH, 2 C, $C_4$), 126.60 (CH, 2 C, $C_3$), 123.85 (CH, 2 C, $C_5$), 118.02 ($C_q$, 2 C, $C_2$), 115.68 ($C_q$, 2 C, $C_6$), 59.33 ($CH_2$, 2 C, $C_8$), 58.17 ($CH_2$, 2 C, $C_1$), 51.71 ($CH_2$, 2 C, $C_{TACN}$), 50.00 ($CH_2$, 2 C, $C_{TACN}$), 49.84 ($CH_2$, 2 C, $C_{TACN}$), 24.59, ($CH_3$, 1 C, $C_{10}$).

**HR-ESI-MS:** $[M+H]^+$ = calc. 469.2205, found: 469.2206.

**HPLC:** $R_f$ = 10.01 min (Method F)

Molar Extinction Coefficient: (20 mM pH 7.4 HEPES, 280 nm)

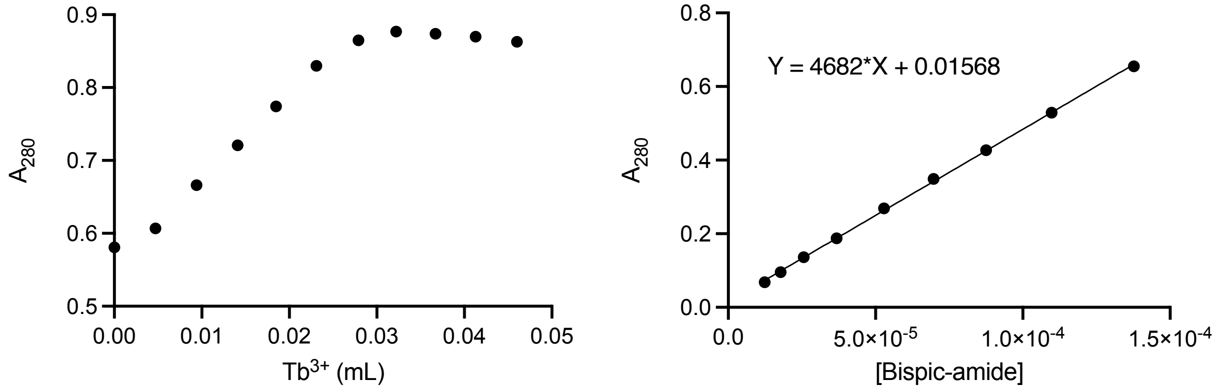


**Figure S2:** Molar Extinction Coefficient Determination of **[Tb(bispic-amide)]**$^{+}$ in 20 mM HEPES buffer.

**Synthesis of Tb(bispic-amide)**

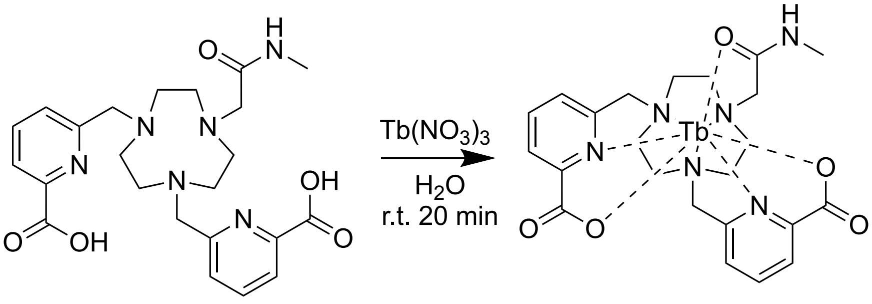


**3** **[Tb(bispic-amide)]**$^{+}$

Ligand **3** (5.3 mg, 11 μmol, 1 eq) was dissolved in 0.8 mL of water and combined with $Tb(NO_3)_3$ (5.9 mg, 17 μmol, 1.5 eq). The solution was stirred for 20 minutes and purified on a C18 cartridge to give the title compound as a white powder (2.8 mg, 11 μmol, 39%).

**[Tb(bispic-amide)]**$^{+}$
**HR-ESI-MS:** $[M+H]^{+}$= calc 627.1369, found: 627.1366

**HPLC:** $R_f$= 7.13 mins (Method F)

**Photophysical characterization**: $\lambda_{max}$= 276 nm, $\phi = 0.68 \pm 0.03$ (in pH 7.4 0.1 M Tris buffer), $\tau(H_2O) = 1.466 \pm 0.001$ ms, $\tau(D_2O) = 2.418 \pm 0.005$ ms, q= 1.0, $\tau(H_2O_{Phosphate})= 1.473 \pm 0.002$ ms $\tau(D_2O_{Phosphate})= 2.44 \pm 0.05$ ms, $q_{Phosphate}$= 1.0

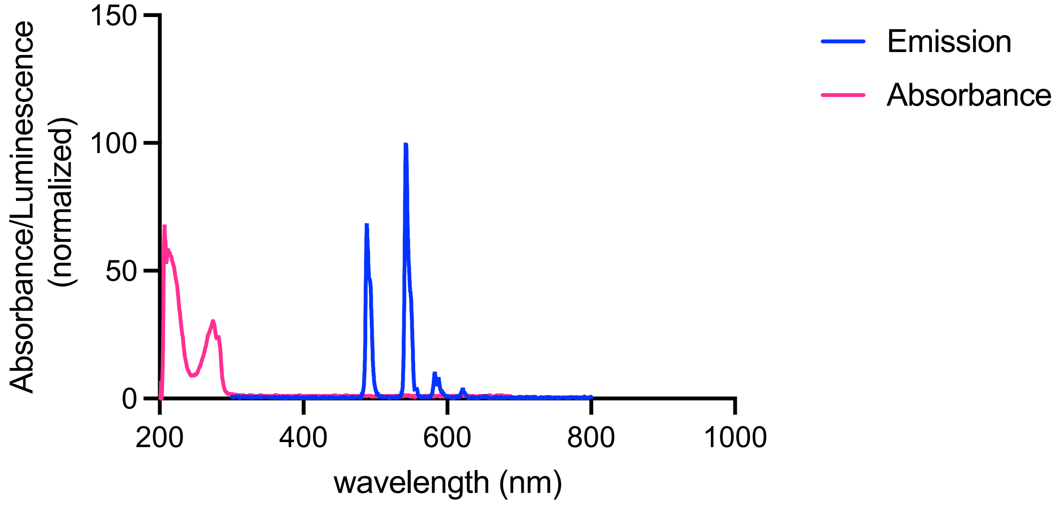


**Figure S3:** Normalized absorbance/emission spectra of [Tb(bispic-amide)]$^{+}$. Excitation at 279 nm (double excitation signal observed at 558 nm)

## 2.2 Synthesis of [Ln(bispic-lysine)]$^{+}$

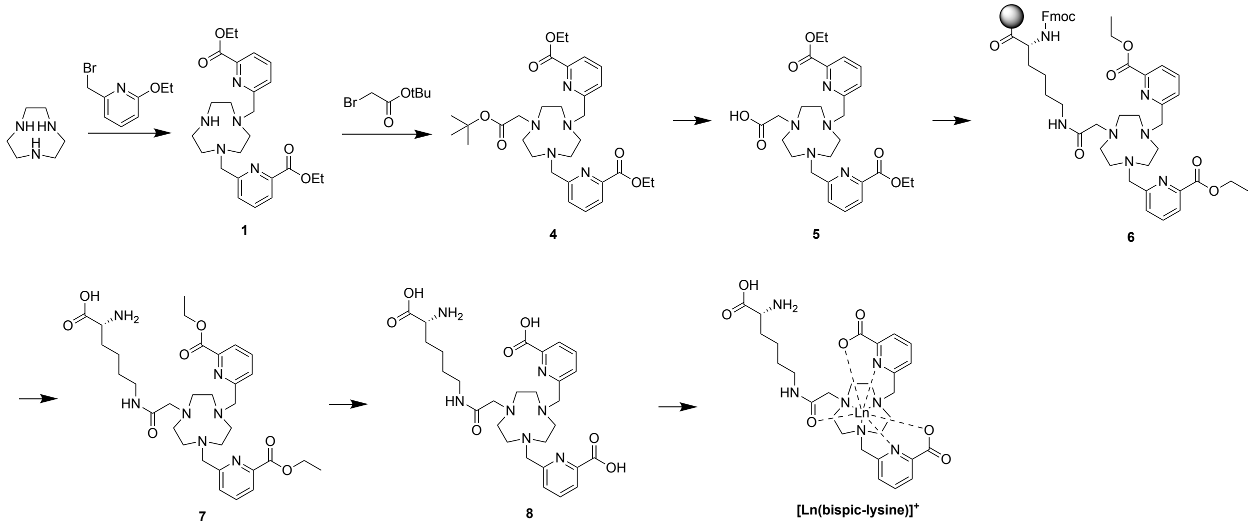


Scheme S2: Synthesis of [Ln(bispic-lysine]$^{+}$. Ln=Eu(III) or Tb(III).

## Synthesis of 4

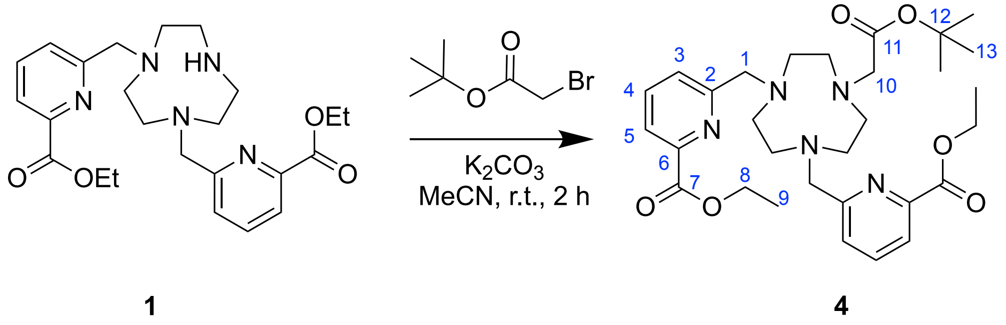

**1** (200 mg, 439 µmol, 1 eq) was dissolved in 10 mL of dry acetonitrile. Potassium carbonate (182 mg, 1.32 mmol, 3 eq) was added and the solution was stirred. The tert-butyl 2-bromoacetate (77.1 mg, 395 µmol, 0.9 eq) was added to the solution and was allowed to stir under nitrogen for one hour. The reaction was filtered and solvent was removed. The brown oil was purified with flash chromatography (method B) and fractions containing the compound were lyophilized to give a yellow-brown oil (191.5 mg, 76.6%).

**$^1$H NMR** (400 MHz, $CD_3OD$_SPE) δ 8.00 (d, $J$ = 7.8, 1.2 Hz, 2H, $H_5$), 7.89 (t, $J$ = 7.8 Hz, 2H, $H_4$), 7.67 (d, $J$ = 7.8 Hz, 1H, $H_3$), 4.35 (m, $J$ = 7.8 Hz, 2H, $H_8$), 4.31 (s, 2H, $H_1$), 3.36 (s, 2H, $H_{10}$), 3.26 (m, $J$ = 3.3, 1.6 Hz, 6 H, $H_{TACN}$), 3.11 (s, 4H, $H_{TACN}$), 2.99 (s, 2H, $H_{TACN}$), 1.39 (s, 9H, $H_{13}$), 1.32 (t, $J$ = 7.1, 6H, $H_9$).

**$^{13}$C NMR** (101 MHz, $CD_3OD$_SPE) δ 170.94 ($C_q$, 2C, $C_{11}$), 165.18 ($C_q$, 2C, $C_7$), 155.84 ($C_q$, 2C, $C_6$), 147.65 ($C_q$, 2C, $C_2$), 138.36 (CH, 2C, $C_4$), 127.21 (CH, 2C, $C_3$), 124.23 (CH, 2C, $C_5$), 81.40 (Cq, 1C, $C_{12}$), 61.63 ($CH_2$, 2C, $C_8$), 59.45 ($CH_2$, 2C, $C_1$), 55.99 ($CH_2$, 1C, $C_{10}$), 51.93 ($CH_2$, 2C, $C_{TACN}$), 49.41 ($CH_2$, 2C, $C_{TACN}$), 47.60 ($CH_2$, 2C, $C_{TACN}$), 47.39, 47.17, 46.96 , 27.00 ($CH_3$, 3C, $C_{13}$), 13.19 ($CH_3$, 2C, $C_9$).

**HR-ESI-MS:** $[M+H]^+$= calc. 570.3286, found: 570.3289

**HPLC:** $R_f$ = 7.30 min with method C

**Synthesis of 5**

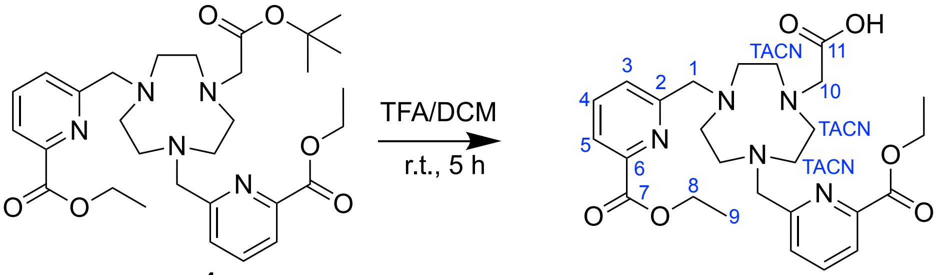


**4** **5**

Compound **4** was dissolved in 3 mL of 50:50 TFA:DCM and stirred for five hours. The product was then purified with flash chromatography (method B) and fractions containing compound **5** were lyophilized to give a yellow oil (132.2 mg, 257.3 µmol, 100%)

$^1$H NMR (500 MHz, $CD_3OD$_SPE) δ 7.97 (d, $J$ = 6.6 Hz, 2 H, $H_5$), 7.91 (t, $J$ = 7.7 Hz, 2 H, $H_4$), 7.66 (d, $J$ = 7.8 Hz, 2 H, $H_3$), 4.43, (s, 4 H, $H_1$), 4.32 (q, $J$ = 7.1 Hz, 4 H, $H_8$), 3.67 (s, 2 H, $H_{10}$), 3.45 (s, 4 H, $H_{TACN}$), 3.27 (m, 8 H, $H_{TACN}$), 1.32 (t, $J$ = 7.1 Hz, 6 H, $H_9$).

$^{13}$C NMR (126 MHz, $CD_3OD$_SPE) δ 171.66 ($C_q$, 1 C, $C_{11}$), 164.65 ($C_q$, 1 C, $C_7$), 155.61 ($C_q$, 1 C, $C_6$), 147.25 ($C_q$, 1 C, $C_2$), 138.48 (CH, 2 C, $C_4$), 127.03 (CH, 2 C, $C_3$), 124.22 (CH, 2 C, $C_5$), 116.97, 114.67, 61.75 ($CH_2$, 2 C, $C_8$), 59.60 ($CH_2$, 2 C, $C_1$), 55.45 ($CH_2$, 1 C, $C_{10}$), 51.88 ($CH_2$, 3 C, $C_{TACN}$), 51.04 ($CH_2$, 3 C, $C_{TACN}$), 13.12 ($CH_2$, 2 C, $C_9$).

**HR-ESI-MS:** $[M+H]^+$= calc. 514.2660, found: 514.2655

**HPLC:** $R_f$ = 15.25 min with method D

**Synthesis of 6**

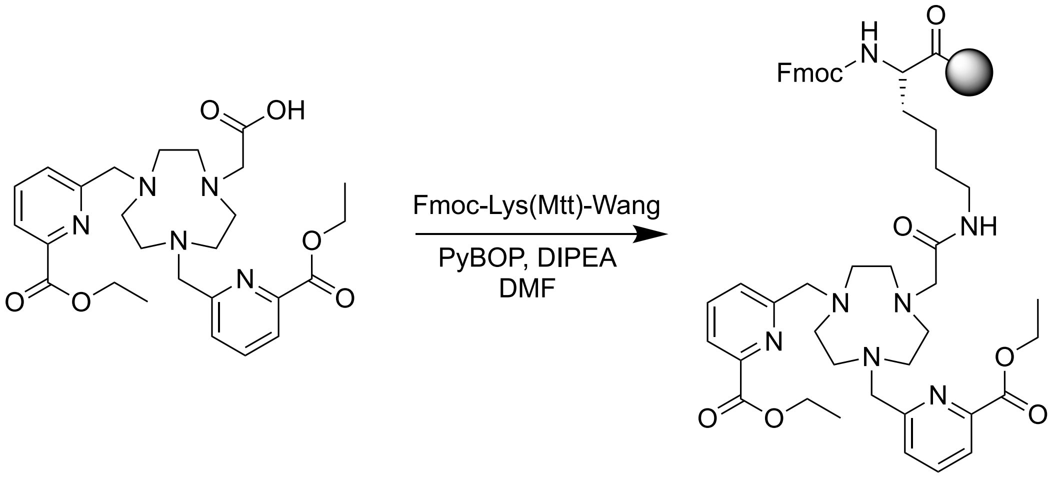


**5** **6**

The Fmoc-Lys(Mtt)-Wang resin (262 mg, 0.62 mmol/g, 162 µmol) was swelled in DMF and the Mtt group removed with 1% TFA in DCM. Compound **5** (125 mg, 243 µmol, 1.5 eq) was dissolved in DMF and stirred with DIPEA (629 mg, 4.87 mmol, 30 eq) for five minutes before adding PyBOP (422 mg, 811 µmol, 5 eq). The solution was then added to the resin and allowed to shake overnight.

**Synthesis of 7**

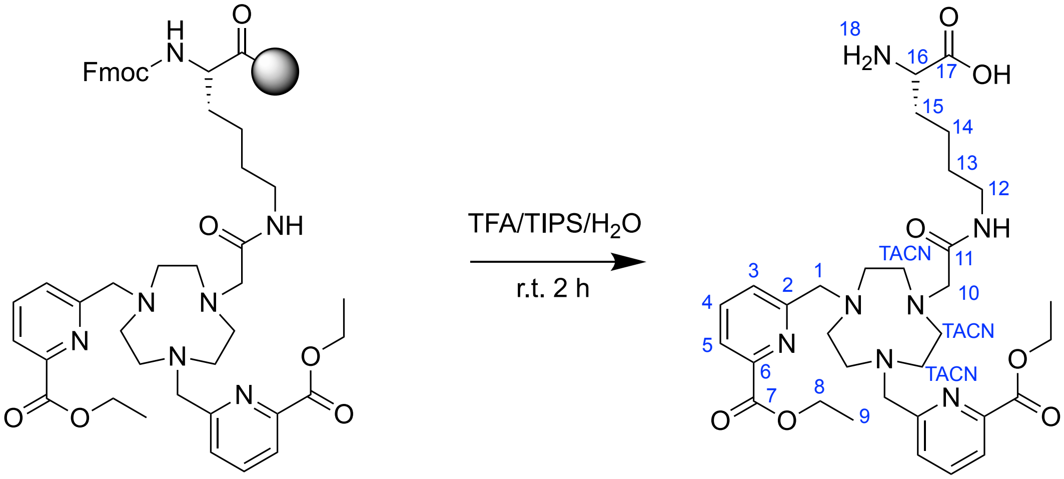


**6** **7**

The DMF was eluted from compound **6** and the Fmoc group was removed with 20% piperidine in DMF. Compound **7** was cleaved from the resin with 95:2.5:2.5 TFA:TIPS:water for 2 hours. The TFA was removed and compound **7** was purified on a preparative HPLC (method A) to give a clear oil (43.8 mg, 68.2 µmol, 43%).

**$^1$H NMR** (500 MHz, $CD_3CN$_SPE) δ 8.34 (s, 2H, $H_{18}$), 7.92 (dd, $J$ = 7.7, 1.0 Hz, 2H, $H_5$), 7.83 (t, $J$ = 7.8 Hz, 2H, $H_4$), 7.66 (d, $J$ = 7.8 Hz, 2H, $H_3$), 4.30 (q, $J$ = 7.1 Hz, 4H, $H_8$), 4.10 (s, 4H,

$H_1$), 3.60 – 3.52 (m, 2H, $H_{TACN}$), 3.09 (m, 2H, $H_{12}$), 3.04 (s, 8H, $H_{TACN}$), 2.94 (s, 2H, $H_{10}$), 1.76 (s, 2H, $H_{13}$), 1.39 (m, $J$ = 7.2 Hz, 4H, $H_{14}$ $H_{15}$), 1.30 (t, $J$ = 7.1 Hz, 6H, $H_9$).

**$^{13}$C NMR** (126 MHz, CD3CN_SPE) δ 173.57 ($C_q$, 1C, $C_{11}$), 168.25 ($C_q$, 1C, $C_{17}$), 165.06 ($C_q$, 1C, $C_{16}$) 164.54 ($C_q$, 2C, $C_7$), 156.96 ($C_q$, 2C, $C_2$), 147.42 ($C_q$, 2C, $C_6$), 137.79 (CH, 2C, $C_4$), 126.81 (CH, 2C, $C_3$), 123.65 (CH, 2C, $C_5$), 61.26 ($CH_2$, 2C, $C_8$), 60.11 ($CH_2$, 2C, $C_1$), 57.65 ($CH_2$, 2C, $C_{TACN}$), 54.08 ($CH_2$, 2C, $C_{TACN}$), 51.48 ($CH_2$, 1C, $C_{10}$), 50.87 ($CH_2$, 1C, $C_{TACN}$), 38.19 ($CH_2$, 2C, $C_{12}$), 30.27 ($CH_2$, 1C, $C_{13}$), 28.15 ($CH_2$, 1C, $C_{15}$), 22.09 ($CH_2$, 1C, $C_{14}$), 13.32 ($CH_3$, 2C, $C_9$).

**HR-ESI-MS:** $[M+H]^+$= calc. 642.3610, found: 642.3602

**HPLC:** $R_f$ = 7.89 min with method D

**Synthesis of 8**

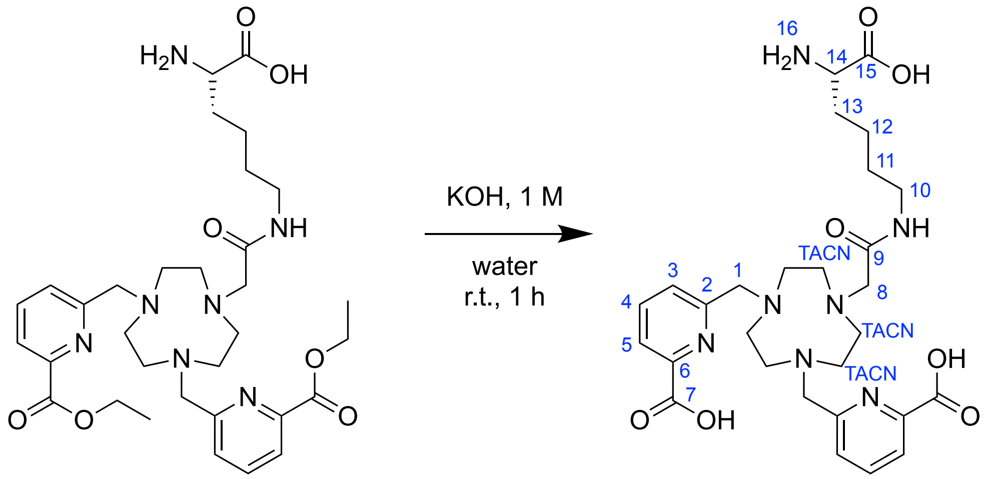


**7** **8**

Compound **7** (43.8 mg, 68.2 µmol, 1 eq) was dissolved in 2 mL of water. The 1M KOH (682 µL, 10 eq) was added to the solution and allowed to stir for 1.5 hours. The solution was then dried on a lyophilizer and purified on a preparative HPLC to give a white powder (method B) (27 mg, 68.2 µmol, 68%).

**$^1$H NMR** (500 MHz, $D_2O$) δ 7.94 (t, $J$ = 7.8 Hz, 2H, $H_4$), 7.77 (dd, $J$ = 7.8, 1.1 Hz, 2H, $H_5$), 7.52 (dd, $J$ = 7.9, 1.1 Hz, 2H, $H_3$), 4.44 (s, 4H, $H_1$), 3.77 (s, 2H, $H_8$), 3.66 (t, $J$ = 6.6, 1H, $H_{14}$), 3.54 (s, 2H, $H_{TACN}$), 3.44 (m, $J$ = 5.7 Hz, 4H, $H_{TACN}$), 3.36 (m, $J$ = 5.7 Hz, 4H, $H_{TACN}$), 2.97 (t, $J$ = 5.0 Hz, 2H, $H_{10}$), 1.75 (m, 2H, $H_{12}$), 1.33 (m, 2H, $H_{11}$), 1.24 (m, 2H, $H_{13}$).

**$^{13}$C NMR** (126 MHz, $D_2O$) δ 174.69 ($C_q$, 1C, $C_{15}$), 169.65 ($C_q$, 1C, $C_9$), 168.44 ($C_q$, 1C, $C_7$), 152.73 ($C_q$, 2C, $C_2$), 150.82 ($C_q$, 2C, $C_6$), 140.59 (CH, 2C, $C_4$), 126.15 (CH, 2C, $C_3$), 124.00 (CH, 2C, $C_5$), 58.80 ($CH_2$, 2C, $C_1$), 57.61 ($CH_2$, 1C, $C_8$), 54.57 (CH, 1C, $C_{14}$), 50.61 ($CH_2$, 2C, $C_{TACN}$), 50.55 ($CH_2$, 2C, $C_{TACN}$), 50.51 ($CH_2$, 2C, $C_{TACN}$), 38.77 ($CH_2$, 1C, $C_{10}$), 29.99 ($CH_2$, 1C, $C_{12}$), 27.83 ($CH_2$, 1C, $C_{11}$), 21.65 ($CH_2$, 1C, $C_{13}$).

**HR-ESI-MS:** $[M-H]^-$= calc. 584.2838, found: 584.2844

**HPLC:** $R_f$= 7.13 mins (method F)

**Synthesis of [Ln(bispic-lysine)]$^+$**

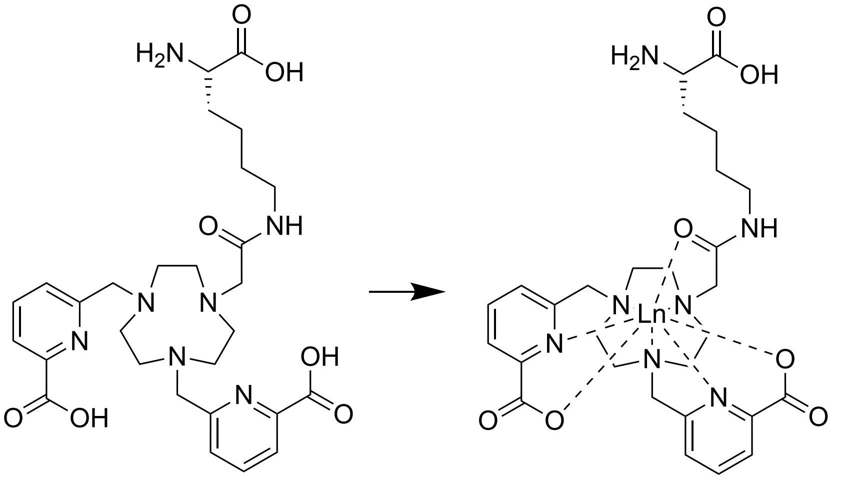


**8** **[Ln(bispic-lysine)]$^+$**

General procedure. Comopound **9** (13 mg, 22 μmol, 1 eq) was dissolved in 1 mL of water and $LnCl_3$ in 0.5 mL of water (1 eq). Solutions were stirred together for 20 minutes and purify through a C18 cartridge to give the title compounds ([Tb(bispic-lysine)]$^+$: 4 mg, 20%; [Eu(bispic-lysine)]$^+$: 5.5 mg, 34%).

**[Tb(bispic-lysine)]$^+$**

**HR-ESI-MS:** $[M+H]^+$= calc. 742.2002, found: 742.1997

**HPLC:** $R_f$= 7.10 min (method F)

**Photophysical characterization**: $\lambda_{max}$= 274 nm, $\phi$ = 0.50 ± 0.01 (in pH 7.4 0.1 M Tris buffer), $\tau(H_2O)$ = 1.477 ± 0.004 ms, $\tau(D_2O)$ = 2.395 ± 0.005 ms, q= 1.0, $\tau(H_2O_{Phosphate})$= 1.4783 ± 0.0005 ms, $\tau(D_2O_{Phosphate})$= 2.387 ± 0.009 ms, $q_{Phosphate}$= 1.0, $\varepsilon_{274\ nm}$ = 9323 $M^{-1}cm^{-1}$, $\phi_{phosphate}$= 0.53 ± 0.02

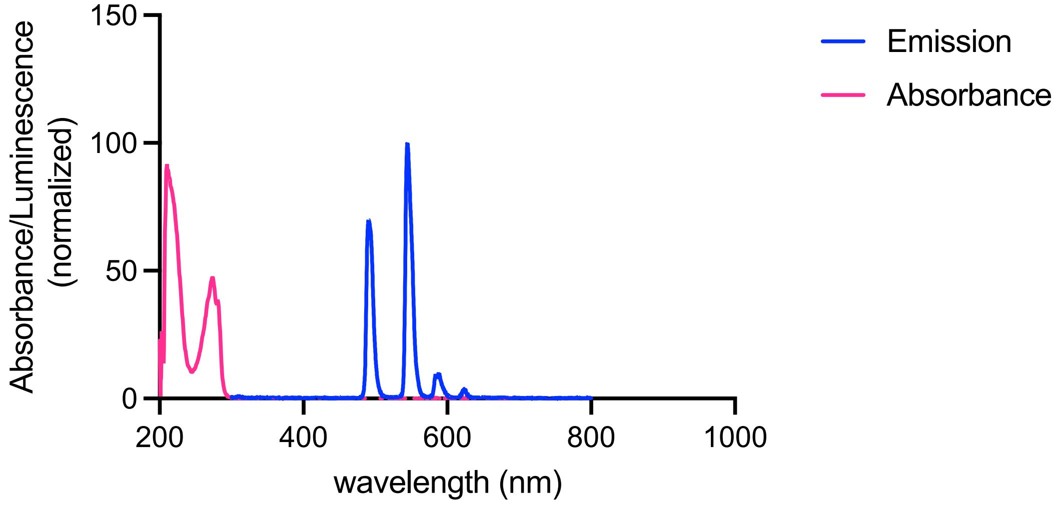

**Figure S4:** Normalized absorbance/emission spectra of **[Tb(bispic-lysine)]**$^+$. Excitation at 279 nm (double excitation signal observed at 558 nm)

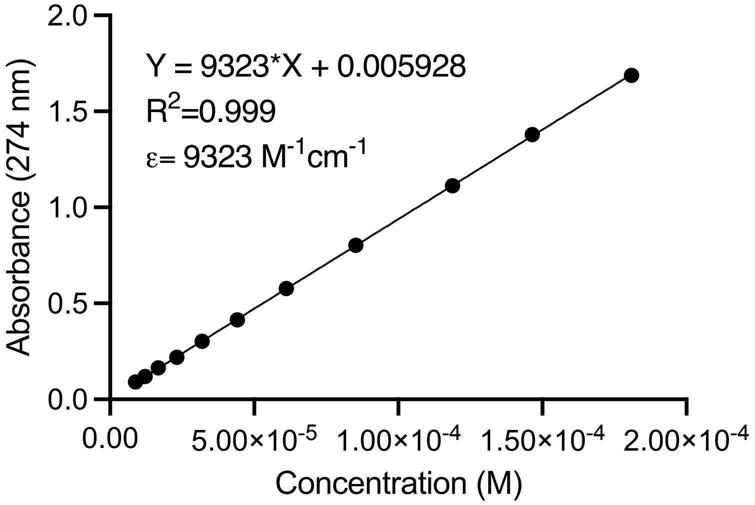


**Figure S5:** Molar Extinction Coefficient Determination of **[Tb(bispic-lysine)]**$^+$ in 20 mM HEPES buffer.

**[Eu(bispic-lysine)]**$^+$

**HR-ESI-MS:** $[M]^+$= calc. 736.1961, found: 736.1957

**HPLC:** $R_f$= 7.17 min (method F)

**Photophysical characterization**: $\lambda_{max}$= 274 nm, $\phi$ = 0.0293 ± 0.0007 (in pH 7.4 0.1 M Tris buffer), $\tau(H_2O)$ = 0.526 ± 0.003 ms, $\tau(D_2O)$ = 1.531 ± 0.005, q= 1.2, $\tau(H_2O_{Phosphate})$= 0.527 ± 0.002 ms, $\varepsilon_{274\ nm}$ = 9335 $M^{-1}cm^{-1}$, $\phi_{phosphate}$ = 0.0358 ± 0.0007 (in pH 7.4 0.1 M Tris buffer)

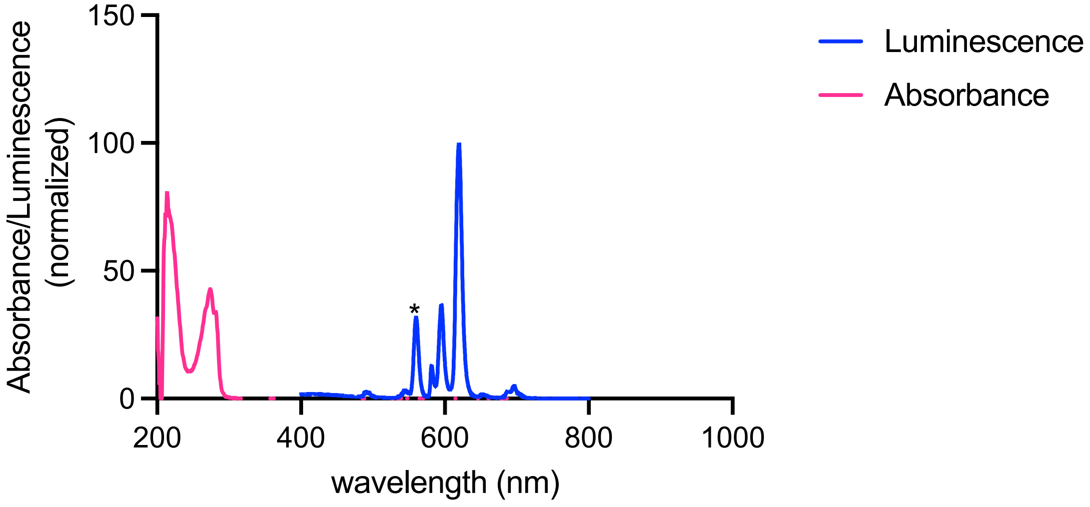


**Figure S6:** Normalized absorbance/emission spectra of **[Eu(bispic-lysine)]**$^+$. Excitation at 279 nm (*double excitation signal observed at 558 nm)

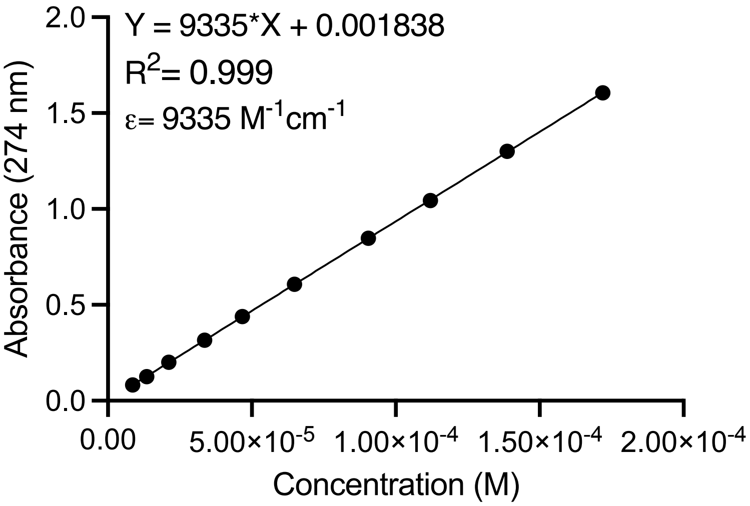


**Figure S7:** Molar Extinction Coefficient Determination of **[Eu(bispic-lysine)]**$^+$ in 20 mM HEPES buffer.

### 2.3 Synthesis of [Ln(trispic)]

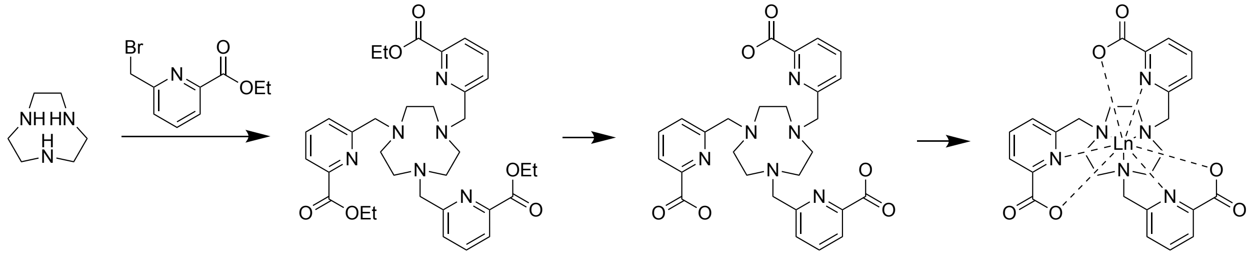


Scheme S3: Synthesis of [Ln(trispic)]. Ln= Tb(III) or Eu(III).

The Ln(trispic) complexes were synthesized with a literature procedure.[5]

**[Tb(trispic)]**
**Photophysical characterization**: $\lambda_{max}$= 274 nm, $\phi$ = 0.714 $\pm$ 0.007 (in pH 7.4 0.1 M Tris buffer), $\tau(H_2O)$ = 2.017 $\pm$ 0.004 ms, $\tau(D_2O)$ = 2.176 $\pm$ 0.001 ms, q= -0.1, $\tau(H_2O_{Phosphate})$= 2.027 $\pm$ 0.002 ms, $\tau(D_2O_{Phosphate})$= 2.165 $\pm$ 0.001 ms, $q_{Phosphate}$= -0.1

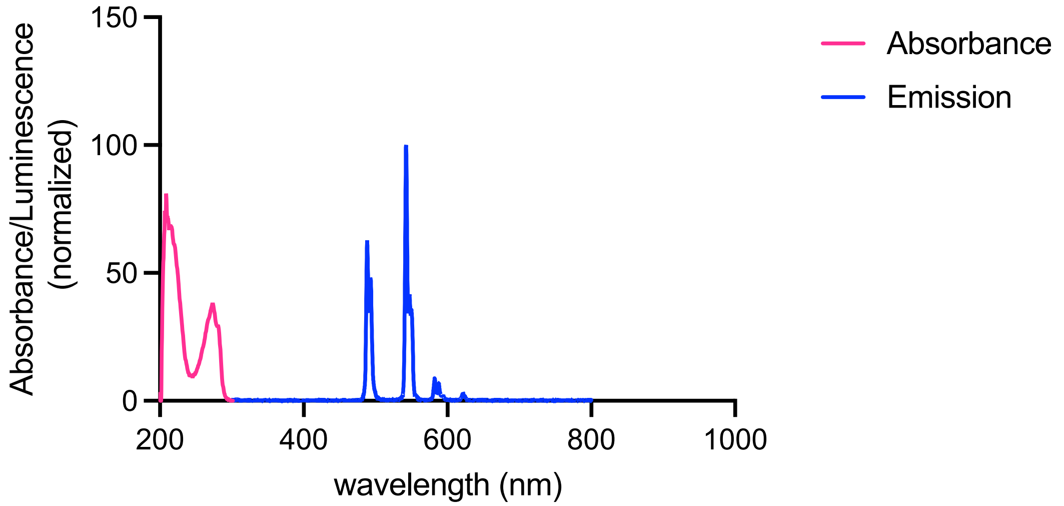


**Figure S8:** Normalized absorbance/emission spectra of [Tb(trispic)]. Excitation at 279 nm (double excitation signal observed at 558 nm)

**[Eu(trispic)]**

**Photophysical characterization**: $\lambda_{max}$= 274 nm, $\phi$ = 0.09 ± 0.01 (in pH 7.4 0.1 M Tris buffer), $\tau(H_2O)$ = 1.083 ± 0.008 ms, $\tau(D_2O)$ = 1.517 ± 0.006 ms, q= 0.0, $\tau(H_2O_{Phosphate})$= 1.077 ± 0.004 ms, $\tau(D_2O_{Phosphate})$= 1.509 ± 0.008 ms, $q_{Phosphate}$= 0.0

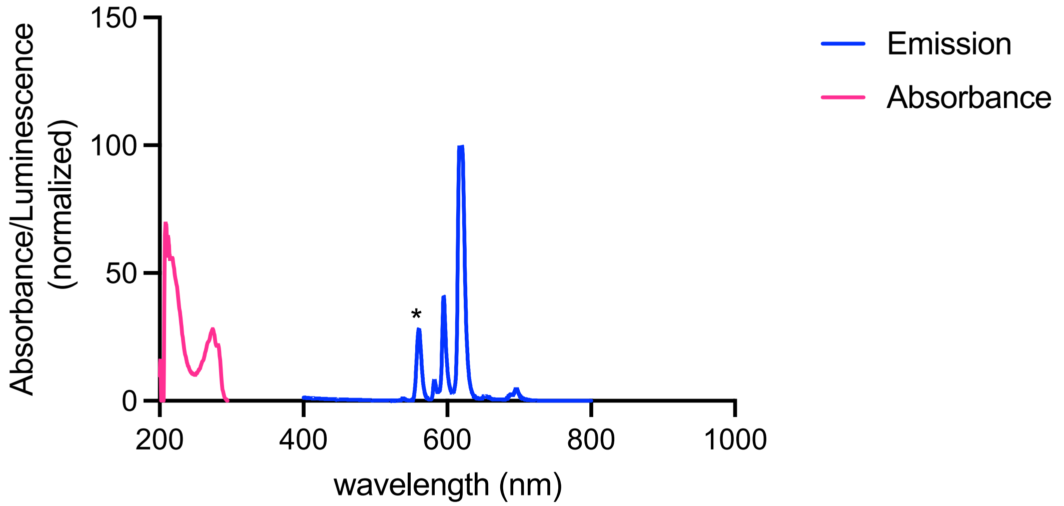


**Figure S9:** Normalized absorbance/emission spectra of [Eu(trispic)]. Excitation at 279 nm (*double excitation signal observed at 558 nm)

## 2.4 Synthesis of [Ln(bispic-acetate)]

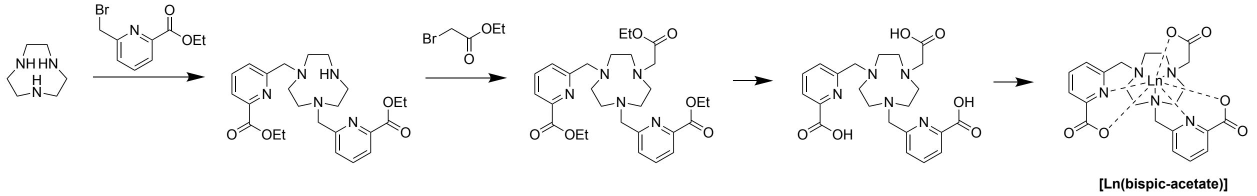

Scheme S4: Synthesis of [Ln(bispic-acetate)]. Ln= Tb(III) or Eu(III).

The Ln(bispic-acetate) complexes were synthesized with a literature procedure.[6]

**[Tb(bispic-acetate)]**

**Photophysical characterization**: $\lambda_{max}$= 274 nm, $\phi$ = 0.52 $\pm$ 0.05 (in pH 7.4 0.1 M Tris buffer), $\tau(H_2O)$ = 1.523 $\pm$ 0.002 ms, $\tau(D_2O)$ = 2.425 $\pm$ 0.007 ms, q= 0.9, $\tau(H_2O_{Phosphate})$= 1.528 $\pm$ 0.001 ms, $\tau(D_2O_{Phosphate})$= 2.414 $\pm$ 0.007 ms, $q_{Phosphate}$= 0.9

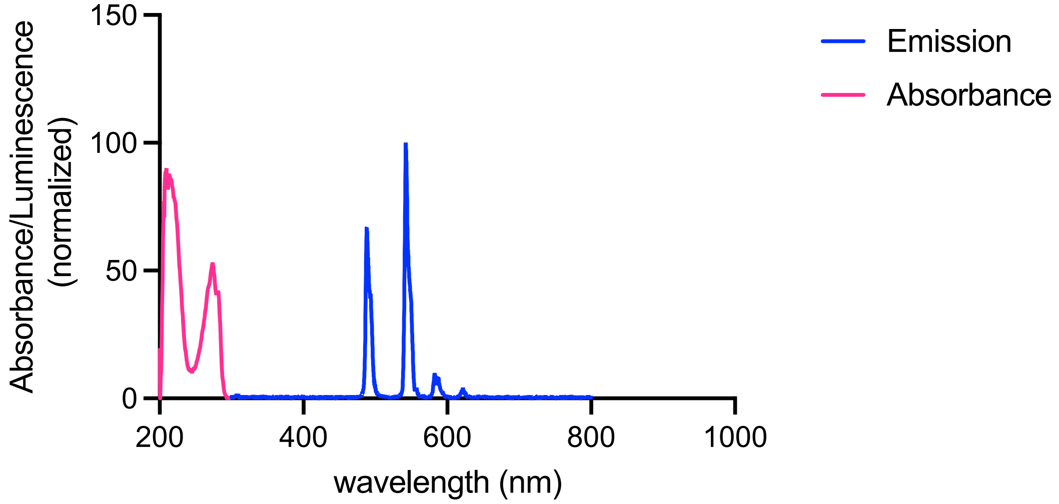


**Figure S10:** Normalized absorbance/emission spectra of [Tb(bispic-acetate)]. Excitation at 279 nm (double excitation signal observed at 558 nm)

**[Eu(bispic-acetate)]**

**Photophysical characterization**: $\lambda_{max}$= 274 nm, $\phi$ = 0.041 $\pm$ 0.001 (in pH 7.4 0.1 M Tris buffer), $\tau(H_2O)$ = 0.536 ± 0.003 ms, $\tau(D_2O)$ = 1.558 $\pm$ 0.001 ms, q= 1.2, $\tau(H_2O_{Phosphate})$= 0.535 ± 0.001 ms, $\tau(D_2O_{Phosphate})$= 1.565 ± 0.001 ms, $q_{Phosphate}$= 1.2

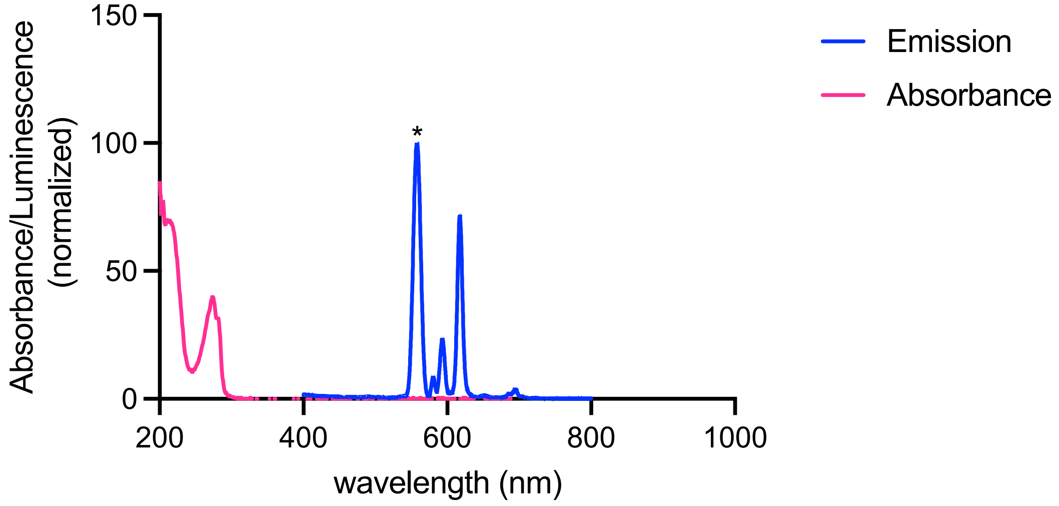

**Figure S11:** Normalized absorbance/emission spectra of [Eu(bispic-acetate)]. Excitation at 279 nm (*double excitation signal observed at 558 nm)

**2.5 Synthesis of [Ln(bispic)]$^+$**

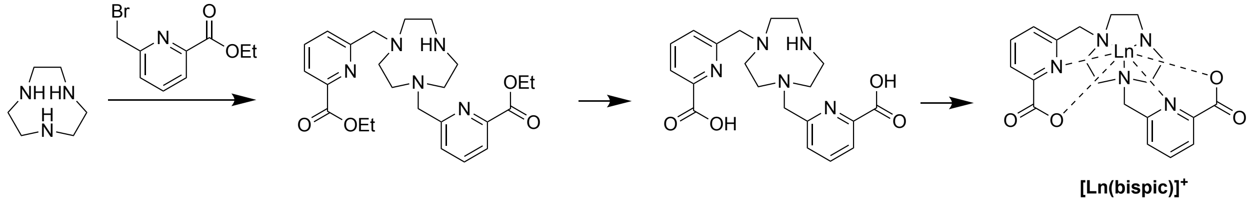


Scheme S5: Synthesis of [Ln(bispic)]$^+$. Ln= Tb(III) or Eu(III).

The [Ln(bispic)]$^+$ complexes were synthesized with a literature procedure.[7]

[Tb(bispic)]$^+$

**Photophysical characterization**: $\lambda_{max}$= 274 nm , $\phi$ = 0.30 $\pm$ 0.02 (in pH 7.4 0.1 M Tris buffer), $\tau(H_2O)$ = 1.137 $\pm$ 0.002 ms, $\tau(D_2O)$ = 2.406 $\pm$ 0.004 ms, q= 2.0, $\tau(H_2O_{Phosphate})$= 1.143 $\pm$ 0.001 ms, $\tau(D_2O_{Phosphate})$= 2.23 $\pm$ 0.02 ms, $q_{Phosphate}$= 1.8

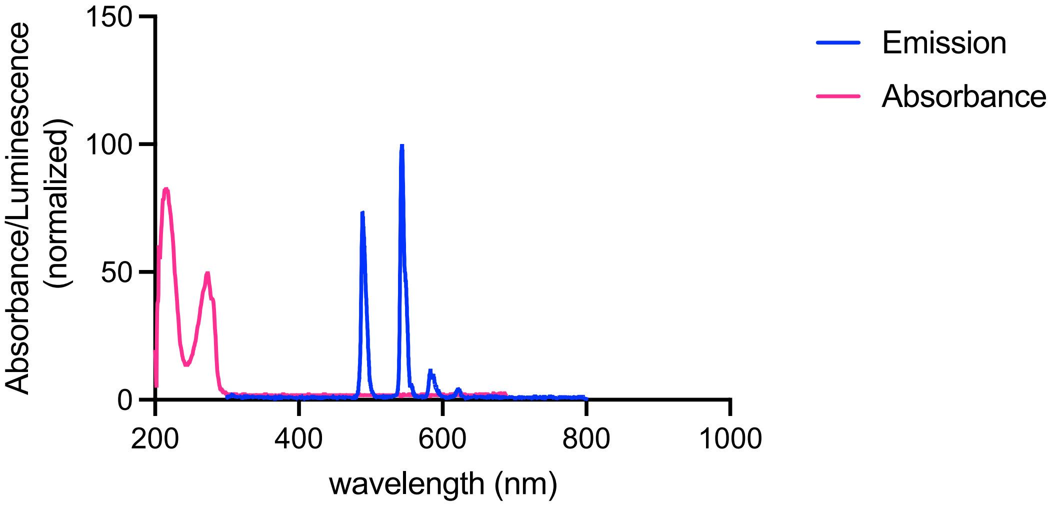


**Figure S12:** Normalized absorbance/emission spectra of [Tb(bispic)]$^+$. Excitation at 279 nm (double excitation signal observed at 558 nm)

[Eu(bispic)]$^+$

**Photophysical characterization**: $\lambda_{max}$= 274 nm, $\phi$ = 0.0151 $\pm$ 0.004 (in pH 7.4 0.1 M Tris buffer), $\tau(H_2O)$ = 0.6114 ± 0.003 ms, $\tau(D_2O)$ = 1.560 $\pm$ 0.001 ms, q= 0.9

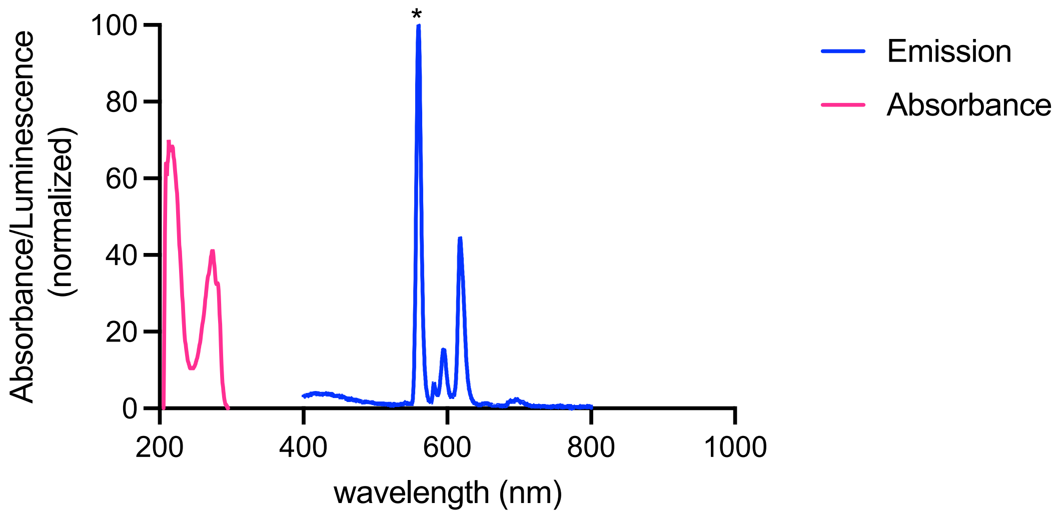


**Figure S13:** Normalized absorbance/emission spectra of $[Eu(bispic)]^{+}$. Excitation at 279 nm (*double excitation signal observed at 558 nm)

## 2.6 Synthesis of $[Ln(bispic\text{-}Ser)]^{+}$

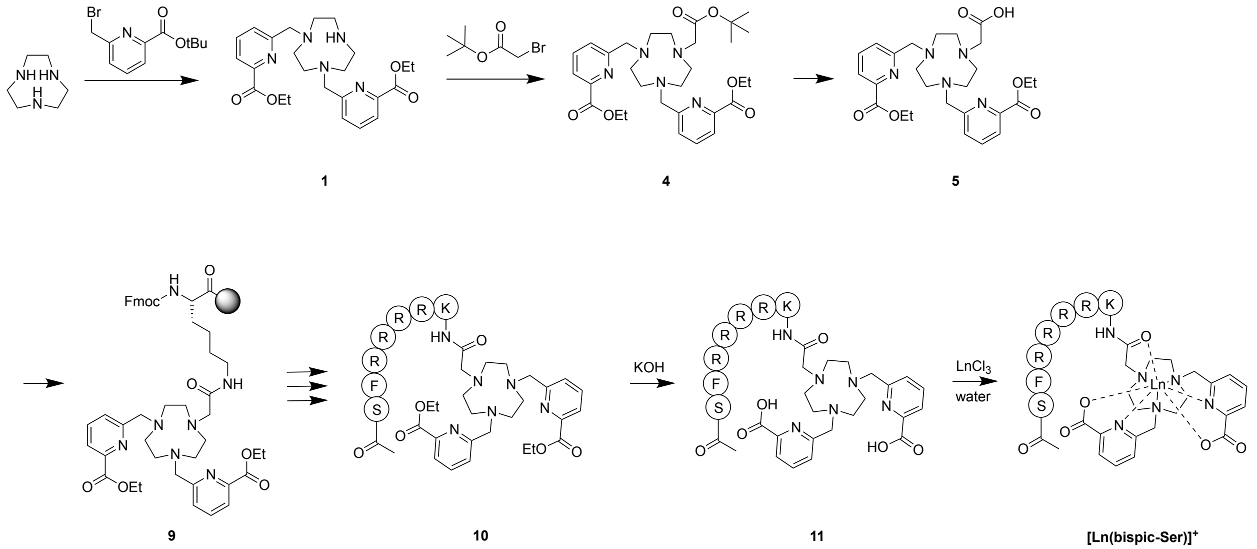


Scheme S6: Synthesis of **$[Ln(bispic\text{-}Ser)]^{+}$**

## Synthesis of 9

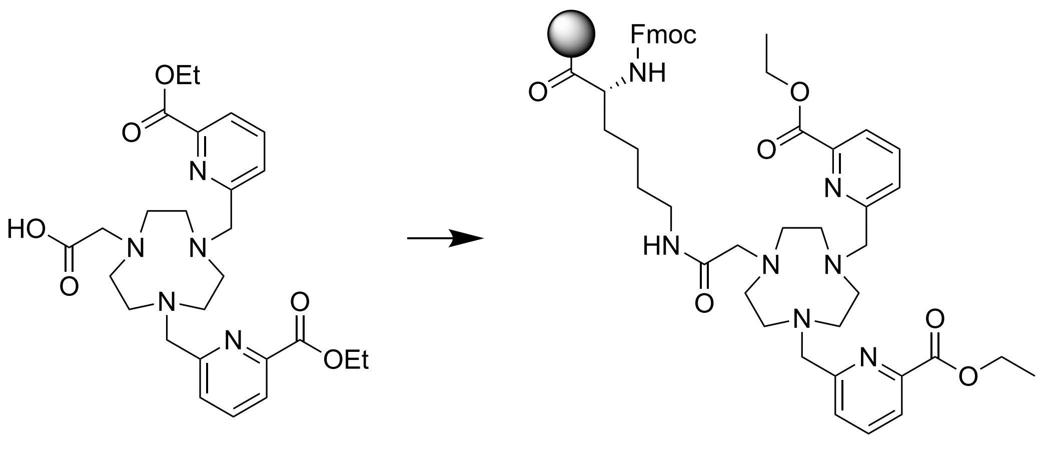


5 9

The Fmoc-Lys(Mtt)-rink (276 mg, 0.62 mmol/g, 171 μmol) was swelled with DMF and the Mtt group removed with 1% TFA in DCM. Compound **5** (132 mg, 257 μmol, 1.5 eq) was dissolved in DMF and stirred with DIPEA (664 mg, 5.13 mmol, 30 eq) for five minutes before adding PyBOP (445 mg, 856 μmol, 5 eq). The solution was then added to the resin and allowed to shake overnight.

**Synthesis of 10**

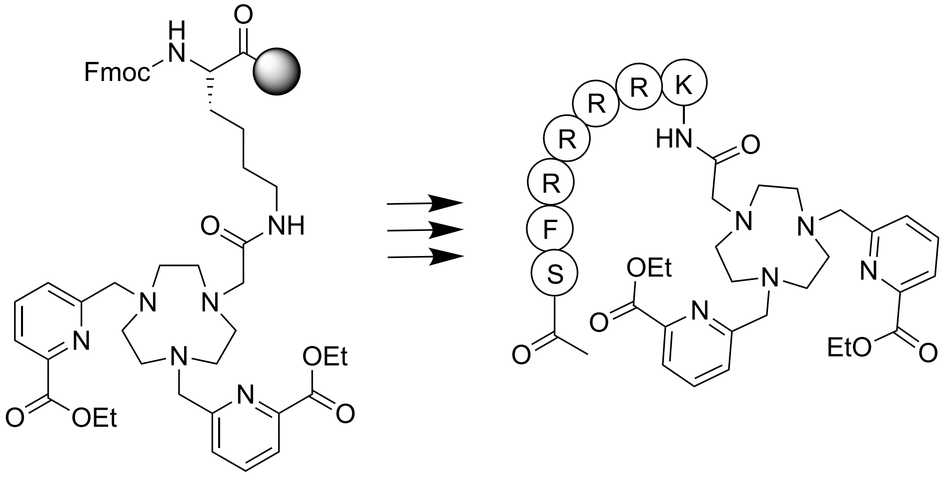


9 10

The peptide was elongated with standard SPPS procedure with Fmoc-Ser(tBu)-OH, Fmoc-Phe-OH, and Fmoc-Arg(Pbf)-OH. The Fmoc was removed with 20% piperidine in DMF and couplings were performed with 3-fold excess of the L-amino acid, 2-fold excess of PyBOP, and four-fold excess of DIPEA. The acetylation was performed with 5 mL of 20% $Ac_2O$ in DMF. Following cleavage (95:2.5:2.5 TFA:water:TIPS), the peptide was washed with cold diethyl ether and purified with a preparative HPLC (method A). Compound **10** was dried on a lyophilizer to give a white powder (13.1 mg, 5%).

**HR-ESI-MS:** $[M+3H]^{3+}$= calc. 514.6367, found: 514.6344; $[M+4H]^{3+}$= calc. 386.2286, found: 386.2278

**MALDI:** $[M+H]^{+}$= calc. 1541.9, found: 1541.8

**LR-ESI-MS:** $[M+2H]^{2+}$= calc. 771.4 found: 771.4 $[M+3H]^{3+}$= calc. 514.6, found: 514.6; $[M+4H]^{3+}$= calc. 386.2, found: 386.4

**HPLC:** $R_f$ = 7.38 mins (method F)

**Synthesis of 11**

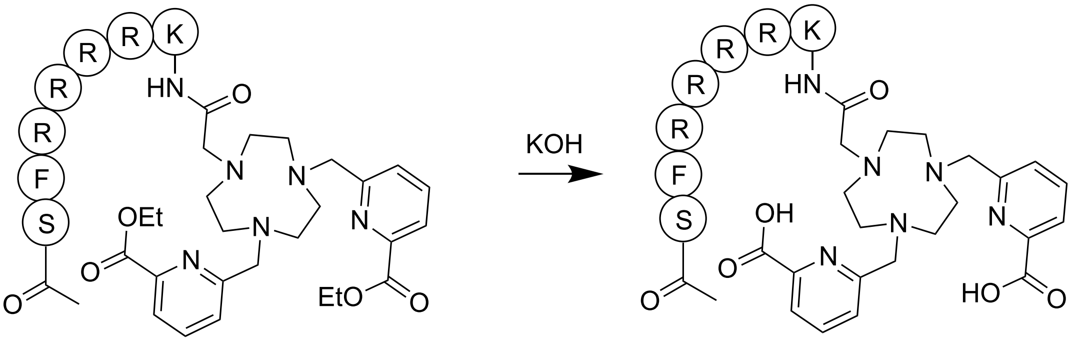


**10** **11**

The peptide **10** (5 mg, 3 µmol, 1 eq) was dissolved in 1 mL of water and stirred with 1 M KOH (30 µL, 10 eq) for one hour. The solution was then purified with an analytical HPLC (method F) and dried on a lyophilizer to give compound **11** as a white powder (5 mg, 3 µmol, 100%).

**HR-ESI-MS:** $[M+3H]^{+3}$= calc., 495.9481 found: 495.9473; $[M+4H]^{+4}$= calc., 372.2129 found: 372.2124

**HPLC:** $R_f$ = 6.97 min (Method F)

**Synthesis of [Tb(bispic-Ser)]$^+$**

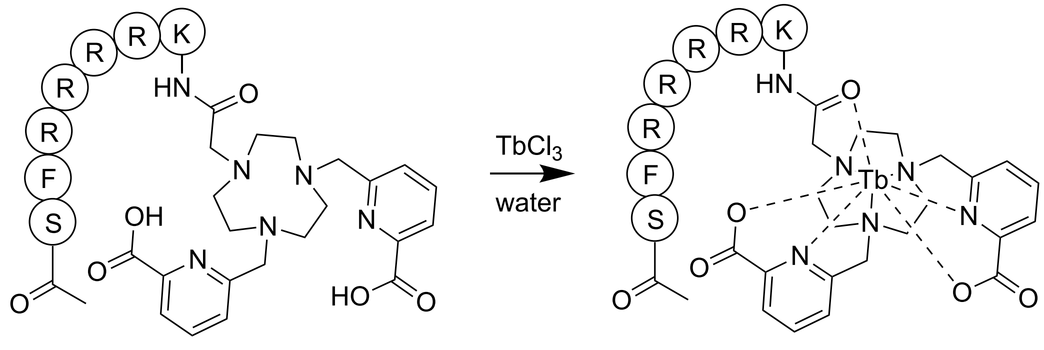


**11** **[Tb(bispic-Ser)]$^+$**

Compound **11** (1 mg, 0.7 µmol, 1 eq) was dissolved in 200 uL of water. A solution $TbCl_3$ in water (0.0090 g/mL) was made to deliver 0.3 mg of $TbCl_3$ to the stirring solution. The pH water

adjusted to 5.5 and the reaction was allowed to stir for 30 minutes before purifying on analytical HPLC (method D) (1 mg, 90%, 0.7 μmol).

**HR-ESI-MS:** $[M+2H]^{3+}$= calc. 547.9154 found 547.9180; $[M+3H]^{+4}$= calc., 411.1884 found: 411.1908; $[M+4H]^{+5}$= calc., 329.1521 found: 329.1544

**Photophysical characterization**: $\lambda_{max}$= 274 nm, $\phi$ = 0.55 ± 0.01(in pH 7.4 0.1 M Tris buffer), $\tau(H_2O)$ = 1.49 ± 0.01 ms, $\tau(D_2O)$ = 2.215 ± 0.001 ms, q= 0.8

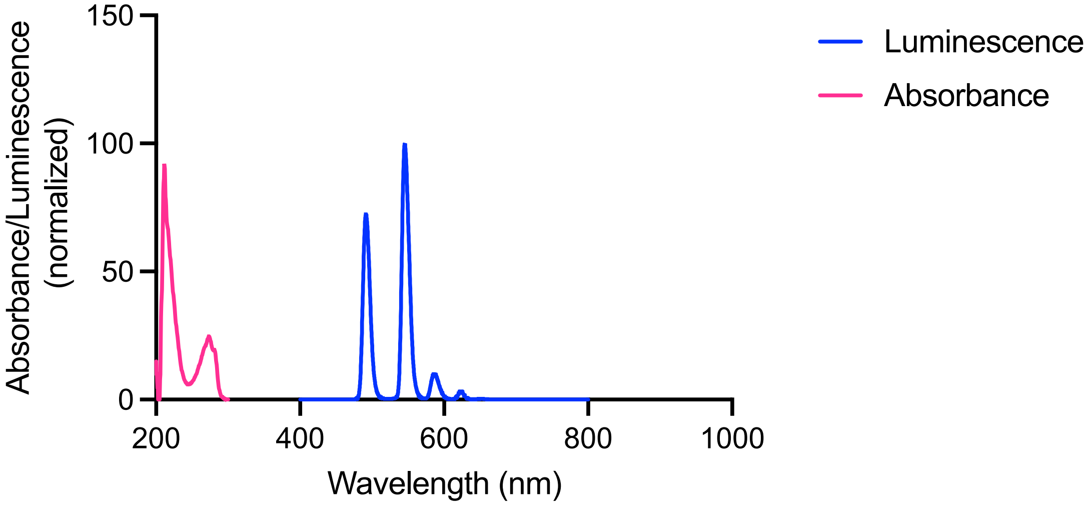


**Figure S14:** Normalized absorbance/emission spectra of $[Tb(bispic\text{-}Ser)]^{+}$. Excitation at 279 nm (double excitation signal observed at 558 nm).

**HPLC:** $R_f$= 6.93 min (method F)

**Synthesis of [Eu(bispic-Ser)]⁺**

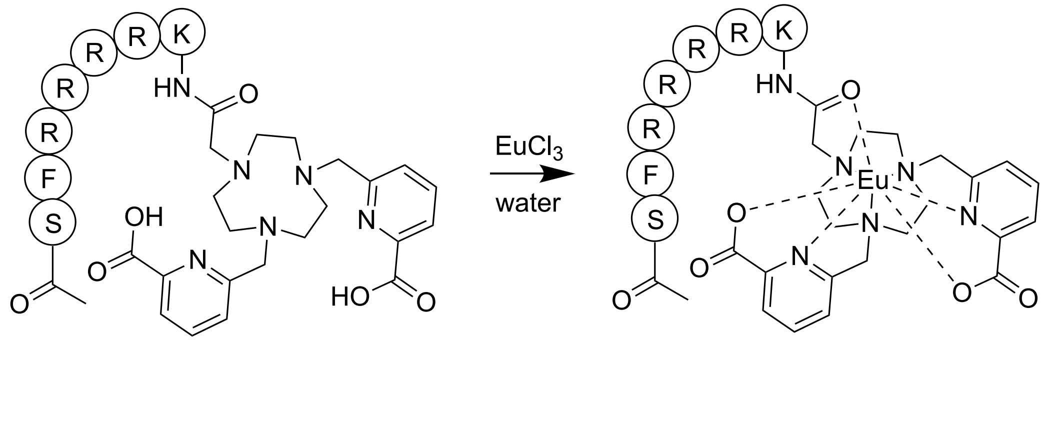


**11** **[Eu(bispic-Ser)]⁺**

Compound **11** (3 mg, 2 μmol, 1 eq) was dissolved in 200 μL of water. A solution $EuCl_3$ in water (0.0071 g/mL) was made to deliver 0.7 mg of $EuCl_3$ to the stirring solution. The pH water adjusted to 5.5 and the reaction was allowed to stir for 30 minutes before purifying on analytical HPLC (method D) (3 mg, 90%, 2 μmol).

**Photophysical characterization**: $\lambda_{max}$= 274 nm, $\phi$ = 0.032 ± 0.008 (in pH 7.4 0.1 M Tris buffer), $\tau(H_2O)$ = 0.552 ± 0.005 ms, $\tau(D_2O)$ = 1.499 ± 0.002 ms, q= 1.1

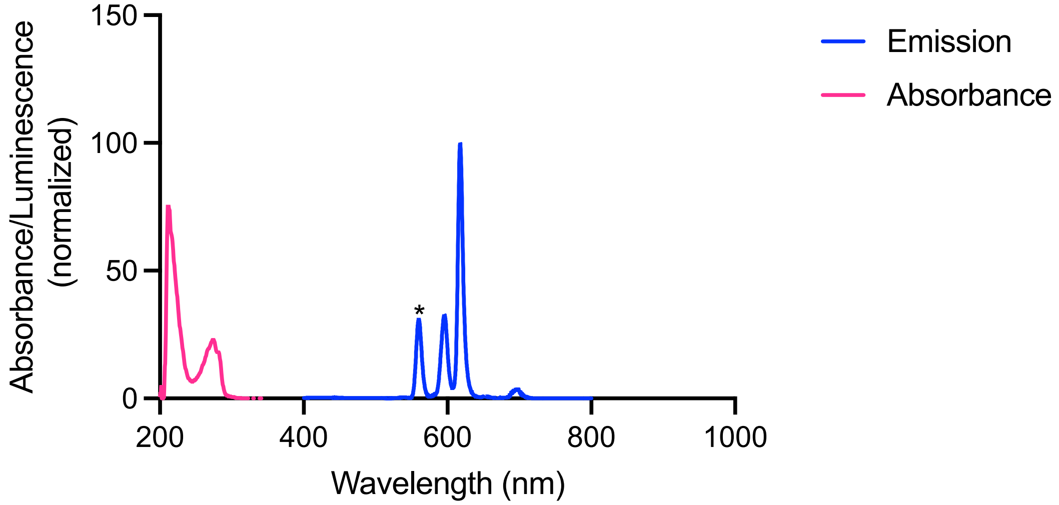


**Figure S15:** Normalized absorbance/emission spectra of [Eu(bispic-Ser)]$^+$. Excitation at 279 nm (*double excitation signal observed at 558 nm).

**HR-ESI-MS:** $[M+2H]^{3+}$= calc., 545.9140 found: 545.9139; $[M+H]^{2+}$= calc., 818.3674 found: 818.3666

**MALDI:** $[M+H]^+$= calc. 1635.7, found: 1635.3

**HPLC:** $R_f$= 6.93 min (method F)

## 2.7 Synthesis of Ln(bispic-peptides)

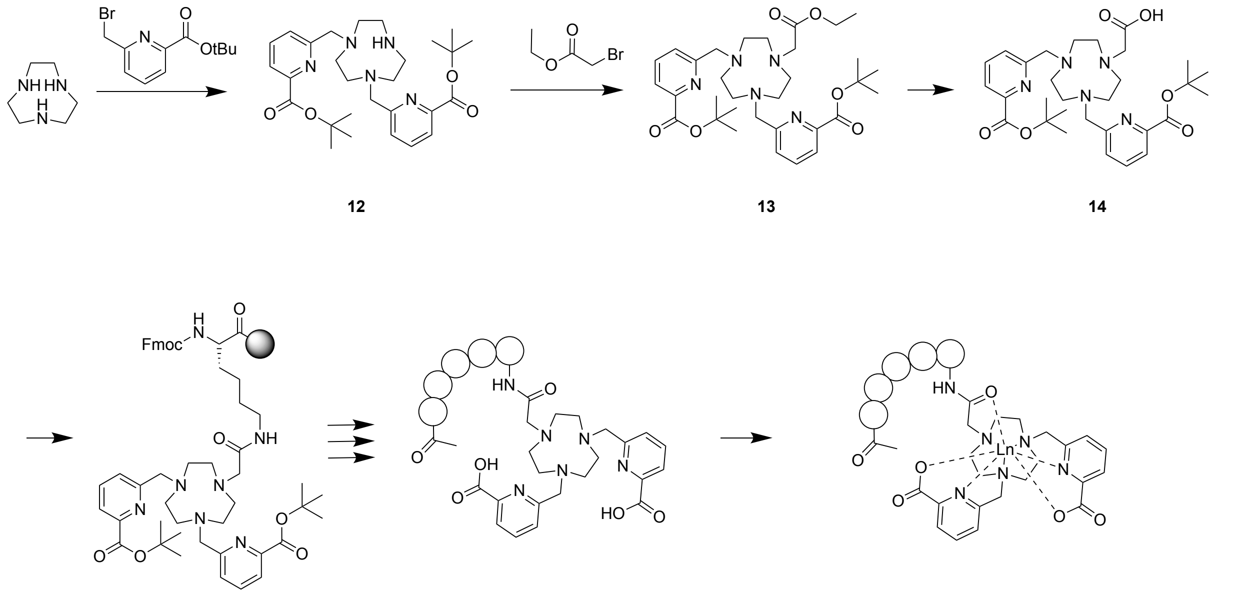


**12** **13** **14**

**15**

**Peptides**

**16:** AcHN-pSFRRRRK-$CONH_2$
**17**: AcHN-SFRRRRGGGK-$CONH_2$
**18**: AcHN-pSFRRRRGGGK-$CONH_2$
**19**: AcHN-SFRRRRPPPK-$CONH_2$
**20**: AcHN-pSFRRRRPPPK-$CONH_2$

**Peptides**

**[Ln(bispic-PSer)]$^+$:** AcHN-pSFRRRRK-$CONH_2$
**[Ln(bispic-GGG-Ser)]$^-$:** AcHN-SFRRRRGGGK-$CONH_2$
**[Ln(bispic-GGG-PSer)]$^+$:** AcHN-pSFRRRRGGGK-$CONH_2$
**[Ln(bispic-PPP-Ser)]$^-$:** AcHN-SFRRRRPPPK-$CONH_2$
**[Ln(bispic-PPP-PSer)]$^+$:** AcHN-pSFRRRRPPPK-$CONH_2$

Scheme S7. Synthesis of Ln(bispic-peptides)

**Synthesis of 12**

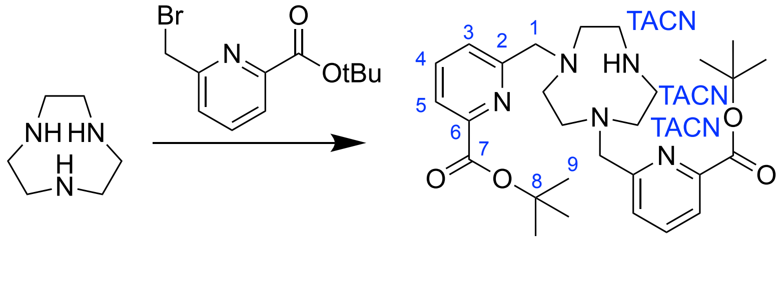


**12**

The tert-butyl 6-(bromomethyl)picolinate was synthesized according to literature procedure.[8] The triazacyclononane (tacn) (456 mg, 3.53 mmol, 2 eq) was dissolved in 10 mL of dry acetonitrile. Potassium carbonate (731 mg, 5.29 mmol, 3 eq) was added and the solution was stirred under nitrogen. The tert-butyl 6-(bromomethyl)picolinate (480 mg, 1.76 mmol, 1 eq) was dissolved in 5 mL of dry acetonitrile and added to solution dropwise. The solution was allowed to stir for one hour before it was filtered and solvent was removed. The yellow oil was purified with flash chromatography (method B) and fractions containing compound **12** were lyophilized to give a yellow oil (136 mg, 15 %).

**$^1$H NMR** (500 MHz, $CD_3CN$) δ 7.84 (d, $J$ = 7.8 Hz, 2H, $H_5$), 7.65 (t, $J$ = 7.7 Hz, 2H, $H_4$), 7.40 (d, $J$ = 8.9 Hz, 2H, $H_3$), 3.95 (s, 4H, $H_1$), 3.21 (t, $J$ = 5.7 Hz, 4H, $H_{TACN}$), 2.95 (t, $J$ = 5.7 Hz, 4H, $H_{TACN}$), 2.74 (s, 4H, $H_{TACN}$), 1.58 (s, 18H, $H_9$).

**$^{13}$C NMR** (126 MHz, $CD_3CN$) δ 164.87 ($C_q$, 2C, $C_7$), 164.17 ($C_q$, 2C, $C_6$), 159.81 ($C_q$, 2C, $C_2$), 137.59 ($C_q$, 1C, $C_{15}$), 125.76 (CH, 2C, $C_5$), 123.16 (CH, 1C, $C_3$), 82.06 ($C_q$, 2C, $C_8$), 59.65 ($CH_2$, 2C, $C_1$), 51.92 ($CH_2$, 2C, $C_{TACN}$), 48.58 ($CH_2$, 2C, $C_{TACN}$), 45.12 ($CH_2$, 2C, $C_{TACN}$), 27.25 ($CH_3$, 2C, $C_9$).

**HR-ESI-MS:** $[M+H]^+$= calc. 512.3231, found: 512.3233

**HPLC:** $R_f$= 6.90 mins (method E)

**Synthesis of 13**

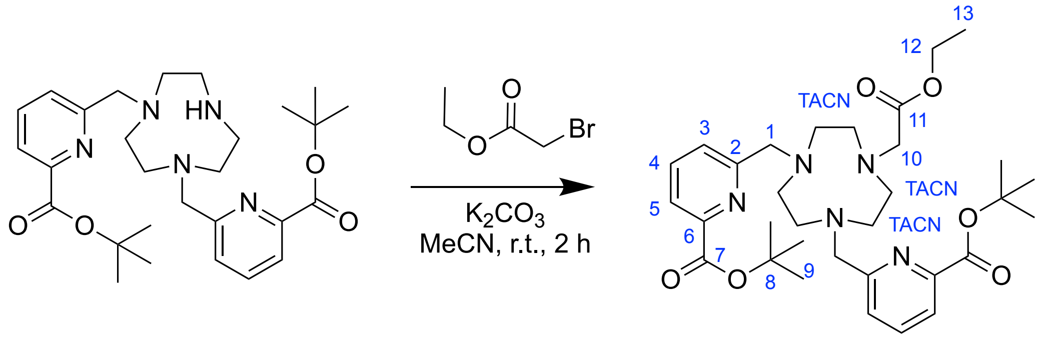


**12** **13**

Compound **12** (136 mg, 266 µmol, 1 eq) was dissolved in 5 mL of dry $CH_3CN$. Potassium carbonate (110 mg, 797 µmol, 3 eq) was added to the stirring solution. The ethyl 2-bromoacetate (44 mg, 266 µmol, 1 eq) was added dropwise under nitrogen and allowed to stir for one hour before it was filtered and solvent was removed. The yellow oil was purified with flash chromatography (method B) and fractions containing compound **13** were lyophilized to give a light yellow oil (139 mg, 87%).

**$^1$H NMR** (500 MHz, $CD_3CN$) δ 7.93 (d, $J$ = 7.8 Hz, 2H, $H_5$), 7.85 (t, $J$ = 7.7 Hz, 2H, $H_4$), 7.61 (d, $J$ = 7.7 Hz, 2H, $H_3$), 4.29 (s, 4H, $H_1$), 4.08 (q, $J$ = 7.1 Hz, 2H, $H_{12}$), 3.35 (s, 2H, $H_{10}$), 3.25 (s, 4H, $H_{TACN}$), 3.05 (t, $J$ = 5.6 Hz, 4H, $H_{TACN}$), 2.92 (t, $J$ = 5.5 Hz, 4H, $H_{TACN}$), 1.55 (s, 18H, $H_9$), 1.19 (t, $J$ = 7.1 Hz, 3H, $H_{13}$).

**$^{13}$C NMR** (126 MHz, $CD_3CN$) δ 171.14 ($C_q$, 1C, $C_{11}$), 164.77, ($C_q$, 2C, $C_7$), 164.77 ($C_q$, 2C, $C_6$), 155.52 ($C_q$, 2C, $C_2$), 138.24, (CH, 2C, $C_4$), 126.76 (CH, 2C, $C_3$), 124.07 ($C_q$, 2C, $C_5$), 82.01 ($C_q$, 2C, $C_8$), 60.33 ($CH_2$, 2C, $C_{12}$), 59.63 ($CH_2$, 2C, $C_1$), 54.95, ($CH_2$, 1C, $C_{10}$), 51.96 ($CH_2$, 2C, $C_{TACN}$), 50.60 ($CH_2$, 2C, $C_{TACN}$), 49.00 ($CH_2$, 2C, $C_{TACN}$), 27.24 ($CH_3$, 6C, $C_9$), 13.53 ($CH_3$, 1C, $C_{13}$).

**HR-ESI-MS:** $[M+H]^+$= calc. 598.3599 , found: 598.3591

**HPLC:** $R_f$= 7.61 mins (method E)

**Synthesis of 15**

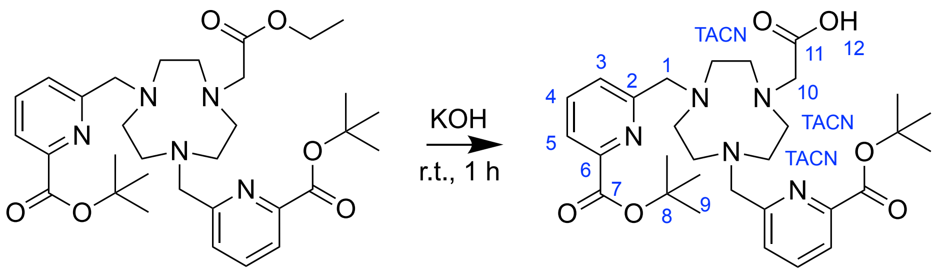


**13** **14**

Compound **13** (164 mg, 274 µmol) was dissolved in 10 mL of 8:2 THF:EtOH. The 1M KOH (2.74 mL, 10 eq) was added to the solution and allowed to stir for 1.5 hours. The solution was then dried on a lyophilizer and purified on a preparative HPLC to give a white powder (method B) (54 mg, 274 µmol, 35%).

**$^{1}$H NMR** (500 MHz, $CD_3CN$) δ 8.14 (s, 1H, $H_{12}$), 7.90 (d, 2H, $H_3$), 7.84 (t, 2H, $H_4$), 7.67 (d, 2H, $H_5$), 4.07 (s, 6H, $H_{TACN}$), 3.56 (s, 2H, $H_{10}$), 3.19 (s, 6H, $H_{TACN}$), 2.84 (s, 4H, $H_1$), 1.56 (s, 18H, $H_9$).

**$^{13}$C NMR** (126 MHz, $CD_3CN$) δ 169.91 ($C_q$, 1C, $C_{11}$), 164.02 ($C_q$, 2C, $C_7$), 162.63 ($C_q$, 2C, $C_6$), 158.01 ($C_q$, 2C, $C_2$), 137.90 (CH, 2C, $C_4$), 126.65 (CH, 2C, $C_3$), 123.56 (CH, 2C, $C_5$), 81.85 ($C_q$, 2C, $C_8$), 60.39 ($CH_2$, 2C, $C_{TACN}$), 57.85 ($CH_2$, 1C, $C_{10}$), 52.37 ($CH_2$, 2C, $C_{TACN}$), 51.19 ($CH_2$, 2C, $C_1$), 49.50 ($CH_2$, 2C, $C_{TACN}$), 27.25 ($CH_3$, 6C, $C_9$).

**HR-ESI-MS:** $[M+H]^+$= calc. 570.3286, found: 570.3280

**HPLC:** $R_f$= 7.16 mins (method E)

**Synthesis of 15**

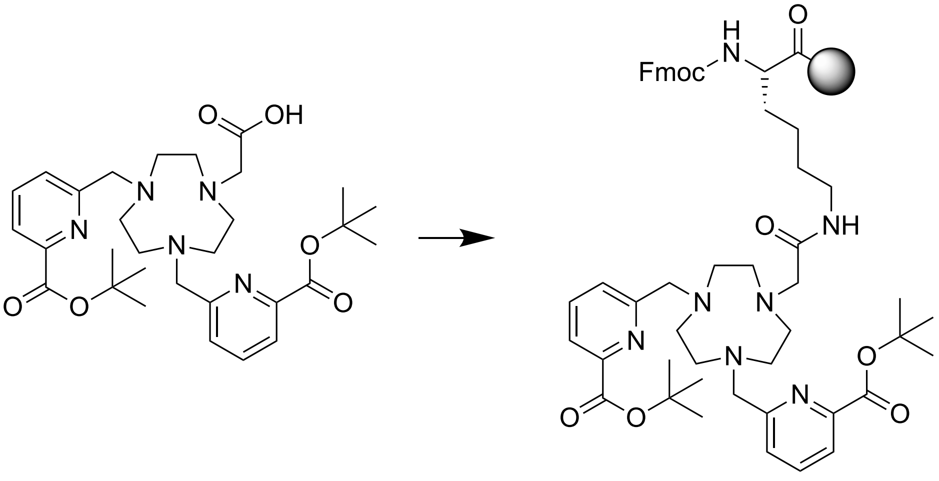


**14** **15**

The Fmoc-Lys(Mtt)-rink (71 mg, 0.80 mmol/g, 63 µmol) was swelled with DMF and the Mtt group removed with 1% TFA in DCM. Compound **14** (54 mg, 95 µmol, 1.5 eq) was dissolved in DMF (1 mL) and stirred with DIPEA (250 mg, 1.9 mmol, 30 eq) for five minutes before adding

PyBOP (160 mg, 320 μmol, 5 eq). The solution was then added to the resin and allowed to shake overnight.

**Synthesis of 16-20**

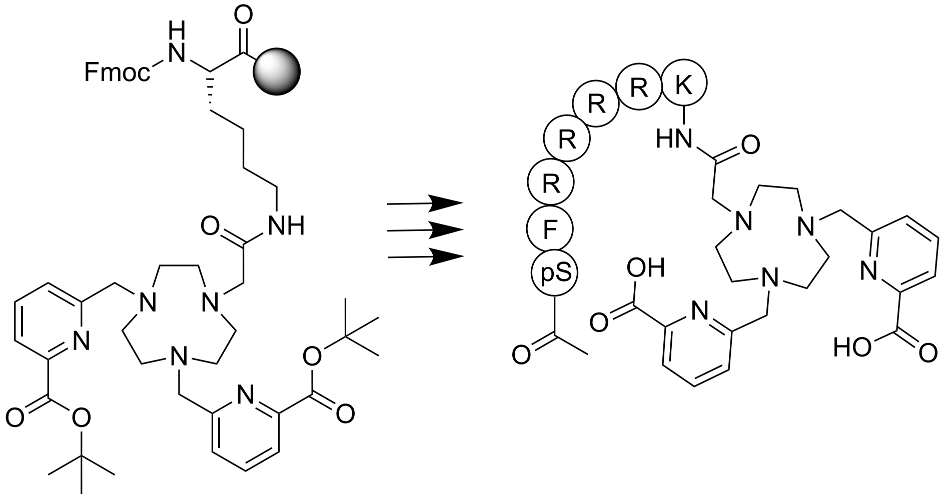


**15** **16**

The peptide was elongated with standard SPPS procedure with Fmoc-Ser(PO(OBzl)OH)-OH, Fmoc-Phe-OH, and Fmoc-Arg(Pbf)-OH. The Fmoc was removed with 20% piperidine in DMF and couplings were performed with 5-fold excess of the L-amino acid, 2-fold excess of PyBOP, and four-fold excess of DIPEA. The PPP derivatives were synthesized with a Fmoc-PPP-OH peptide from AAPPTec (>95% purity, TFA salt). The acetylation was performed with 5 mL of 20% $Ac_2O$ in DMF. Following cleavage (95:2.5:2.5 TFA:water:TIPS), the peptide was washed with cold diethyl ether and purified with a preparative HPLC (method A). The title compound was dried on a lyophilizer to give a white powder.

**Characterization of 16 (AcHN-pSFRRRRK-CONH$_2$):**

**HR-ESI-MS:** $[M+2H]^{2+}$= calc. 738.4017, found: 728.3985; $[M+3H]^{3+}$= calc. 522.6036, found: 522.6014

**HPLC:** $R_t$ = 5.73 mins (method E)

**Yield:** 24.8 mg, 25%

**Characterization of 17 (AcHN-SFRRRRGGGK-CONH$_2$):**

**HR-ESI-MS:** $[M+2H]^{2+}$= calc. 828.9507, found: 828.9505; $[M+3H]^{3+}$= 552.9696, found: 552.9697

**HPLC:** $R_t$ = 7.08 mins (method F)

**Yield:** 5 mg, 8%

**Characterization of 18 (AcHN-pSFRRRRGGGK-CONH$_2$):**

**LR-ESI-MS:** $[M+2H]^{2+}$= calc. 868.9, found: 868.8; $[M+3H]^{3+}$= calc. 579.6, found: 579.8; $[M+4H]^{4+}$= calc. 435.0, found: 435.1

**HPLC:** $R_t$ = 7.04 mins (method D)

**Yield:** 4 mg, 6%

**Characterization of 19 (AcHN-SFRRRRPPPK-CONH$_2$):**

**HPLC:** $R_t$= 7.06 (method D)

**HR-ESI-MS:** $[M+2H]^{2+}$= calc. 888.9977, found: 888.9976; $[M+3H]^{3+}$= calc. 593.0009, found: 593.3357

**Yield:** 29.3 mg, 23%

**Characterization of 20 (AcHN-pSFRRRRPPPK-CONH$_2$):**

**HR-ESI-MS:** $[M+2H]^{2+}$= calc. 928.9809, found: 928.9814; $[M+H+Na]^{2+}$= calc. 939.9725, found 939.9718

**HPLC:** $R_t$ = 7.14 mins (method D)

**Yield:** 8.3 mg, 6%

**Synthesis of [Ln(bispic-PSer)]$^+$**

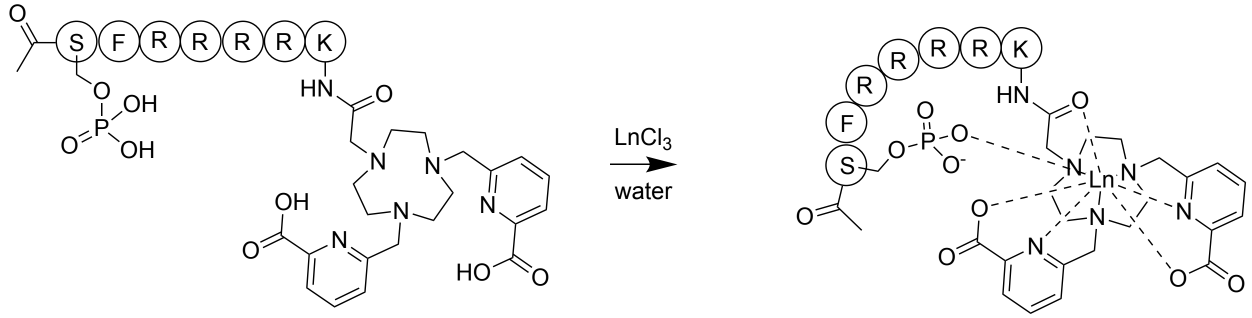


Compound **16** (3 mg, 2 μmol, 1 eq) was dissolved in 200 μL of water. A solution $LnCl_3$ in water (0.0071 g/mL) was made to deliver 1 equivalent of the appropriate $LnCl_3$ to the stirring solution. The pH water adjusted to 5.5 and the reaction was allowed to stir for 30 minutes before purifying on analytical HPLC (method D) (3 mg, 90%, 2 μmol).

**[Tb(bispic-PSer)]$^-$**

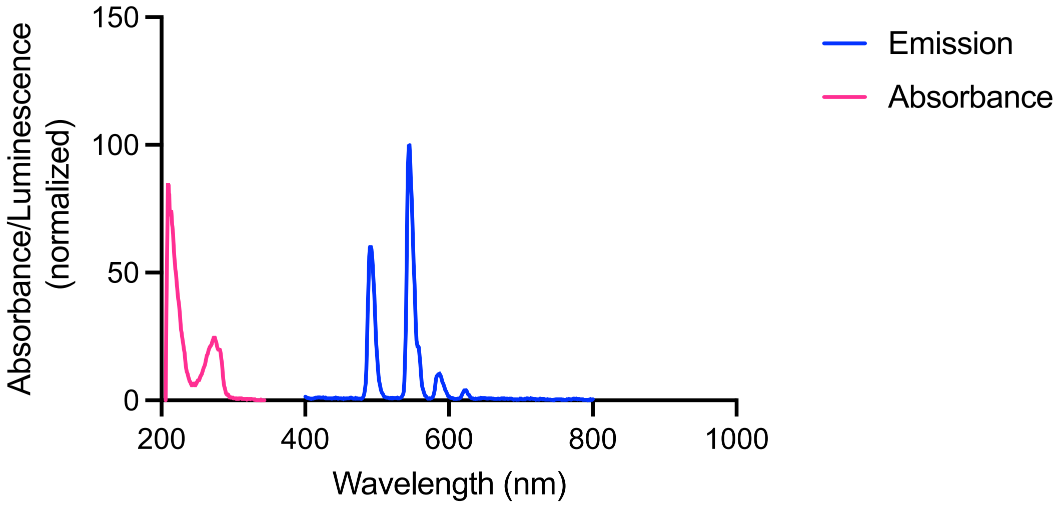


**Figure S16:** Normalized absorbance/emission spectra of [Tb(bispic-PSer)]$^-$. Excitation at 279 nm.

**Photophysical characterization**: $\lambda_{max}$= 274 nm, $\phi$ = 0.70 ± 0.20 (in pH 7.4 0.1 M Tris buffer) $\tau(H_2O)$ = 2.11 ± 0.03 ms, $\tau(D_2O)$ = 2.43 ± 0.01 ms, q= 0.0

**HPLC:** $R_t$: 11.53 min (Method I)

**HR-ESI-MS:** $[M+H]^{2+}$= calc. 861.3526, found 861.3496; $[M+2H]^{3+}$= calc. 574.5708, found 574.5692; $[M+3H]^{4+}$ calc. 431.1800, found 431.1789

**[Eu(bispic-PSer)]$^-$**

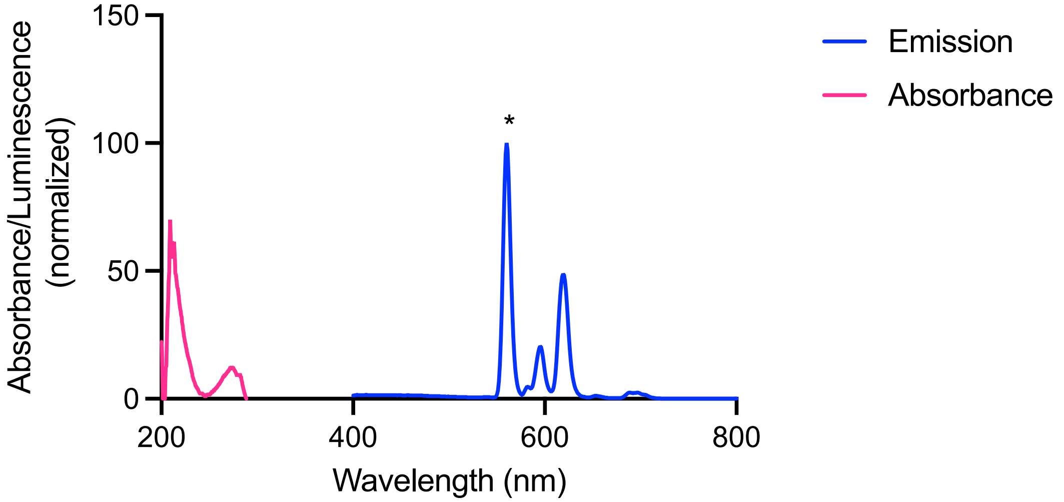


**Figure S17:** Normalized absorbance/emission spectra of [Eu(bispic-PSer)]$^-$. Excitation at 279 nm. (*double excitation signal observed at 558 nm)

**Photophysical characterization**: $\lambda_{max}$= 274 nm, $\phi$ = 0.039 ± 0.006 (in pH 7.4 0.1 M Tris buffer) $\tau(H_2O)$ = 0.67 ± 0.05 ms, $\tau(D_2O)$ = 1.7 ± 0.2 ms, q= 0.8

**HPLC:** $R_t$: 7.18 min (Method F)

**HR-ESI-MS:** $[M+H]^{2+}$= calc. 858.3506, found 858.3528; $[M+2H]^{3+}$ 572.5695, found 572.5716; $[M+3]^{4+}$ calc. 429.6789, found 429.6809

**[Lu(bispic-PSer)]$^{-}$**

**HPLC:** $R_t$: 7.02 min (Method D)

**HR-ESI-MS:** $[M+H]^{2+}$= calc. 869.3604, found 869.3610; $[M+2H]^{3+}$ 579.9093, found 369.9103; $[M+3]^{4+}$ calc. 435.1838, found 435.1847

**[Tb(bispic-GGG-Ser)]$^{+}$**

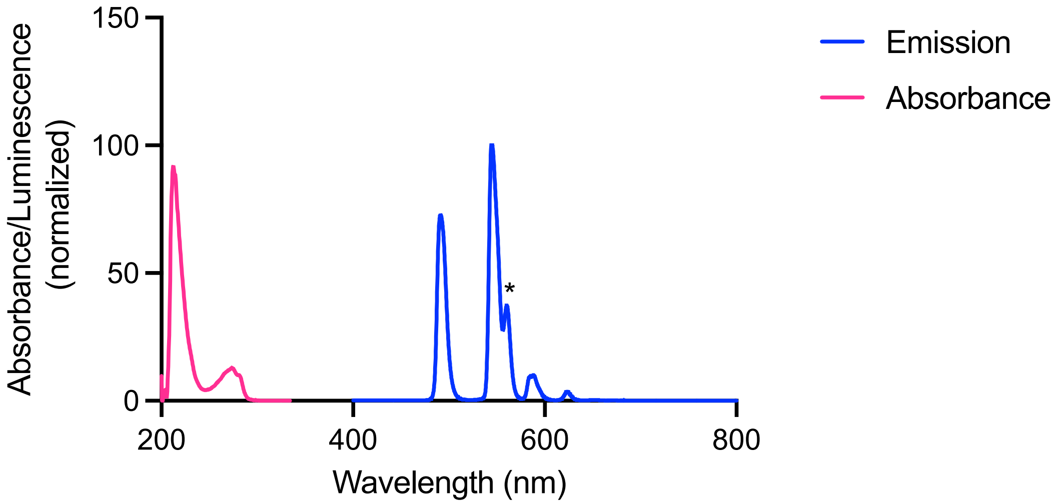


**Figure S18:** Normalized absorbance/emission spectra of [Tb(bispic-GGG-Ser)]$^{+}$. Excitation at 279 nm. (*double excitation signal observed at 558 nm)

**Photophysical characterization**: $\lambda_{max}$= 274 nm, $\phi$ = 0.44 ± 0.09 (in pH 7.4 0.1 M Tris buffer) $\tau(H_2O)$ = 1.446 ± 0.009 ms, $\tau(D_2O)$ = 2.28 ± 0.02 ms, q= 1.0

**HPLC:** $R_t$ : 6.9 min (Method D)

**HR-ESI-MS:** calc. $[M+3H]^{4+}$= calc. 453.9550, found: 453.9546

**[Tb(bispic-GGG-PSer)]$^{-}$**

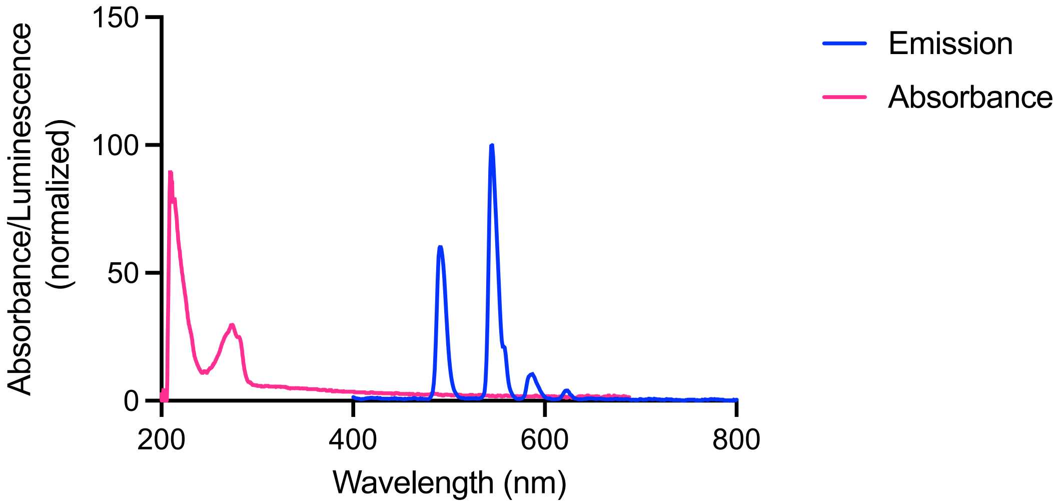


**Figure S19:** Normalized absorbance/emission spectra of [Tb(bispic-GGG-PSer)]$^{-}$. Excitation at 279 nm.

**Photophysical characterization**: $\lambda_{max}$= 274 nm, $\phi$ = 0.572 ± 0.009 (in pH 7.4 0.1 M Tris buffer) $\tau(H_2O)$ = 1.94 ± 0.02 ms, $\tau(D_2O)$ = 2.605 ± 0.002 ms, q= 0.4

**HPLC:** $R_t$: 7.11 min (Method D)

**HR-ESI-MS:** $[M+3H]^{4+}$= calc. 473.9466, found 473.9470; $[M+2H]^{3+}$= calc. 631. 5929, found 631.5933

**Eu(bispic-GGG-Ser)]$^{+}$**

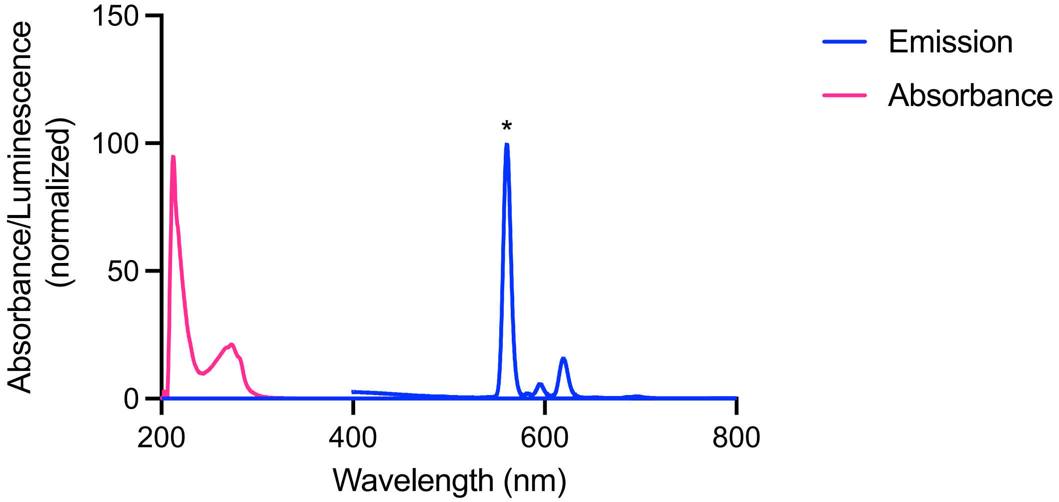


**Figure S20:** Normalized absorbance/emission spectra of [Eu(bispic-GGG-Ser)]$^{+}$. Excitation at 279 nm. (*double excitation signal observed at 558 nm)

**Photophysical characterization**: $\lambda_{max}$= 274 nm, $\phi$ = 0.02 ± 0.01 (in pH 7.4 0.1 M Tris buffer) $\tau(H_2O)$ = 0.56 ± 0.02 ms, $\tau(D_2O)$ = 1.56 ± 0.02 ms, q= 1.1

**HPLC:** $R_t$: 6.96 min (Method D)

**HR-ESI-MS:** $[M+3H]^{4+}$= calc. 452.2051, found 452.4543; $[M+4H]^{5+}$= calc. 362.1674, found 362.1654.

**Eu(bispic-GGG-PSer)]⁻**

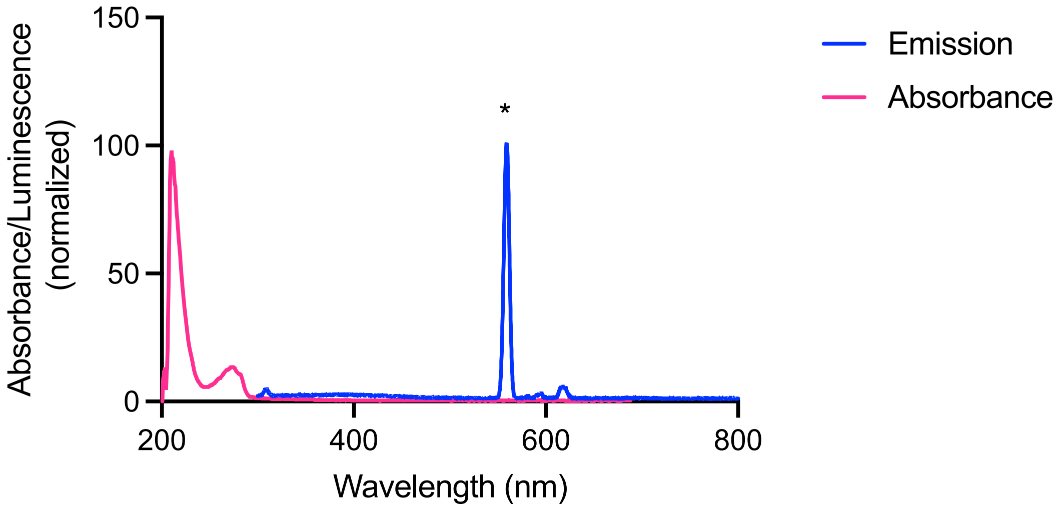


**Figure S21:** Normalized absorbance/emission spectra of [Eu(bispic-GGG-PSer)]⁻. Excitation at 279 nm (*double excitation signal observed at 558 nm).

**Photophysical characterization**: $\lambda_{max}$= 274 nm, $\phi$ = 0.02 ± 0.01 (in pH 7.4 0.1 M Tris buffer) $\tau(H_2O)$ = 0.67± 0.05 ms, $\tau(D_2O)$ = 1.55 ± 0.04 ms, q= 0.7

**HPLC:** $R_t$: 7.07 min (Method D)

**HR-ESI-MS:** $[M+3H]^{4+}$= calc. 472.4456, found 472.4459; $[M+2H]^{3+}$= calc. 629.5912, found 629.5921

**[Tb(bispic-PPP-Ser)]⁺**

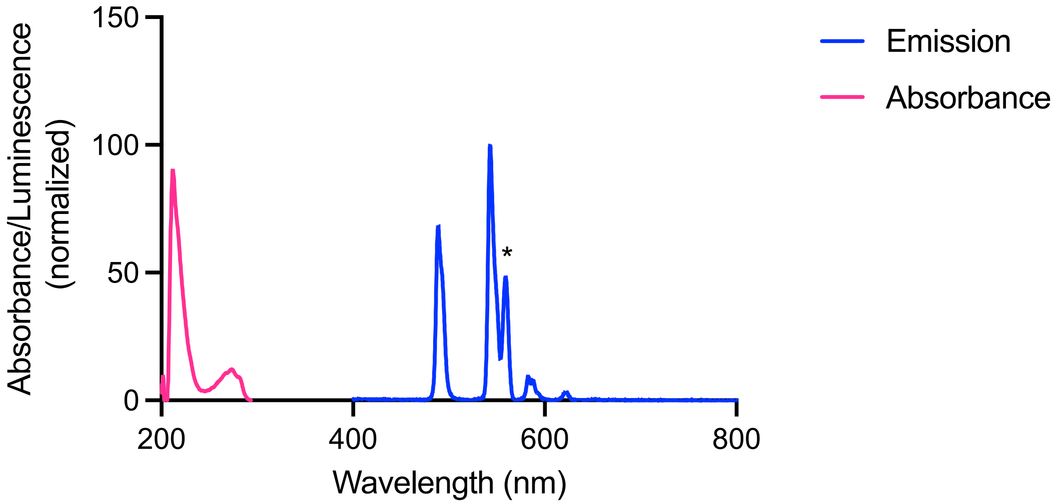


**Figure S22:** Normalized absorbance/emission spectra of [Tb(bispic-PPP-Ser)]$^+$. Excitation at 279 nm. (*double excitation signal observed at 558 nm)

**Photophysical characterization**: $\lambda_{max}$= 274 nm, $\phi$ = 0.40 ± 0.03 (in pH 7.4 0.1 M Tris buffer) $\tau(H_2O)$ = 1.452 ± 0.009 ms, $\tau(D_2O)$ = 2.35 ± 0.02 ms, q= 1.0

**HPLC:** $R_t$: 7.00 min (Method D)

**MALDI:** $[M+4H]^{4+}$= calc. 484.2304, found: 484.2286; $[M+5H]^{5+}$= calc. 387.5859, found: 387.5844

**[Tb(bispic-PPP-PSer)]$^-$**

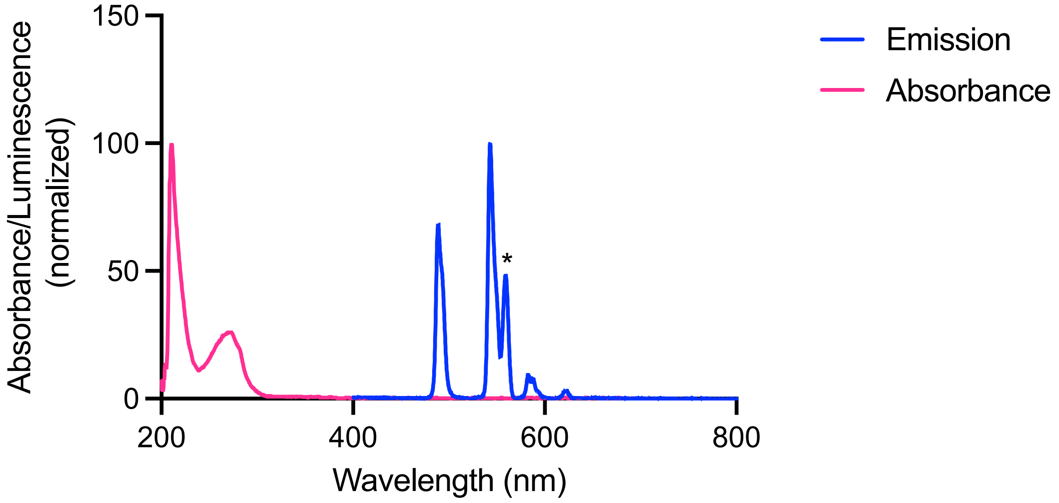


**Figure S23:** Normalized absorbance/emission spectra of [Tb(bispic-PPP-PSer)]$^-$. Excitation at 279 nm. (*double excitation signal observed at 558 nm)

**Photophysical characterization**: $\lambda_{max}$= 274 nm, $\phi$ = 0.5 ± 0.2 (in pH 7.4 0.1 M Tris buffer) $\tau(H_2O)$ = 1.578 ± 0.007 ms, $\tau(D_2O)$ = 2.45 ± 0.03 ms, q= 0.8

**HPLC:** $R_t$: 7.11 min (Method D)

**HR-ESI-MS:** $[M+2H]^{3+}$: calc. 671.6242, found: 671.6232; $[M+3H]^{4+}$: calc. 503.9701, found: 503.9697

**Eu(bispic-PPP-Ser)]$^+$**

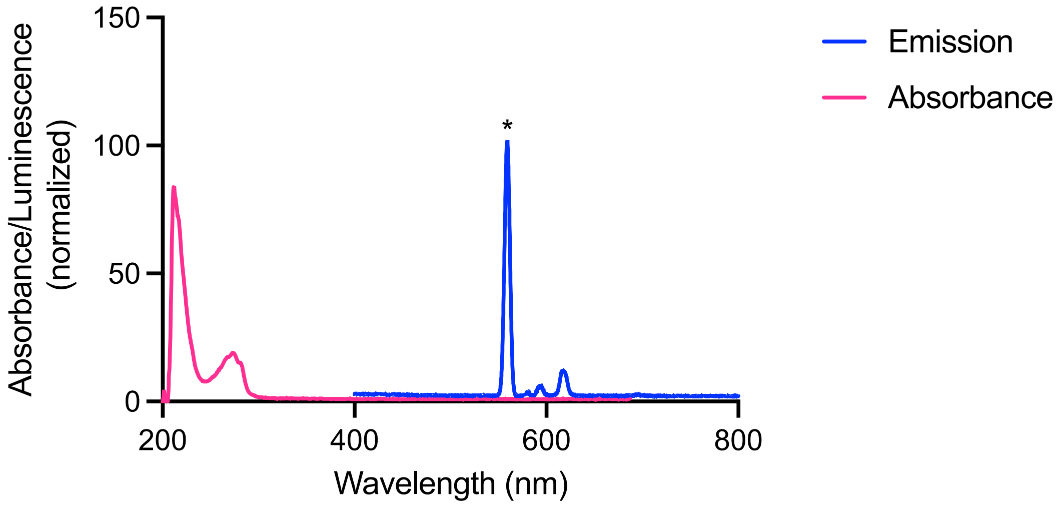


**Figure S24:** Normalized absorbance/emission spectra of [Eu(bispic-PPP-Ser)]$^+$. Excitation at 279 nm. (*double excitation signal observed at 558 nm)

**Photophysical characterization**: $\lambda_{max}$= 274 nm, $\phi$ = 0.013 ± 0.005 (in pH 7.4 0.1 M Tris buffer) $\tau(H_2O)$ = 0.532 ± 0.001 ms, $\tau(D_2O)$ = 1.549 ± 0.003 ms, q= 1.2

**HPLC:** 7.01 min (method D)

**HR-ESI-MS:** $[M+3H]^{4+}$: calc. 482.7294, found: 482.4775; $[M+4H]^{5+}$: calc. 386.3851, found: 386.1839.

**Eu(bispic-PPP-PSer)]$^-$**

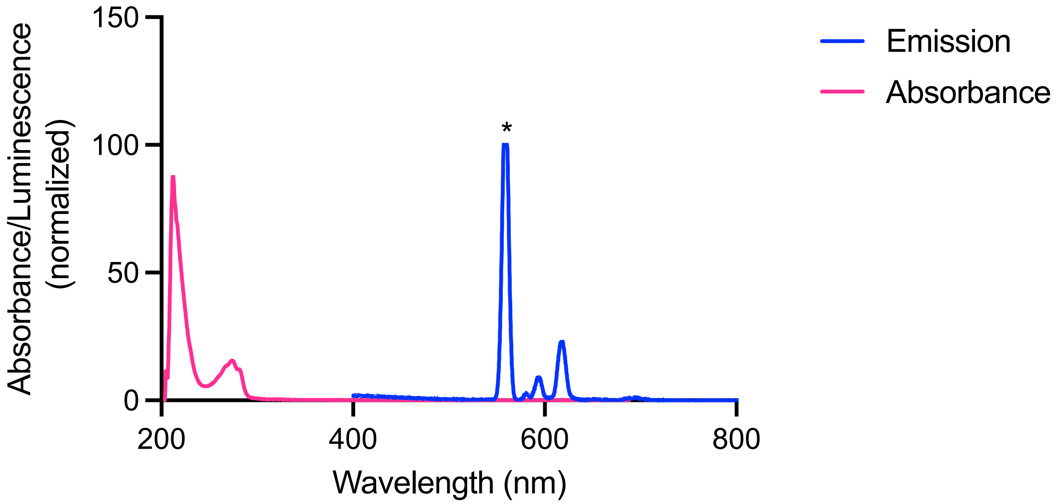


**Figure S25:** Normalized absorbance/emission spectra of [Eu(bispic-PPP-PSer)]$^-$. Excitation at 279 nm. (*double excitation signal observed at 558 nm)

**Photophysical characterization**: $\lambda_{max}$= 274 nm, $\phi$ = 0.07 ± 0.01 (in pH 7.4 0.1 M Tris buffer) $\tau(H_2O)$ = 0.65 ± 0.01 ms, $\tau(D_2O)$ = 1.609 ± 0.006 ms, q= 0.8

**HPLC:** $R_t$: 7.31 min (Method D)

**HR-ESI-MS:** $[M+H]^{2+}$ calc. 1003.9297, found: 1003.9310; $[M+2H]^{3+}$ calc. 669.6222, found: 669.6234.

**Radioactive and Optical Imaging Experiments**

$^{68}$Ga was received from the University of Wisconsin Radiopharmaceutical Production Facility from a $^{68}$Ge/$^{68}$Ga-Generator and eluted in 0.1 M HCl (pH 2).

## 3.1 Radiochemical Synthesis Protocols

**HPLC analysis of radiochemical complexes:** A quantitatively (>95%) radiolabeled sample was determined by radioHPLC for each radiochemical complex to confirm a single radiochemical species.

**AT$^{32}$P radiolabeling and formulation for Cerenkov Radiation Energy Transfer Plate.** To a 25 uL solution (20 mM pH 7.4 HEPES, 5 mM $MgCl_2$, 0.44 mM $CaCl_2$, 2 mM DTT, 0.024 mg phosphatidylserine, 0.005 mg diacylglycerol) was added [Tb(bispic-PSer)]$^-$, 1.6 µL, 0.5 nmol; PKC$\alpha$ 5.6 µL, 0.28 µg; and an aliquot of [$\gamma$-$^{32}$P]ATP, 3.9 µL, 50 µCi. The solution was allowed to sit at 37 °C for one hour. Thirty second fractions were collected from a radioHPLC (method D) before the trace was reconstructed with an LSC.
The intermolecular plate (excited by ATP-$^{32}$P) was supplemented with 0.1 nmol of **[Tb(bispic-Ser)]$^+$** per well to account for the molar activity of the **[Tb(bispic-$^{32}$PSer)]$^-$** radiolabeling.

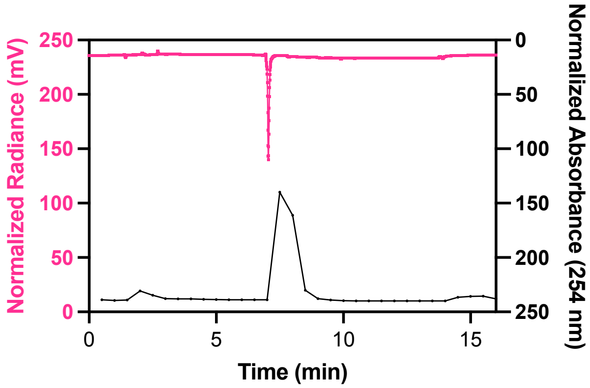


**Figure S26:** Stacked ligand, $^{nat}P$ complex and $^{32}P$ complex HPLC chromatograms (Method D). [$^{nat}P$][Tb(**bispic-PSer**)]$^-$ $t_R$ = 7.06 min.; [$^{32}P$][Tb(**bispic-PSer**)]$^-$ $t_R$ = 7.5 min.

### 3.2 Phantom Image Assays

Phantom images were recorded on an IVIS Lumina series III from Caliper LifeSciences or Lago optical imaging system from Spectral Instruments Imaging small animal imager. The collection time was five minutes. All wells were doped with 10 uL of Cerenkov emitter resulting in a total volume of 210 μL. Image analysis was completed with Aura software. The

[$^{161}$Tb][Tb(trispic)] phantom image assay was performed with 1 mm turkey slices to simulate tissue.

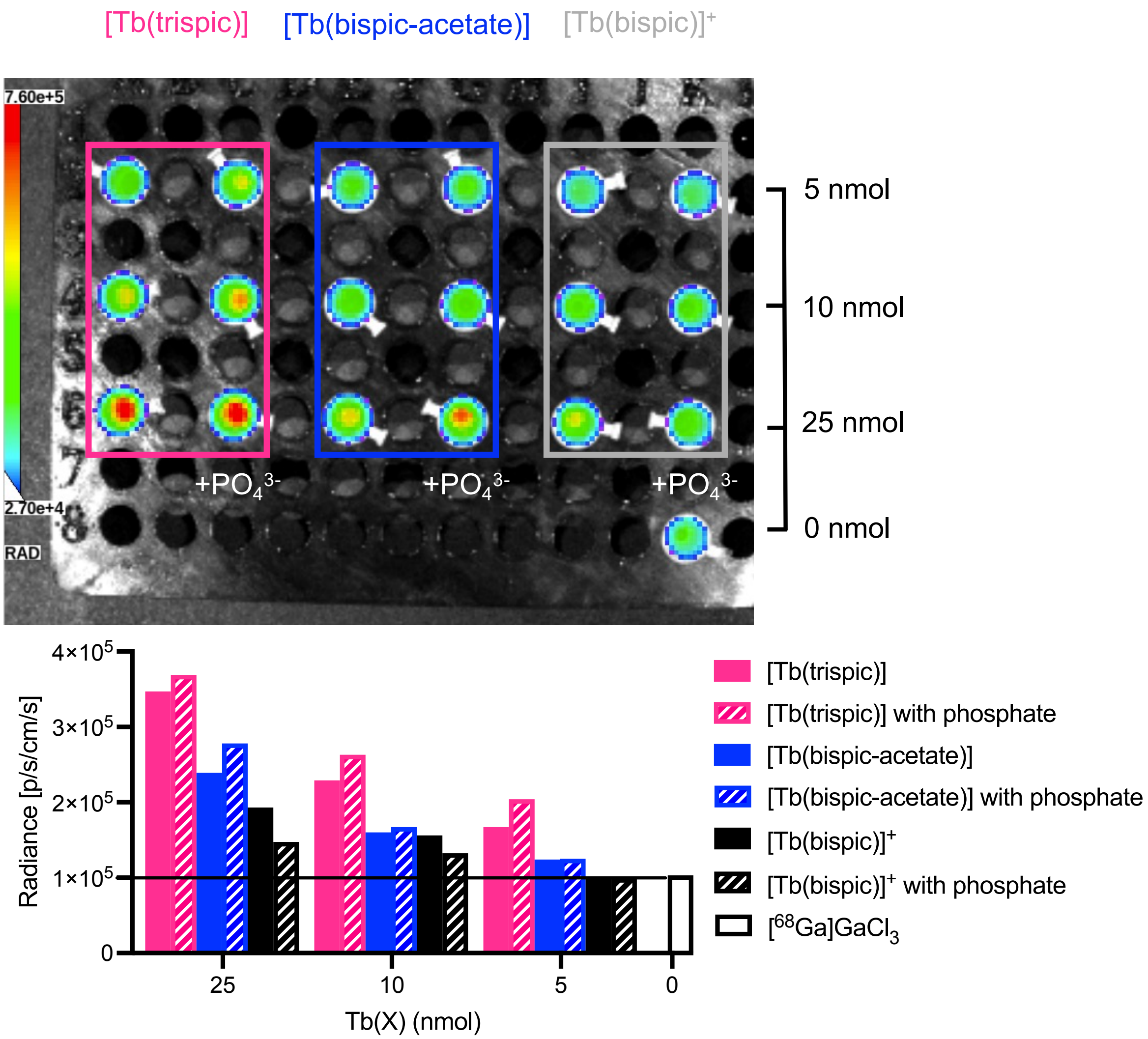


**Figure S27:** Phantom image and ROI analysis of dilution series of Tb(III) complexes in the presence of the Cerenkov emitting [$^{68}$Ga]$GaCl_3$ (10 µCi/well). Images were performed with a 540 nm filter. Solutions were prepared in 100 mM pH 7.4 HEPES buffer. Samples containing $PO_4^{3-}$ were prepared with 20 equivalents of PBS buffer.

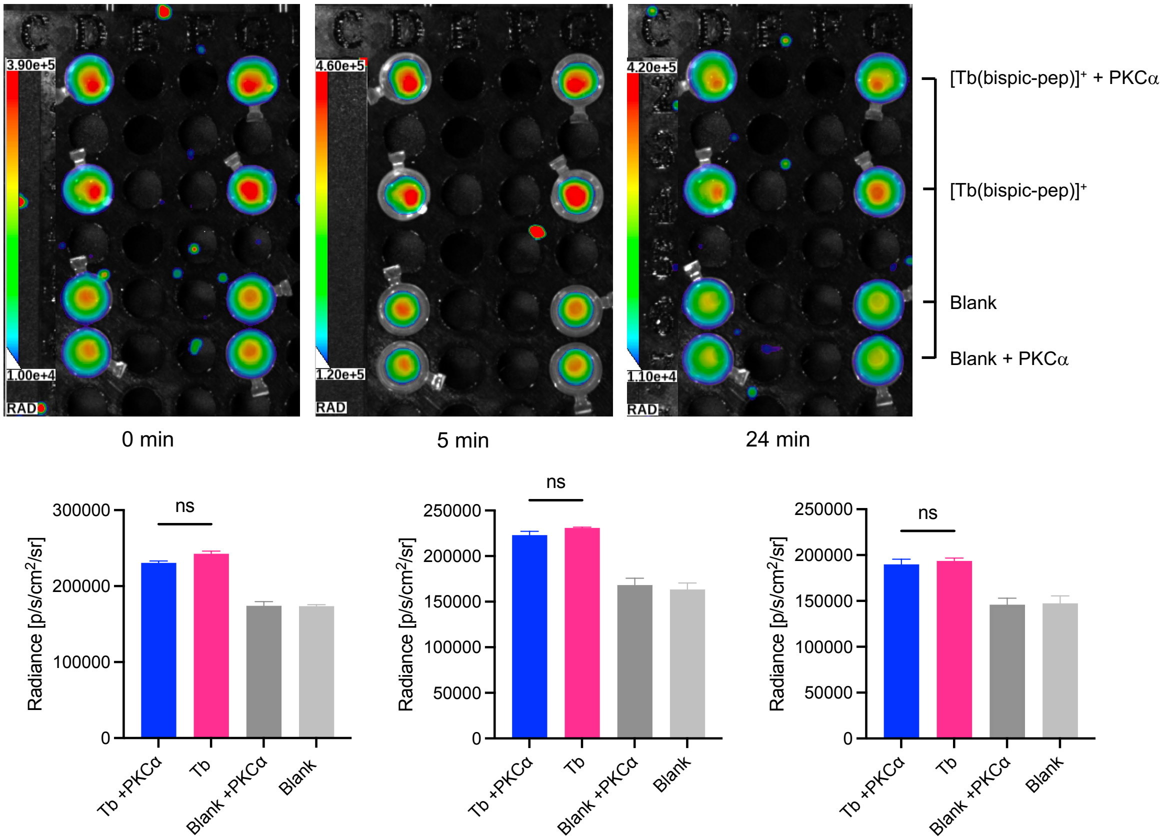


**Figure S28:** Phantom images of [Tb(bispic-Ser)]$^+$ (15 nmol/well) in the presence of the Cerenkov emitting [$^{68}$Ga]$GaCl_3$ 0 min (11 µCi), 5 min (11 µCi), and 24 min (10 µCi) phosphorylation with PKCα. Images were performed with a 620 nm filter. Solutions were prepared in 15 mM pH 7.4 HEPES buffer.

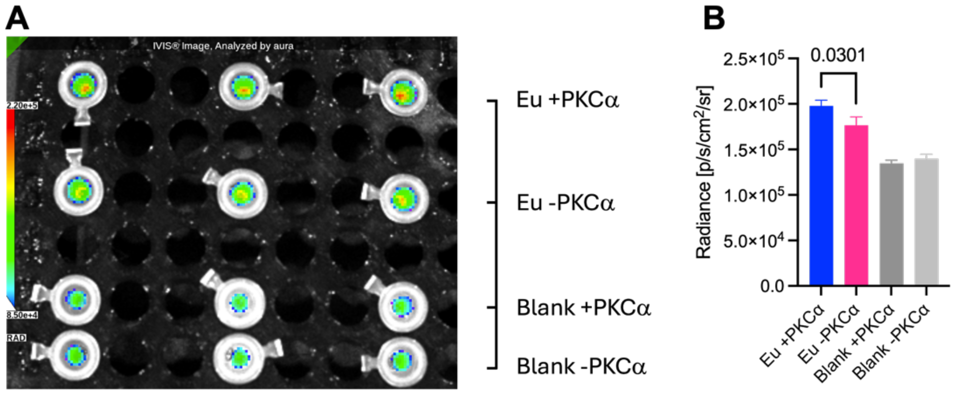


**Figure S29**:(a) Phantom image and (b) ROI analysis of [Eu(bispic-Ser)]$^+$ (40 nmol/well) in the presence of the Cerenkov emitting [$^{68}$Ga]$GaCl_3$ after phosphorylation with PKCα. Images were performed with a 620 nm filter. Solutions were prepared in 15 mM pH 7.4 HEPES buffer. Statistical significance determined with an unpaired t test.

## Luminescence Studies

### 4.1 Luminescence Titrations

General Procedure: A 1 mL solution of complex in pH 7.4 water was added to a quartz cuvette to achieve an absorbance of 0.1 (12.6-13.4 μM). The desired titrant ($PO_4^{3-}$, phosphorylated serine, or serine) was added in steps of 0.1 eq until 1.5 eq is reached for [Tb(trispic)] or [Tb(bispic-acetate)] or until 2.5 eq is reached for $[Tb(bispic)]^+$ taking an emission spectrum after each addition. Titrant was then added in steps of 0.5 eq until 8 equivalents is reached for [Tb(trispic)] or [Tb(bispic-acetate)] or 10 for $[Tb(bispic)]^+$.The cuvette was soaked in 5% nitric acid overnight between trials.

Parameters: Ex WL- 285 nm; Em WL- 300-800 nm; scan speed-1200 nm/min; Ex slits- 1 nm; Em slit- 1 nm; PM voltage- 700 V; response=0.05 s

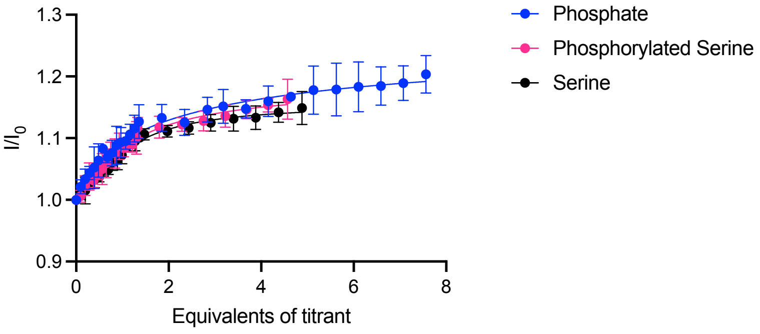


**Figure S30:** $[Tb(bispic\text{-}amide)]^+$ luminescence titration

### 4.2 Eu(bispic-lysine) Quantum Yield Comparisons

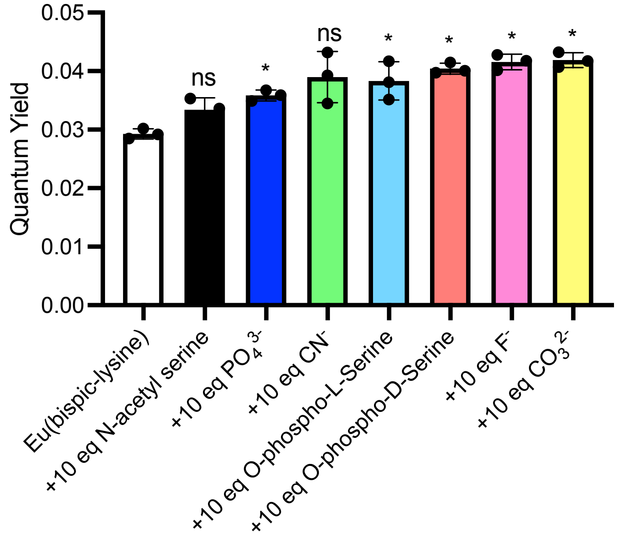


**Figure S31:** Eu(bispic-lysine) quantum yield comparisons in the presence of 10 equivalents of relevant substrates.

| Eu(bispic-lysine) | + N-acetyl serine | + $PO_4^{3-}$ | + $CN^-$ | O-phospho-L-Serine | O-phospho-D-Serine | $F^-$ | $CO_3^{2-}$ |
|---|---|---|---|---|---|---|---|
| 2.92 ± 0.09 | 3.3 ± 0.2 | 3.58 ± 0.09 | 3.9 ± 0.04 | 3.8 ± 0.03 | 4.04 ± 0.09 | 4.2 ± 0.01 | 4.2 ± 0.01 |

**Table S3. Quantum yields of Eu(bispic-lysine) with 10 eq. of relevant substrates (%)**

## 4.3 Tb(bispic-lysine) Quantum Yield Comparisons

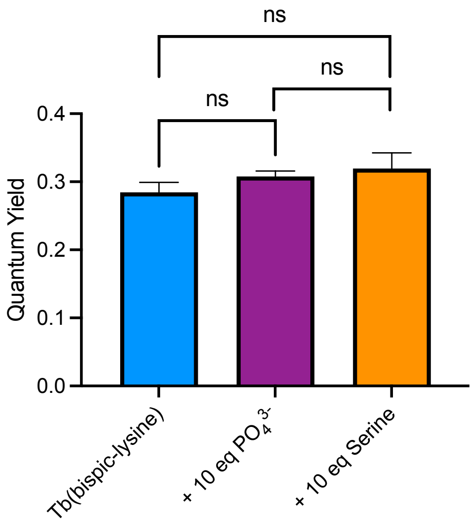


**Figure S32:** Tb(bispic-lysine) quantum yield comparisons in the presence of 10 equivalents of relevant substrates.

**Table S4. Quantum yields of Tb(bispic-lysine) with 10 eq. of relevant substrates (%)**

| Tb(bispic-lysine) | + N-acetyl serine | + $PO_4^{3-}$ |
|---|---|---|
| 30.8 ± 0.8 | 32 ± 2 | 30.8 ± 0.08 |

## 4.4 Eu(bispic-acetate) Quantum Yield Comparisons

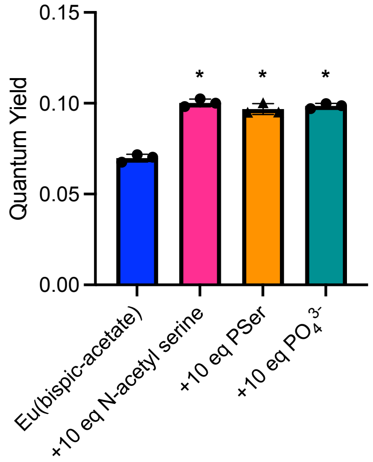


**Figure S33:** Eu(bispic-acetate) quantum yield comparisons in the presence of 10 equivalents of relevant substrates.

**Table S5. Quantum yields of Eu(bispic-lysine) with 10 eq. of relevant substrates (%)**

| Eu(bispic-acetate) | + N-acetyl serine | + $PO_4^{3-}$ | + O-phospho-L-Serine |
|---|---|---|---|
| 6.9 ± 0.02 | 10.0 ± 0.2 | 9.9 ± 0.01 | 9.7 ± 0.03 |

## 4.5 Phosphorylation with PKCα of [natLn][(Ln(bispic-peptides)]

A cuvette containing a 200 μL solution (20 mM HEPES, 2.5 mM $MgCl_2$, 0.225 mM CaCl2, 500 μM ATP, 1 mM DTT, 0.1 μg phosphatidylserine, 0.02 μg diacylglycerol, 20 μM peptide, pH 7.4) was monitored at its peak emission wavelength (Eu=616 nm, Tb=545 nm) for 10 minutes. To the solution, 0.136 μg of PKCα was added and the luminescence was monitored for another 60 minutes.

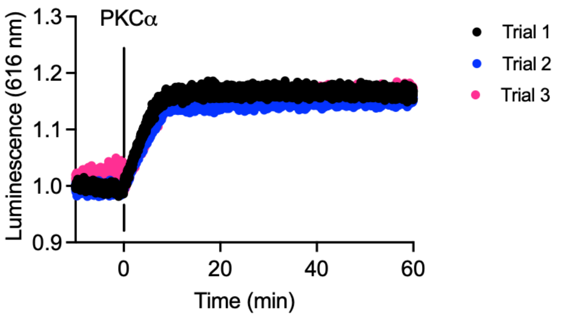


**Figure S34:** Luminescence at max emission wavelength (616 nm) tracking the phosphorylation of $[Eu(bispic\text{-}Ser)]^{+}$ with the kinase, PKCα

Parameters for Eu complexes: Ex WL- 274 nm; Em WL- 616 nm; delay-0.0 s; Ex slits- 2.5 nm; Em slit- 5.0 nm; PM voltage- 700 V; response=0.5 s

Parameters for Tb complexes: Ex WL- 280 nm; Em WL- 545 nm; delay-0.0 s; Ex slits- 2.5 nm; Em slit- 1.0 nm; PM voltage- 700 V; response=0.5 s

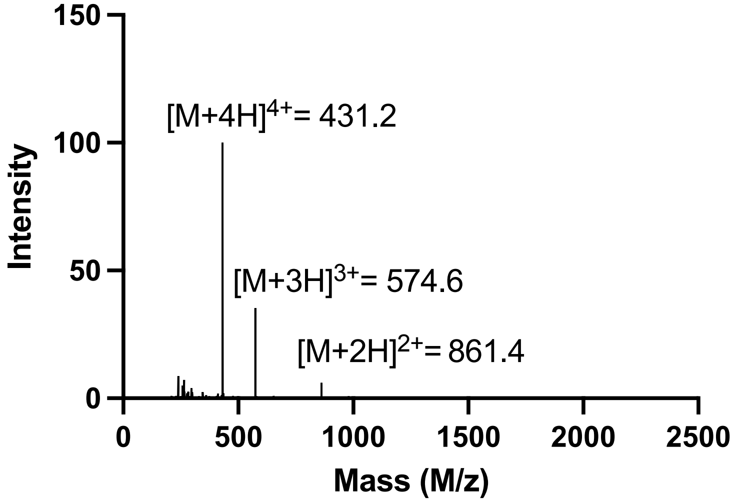

**Figure S35:** Low resolution mass spectrum following phosphorylation of [Tb(bispic-Ser)]$^+$

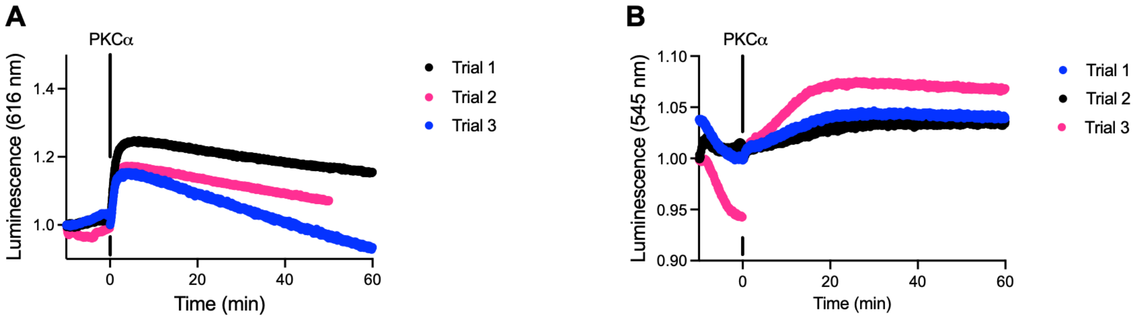


**Figure S36:** Luminescence at max emission wavelength for (a), Tb(III) (545 nm), and (b), Eu(III) (616 nm) tracking the phosphorylation of [Ln(bispic-GGG-Ser)]$^+$ with the kinase, PKCα

Parameters for Eu complexes: Ex WL- 274 nm; Em WL- 616 nm; delay-0.0 s; Ex slits- 5 nm; Em slit- 10.0 nm; PM voltage- 700 V; response=0.5 s

Parameters for Tb complexes: Ex WL- 280 nm; Em WL- 545 nm; delay-0.0 s; Ex slits- 2.5 nm; Em slit- 1.0 nm; PM voltage- 700 V; response=0.5 s

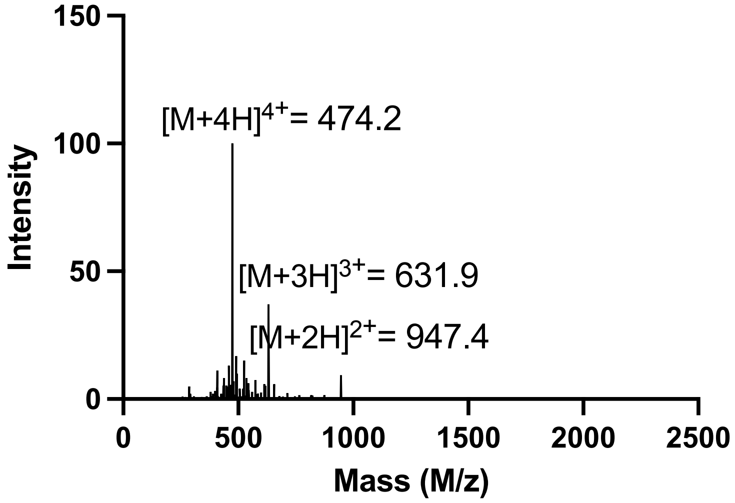


**Figure S37:** Low resolution mass spectrum following phosphorylation of [Tb(bispic-GGG-Ser)]$^+$

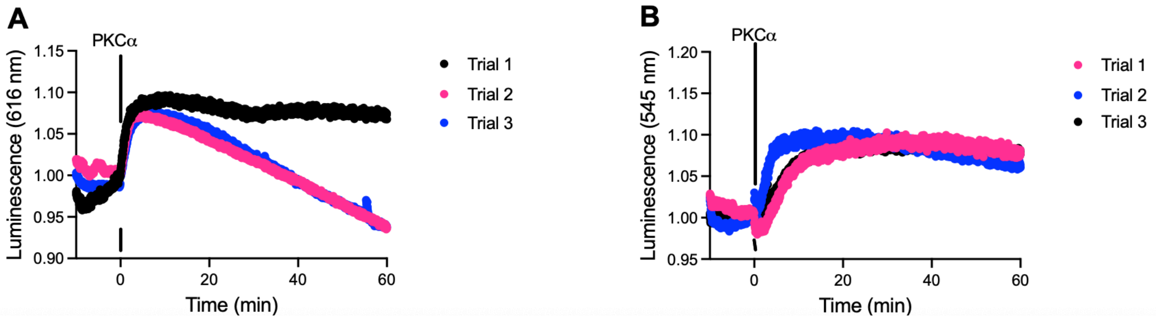

**Figure S38:** Luminescence at max emission wavelength for (a), Tb(III) (545 nm), and (b), Eu(III) (616 nm) tracking the phosphorylation of [Ln(bispic-PPP-Ser)]$^{+}$ with the kinase, PKCα

Parameters for Eu complexes: Ex WL- 274 nm; Em WL- 616 nm; delay-0.0 s; Ex slits- 5 nm; Em slit- 10.0 nm; PM voltage- 700 V; response=0.5 s

Parameters for Tb complexes: Ex WL- 280 nm; Em WL- 545 nm; delay-0.0 s; Ex slits- 2.5 nm; Em slit- 1.0 nm; PM voltage- 700 V; response=0.5 s

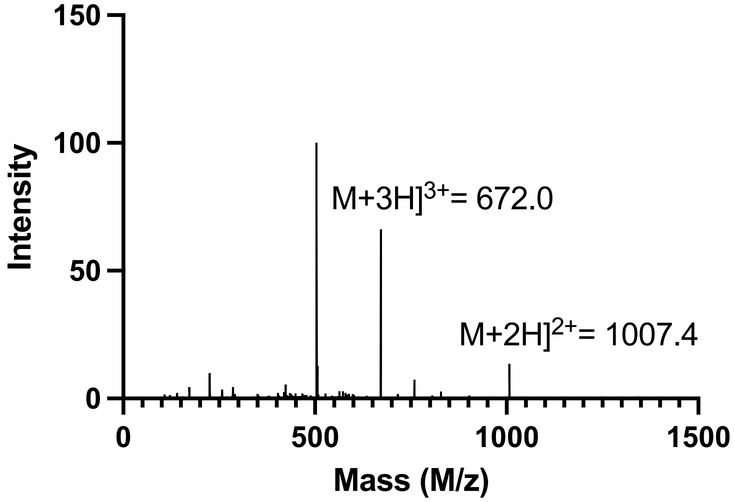


**Figure S39:** Low resolution mass spectrum following phosphorylation of [Tb(bispic-PPP-Ser)]$^{+}$

## 4.6 Water Lifetime Experiments

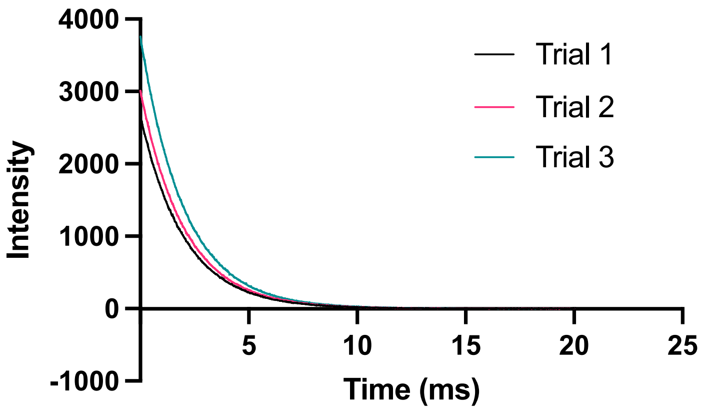


| One phase decay | | | |
|---|---|---|---|
| **Best-fit values** | | | |
| Y0 | 2665 | 3012 | 3761 |
| Plateau | 0.2576 | 0.5008 | 0.8523 |
| K | 0.4941 | 0.4944 | 0.4963 |
| Half Life | 1.403 | 1.402 | 1.397 |
| Tau | 2.024 | 2.023 | 2.015 |
| Span | 2665 | 3012 | 3760 |
| **95% CI (profile likelihood)** | | | |
| Y0 | 2664 to 2666 | 3011 to 3013 | 3759 to 3762 |
| Plateau | -0.006158 to 0.5 | 0.2296 to 0.7718 | 0.5425 to 1.162 |
| K | 0.4937 to 0.4945 | 0.4940 to 0.4947 | 0.4959 to 0.4966 |
| Half Life | 1.402 to 1.404 | 1.401 to 1.403 | 1.396 to 1.398 |
| Tau | 2.022 to 2.025 | 2.021 to 2.024 | 2.014 to 2.016 |
| **Goodness of Fit** | | | |
| Degrees of Freedom | 1998 | 1997 | 1998 |
| R squared | 0.9999 | 0.9999 | 0.9999 |
| Sum of Squares | 43102 | 45467 | 59570 |
| Sy.x | 4.645 | 4.772 | 5.460 |
| **Constraints** | | | |
| K | K > 0 | K > 0 | K > 0 |
| | | | |
| **Number of points** | | | |
| # of X values | 2001 | 2001 | 2001 |
| # Y values analyzed | 2001 | 2000 | 2001 |

**Figure S40:** Tb(trispic) lifetimes in water

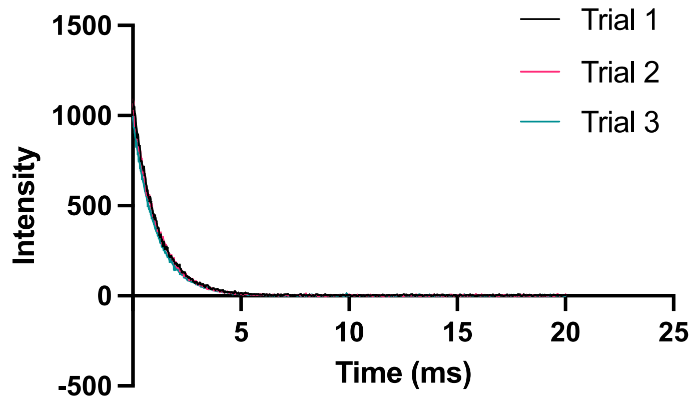


| One phase decay | | | |
|---|---|---|---|
| **Best-fit values** | | | |
| Y0 | 1091 | 1068 | 984.2 |
| Plateau | 3.085 | 1.948 | 1.611 |
| K | 0.9223 | 0.9147 | 0.9321 |
| Half Life | 0.7516 | 0.7578 | 0.7437 |
| Tau | 1.084 | 1.093 | 1.073 |
| Span | 1088 | 1066 | 982.6 |
| **95% CI (profile likelihood)** | | | |
| Y0 | 1089 to 1093 | 1066 to 1070 | 982.6 to 985.8 |
| Plateau | 2.821 to 3.350 | 1.686 to 2.210 | 1.401 to 1.821 |
| K | 0.9197 to 0.92 | 0.9122 to 0.91 | 0.9298 to 0.93 |
| Half Life | 0.7495 to 0.75 | 0.7556 to 0.75 | 0.7418 to 0.74 |
| Tau | 1.081 to 1.087 | 1.090 to 1.096 | 1.070 to 1.076 |
| **Goodness of Fit** | | | |
| Degrees of Freedom | 1998 | 1998 | 1998 |
| R squared | 0.9990 | 0.9990 | 0.9992 |
| Sum of Squares | 56838 | 55937 | 36056 |
| Sy.x | 5.334 | 5.291 | 4.248 |
| **Constraints** | | | |
| K | K > 0 | K > 0 | K > 0 |
| | | | |
| **Number of points** | | | |
| # of X values | 2001 | 2001 | 2001 |
| # Y values analyzed | 2001 | 2001 | 2001 |

**Figure S41:** Eu(trispic) lifetimes in water

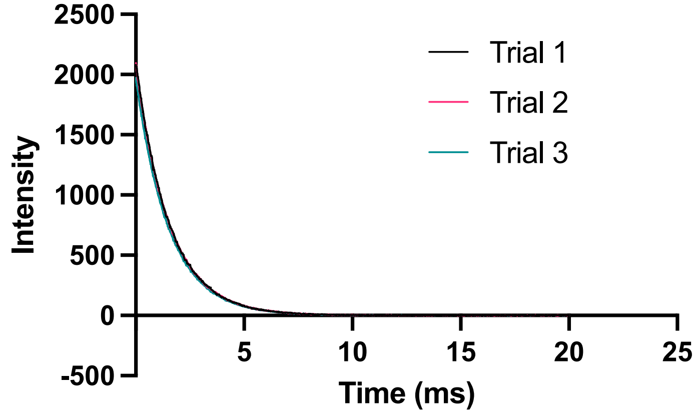


| One phase decay | | | |
|---|---|---|---|
| **Best-fit values** | | | |
| Y0 | 2107 | 2090 | 1975 |
| Plateau | 0.5552 | 0.5047 | 0.7134 |
| K | 0.6576 | 0.6553 | 0.6569 |
| Half Life | 1.054 | 1.058 | 1.055 |
| Tau | 1.521 | 1.526 | 1.522 |
| Span | 2106 | 2089 | 1974 |
| **95% CI (profile likelihood)** | | | |
| Y0 | 2106 to 2108 | 2089 to 2091 | 1974 to 1976 |
| Plateau | 0.3524 to 0.7579 | 0.3297 to 0.6797 | 0.5249 to 0.9018 |
| K | 0.6570 to 0.6582 | 0.6548 to 0.6558 | 0.6563 to 0.6575 |
| Half Life | 1.053 to 1.055 | 1.057 to 1.059 | 1.054 to 1.056 |
| Tau | 1.519 to 1.522 | 1.525 to 1.527 | 1.521 to 1.524 |
| **Goodness of Fit** | | | |
| Degrees of Freedom | 1998 | 1998 | 1998 |
| R squared | 0.9999 | 0.9999 | 0.9999 |
| Sum of Squares | 29740 | 22115 | 25676 |
| Sy.x | 3.858 | 3.327 | 3.585 |
| **Constraints** | | | |
| K | K > 0 | K > 0 | K > 0 |
| | | | |
| **Number of points** | | | |
| # of X values | 2001 | 2001 | 2001 |
| # Y values analyzed | 2001 | 2001 | 2001 |

**Figure S42:** Tb(bispic-acetate) lifetimes in water

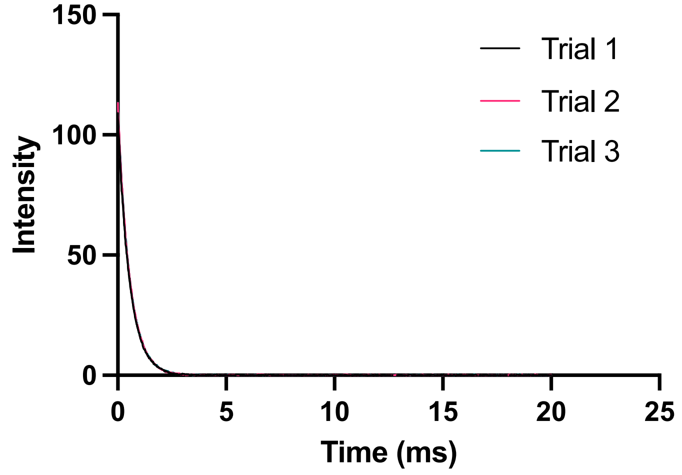


| | | | | |
|---|---|---|---|---|
| 1 | **One phase decay** | | | |
| 2 | **Best-fit values** | | | |
| 3 | Y0 | 40.89 | 41.48 | 253.1 |
| 4 | Plateau | -0.4404 | -0.4239 | 2.605 |
| 5 | K | 1.853 | 1.884 | 1.863 |
| 6 | Half Life | 0.3741 | 0.3680 | 0.3720 |
| 7 | Tau | 0.5397 | 0.5309 | 0.5367 |
| 8 | Span | 41.33 | 41.91 | 250.5 |
| 9 | **95% CI (profile likelihood)** | | | |
| 10 | Y0 | 40.84 to 40.9 | 41.43 to 41.5 | 252.8 to 253. |
| 11 | Plateau | -0.4450 to -0. | -0.4281 to -0. | 2.580 to 2.63 |
| 12 | K | 1.850 to 1.85 | 1.881 to 1.88 | 1.860 to 1.86 |
| 13 | Half Life | 0.3734 to 0.3 | 0.3674 to 0.3 | 0.3714 to 0.3 |
| 14 | Tau | 0.5387 to 0.5 | 0.5300 to 0.5 | 0.5358 to 0.5 |
| 15 | **Goodness of Fit** | | | |
| 16 | Degrees of Freedom | 1998 | 1998 | 1998 |
| 17 | R squared | 0.9996 | 0.9996 | 0.9996 |
| 18 | Sum of Squares | 18.92 | 16.46 | 574.4 |
| 19 | Sy.x | 0.09730 | 0.09078 | 0.5362 |
| 20 | **Constraints** | | | |
| 21 | K | K > 0 | K > 0 | K > 0 |
| 22 | | | | |
| 23 | **Number of points** | | | |
| 24 | # of X values | 2001 | 2001 | 2001 |
| 25 | # Y values analyzed | 2001 | 2001 | 2001 |

**Figure S43:** Eu(bispic-acetate) lifetimes in water

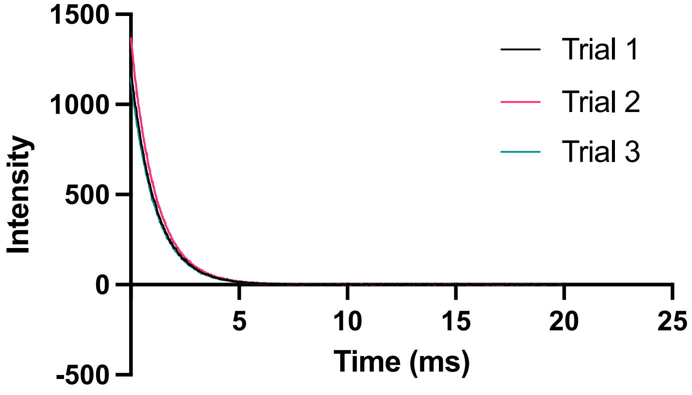


| One phase decay | | | |
|---|---|---|---|
| **Best-fit values** | | | |
| Y0 | 1200 | 1369 | 1149 |
| Plateau | 0.8052 | 0.8662 | 0.8137 |
| K | 0.8801 | 0.8819 | 0.8773 |
| Half Life | 0.7876 | 0.7860 | 0.7901 |
| Tau | 1.136 | 1.134 | 1.140 |
| Span | 1200 | 1368 | 1148 |
| **95% CI (profile likelihood)** | | | |
| Y0 | 1199 to 1201 | 1368 to 1369 | 1148 to 1150 |
| Plateau | 0.6680 to 0.9424 | 0.7478 to 0.9845 | 0.6976 to 0.9298 |
| K | 0.8789 to 0.8812 | 0.8811 to 0.8828 | 0.8763 to 0.8782 |
| Half Life | 0.7866 to 0.7886 | 0.7852 to 0.7867 | 0.7892 to 0.7910 |
| Tau | 1.135 to 1.138 | 1.133 to 1.135 | 1.139 to 1.141 |
| **Goodness of Fit** | | | |
| Degrees of Freedom | 1998 | 1998 | 1998 |
| R squared | 0.9998 | 0.9999 | 0.9998 |
| Sum of Squares | 15116 | 11252 | 10816 |
| Sy.x | 2.751 | 2.373 | 2.327 |
| **Constraints** | | | |
| K | K > 0 | K > 0 | K > 0 |
| | | | |
| **Number of points** | | | |
| # of X values | 2001 | 2001 | 2001 |
| # Y values analyzed | 2001 | 2001 | 2001 |

**Figure S44:** Tb(bispic) lifetimes in water

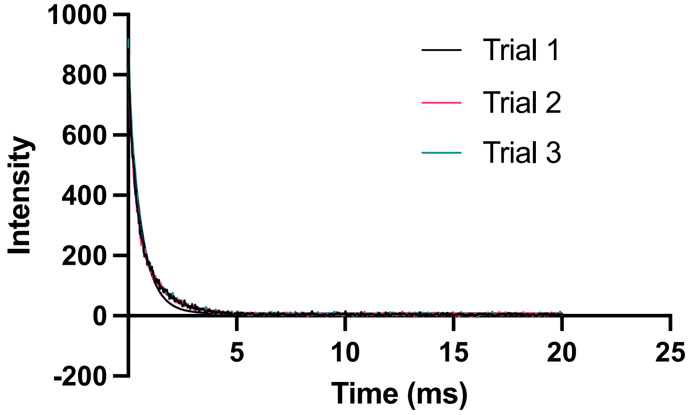


| One phase decay | | | |
|---|---|---|---|
| **Best-fit values** | | | |
| Y0 | 764.0 | 751.3 | 816.3 |
| Plateau | 7.271 | 7.591 | 8.357 |
| K | 1.614 | 1.621 | 1.674 |
| Half Life | 0.4295 | 0.4277 | 0.4141 |
| Tau | 0.6197 | 0.6170 | 0.5974 |
| Span | 756.7 | 743.7 | 807.9 |
| **95% CI (profile likelihood)** | | | |
| Y0 | 758.1 to 770.0 | 745.1 to 757.5 | 810.0 to 822.5 |
| Plateau | 6.736 to 7.806 | 7.035 to 8.147 | 7.806 to 8.908 |
| K | 1.594 to 1.634 | 1.600 to 1.642 | 1.654 to 1.694 |
| Half Life | 0.4243 to 0.434 | 0.4221 to 0.433 | 0.4091 to 0.419 |
| Tau | 0.6121 to 0.627 | 0.6090 to 0.625 | 0.5902 to 0.604 |
| **Goodness of Fit** | | | |
| Degrees of Freedom | 1998 | 1998 | 1998 |
| R squared | 0.9851 | 0.9833 | 0.9856 |
| Sum of Squares | 256043 | 276498 | 272716 |
| Sy.x | 11.32 | 11.76 | 11.68 |
| **Constraints** | | | |
| K | K > 0 | K > 0 | K > 0 |
| | | | |
| **Number of points** | | | |
| # of X values | 2001 | 2001 | 2001 |
| # Y values analyzed | 2001 | 2001 | 2001 |

**Figure S45:** Eu(bispic) lifetimes in water

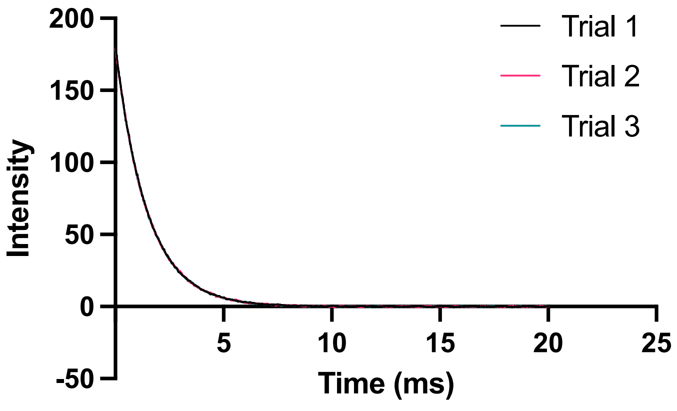


| One phase decay | | | |
|---|---|---|---|
| **Best-fit values** | | | |
| Y0 | 181.1 | 180.4 | 180.2 |
| Plateau | 0.01609 | 0.01822 | 0.01091 |
| K | 0.6826 | 0.6819 | 0.6823 |
| Half Life | 1.015 | 1.016 | 1.016 |
| Tau | 1.465 | 1.466 | 1.466 |
| Span | 181.1 | 180.4 | 180.2 |
| **95% CI (profile likelihood)** | | | |
| Y0 | 181.0 to 181.2 | 180.3 to 180.5 | 180.1 to 180.3 |
| Plateau | 3.561e-005 to 0. | 0.001676 to 0.03 | -0.005342 to 0.0 |
| K | 0.6820 to 0.6832 | 0.6813 to 0.6825 | 0.6817 to 0.6829 |
| Half Life | 1.015 to 1.016 | 1.016 to 1.017 | 1.015 to 1.017 |
| Tau | 1.464 to 1.466 | 1.465 to 1.468 | 1.464 to 1.467 |
| **Goodness of Fit** | | | |
| Degrees of Freedom | 1998 | 1998 | 1998 |
| R squared | 0.9999 | 0.9999 | 0.9999 |
| Sum of Squares | 189.3 | 201.1 | 193.9 |
| Sy.x | 0.3078 | 0.3173 | 0.3116 |
| **Constraints** | | | |
| K | K > 0 | K > 0 | K > 0 |
| | | | |
| **Number of points** | | | |
| # of X values | 2001 | 2001 | 2001 |
| # Y values analyzed | 2001 | 2001 | 2001 |

**Figure S46:** Tb(bispic-amide) lifetimes in water

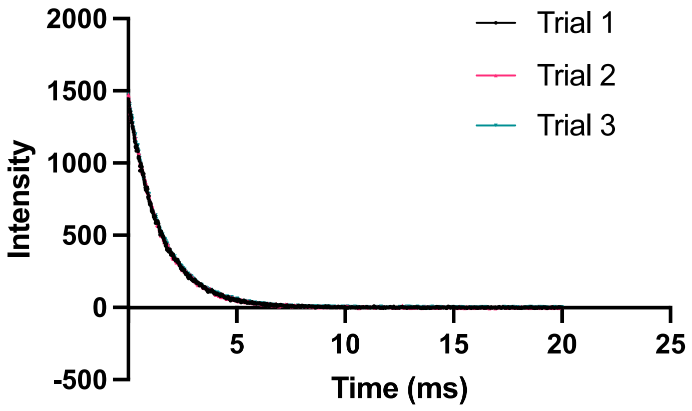


| One phase decay | | | |
|---|---|---|---|
| **Best-fit values** | | | |
| Y0 | 1435 | 1457 | 1464 |
| Plateau | 0.05970 | 0.6404 | 1.042 |
| K | 0.6750 | 0.6761 | 0.6798 |
| Half Life | 1.027 | 1.025 | 1.020 |
| Tau | 1.481 | 1.479 | 1.471 |
| Span | 1435 | 1456 | 1463 |
| **95% CI (profile likelihood)** | | | |
| Y0 | 1434 to 1437 | 1455 to 1459 | 1462 to 1466 |
| Plateau | -0.2507 to 0.37 | 0.3455 to 0.93 | 0.7519 to 1.33 |
| K | 0.6736 to 0.67 | 0.6748 to 0.67 | 0.6785 to 0.68 |
| Half Life | 1.025 to 1.029 | 1.023 to 1.027 | 1.018 to 1.022 |
| Tau | 1.478 to 1.484 | 1.476 to 1.482 | 1.468 to 1.474 |
| **Goodness of Fit** | | | |
| Degrees of Freedom | 1998 | 1998 | 1998 |
| R squared | 0.9995 | 0.9995 | 0.9995 |
| Sum of Squares | 70366 | 63551 | 61592 |
| Sy.x | 5.934 | 5.640 | 5.552 |
| **Constraints** | | | |
| K | K > 0 | K > 0 | K > 0 |
| | | | |
| **Number of points** | | | |
| # of X values | 2001 | 2001 | 2001 |
| # Y values analyzed | 2001 | 2001 | 2001 |

**Figure S47:** Tb(bispic-lysine) lifetimes in water

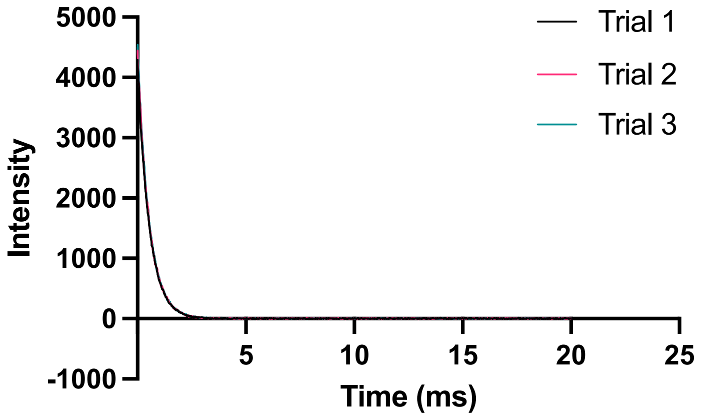


| One phase decay | | | |
|---|---|---|---|
| **Best-fit values** | | | |
| Y0 | 4278 | 4433 | 4540 |
| Plateau | 0.1996 | 1.121 | 1.702 |
| K | 1.883 | 1.903 | 1.914 |
| Half Life | 0.3680 | 0.3643 | 0.3622 |
| Tau | 0.5309 | 0.5256 | 0.5225 |
| Span | 4278 | 4432 | 4539 |
| **95% CI (profile likelihood)** | | | |
| Y0 | 4275 to 4282 | 4429 to 4438 | 4537 to 4544 |
| Plateau | -0.1296 to 0.52 | 0.7372 to 1.50 | 1.374 to 2.030 |
| K | 1.881 to 1.886 | 1.900 to 1.905 | 1.912 to 1.916 |
| Half Life | 0.3675 to 0.36 | 0.3638 to 0.36 | 0.3617 to 0.36 |
| Tau | 0.5303 to 0.53 | 0.5248 to 0.52 | 0.5219 to 0.52 |
| **Goodness of Fit** | | | |
| Degrees of Freedom | 1998 | 1998 | 1998 |
| R squared | 0.9998 | 0.9997 | 0.9998 |
| Sum of Squares | 100717 | 137131 | 100005 |
| Sy.x | 7.100 | 8.285 | 7.075 |
| **Constraints** | | | |
| K | K > 0 | K > 0 | K > 0 |
| | | | |
| **Number of points** | | | |
| # of X values | 2001 | 2001 | 2001 |
| # Y values analyzed | 2001 | 2001 | 2001 |

**Figure S48:** Eu(bispic-lysine) lifetimes in water

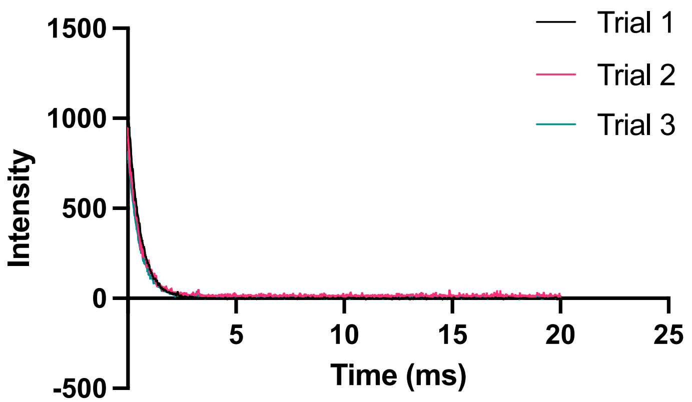


| One phase decay | | | |
|---|---|---|---|
| **Best-fit values** | | | |
| Y0 | 1059 | 886.1 | 815.8 |
| Plateau | -0.1676 | 14.23 | 3.718 |
| K | 1.825 | 1.786 | 1.821 |
| Half Life | 0.3799 | 0.3881 | 0.3806 |
| Tau | 0.5480 | 0.5599 | 0.5491 |
| Span | 1059 | 871.9 | 812.1 |
| **95% CI (profile likelihood)** | | | |
| Y0 | 1057 to 1061 | 881.5 to 890.7 | 813.1 to 818.5 |
| Plateau | -0.3611 to 0.02 | 13.81 to 14.64 | 3.479 to 3.957 |
| K | 1.819 to 1.830 | 1.772 to 1.800 | 1.812 to 1.830 |
| Half Life | 0.3787 to 0.38 | 0.3851 to 0.39 | 0.3787 to 0.38 |
| Tau | 0.5464 to 0.54 | 0.5556 to 0.56 | 0.5464 to 0.55 |
| **Goodness of Fit** | | | |
| Degrees of Freedom | 1998 | 1998 | 1998 |
| R squared | 0.9988 | 0.9923 | 0.9970 |
| Sum of Squares | 34677 | 158339 | 52960 |
| Sy.x | 4.166 | 8.902 | 5.148 |
| **Constraints** | | | |
| K | K > 0 | K > 0 | K > 0 |
| | | | |
| **Number of points** | | | |
| # of X values | 2001 | 2001 | 2001 |
| # Y values analyzed | 2001 | 2001 | 2001 |

**Figure S49:** Eu(bispic-Ser) lifetimes in water

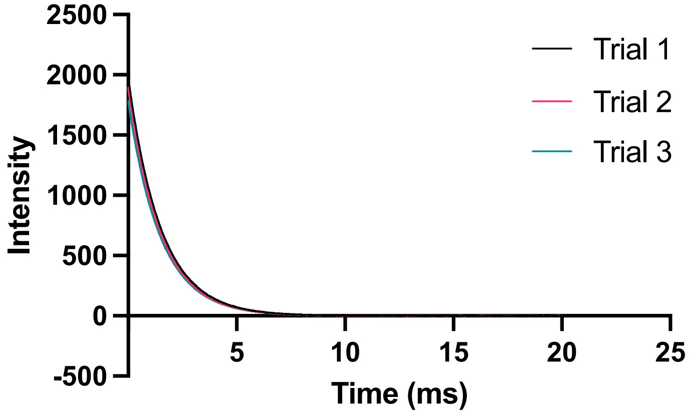


| One phase decay | | | |
|---|---|---|---|
| **Best-fit values** | | | |
| Y0 | 1973 | 1889 | 1781 |
| Plateau | -0.04575 | -0.04090 | -0.02450 |
| K | 0.6645 | 0.6753 | 0.6707 |
| Half Life | 1.043 | 1.026 | 1.033 |
| Tau | 1.505 | 1.481 | 1.491 |
| Span | 1973 | 1889 | 1781 |
| **95% CI (profile likelihood)** | | | |
| Y0 | 1973 to 1974 | 1888 to 1889 | 1781 to 1781 |
| Plateau | -0.09635 to 0.0 | -0.08464 to 0.0 | -0.07373 to 0.0 |
| K | 0.6643 to 0.66 | 0.6752 to 0.67 | 0.6705 to 0.67 |
| Half Life | 1.043 to 1.043 | 1.026 to 1.027 | 1.033 to 1.034 |
| Tau | 1.505 to 1.505 | 1.480 to 1.481 | 1.491 to 1.491 |
| **Goodness of Fit** | | | |
| Degrees of Freedom | 1998 | 1998 | 1998 |
| R squared | 1.000 | 1.000 | 1.000 |
| Sum of Squares | 1860 | 1399 | 1768 |
| Sy.x | 0.9649 | 0.8369 | 0.9407 |
| **Constraints** | | | |
| K | K > 0 | K > 0 | K > 0 |
| | | | |
| **Number of points** | | | |
| # of X values | 2001 | 2001 | 2001 |
| # Y values analyzed | 2001 | 2001 | 2001 |

**Figure S50:** Tb(bispic-Ser) lifetimes in water

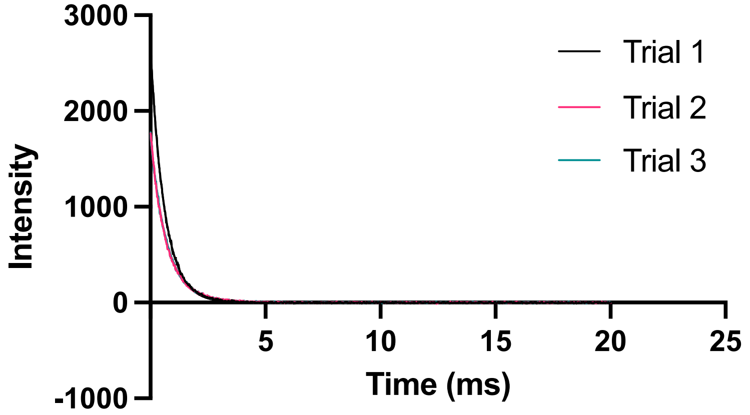


| One phase decay | | | |
|---|---|---|---|
| **Best-fit values** | | | |
| Y0 | 2665 | 1747 | 1741 |
| Plateau | -0.6038 | 2.156 | 1.547 |
| K | 1.631 | 1.447 | 1.424 |
| Half Life | 0.4249 | 0.4791 | 0.4866 |
| Tau | 0.6130 | 0.6912 | 0.7021 |
| Span | 2665 | 1745 | 1740 |
| **95% CI (profile likelihood)** | | | |
| Y0 | 2662 to 2668 | 1743 to 1751 | 1738 to 1745 |
| Plateau | -0.9126 to -0.2950 | 1.763 to 2.549 | 1.214 to 1.880 |
| K | 1.628 to 1.634 | 1.442 to 1.452 | 1.420 to 1.428 |
| Half Life | 0.4241 to 0.4257 | 0.4775 to 0.480 | 0.4853 to 0.488 |
| Tau | 0.6119 to 0.6141 | 0.6889 to 0.693 | 0.7001 to 0.704 |
| **Goodness of Fit** | | | |
| Degrees of Freedom | 1998 | 1998 | 1998 |
| R squared | 0.9996 | 0.9986 | 0.9990 |
| Sum of Squares | 86846 | 137761 | 98801 |
| Sy.x | 6.593 | 8.304 | 7.032 |
| **Constraints** | | | |
| K | K > 0 | K > 0 | K > 0 |
| | | | |
| **Number of points** | | | |
| # of X values | 2001 | 2001 | 2001 |
| # Y values analyzed | 2001 | 2001 | 2001 |

**Figure S51:** Eu(bispic-PSer) lifetimes in water

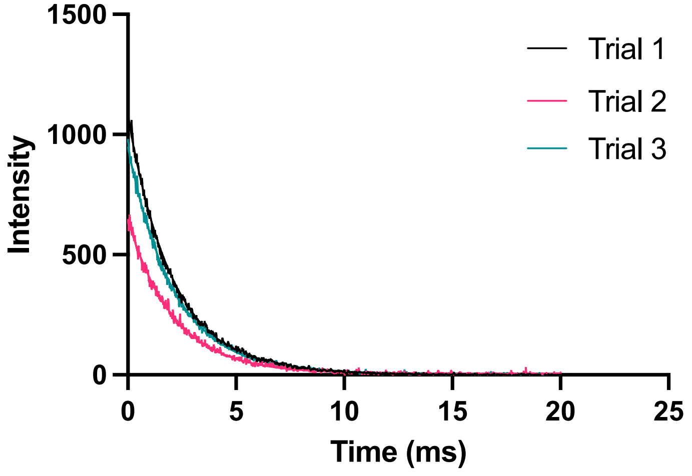


| One phase decay | | | |
|---|---|---|---|
| **Best-fit values** | | | |
| Y0 | 1078 | 643.9 | 947.3 |
| Plateau | 3.119 | 5.357 | 4.313 |
| K | 0.4809 | 0.4687 | 0.4691 |
| Half Life | 1.441 | 1.479 | 1.478 |
| Tau | 2.079 | 2.133 | 2.132 |
| Span | 1075 | 638.5 | 942.9 |
| **95% CI (profile likelihood)** | | | |
| Y0 | 1076 to 1080 | 641.7 to 646. | 945.3 to 949. |
| Plateau | 2.643 to 3.59 | 4.898 to 5.81 | 3.900 to 4.72 |
| K | 0.4792 to 0.4 | 0.4661 to 0.4 | 0.4675 to 0.4 |
| Half Life | 1.436 to 1.44 | 1.471 to 1.48 | 1.473 to 1.48 |
| Tau | 2.072 to 2.08 | 2.122 to 2.14 | 2.124 to 2.13 |
| **Goodness of Fit** | | | |
| Degrees of Freedom | 1998 | 1998 | 1998 |
| R squared | 0.9986 | 0.9964 | 0.9987 |
| Sum of Squares | 136384 | 124918 | 100841 |
| Sy.x | 8.262 | 7.907 | 7.104 |
| **Constraints** | | | |
| K | K > 0 | K > 0 | K > 0 |
| | | | |
| **Number of points** | | | |
| # of X values | 2001 | 2001 | 2001 |
| # Y values analyzed | 2001 | 2001 | 2001 |

**Figure S52:** Tb(bispic-PSer) lifetimes in water

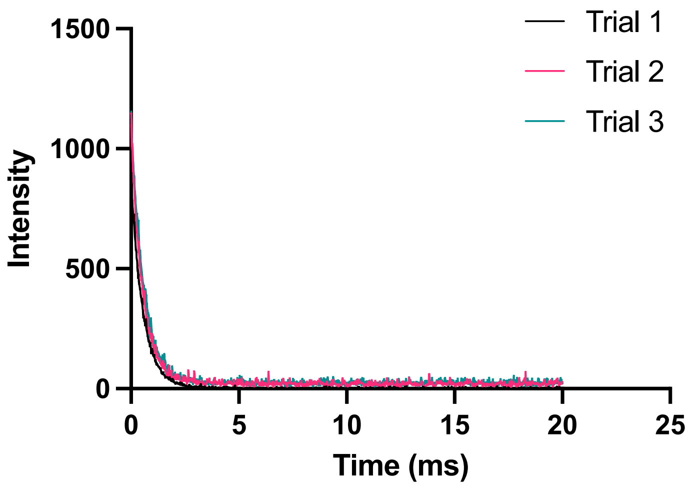


| One phase decay | | | |
|---|---|---|---|
| **Best-fit values** | | | |
| Y0 | 869.5 | 1100 | 1116 |
| Plateau | 1.397 | 21.97 | 25.57 |
| K | 1.774 | 1.870 | 1.727 |
| Half Life | 0.3908 | 0.3708 | 0.4015 |
| Tau | 0.5638 | 0.5349 | 0.5792 |
| Span | 868.1 | 1078 | 1091 |
| **95% CI (profile likelihood)** | | | |
| Y0 | 866.8 to 872.1 | 1094 to 1105 | 1110 to 1122 |
| Plateau | 1.159 to 1.635 | 21.46 to 22.48 | 25.04 to 26.11 |
| K | 1.766 to 1.782 | 1.855 to 1.885 | 1.713 to 1.740 |
| Half Life | 0.3891 to 0.392 | 0.3678 to 0.37 | 0.3983 to 0.40 |
| Tau | 0.5613 to 0.566 | 0.5306 to 0.53 | 0.5747 to 0.58 |
| **Goodness of Fit** | | | |
| Degrees of Freedom | 1998 | 1998 | 1998 |
| R squared | 0.9974 | 0.9920 | 0.9921 |
| Sum of Squares | 52193 | 240868 | 261942 |
| Sy.x | 5.111 | 10.98 | 11.45 |
| **Constraints** | | | |
| K | K > 0 | K > 0 | K > 0 |
| | | | |
| **Number of points** | | | |
| # of X values | 2001 | 2001 | 2001 |
| # Y values analyzed | 2001 | 2001 | 2001 |

**Figure S53:** Eu(bispic-GGG-Ser) lifetimes in water

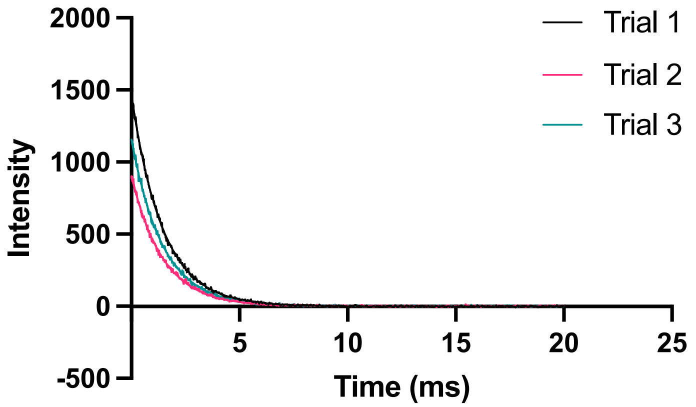


| One phase decay | | | |
|---|---|---|---|
| **Best-fit values** | | | |
| Y0 | 1464 | 902.5 | 1157 |
| Plateau | 0.03700 | 1.299 | 1.255 |
| K | 0.6896 | 0.6881 | 0.6964 |
| Half Life | 1.005 | 1.007 | 0.9953 |
| Tau | 1.450 | 1.453 | 1.436 |
| Span | 1464 | 901.2 | 1155 |
| **95% CI (profile likelihood)** | | | |
| Y0 | 1462 to 1466 | 900.7 to 904.3 | 1155 to 1159 |
| Plateau | -0.2890 to 0.36 | 1.016 to 1.581 | 0.9289 to 1.58 |
| K | 0.6881 to 0.69 | 0.6860 to 0.690 | 0.6945 to 0.69 |
| Half Life | 1.003 to 1.007 | 1.004 to 1.010 | 0.9926 to 0.99 |
| Tau | 1.447 to 1.453 | 1.449 to 1.458 | 1.432 to 1.440 |
| **Goodness of Fit** | | | |
| Degrees of Freedom | 1998 | 1998 | 1998 |
| R squared | 0.9994 | 0.9988 | 0.9990 |
| Sum of Squares | 78367 | 58815 | 78786 |
| Sy.x | 6.263 | 5.426 | 6.280 |
| **Constraints** | | | |
| K | K > 0 | K > 0 | K > 0 |
| | | | |
| **Number of points** | | | |
| # of X values | 2001 | 2001 | 2001 |
| # Y values analyzed | 2001 | 2001 | 2001 |

**Figure S54:** Tb(bispic-GGG-Ser) lifetimes in water

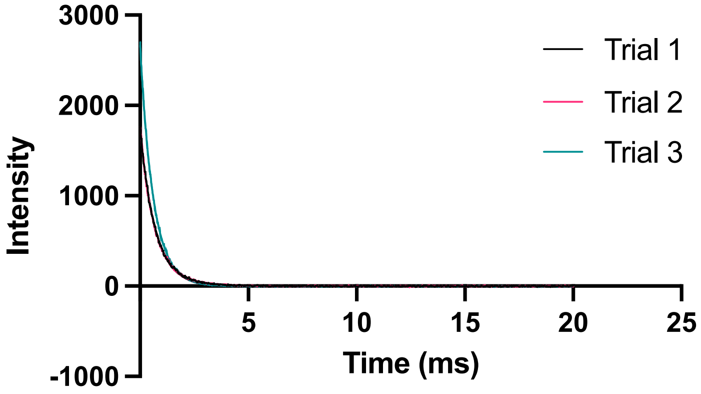


| One phase decay | | | |
|---|---|---|---|
| **Best-fit values** | | | |
| Y0 | 1741 | 1747 | 2665 |
| Plateau | 1.547 | 2.156 | -0.6038 |
| K | 1.424 | 1.447 | 1.631 |
| Half Life | 0.4866 | 0.4791 | 0.4249 |
| Tau | 0.7021 | 0.6912 | 0.6130 |
| Span | 1740 | 1745 | 2665 |
| **95% CI (profile likelihood)** | | | |
| Y0 | 1738 to 1745 | 1743 to 1751 | 2662 to 2668 |
| Plateau | 1.214 to 1.880 | 1.763 to 2.549 | -0.9126 to -0.2950 |
| K | 1.420 to 1.428 | 1.442 to 1.452 | 1.628 to 1.634 |
| Half Life | 0.4853 to 0.488 | 0.4775 to 0.480 | 0.4241 to 0.4257 |
| Tau | 0.7001 to 0.704 | 0.6889 to 0.693 | 0.6119 to 0.6141 |
| **Goodness of Fit** | | | |
| Degrees of Freedom | 1998 | 1998 | 1998 |
| R squared | 0.9990 | 0.9986 | 0.9996 |
| Sum of Squares | 98801 | 137761 | 86846 |
| Sy.x | 7.032 | 8.304 | 6.593 |
| **Constraints** | | | |
| K | K > 0 | K > 0 | K > 0 |
| | | | |
| **Number of points** | | | |
| # of X values | 2001 | 2001 | 2001 |
| # Y values analyzed | 2001 | 2001 | 2001 |

**Figure S55:** Eu(bispic-GGG-PSer) lifetimes in water

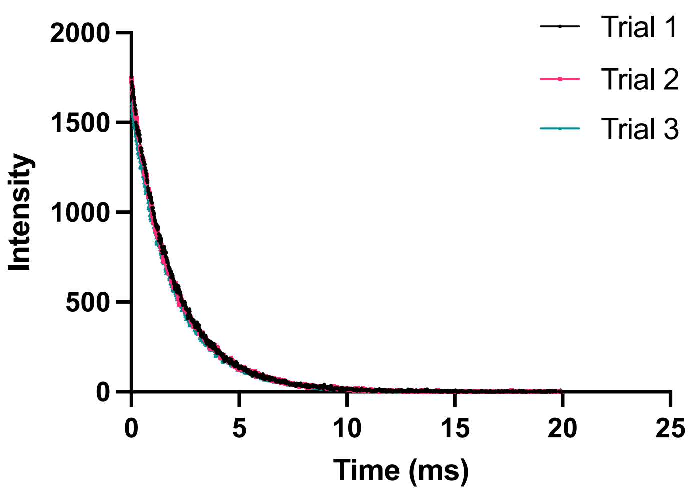


| One phase decay | | | |
|---|---|---|---|
| **Best-fit values** | | | |
| Y0 | 1712 | 1691 | 1576 |
| Plateau | 3.937 | 5.228 | 4.400 |
| K | 0.5137 | 0.5218 | 0.5124 |
| Half Life | 1.349 | 1.328 | 1.353 |
| Tau | 1.947 | 1.916 | 1.952 |
| Span | 1708 | 1686 | 1572 |
| **95% CI (profile likelihood)** | | | |
| Y0 | 1710 to 1715 | 1688 to 1695 | 1573 to 1579 |
| Plateau | 3.444 to 4.42 | 4.611 to 5.84 | 3.907 to 4.89 |
| K | 0.5125 to 0.5 | 0.5203 to 0.5 | 0.5111 to 0.5 |
| Half Life | 1.346 to 1.35 | 1.324 to 1.33 | 1.349 to 1.35 |
| Tau | 1.942 to 1.95 | 1.910 to 1.92 | 1.947 to 1.95 |
| **Goodness of Fit** | | | |
| Degrees of Freedom | 1998 | 1998 | 1998 |
| R squared | 0.9993 | 0.9989 | 0.9992 |
| Sum of Squares | 153434 | 240993 | 153396 |
| Sy.x | 8.763 | 10.98 | 8.762 |
| **Constraints** | | | |
| K | K > 0 | K > 0 | K > 0 |
| | | | |
| **Number of points** | | | |
| # of X values | 2001 | 2001 | 2001 |
| # Y values analyzed | 2001 | 2001 | 2001 |

**Figure S56:** Tb(bispic-GGG-PSer) lifetimes in water

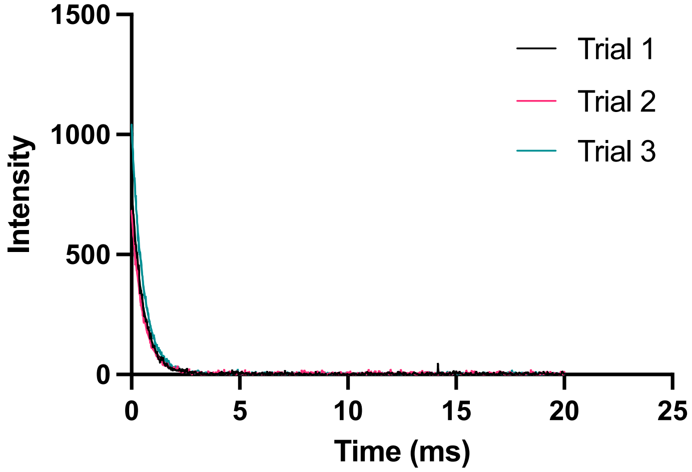


| One phase decay | | | |
|---|---|---|---|
| **Best-fit values** | | | |
| Y0 | 784.2 | 682.6 | 1042 |
| Plateau | 2.900 | 4.505 | 3.432 |
| K | 1.878 | 1.878 | 1.886 |
| Half Life | 0.3691 | 0.3691 | 0.3675 |
| Tau | 0.5324 | 0.5326 | 0.5302 |
| Span | 781.3 | 678.1 | 1039 |
| **95% CI (profile likelihood)** | | | |
| Y0 | 781.3 to 787.1 | 679.7 to 685.5 | 1039 to 1045 |
| Plateau | 2.645 to 3.155 | 4.251 to 4.759 | 3.195 to 3.670 |
| K | 1.868 to 1.888 | 1.866 to 1.890 | 1.879 to 1.893 |
| Half Life | 0.3670 to 0.371 | 0.3668 to 0.371 | 0.3661 to 0.368 |
| Tau | 0.5295 to 0.535 | 0.5292 to 0.535 | 0.5282 to 0.532 |
| **Goodness of Fit** | | | |
| Degrees of Freedom | 1998 | 1998 | 1998 |
| R squared | 0.9962 | 0.9950 | 0.9981 |
| Sum of Squares | 60546 | 59918 | 52540 |
| Sy.x | 5.505 | 5.476 | 5.128 |
| **Constraints** | | | |
| K | K > 0 | K > 0 | K > 0 |
| | | | |
| **Number of points** | | | |
| # of X values | 2001 | 2001 | 2001 |
| # Y values analyzed | 2001 | 2001 | 2001 |

**Figure S57:** Eu(bispic-PPP-Ser) lifetimes in water

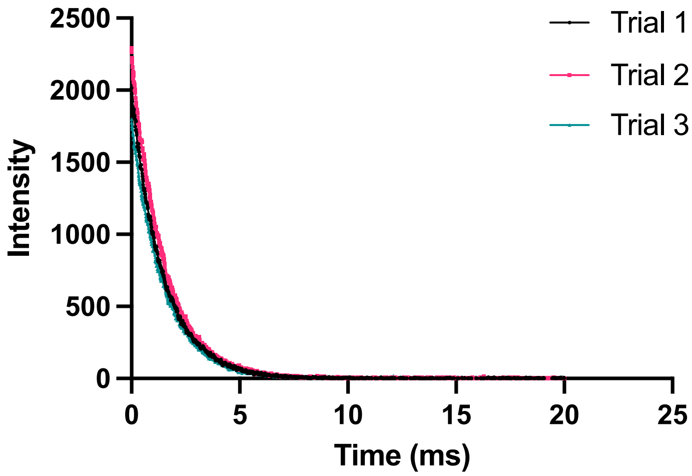


| | | Y | Y | Y |
|---|---|---|---|---|
| 1 | **One phase decay** | | | |
| 2 | **Best-fit values** | | | |
| 3 | Y0 | 1981 | 2232 | 1779 |
| 4 | Plateau | 0.1075 | 0.6400 | 1.323 |
| 5 | K | 0.6935 | 0.6874 | 0.6848 |
| 6 | Half Life | 0.9994 | 1.008 | 1.012 |
| 7 | Tau | 1.442 | 1.455 | 1.460 |
| 8 | Span | 1981 | 2232 | 1777 |
| 9 | **95% CI (profile likelihood)** | | | |
| 10 | Y0 | 1979 to 1983 | 2229 to 2235 | 1776 to 1781 |
| 11 | Plateau | -0.2793 to 0.4942 | 0.1609 to 1.119 | 0.9190 to 1.727 |
| 12 | K | 0.6922 to 0.6949 | 0.6860 to 0.6889 | 0.6833 to 0.6863 |
| 13 | Half Life | 0.9975 to 1.001 | 1.006 to 1.010 | 1.010 to 1.014 |
| 14 | Tau | 1.439 to 1.445 | 1.452 to 1.458 | 1.457 to 1.464 |
| 15 | **Goodness of Fit** | | | |
| 16 | Degrees of Freedom | 1998 | 1998 | 1998 |
| 17 | R squared | 0.9995 | 0.9995 | 0.9994 |
| 18 | Sum of Squares | 110531 | 169381 | 120293 |
| 19 | Sy.x | 7.438 | 9.207 | 7.759 |
| 20 | **Constraints** | | | |
| 21 | K | K > 0 | K > 0 | K > 0 |
| 22 | | | | |
| 23 | **Number of points** | | | |
| 24 | # of X values | 2001 | 2001 | 2001 |
| 25 | # Y values analyzed | 2001 | 2001 | 2001 |

**Figure S58:** Tb(bispic-PPP-Ser) lifetimes in water

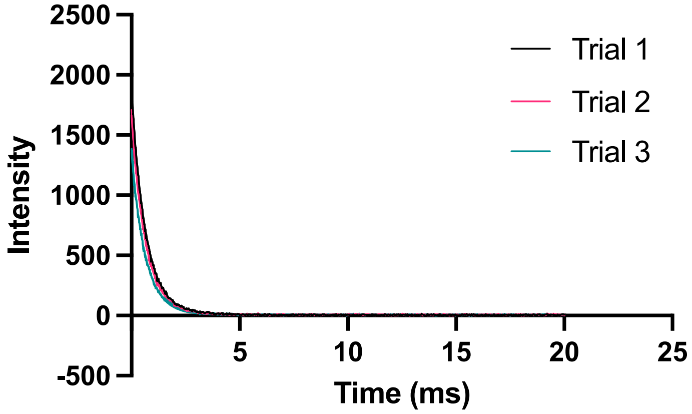


| One phase decay | | | |
|---|---|---|---|
| Best-fit values | | | |
| Y0 | 1861 | 1667 | 1356 |
| Plateau | 7.142 | 7.618 | 7.514 |
| K | 1.502 | 1.535 | 1.569 |
| Half Life | 0.4614 | 0.4516 | 0.4418 |
| Tau | 0.6657 | 0.6516 | 0.6373 |
| Span | 1854 | 1659 | 1348 |
| 95% CI (profile likelihood) | | | |
| Y0 | 1858 to 1864 | 1663 to 1670 | 1352 to 1359 |
| Plateau | 6.829 to 7.456 | 7.280 to 7.956 | 7.182 to 7.846 |
| K | 1.498 to 1.506 | 1.530 to 1.540 | 1.563 to 1.575 |
| Half Life | 0.4602 to 0.46 | 0.4502 to 0.45 | 0.4401 to 0.44 |
| Tau | 0.6640 to 0.66 | 0.6495 to 0.65 | 0.6349 to 0.63 |
| Goodness of Fit | | | |
| Degrees of Freedom | 1998 | 1998 | 1998 |
| R squared | 0.9992 | 0.9988 | 0.9982 |
| Sum of Squares | 88284 | 103091 | 99604 |
| Sy.x | 6.647 | 7.183 | 7.061 |
| Constraints | | | |
| K | K > 0 | K > 0 | K > 0 |
| | | | |
| Number of points | | | |
| # of X values | 2001 | 2001 | 2001 |
| # Y values analyzed | 2001 | 2001 | 2001 |

**Figure S59:** Eu(bispic-PPP-PSer) lifetimes in water

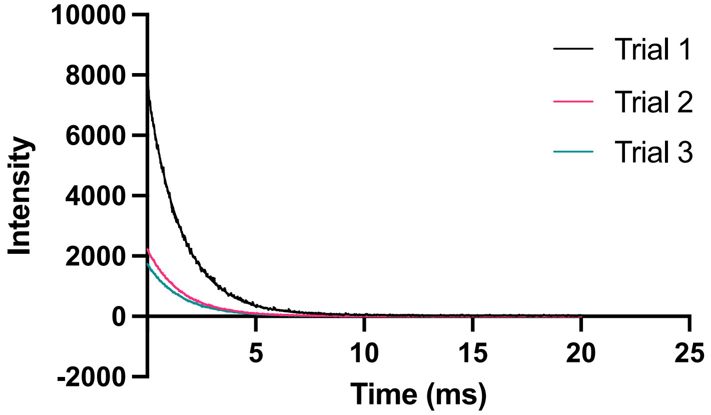


| One phase decay | | | |
|---|---|---|---|
| Best-fit values | | | |
| Y0 | 7597 | 2217 | 1726 |
| Plateau | 37.33 | 4.032 | 5.734 |
| K | 0.6324 | 0.6375 | 0.6316 |
| Half Life | 1.096 | 1.087 | 1.098 |
| Tau | 1.581 | 1.569 | 1.583 |
| Span | 7559 | 2213 | 1721 |
| 95% CI (profile likelihood) | | | |
| Y0 | 7585 to 7609 | 2214 to 2220 | 1724 to 1729 |
| Plateau | 35.33 to 39.32 | 3.567 to 4.496 | 5.344 to 6.123 |
| K | 0.6309 to 0.63 | 0.6362 to 0.63 | 0.6302 to 0.63 |
| Half Life | 1.093 to 1.099 | 1.085 to 1.089 | 1.095 to 1.100 |
| Tau | 1.577 to 1.585 | 1.566 to 1.572 | 1.580 to 1.587 |
| Goodness of Fit | | | |
| Degrees of Freedom | 1998 | 1998 | 1998 |
| R squared | 0.9993 | 0.9995 | 0.9995 |
| Sum of Squares | 2819953 | 153523 | 107443 |
| Sy.x | 37.57 | 8.766 | 7.333 |
| Constraints | | | |
| K | K > 0 | K > 0 | K > 0 |
| | | | |
| Number of points | | | |
| # of X values | 2001 | 2001 | 2001 |
| # Y values analyzed | 2001 | 2001 | 2001 |

**Figure S60:** Tb(bispic-PPP-PSer) lifetimes in water

## 4.7 $D_2O$ Lifetime Experiments

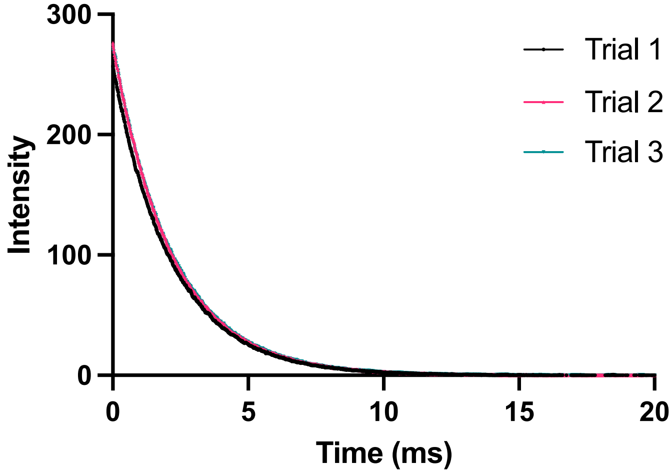


| One phase decay | | | |
|---|---|---|---|
| Best-fit values | | | |
| Y0 | 256.3 | 275.5 | 274.6 |
| Plateau | -0.07761 | -0.04504 | -0.02265 |
| K | 0.4601 | 0.4597 | 0.4594 |
| Half Life | 1.507 | 1.508 | 1.509 |
| Tau | 2.173 | 2.175 | 2.177 |
| Span | 256.4 | 275.5 | 274.7 |
| 95% CI (profile likelihood) | | | |
| Y0 | 256.2 to 256.4 | 275.4 to 275.5 | 274.5 to 274.7 |
| Plateau | -0.09791 to -0.0 | -0.06555 to -0.0 | -0.04396 to -0.0 |
| K | 0.4598 to 0.460 | 0.4594 to 0.459 | 0.4592 to 0.459 |
| Half Life | 1.506 to 1.507 | 1.507 to 1.509 | 1.508 to 1.510 |
| Tau | 2.172 to 2.175 | 2.174 to 2.177 | 2.175 to 2.178 |
| Goodness of Fit | | | |
| Degrees of Freedom | 1998 | 1998 | 1998 |
| R squared | 1.000 | 1.000 | 1.000 |
| Sum of Squares | 242.5 | 247.5 | 267.0 |
| Sy.x | 0.3484 | 0.3519 | 0.3655 |
| Constraints | | | |
| K | K > 0 | K > 0 | K > 0 |
| | | | |
| Number of points | | | |
| # of X values | 2001 | 2001 | 2001 |
| # Y values analyzed | 2001 | 2001 | 2001 |

**Figure S61:** Tb(trispic) lifetimes in $D_2O$

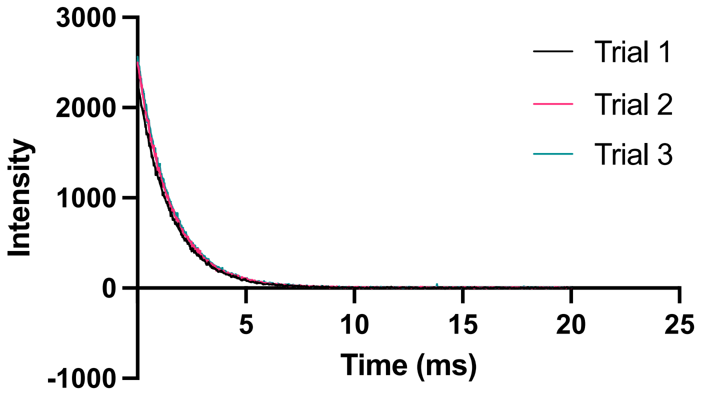


| | | | |
|---|---|---|---|
| **One phase decay** | | | |
| **Best-fit values** | | | |
| Y0 | 2291 | 2509 | 2566 |
| Plateau | 2.086 | 4.264 | 4.412 |
| K | 0.6601 | 0.6618 | 0.6553 |
| Half Life | 1.050 | 1.047 | 1.058 |
| Tau | 1.515 | 1.511 | 1.526 |
| Span | 2289 | 2505 | 2562 |
| **95% CI (profile likelihood)** | | | |
| Y0 | 2288 to 2294 | 2506 to 2512 | 2563 to 2570 |
| Plateau | 1.560 to 2.611 | 3.718 to 4.810 | 3.850 to 4.974 |
| K | 0.6586 to 0.66 | 0.6604 to 0.66 | 0.6540 to 0.65 |
| Half Life | 1.048 to 1.052 | 1.045 to 1.050 | 1.056 to 1.060 |
| Tau | 1.512 to 1.518 | 1.508 to 1.514 | 1.523 to 1.529 |
| **Goodness of Fit** | | | |
| Degrees of Freedom | 1998 | 1998 | 1998 |
| R squared | 0.9994 | 0.9995 | 0.9995 |
| Sum of Squares | 200434 | 216463 | 228424 |
| Sy.x | 10.02 | 10.41 | 10.69 |
| **Constraints** | | | |
| K | K > 0 | K > 0 | K > 0 |
| | | | |
| **Number of points** | | | |
| # of X values | 2001 | 2001 | 2001 |
| # Y values analyzed | 2001 | 2001 | 2001 |

**Figure S62:** Eu(trispic) lifetimes in $D_2O$

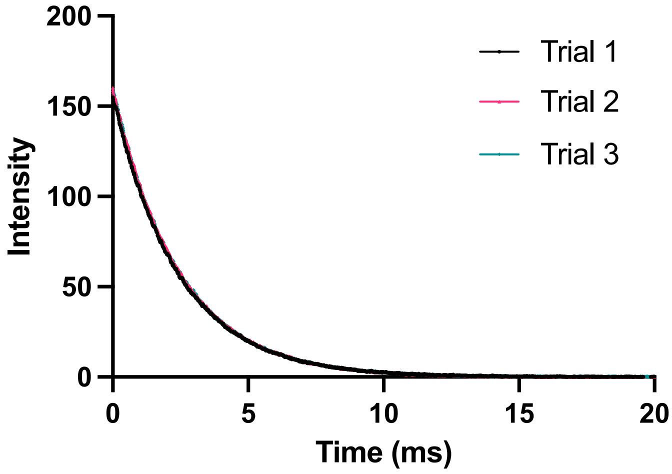


| | | | |
|---|---|---|---|
| **One phase decay** | | | |
| **Best-fit values** | | | |
| Y0 | 155.6 | 159.9 | 159.9 |
| Plateau | -0.01343 | -0.04944 | 0.003465 |
| K | 0.4121 | 0.4108 | 0.4139 |
| Half Life | 1.682 | 1.687 | 1.674 |
| Tau | 2.426 | 2.434 | 2.416 |
| Span | 155.6 | 160.0 | 159.9 |
| **95% CI (profile likelihood)** | | | |
| Y0 | 155.5 to 155.7 | 159.8 to 160.0 | 159.9 to 160.0 |
| Plateau | -0.03234 to 0.0 | -0.06864 to -0.0 | -0.01570 to 0.02 |
| K | 0.4118 to 0.412 | 0.4105 to 0.411 | 0.4136 to 0.414 |
| Half Life | 1.680 to 1.683 | 1.686 to 1.689 | 1.673 to 1.676 |
| Tau | 2.424 to 2.428 | 2.432 to 2.436 | 2.414 to 2.418 |
| **Goodness of Fit** | | | |
| Degrees of Freedom | 1998 | 1998 | 1998 |
| R squared | 0.9999 | 0.9999 | 0.9999 |
| Sum of Squares | 192.1 | 197.7 | 198.2 |
| Sy.x | 0.3101 | 0.3146 | 0.3149 |
| **Constraints** | | | |
| K | K > 0 | K > 0 | K > 0 |
| | | | |
| **Number of points** | | | |
| # of X values | 2001 | 2001 | 2001 |
| # Y values analyzed | 2001 | 2001 | 2001 |

**Figure S63:** Tb(bispic-acetate) lifetimes in $D_2O$

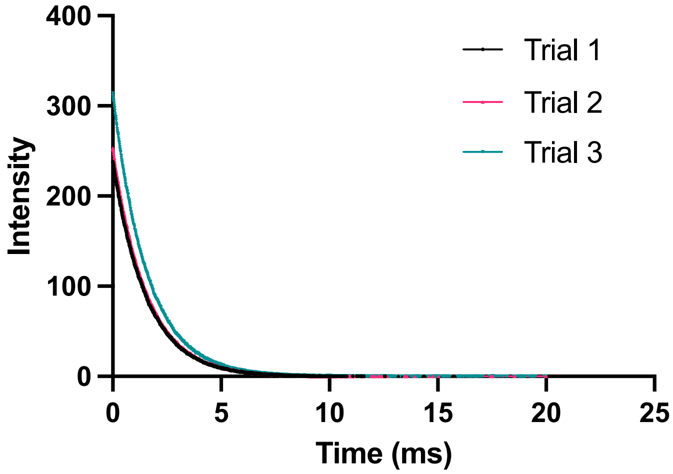


| | | | |
|---|---|---|---|
| **One phase decay** | | | |
| **Best-fit values** | | | |
| Y0 | 238.8 | 252.7 | 313.6 |
| Plateau | -0.3412 | -0.2848 | -0.1054 |
| K | 0.6420 | 0.6414 | 0.6422 |
| Half Life | 1.080 | 1.081 | 1.079 |
| Tau | 1.558 | 1.559 | 1.557 |
| Span | 239.1 | 253.0 | 313.7 |
| **95% CI (profile likelihood)** | | | |
| Y0 | 238.7 to 238.9 | 252.6 to 252.8 | 313.5 to 313.7 |
| Plateau | -0.3567 to -0.32 | -0.3018 to -0.26 | -0.1253 to -0.08 |
| K | 0.6417 to 0.642 | 0.6410 to 0.641 | 0.6418 to 0.642 |
| Half Life | 1.079 to 1.080 | 1.080 to 1.081 | 1.079 to 1.080 |
| Tau | 1.557 to 1.558 | 1.558 to 1.560 | 1.556 to 1.558 |
| **Goodness of Fit** | | | |
| Degrees of Freedom | 1998 | 1998 | 1998 |
| R squared | 1.000 | 1.000 | 1.000 |
| Sum of Squares | 171.1 | 206.9 | 283.8 |
| Sy.x | 0.2927 | 0.3218 | 0.3769 |
| **Constraints** | | | |
| K | K > 0 | K > 0 | K > 0 |
| | | | |
| **Number of points** | | | |
| # of X values | 2001 | 2001 | 2001 |
| # Y values analyzed | 2001 | 2001 | 2001 |

**Figure S64:** Eu(bispic-acetate) lifetimes in $D_2O$

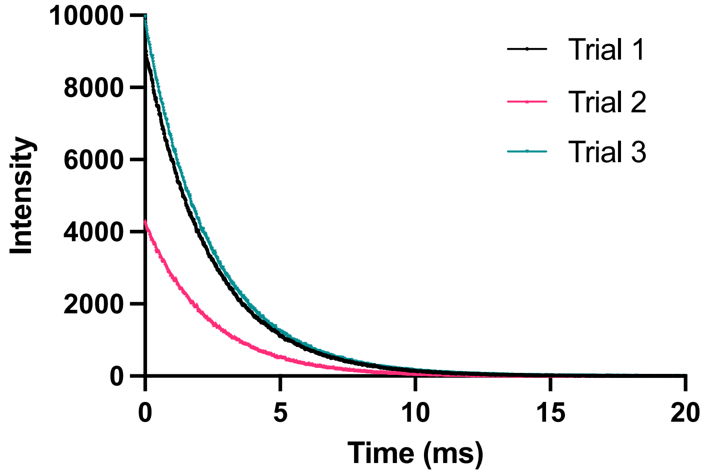


| One phase decay | | | |
|---|---|---|---|
| Best-fit values | | | |
| Y0 | 9057 | 4252 | 9806 |
| Plateau | 1.522 | -0.6286 | 2.825 |
| K | 0.4163 | 0.4160 | 0.4147 |
| Half Life | 1.665 | 1.666 | 1.671 |
| Tau | 2.402 | 2.404 | 2.411 |
| Span | 9055 | 4253 | 9803 |
| 95% CI (profile likelihood) | | | |
| Y0 | 9052 to 9061 | 4249 to 4255 | 9801 to 9811 |
| Plateau | 0.4415 to 2.60 | -1.394 to 0.136 | 1.654 to 3.995 |
| K | 0.4160 to 0.41 | 0.4154 to 0.41 | 0.4144 to 0.41 |
| Half Life | 1.664 to 1.666 | 1.664 to 1.668 | 1.670 to 1.673 |
| Tau | 2.400 to 2.404 | 2.401 to 2.407 | 2.409 to 2.413 |
| Goodness of Fit | | | |
| Degrees of Freedom | 1998 | 1998 | 1998 |
| R squared | 0.9999 | 0.9998 | 0.9999 |
| Sum of Squares | 632252 | 317403 | 739619 |
| Sy.x | 17.79 | 12.60 | 19.24 |
| Constraints | | | |
| K | K > 0 | K > 0 | K > 0 |
| | | | |
| Number of points | | | |
| # of X values | 2001 | 2001 | 2001 |
| # Y values analyzed | 2001 | 2001 | 2001 |

**Figure S65:** Tb(bispic) lifetimes in $D_2O$

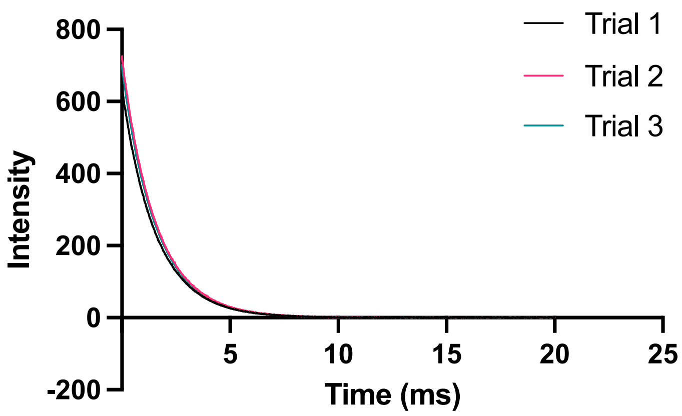


| One phase decay | | | |
|---|---|---|---|
| Best-fit values | | | |
| Y0 | 635.7 | 718.4 | 694.5 |
| Plateau | 0.2344 | 0.2748 | 0.2499 |
| K | 0.6407 | 0.6420 | 0.6404 |
| Half Life | 1.082 | 1.080 | 1.082 |
| Tau | 1.561 | 1.558 | 1.561 |
| Span | 635.5 | 718.2 | 694.3 |
| 95% CI (profile likelihood) | | | |
| Y0 | 635.4 to 636.0 | 718.1 to 718.8 | 694.2 to 694.9 |
| Plateau | 0.1827 to 0.28 | 0.2186 to 0.33 | 0.1917 to 0.30 |
| K | 0.6402 to 0.64 | 0.6415 to 0.64 | 0.6399 to 0.64 |
| Half Life | 1.081 to 1.083 | 1.079 to 1.081 | 1.081 to 1.083 |
| Tau | 1.560 to 1.562 | 1.557 to 1.559 | 1.560 to 1.563 |
| Goodness of Fit | | | |
| Degrees of Freedom | 1998 | 1998 | 1998 |
| R squared | 0.9999 | 0.9999 | 0.9999 |
| Sum of Squares | 1906 | 2257 | 2415 |
| Sy.x | 0.9768 | 1.063 | 1.099 |
| Constraints | | | |
| K | K > 0 | K > 0 | K > 0 |
| | | | |
| Number of points | | | |
| # of X values | 2001 | 2001 | 2001 |
| # Y values analyzed | 2001 | 2001 | 2001 |

**Figure S66:** Eu(bispic) lifetimes in $D_2O$

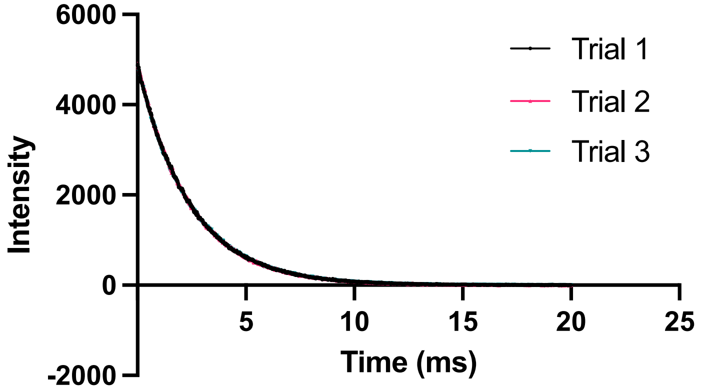


| One phase decay | | | |
|---|---|---|---|
| Best-fit values | | | |
| Y0 | 4875 | 4924 | 4837 |
| Plateau | 1.039 | 1.108 | 2.306 |
| K | 0.4127 | 0.4135 | 0.4144 |
| Half Life | 1.679 | 1.676 | 1.673 |
| Tau | 2.423 | 2.418 | 2.413 |
| Span | 4874 | 4923 | 4835 |
| 95% CI (profile likelihood) | | | |
| Y0 | 4872 to 4878 | 4921 to 4928 | 4834 to 4841 |
| Plateau | 0.3110 to 1.76 | 0.2492 to 1.96 | 1.444 to 3.169 |
| K | 0.4123 to 0.41 | 0.4130 to 0.41 | 0.4139 to 0.41 |
| Half Life | 1.678 to 1.681 | 1.674 to 1.678 | 1.671 to 1.675 |
| Tau | 2.420 to 2.425 | 2.415 to 2.421 | 2.410 to 2.416 |
| Goodness of Fit | | | |
| Degrees of Freedom | 1998 | 1998 | 1998 |
| R squared | 0.9999 | 0.9998 | 0.9998 |
| Sum of Squares | 285613 | 397118 | 401360 |
| Sy.x | 11.96 | 14.10 | 14.17 |
| Constraints | | | |
| K | K > 0 | K > 0 | K > 0 |
| | | | |
| Number of points | | | |
| # of X values | 2001 | 2001 | 2001 |
| # Y values analyzed | 2001 | 2001 | 2001 |

**Figure S67:** Tb(bispic-amide) lifetimes in $D_2O$

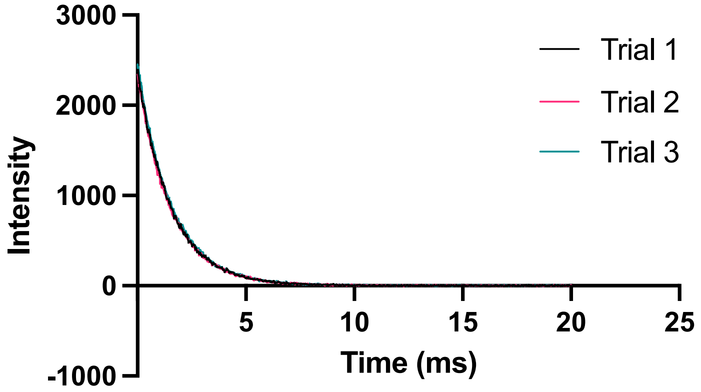


**Figure S68:** Eu(bispic-lysine) lifetimes in $D_2O$

| One phase decay | | | |
|---|---|---|---|
| Best-fit values | | | |
| Y0 | 2388 | 2341 | 2449 |
| Plateau | 1.687 | 3.021 | 3.774 |
| K | 0.6532 | 0.6560 | 0.6506 |
| Half Life | 1.061 | 1.057 | 1.065 |
| Tau | 1.531 | 1.524 | 1.537 |
| Span | 2386 | 2338 | 2445 |
| 95% CI (profile likelihood) | | | |
| Y0 | 2385 to 2391 | 2338 to 2344 | 2446 to 2452 |
| Plateau | 1.171 to 2.203 | 2.526 to 3.516 | 3.242 to 4.306 |
| K | 0.6518 to 0.65 | 0.6547 to 0.65 | 0.6493 to 0.65 |
| Half Life | 1.059 to 1.063 | 1.055 to 1.059 | 1.063 to 1.068 |
| Tau | 1.528 to 1.534 | 1.521 to 1.527 | 1.534 to 1.540 |
| Goodness of Fit | | | |
| Degrees of Freedom | 1998 | 1998 | 1998 |
| R squared | 0.9995 | 0.9995 | 0.9995 |
| Sum of Squares | 191761 | 176828 | 203725 |
| Sy.x | 9.797 | 9.408 | 10.10 |
| Constraints | | | |
| K | K > 0 | K > 0 | K > 0 |
| | | | |
| Number of points | | | |
| # of X values | 2001 | 2001 | 2001 |
| # Y values analyzed | 2001 | 2001 | 2001 |

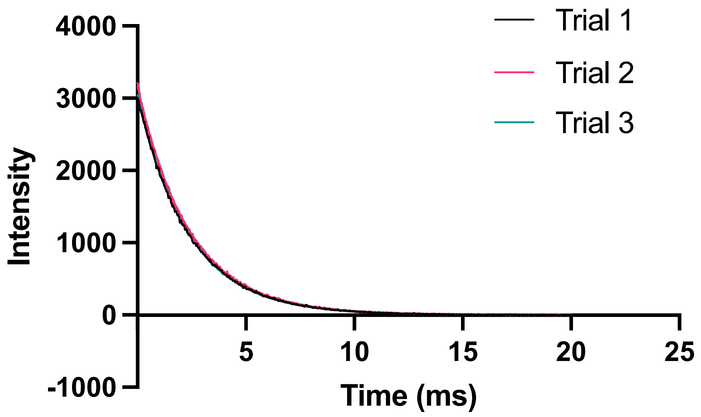


**Figure S69:** Tb(bispic-lysine) lifetimes in $D_2O$

| One phase decay | | | |
|---|---|---|---|
| Best-fit values | | | |
| Y0 | 3014 | 3163 | 3040 |
| Plateau | 2.021 | 3.582 | 3.454 |
| K | 0.4185 | 0.4171 | 0.4167 |
| Half Life | 1.656 | 1.662 | 1.663 |
| Tau | 2.389 | 2.397 | 2.400 |
| Span | 3012 | 3160 | 3036 |
| 95% CI (profile likelihood) | | | |
| Y0 | 3011 to 3016 | 3161 to 3166 | 3037 to 3042 |
| Plateau | 1.439 to 2.602 | 2.948 to 4.216 | 2.842 to 4.066 |
| K | 0.4179 to 0.41 | 0.4165 to 0.41 | 0.4161 to 0.41 |
| Half Life | 1.654 to 1.658 | 1.659 to 1.664 | 1.661 to 1.666 |
| Tau | 2.386 to 2.393 | 2.394 to 2.401 | 2.396 to 2.403 |
| Goodness of Fit | | | |
| Degrees of Freedom | 1998 | 1998 | 1998 |
| R squared | 0.9998 | 0.9998 | 0.9998 |
| Sum of Squares | 183583 | 217903 | 202860 |
| Sy.x | 9.586 | 10.44 | 10.08 |
| Constraints | | | |
| K | K > 0 | K > 0 | K > 0 |
| | | | |
| Number of points | | | |
| # of X values | 2001 | 2001 | 2001 |
| # Y values analyzed | 2001 | 2001 | 2001 |

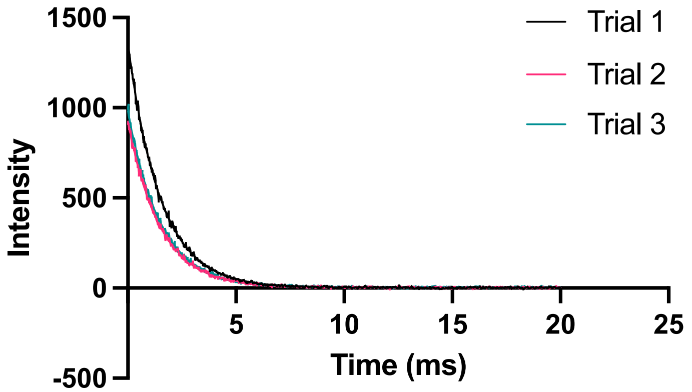


**Figure S70:** Eu(bispic-Ser) lifetimes in $D_2O$

| One phase decay | | | |
|---|---|---|---|
| Best-fit values | | | |
| Y0 | 1356 | 924.6 | 982.1 |
| Plateau | 1.720 | 2.399 | 3.593 |
| K | 0.6657 | 0.6678 | 0.6669 |
| Half Life | 1.041 | 1.038 | 1.039 |
| Tau | 1.502 | 1.497 | 1.500 |
| Span | 1355 | 922.2 | 978.5 |
| 95% CI (profile likelihood) | | | |
| Y0 | 1354 to 1359 | 922.7 to 926.5 | 980.1 to 984.2 |
| Plateau | 1.339 to 2.100 | 2.086 to 2.711 | 3.257 to 3.929 |
| K | 0.6639 to 0.66 | 0.6656 to 0.66 | 0.6647 to 0.66 |
| Half Life | 1.039 to 1.044 | 1.035 to 1.041 | 1.036 to 1.043 |
| Tau | 1.498 to 1.506 | 1.493 to 1.502 | 1.495 to 1.504 |
| Goodness of Fit | | | |
| Degrees of Freedom | 1998 | 1998 | 1998 |
| R squared | 0.9991 | 0.9987 | 0.9987 |
| Sum of Squares | 105400 | 71175 | 82062 |
| Sy.x | 7.263 | 5.969 | 6.409 |
| Constraints | | | |
| K | K > 0 | K > 0 | K > 0 |
| | | | |
| Number of points | | | |
| # of X values | 2001 | 2001 | 2001 |
| # Y values analyzed | 2001 | 2001 | 2001 |

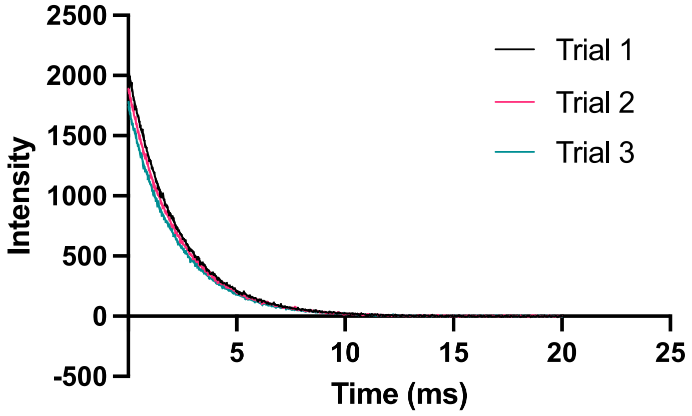


| | | | |
|---|---|---|---|
| **One phase decay** | | | |
| **Best-fit values** | | | |
| Y0 | 2032 | 1889 | 1744 |
| Plateau | 0.01136 | 0.8089 | 1.022 |
| K | 0.4516 | 0.4512 | 0.4515 |
| Half Life | 1.535 | 1.536 | 1.535 |
| Tau | 2.214 | 2.217 | 2.215 |
| Span | 2032 | 1888 | 1743 |
| **95% CI (profile likelihood)** | | | |
| Y0 | 2029 to 2034 | 1887 to 1891 | 1742 to 1746 |
| Plateau | -0.4829 to 0.5 | 0.3525 to 1.26 | 0.5255 to 1.51 |
| K | 0.4508 to 0.45 | 0.4503 to 0.45 | 0.4505 to 0.45 |
| Half Life | 1.532 to 1.538 | 1.534 to 1.539 | 1.532 to 1.539 |
| Tau | 2.210 to 2.218 | 2.213 to 2.221 | 2.210 to 2.220 |
| **Goodness of Fit** | | | |
| Degrees of Freedom | 1998 | 1998 | 1998 |
| R squared | 0.9996 | 0.9996 | 0.9995 |
| Sum of Squares | 141548 | 120526 | 142408 |
| Sy.x | 8.417 | 7.767 | 8.442 |
| **Constraints** | | | |
| K | K > 0 | K > 0 | K > 0 |
| | | | |
| **Number of points** | | | |
| # of X values | 2001 | 2001 | 2001 |
| # Y values analyzed | 2001 | 2001 | 2001 |

**Figure S71:** Tb(bispic-Ser) lifetimes in $D_2O$

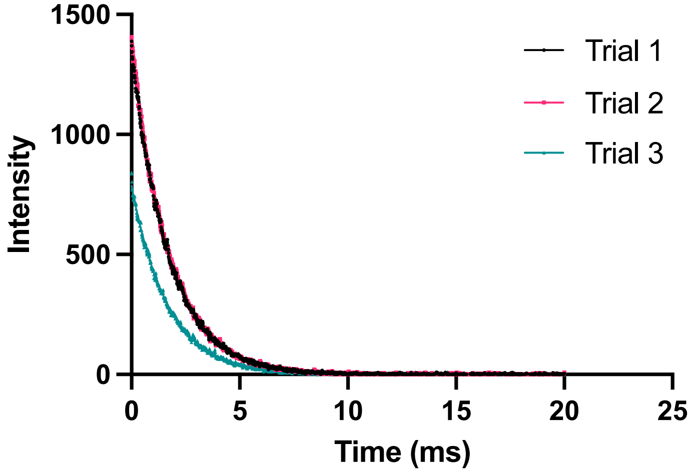


| | | | | |
|---|---|---|---|---|
| 1 | **One phase decay** | | | |
| 2 | **Best-fit values** | | | |
| 3 | Y0 | 1352 | 1385 | 786.5 |
| 4 | Plateau | -0.2574 | 0.1008 | 0.5511 |
| 5 | K | 0.5845 | 0.5901 | 0.5950 |
| 6 | Half Life | 1.186 | 1.175 | 1.165 |
| 7 | Tau | 1.711 | 1.695 | 1.681 |
| 8 | Span | 1352 | 1385 | 785.9 |
| 9 | **95% CI (profile likelihood)** | | | |
| 10 | Y0 | 1350 to 1354 | 1383 to 1387 | 784.9 to 788. |
| 11 | Plateau | -0.6186 to 0. | -0.2406 to 0. | 0.2703 to 0.8 |
| 12 | K | 0.5831 to 0.5 | 0.5888 to 0.5 | 0.5931 to 0.5 |
| 13 | Half Life | 1.183 to 1.18 | 1.172 to 1.17 | 1.161 to 1.16 |
| 14 | Tau | 1.707 to 1.71 | 1.691 to 1.69 | 1.675 to 1.68 |
| 15 | **Goodness of Fit** | | | |
| 16 | Degrees of Freedom | 1998 | 1998 | 1998 |
| 17 | R squared | 0.9993 | 0.9994 | 0.9988 |
| 18 | Sum of Squares | 89261 | 80039 | 54324 |
| 19 | Sy.x | 6.684 | 6.329 | 5.214 |
| 20 | **Constraints** | | | |
| 21 | K | K > 0 | K > 0 | K > 0 |
| 22 | | | | |
| 23 | **Number of points** | | | |
| 24 | # of X values | 2001 | 2001 | 2001 |
| 25 | # Y values analyzed | 2001 | 2001 | 2001 |

**Figure S72:** Eu(bispic-PSer) lifetimes in $D_2O$

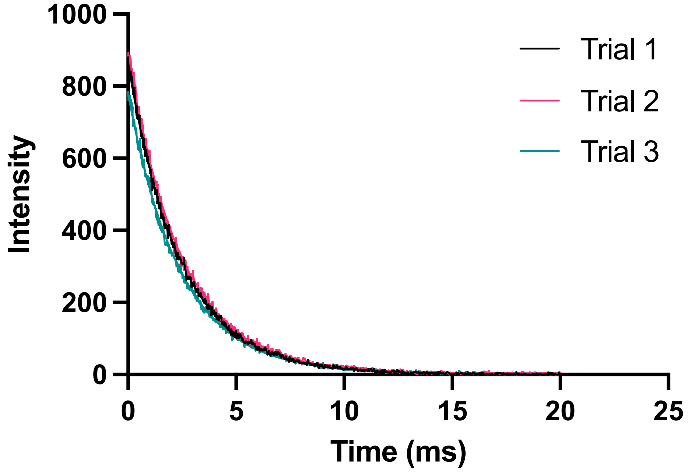


| | | | |
|---|---|---|---|
| **One phase decay** | | | |
| **Best-fit values** | | | |
| Y0 | 864.5 | 892.1 | 779.4 |
| Plateau | 2.055 | 3.549 | 3.895 |
| K | 0.4140 | 0.4089 | 0.4138 |
| Half Life | 1.674 | 1.695 | 1.675 |
| Tau | 2.416 | 2.446 | 2.416 |
| Span | 862.4 | 888.5 | 775.5 |
| **95% CI (profile likelihood)** | | | |
| Y0 | 863.0 to 866. | 890.3 to 893. | 777.8 to 780. |
| Plateau | 1.712 to 2.39 | 3.132 to 3.96 | 3.534 to 4.25 |
| K | 0.4128 to 0.4 | 0.4075 to 0.4 | 0.4125 to 0.4 |
| Half Life | 1.670 to 1.67 | 1.690 to 1.70 | 1.669 to 1.68 |
| Tau | 2.409 to 2.42 | 2.438 to 2.45 | 2.408 to 2.42 |
| **Goodness of Fit** | | | |
| Degrees of Freedom | 1998 | 1998 | 1998 |
| R squared | 0.9991 | 0.9987 | 0.9987 |
| Sum of Squares | 63223 | 92129 | 69922 |
| Sy.x | 5.625 | 6.790 | 5.916 |
| **Constraints** | | | |
| K | K > 0 | K > 0 | K > 0 |
| | | | |
| **Number of points** | | | |
| # of X values | 2001 | 2001 | 2001 |
| # Y values analyzed | 2001 | 2001 | 2001 |

**Figure S73:** Tb(bispic-PSer) lifetimes in $D_2O$

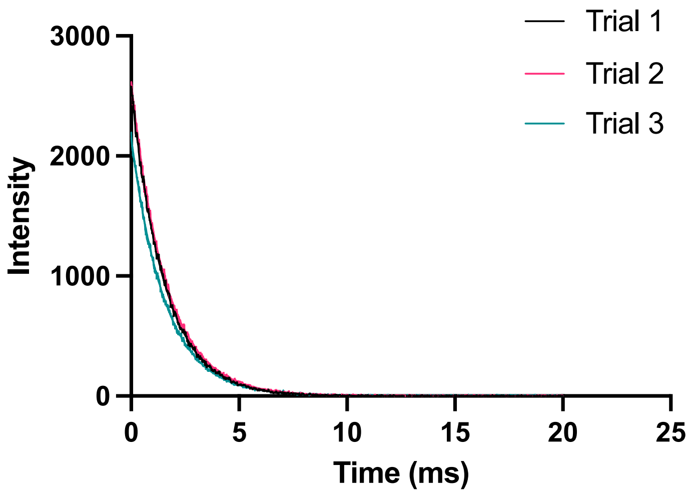


| One phase decay | | | |
|---|---|---|---|
| Best-fit values | | | |
| Y0 | 2571 | 2617 | 2151 |
| Plateau | -0.9470 | 1.846 | 3.742 |
| K | 0.6492 | 0.6321 | 0.6399 |
| Half Life | 1.068 | 1.097 | 1.083 |
| Tau | 1.540 | 1.582 | 1.563 |
| Span | 2572 | 2615 | 2147 |
| 95% CI (profile likelihood) | | | |
| Y0 | 2567 to 2574 | 2614 to 2620 | 2148 to 2154 |
| Plateau | -1.483 to -0.4 | 1.313 to 2.37 | 3.255 to 4.22 |
| K | 0.6479 to 0.6 | 0.6309 to 0.6 | 0.6386 to 0.6 |
| Half Life | 1.066 to 1.07 | 1.095 to 1.09 | 1.081 to 1.08 |
| Tau | 1.537 to 1.54 | 1.579 to 1.58 | 1.559 to 1.56 |
| Goodness of Fit | | | |
| Degrees of Freedom | 1998 | 1998 | 1998 |
| R squared | 0.9995 | 0.9996 | 0.9994 |
| Sum of Squares | 206956 | 201637 | 169462 |
| Sy.x | 10.18 | 10.05 | 9.210 |
| Constraints | | | |
| K | K > 0 | K > 0 | K > 0 |
| | | | |
| Number of points | | | |
| # of X values | 2001 | 2001 | 2001 |
| # Y values analyzed | 2001 | 2001 | 2001 |

**Figure S74:** Eu(bispic-GGG-Ser) lifetimes in $D_2O$

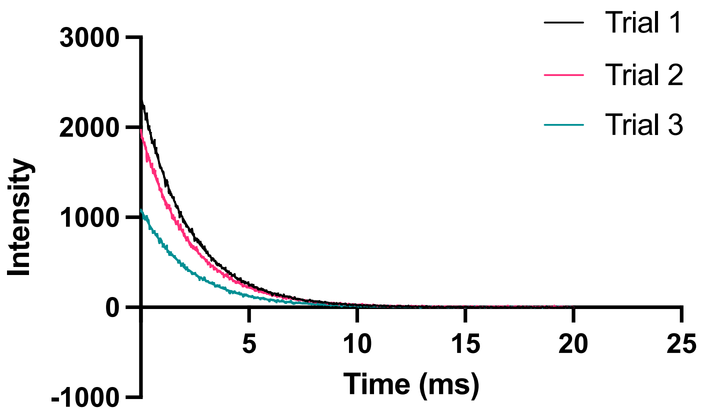


| One phase decay | | | |
|---|---|---|---|
| Best-fit values | | | |
| Y0 | 2351 | 1945 | 1088 |
| Plateau | -0.8500 | 3.985 | 2.523 |
| K | 0.4435 | 0.4386 | 0.4351 |
| Half Life | 1.563 | 1.580 | 1.593 |
| Tau | 2.255 | 2.280 | 2.298 |
| Span | 2352 | 1941 | 1086 |
| 95% CI (profile likelihood) | | | |
| Y0 | 2348 to 2355 | 1942 to 1948 | 1086 to 1091 |
| Plateau | -1.552 to -0.1 | 3.251 to 4.71 | 1.988 to 3.05 |
| K | 0.4425 to 0.4 | 0.4374 to 0.4 | 0.4335 to 0.4 |
| Half Life | 1.559 to 1.56 | 1.576 to 1.58 | 1.587 to 1.59 |
| Tau | 2.250 to 2.26 | 2.273 to 2.28 | 2.290 to 2.30 |
| Goodness of Fit | | | |
| Degrees of Freedom | 1998 | 1998 | 1998 |
| R squared | 0.9994 | 0.9991 | 0.9985 |
| Sum of Squares | 281368 | 304996 | 161042 |
| Sy.x | 11.87 | 12.36 | 8.978 |
| Constraints | | | |
| K | K > 0 | K > 0 | K > 0 |
| | | | |
| Number of points | | | |
| # of X values | 2001 | 2001 | 2001 |
| # Y values analyzed | 2001 | 2001 | 2001 |

**Figure S75:** Tb(bispic-GGG-Ser) lifetimes in $D_2O$

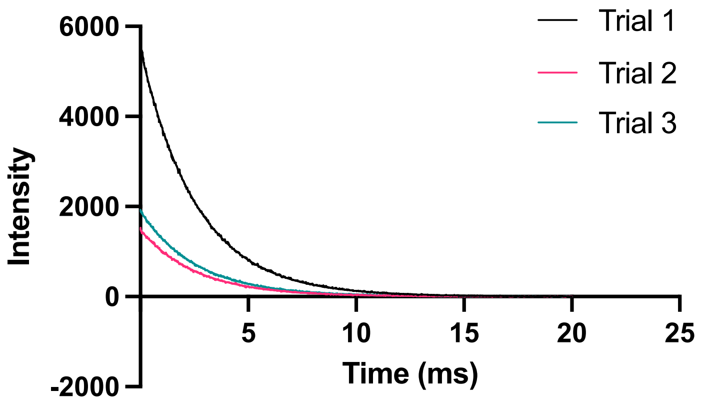


| One phase decay | | | |
|---|---|---|---|
| Best-fit values | | | |
| Y0 | 5500 | 1481 | 1896 |
| Plateau | 6.545 | 3.577 | 4.457 |
| K | 0.3834 | 0.3841 | 0.3840 |
| Half Life | 1.808 | 1.805 | 1.805 |
| Tau | 2.608 | 2.604 | 2.604 |
| Span | 5493 | 1478 | 1892 |
| 95% CI (profile likelihood) | | | |
| Y0 | 5495 to 5504 | 1479 to 1483 | 1894 to 1899 |
| Plateau | 5.445 to 7.645 | 3.070 to 4.084 | 3.893 to 5.022 |
| K | 0.3829 to 0.38 | 0.3832 to 0.38 | 0.3832 to 0.38 |
| Half Life | 1.805 to 1.810 | 1.801 to 1.809 | 1.802 to 1.809 |
| Tau | 2.605 to 2.612 | 2.598 to 2.610 | 2.599 to 2.610 |
| Goodness of Fit | | | |
| Degrees of Freedom | 1998 | 1998 | 1998 |
| R squared | 0.9998 | 0.9994 | 0.9995 |
| Sum of Squares | 605388 | 128921 | 159500 |
| Sy.x | 17.41 | 8.033 | 8.935 |
| Constraints | | | |
| K | K > 0 | K > 0 | K > 0 |
| | | | |
| Number of points | | | |
| # of X values | 2001 | 2001 | 2001 |
| # Y values analyzed | 2001 | 2001 | 2001 |

**Figure S76:** Tb(bispic-GGG-PSer) lifetimes in $D_2O$

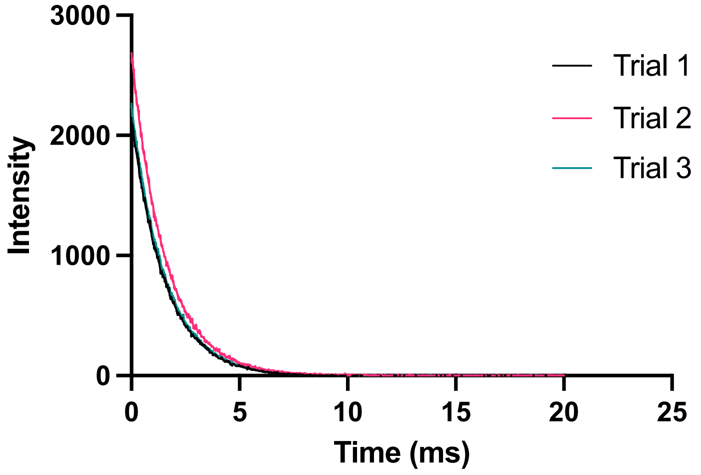


| | Y | Y | Y |
|---|---|---|---|
| Y0 | 2153 | 2686 | 2246 |
| Plateau | -6.654 | 0.2128 | -3.888 |
| K | 0.6473 | 0.6446 | 0.6451 |
| Half Life | 1.071 | 1.075 | 1.074 |
| Tau | 1.545 | 1.551 | 1.550 |
| Span | 2160 | 2685 | 2250 |
| **95% CI (profile likelihood)** | | | |
| Y0 | 2150 to 2156 | 2682 to 2689 | 2243 to 2249 |
| Plateau | -7.112 to -6.19 | -0.3247 to 0.75 | -4.337 to -3.43 |
| K | 0.6460 to 0.64 | 0.6434 to 0.64 | 0.6439 to 0.64 |
| Half Life | 1.069 to 1.073 | 1.073 to 1.077 | 1.072 to 1.076 |
| Tau | 1.542 to 1.548 | 1.549 to 1.554 | 1.547 to 1.553 |
| **Goodness of Fit** | | | |
| Degrees of Freedom | 1998 | 1998 | 1998 |
| R squared | 0.9995 | 0.9996 | 0.9996 |
| Sum of Squares | 150497 | 207044 | 144708 |
| Sy.x | 8.679 | 10.18 | 8.510 |
| **Constraints** | | | |
| K | K > 0 | K > 0 | K > 0 |
| | | | |
| **Number of points** | | | |
| # of X values | 2001 | 2001 | 2001 |
| # Y values analyzed | 2001 | 2001 | 2001 |

**Figure S77:** Eu(bispic-PPP_Ser) lifetimes in $D_2O$

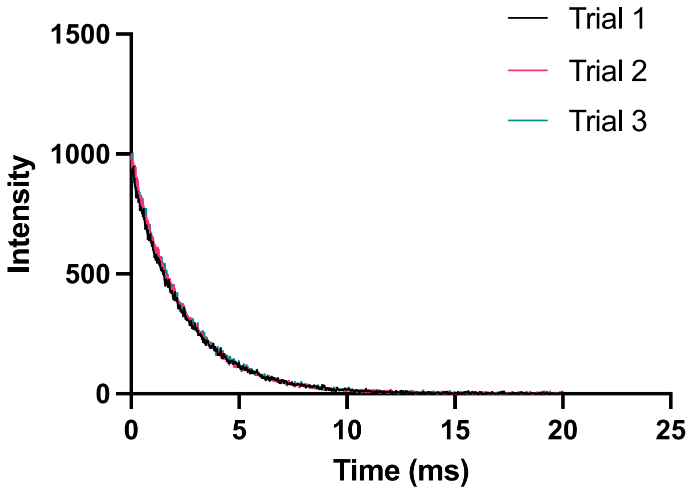


| | | | | |
|---|---|---|---|---|
| 1 | **One phase decay** | | | |
| 2 | **Best-fit values** | | | |
| 3 | Y0 | 936.4 | 985.4 | 984.7 |
| 4 | Plateau | 1.682 | 1.397 | 1.797 |
| 5 | K | 0.4223 | 0.4305 | 0.4246 |
| 6 | Half Life | 1.641 | 1.610 | 1.633 |
| 7 | Tau | 2.368 | 2.323 | 2.355 |
| 8 | Span | 934.7 | 984.0 | 982.9 |
| 9 | **95% CI (profile likelihood)** | | | |
| 10 | Y0 | 934.6 to 938.1 | 983.6 to 987.2 | 982.7 to 986.6 |
| 11 | Plateau | 1.269 to 2.095 | 0.9839 to 1.811 | 1.348 to 2.245 |
| 12 | K | 0.4210 to 0.4237 | 0.4292 to 0.4318 | 0.4232 to 0.4259 |
| 13 | Half Life | 1.636 to 1.647 | 1.605 to 1.615 | 1.627 to 1.638 |
| 14 | Tau | 2.360 to 2.375 | 2.316 to 2.330 | 2.348 to 2.363 |
| 15 | **Goodness of Fit** | | | |
| 16 | Degrees of Freedom | 1998 | 1998 | 1998 |
| 17 | R squared | 0.9988 | 0.9989 | 0.9987 |
| 18 | Sum of Squares | 93302 | 95507 | 110874 |
| 19 | Sy.x | 6.834 | 6.914 | 7.449 |
| 20 | **Constraints** | | | |
| 21 | K | K > 0 | K > 0 | K > 0 |
| 22 | | | | |
| 23 | **Number of points** | | | |
| 24 | # of X values | 2001 | 2001 | 2001 |
| 25 | # Y values analyzed | 2001 | 2001 | 2001 |

**Figure S78:** Tb(bispic-PPP_Ser) lifetimes in $D_2O$

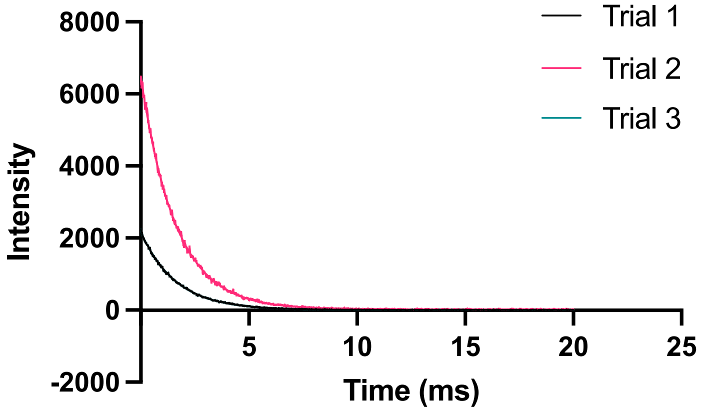


| | | | |
|---|---|---|---|
| **One phase decay** | | | |
| **Best-fit values** | | | |
| Y0 | 2170 | 6484 | 2170 |
| Plateau | 5.015 | 20.40 | 5.015 |
| K | 0.6198 | 0.6243 | 0.6198 |
| Half Life | 1.118 | 1.110 | 1.118 |
| Tau | 1.613 | 1.602 | 1.613 |
| Span | 2165 | 6464 | 2165 |
| **95% CI (profile likelihood)** | | | |
| Y0 | 2167 to 2173 | 6474 to 6494 | 2167 to 2173 |
| Plateau | 4.465 to 5.56 | 18.74 to 22.0 | 4.465 to 5.56 |
| K | 0.6183 to 0.6 | 0.6228 to 0.6 | 0.6183 to 0.6 |
| Half Life | 1.116 to 1.12 | 1.108 to 1.11 | 1.116 to 1.12 |
| Tau | 1.610 to 1.61 | 1.598 to 1.60 | 1.610 to 1.61 |
| **Goodness of Fit** | | | |
| Degrees of Freedom | 1998 | 1998 | 1998 |
| R squared | 0.9993 | 0.9993 | 0.9993 |
| Sum of Squares | 211766 | 1937886 | 211766 |
| Sy.x | 10.30 | 31.14 | 10.30 |
| **Constraints** | | | |
| K | K > 0 | K > 0 | K > 0 |
| | | | |
| **Number of points** | | | |
| # of X values | 2001 | 2001 | 2001 |
| # Y values analyzed | 2001 | 2001 | 2001 |

**Figure S79:** Eu(bispic-PPP-PSer) lifetimes in $D_2O$

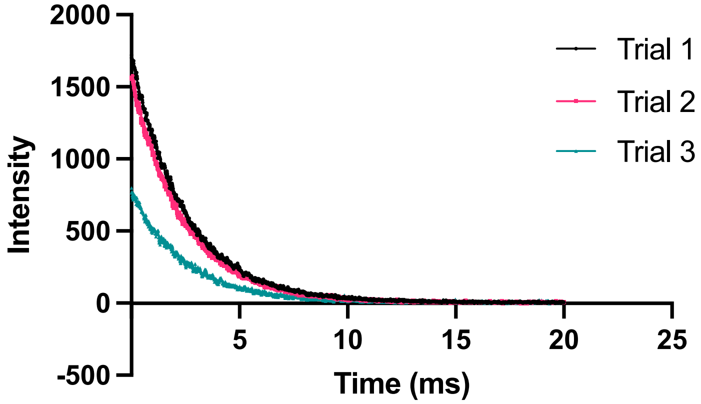


| | | | |
|---|---|---|---|
| **One phase decay** | | | |
| **Best-fit values** | | | |
| Y0 | 1715 | 1562 | 769.9 |
| Plateau | 5.340 | 5.266 | 6.816 |
| K | 0.4076 | 0.4150 | 0.4040 |
| Half Life | 1.700 | 1.670 | 1.716 |
| Tau | 2.453 | 2.410 | 2.475 |
| Span | 1710 | 1556 | 763.1 |
| **95% CI (profile likelihood)** | | | |
| Y0 | 1713 to 1718 | 1559 to 1564 | 767.6 to 772. |
| Plateau | 4.765 to 5.91 | 4.705 to 5.82 | 6.258 to 7.37 |
| K | 0.4067 to 0.4 | 0.4139 to 0.4 | 0.4020 to 0.4 |
| Half Life | 1.696 to 1.70 | 1.666 to 1.67 | 1.707 to 1.72 |
| Tau | 2.447 to 2.45 | 2.404 to 2.41 | 2.463 to 2.48 |
| **Goodness of Fit** | | | |
| Degrees of Freedom | 1998 | 1998 | 1998 |
| R squared | 0.9994 | 0.9992 | 0.9970 |
| Sum of Squares | 176050 | 169696 | 163796 |
| Sy.x | 9.387 | 9.216 | 9.054 |
| **Constraints** | | | |
| K | K > 0 | K > 0 | K > 0 |
| | | | |
| **Number of points** | | | |
| # of X values | 2001 | 2001 | 2001 |
| # Y values analyzed | 2001 | 2001 | 2001 |

**Figure S80:** Tb(bispic-PPP-PPSer) lifetimes in $D_2O$

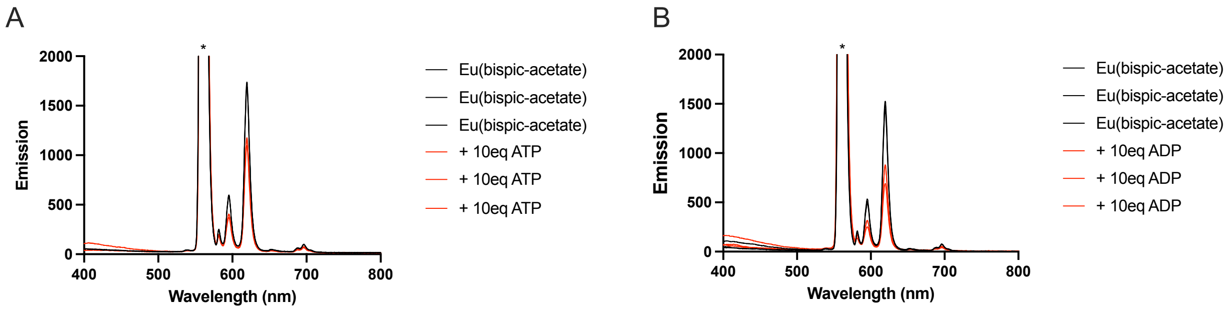


**Figure S81:** Emission spectra comparing Eu(bispic-acetate) emission with and without 10 equivalents of ATP (A) or ADP (B).

## NMR Experiments

NMR experiments were performed on ligands (5 mg of bispic-PSer or bispic-PPP-PSer or 1 mg of bispic-GGG-PSer) dissolved in $D_2O$ (450 μL), and the residual solvent peak in the $^1H$ was referenced to 4.79 ppm. All other experiments were absolute referenced to the $^1H$ experiment. Following ligand characterization, one equivalent of $Eu^{3+}$ or $Lu^{3+}$ ($Lu^{3+}$ only for bispic-PSer) dissolved in 2 μL of $D_2O$ was added to the NMR tube, and the experiments were repeated.

## 5.1 **Bispic-PSer**

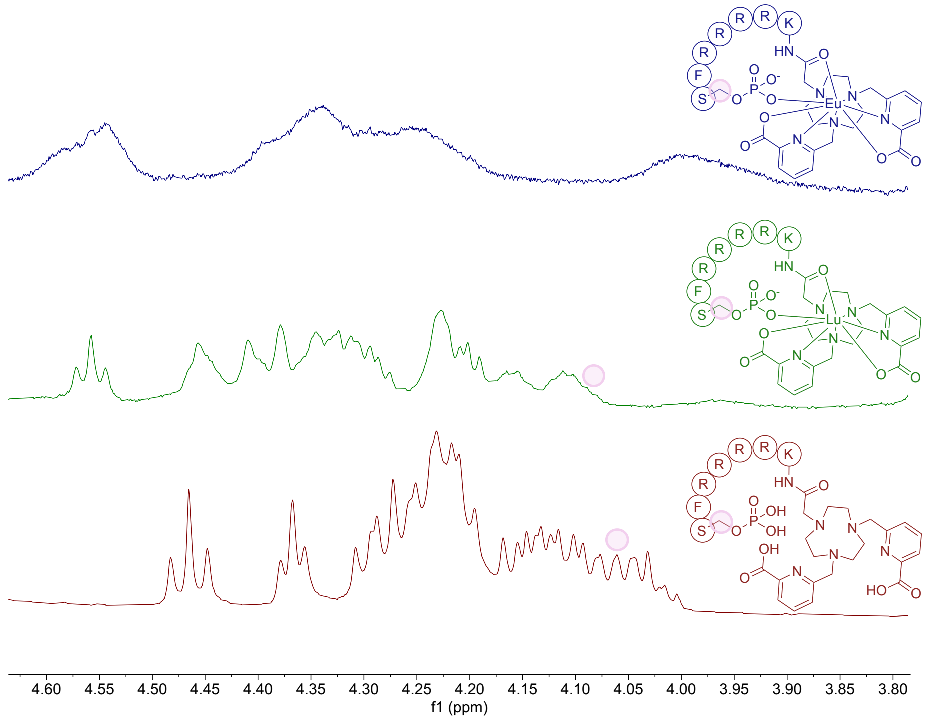

**Figure S82:** $^{1}$H NMR comparing bispic-PSer to [Eu(bispic-PSer)]$^{-}$ and [Lu(bispic-PSer)]$^{-}$. The pink circles indicate the $CH_2$ on the phosphorylated serine as confirmed by $^{1}$H$^{31}$P HMBC.

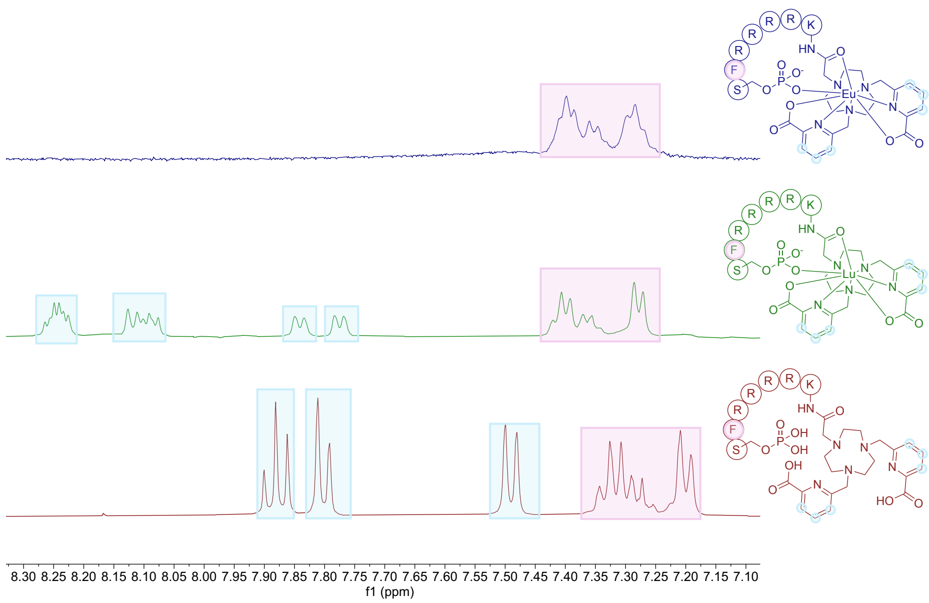

**Figure S83:** $^{1}$H NMR comparing bispic-PSer to [Eu(bispic-PSer)]$^{-}$ and [Lu(bispic-PSer)]$^{-}$. The pink indicates the phenylalanine residue, and the blue indicated the picolinate protons. The phenylalanine protons do not coordinate to the metal center, so they do not shift substantially. The picolinate protons are in close proximity to the metal center, affecting their NMR shift.

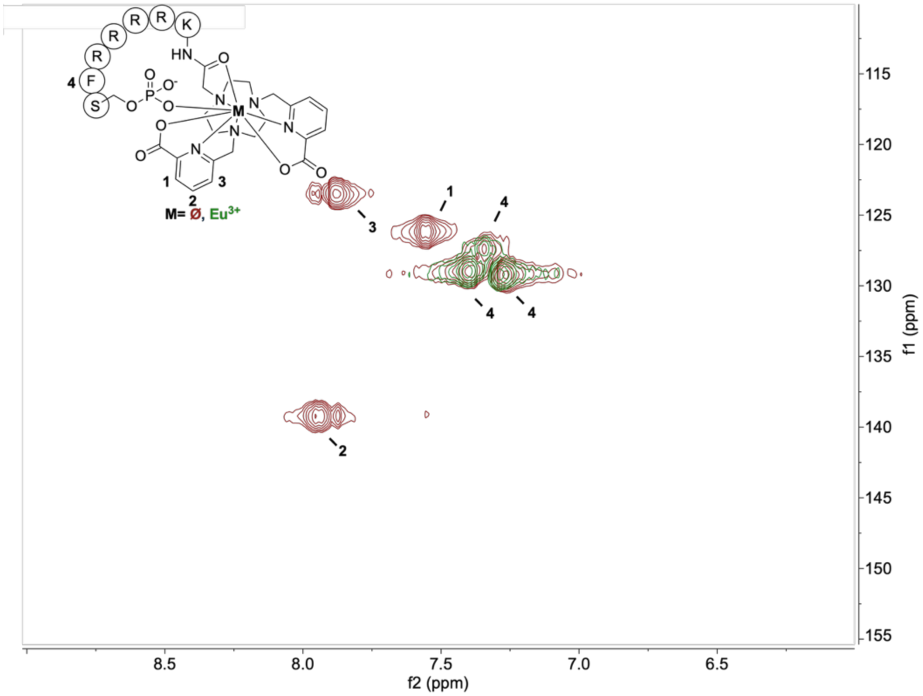


**Figure S84:** Aromatic region of HSQC NMR comparing bispic-PSer to [Eu(bispic-PSer)]$^-$. The disappearance of peaks indicates close proximity to $Eu^{3+}$.

5.2 **Bispic-PPP-PSer**

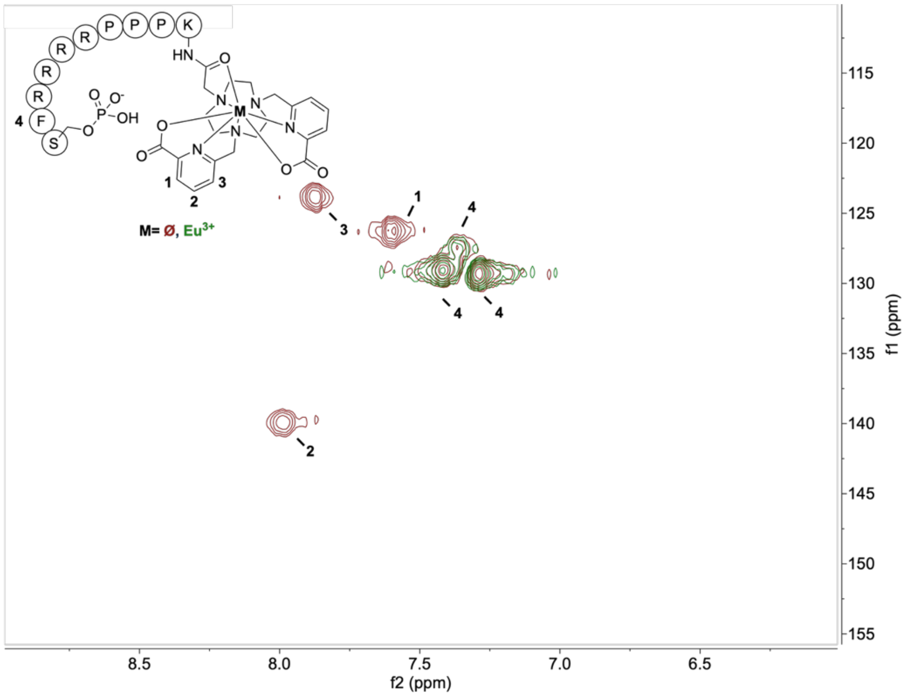


**Figure S85:** Aromatic region of HSQC NMR comparing bispic-PPP-PSer to [Eu(bispic-PPP-PSer)]$^-$. The disappearance of peaks indicates close proximity to $Eu^{3+}$.

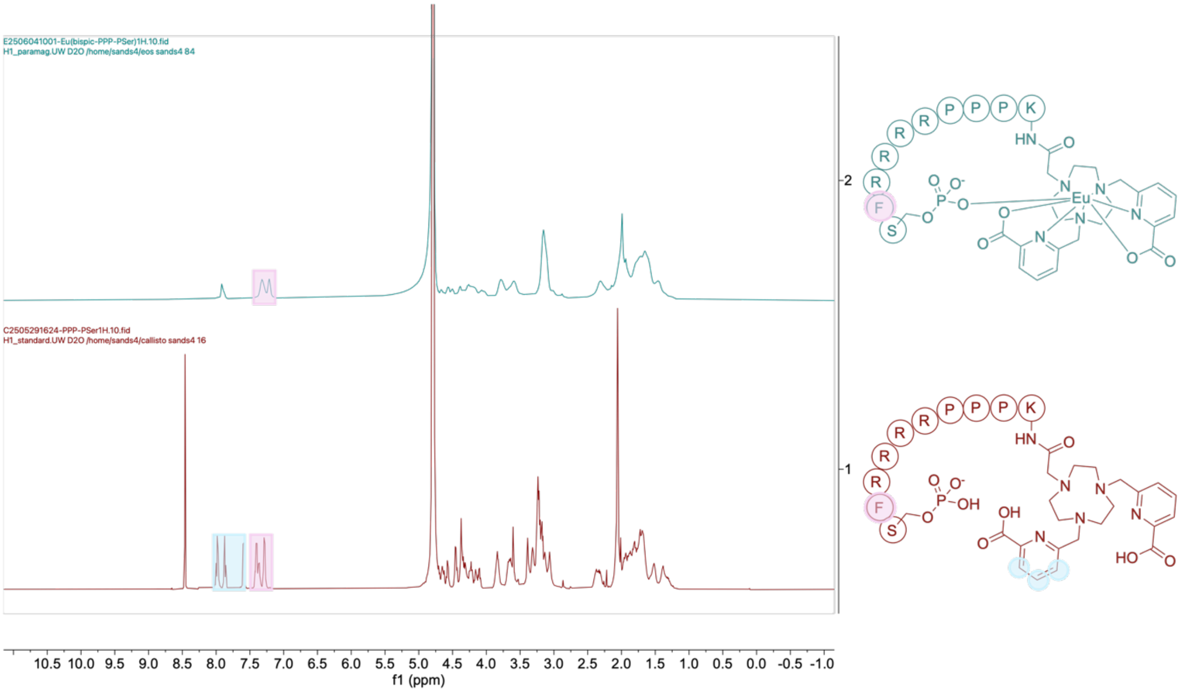


**Figure S86:** $^{1}$H NMR comparing bispic-PPP-PSer to [Eu(bispic-PPP-PSer)]$^{-}$.

## 5.3 **Bispic-GGG-PSer**

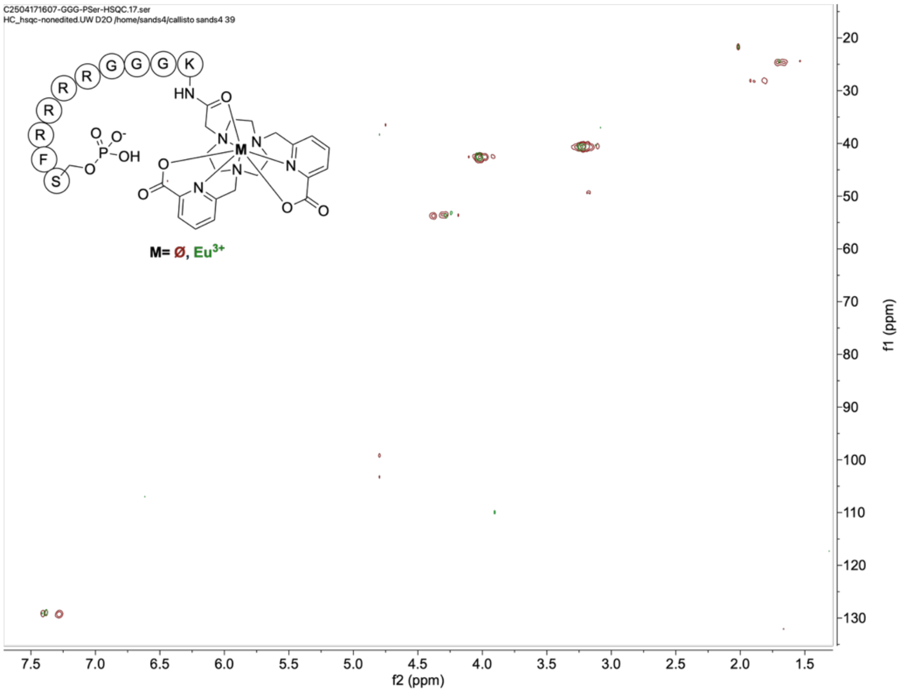


**Figure S87:** $^{1}$H NMR comparing bispic-GGG-PSer to [Eu(bispic-GGG-PSer)]$^{-}$.

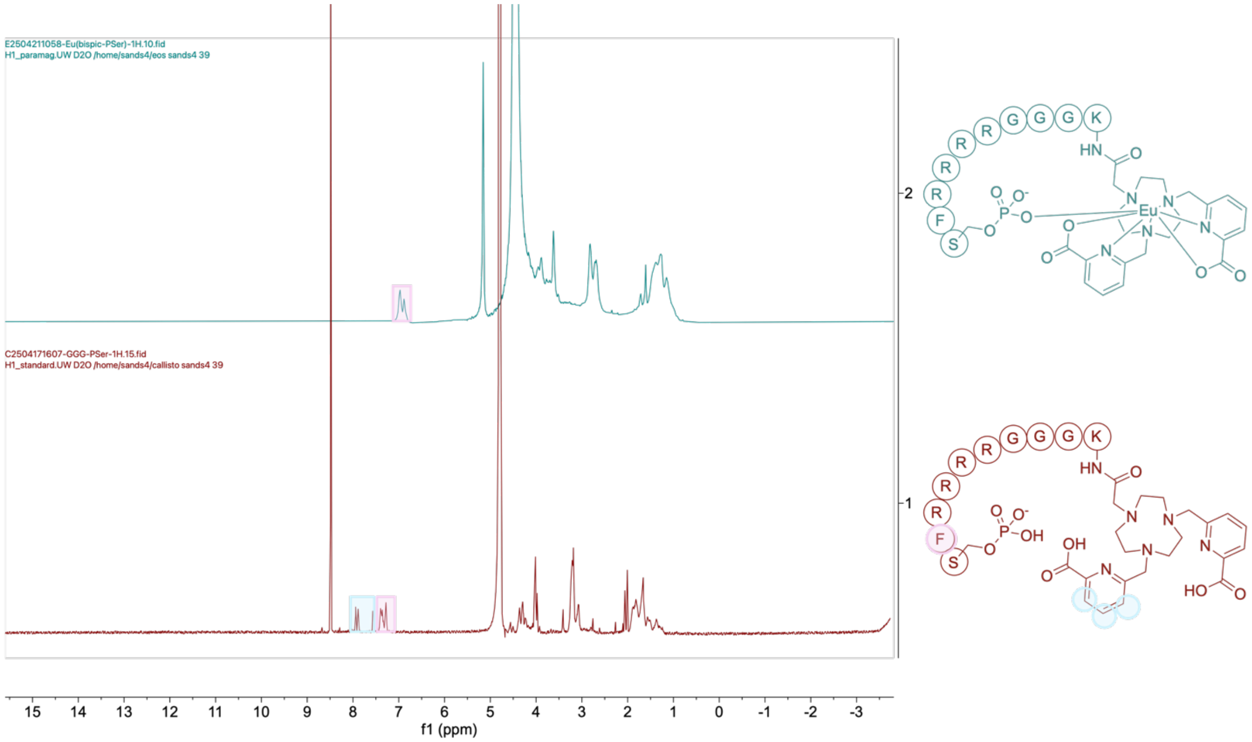


**Figure S88:** $^{1}$H NMR comparing bispic-GGG-PSer to [Eu(bispic-GGG-PSer)]$^{-}$.

## Computational Methods

6.1 Water coordination and binding affinities for $Tb^{3+}$ complexes with different linker peptides

**Table S6. Summary of water coordination and binding affinities for $Tb^{3+}$ complexes with different linker peptides.**

| System | #Coordinating water with Ser | #Coordinating water with PSer | Binding Affinity with Ser (kJ/mol) | Binding Affinity with PSer (kJ/mol) |
|---|---|---|---|---|
| **Tb(bispic)** | 0.88 ± 0.17 | 0.08 ± 0.02 | -93.22 ± 42.12 | -1218.92 ± 37.22 |
| **Tb(bispic-GGG)** | 0.90 ± 0.15 | 0.10 ± 0.03 | -85.01 ± 45.37 | -1109.83 ± 56.50 |
| **Tb(bispic-PPP)** | 0.87 ± 0.20 | 0.11 ± 0.03 | -88.74 ± 31.75 | -1059.15 ± 68.79 |

## Part III - Spectra

## Synthesis of [Ln(bispic-amide)]$^+$

## S1

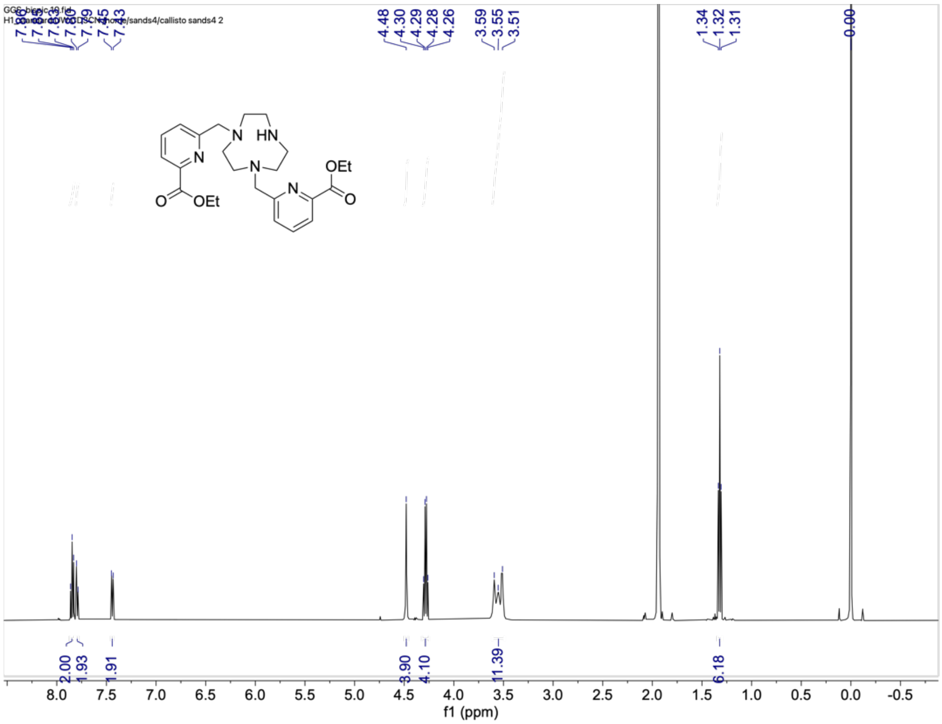

**Figure S89:** $^{1}$H NMR of **1**

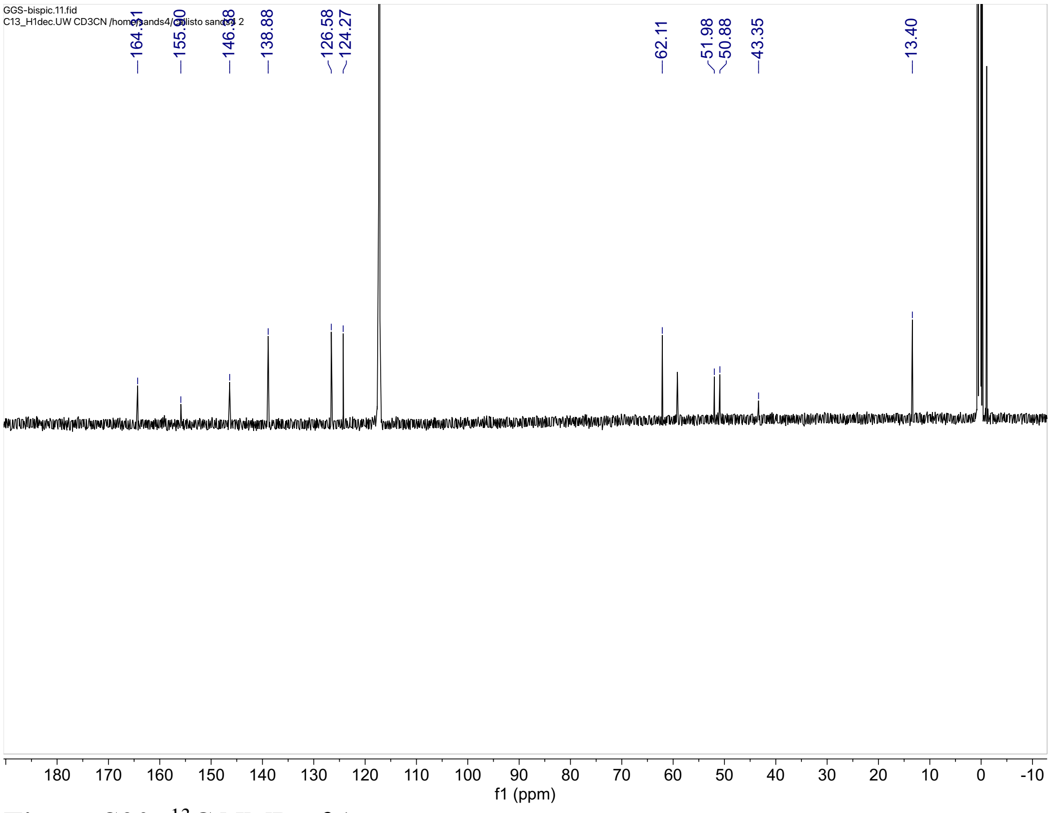

**Figure S90:** $^{13}$C NMR of **1**

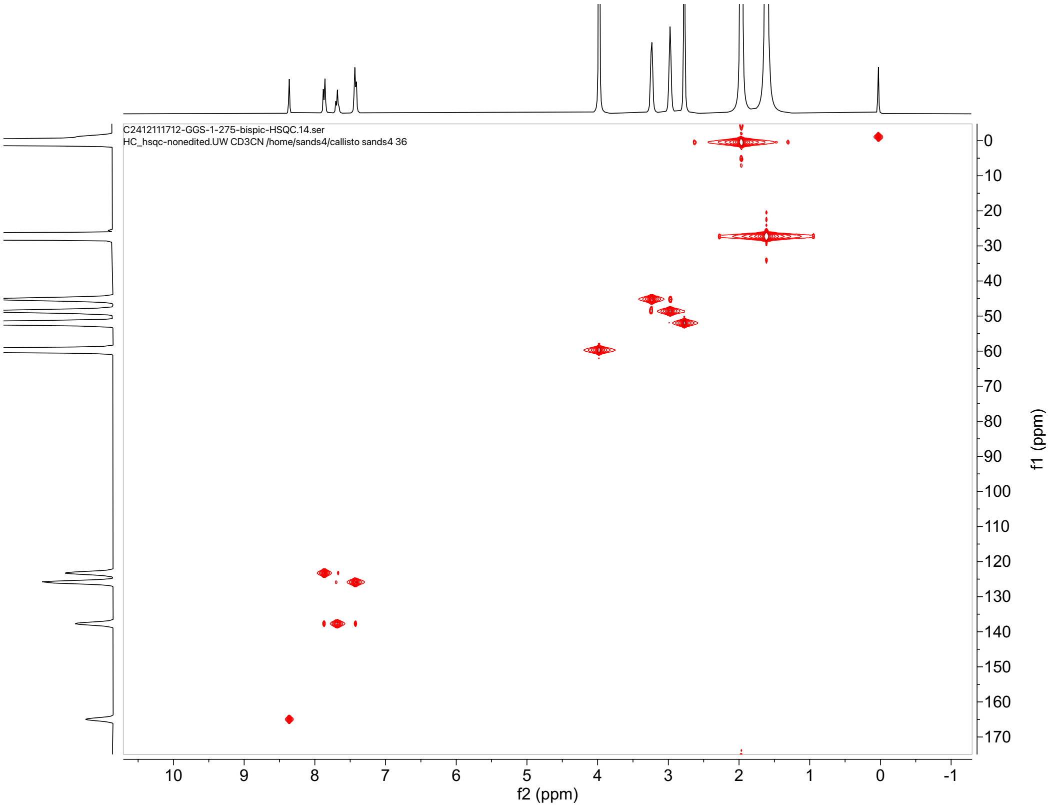


**Figure S91:** HSQC of **1**

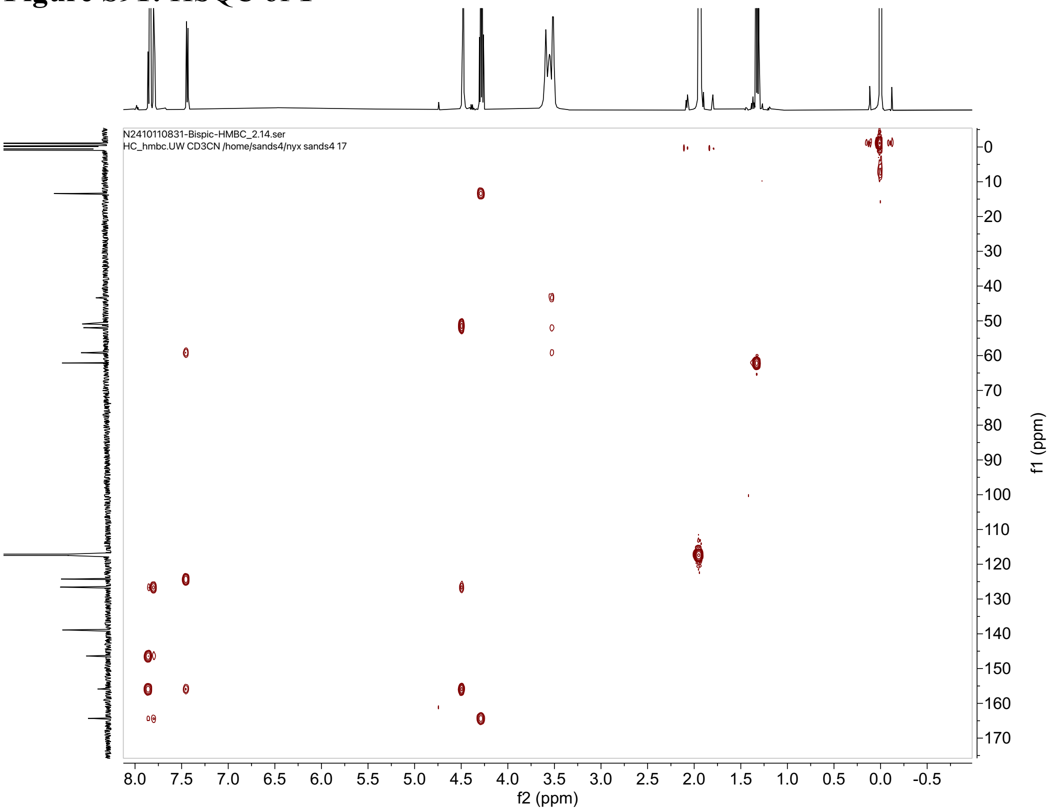


**Figure S92:** HMBC of **1**

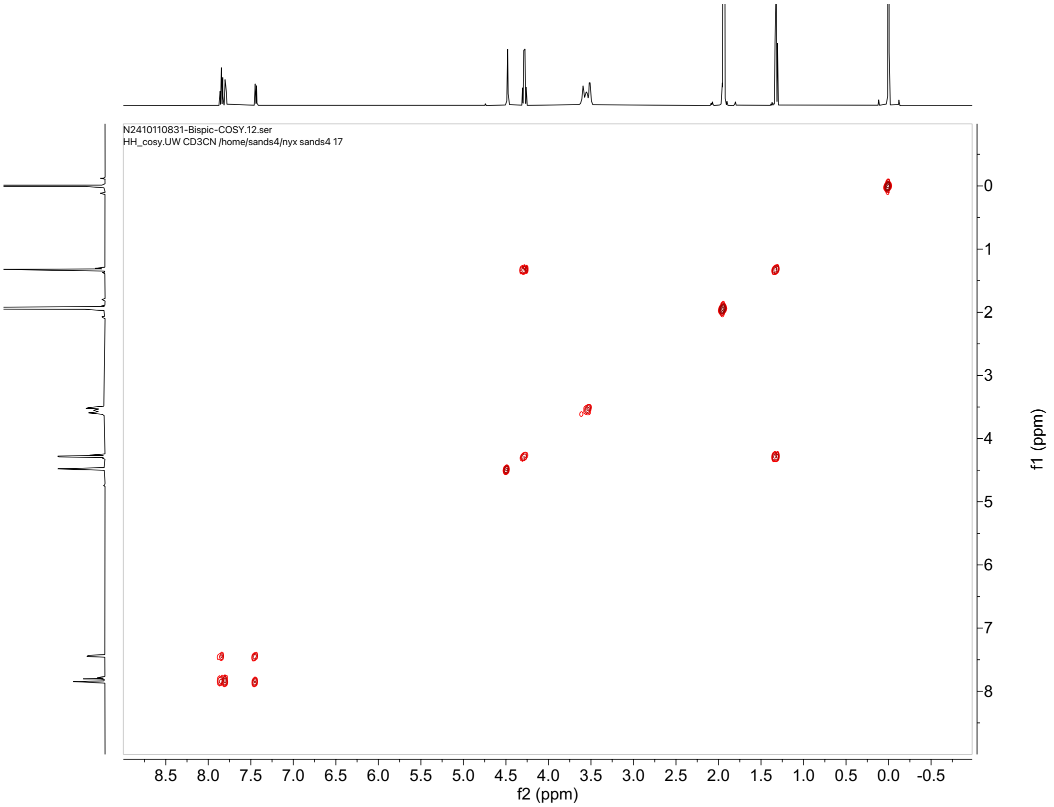


**Figure S93:** COSY of **1**

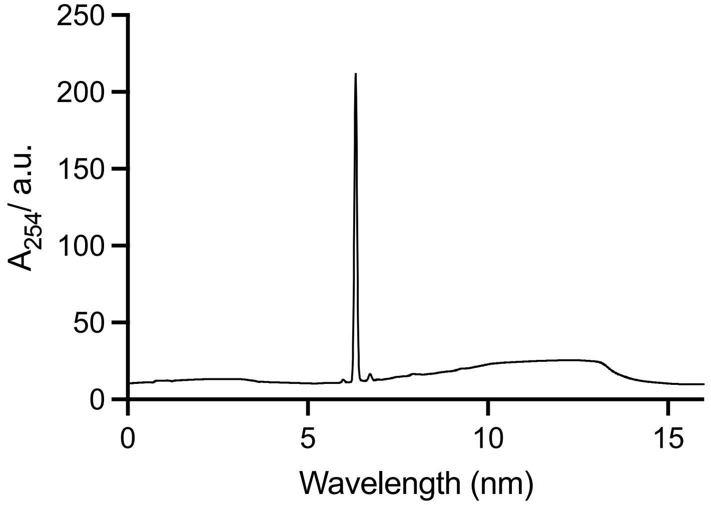


**Figure S94:** HPLC trace of **1** ($R_t$= 6.34 min) (method E)

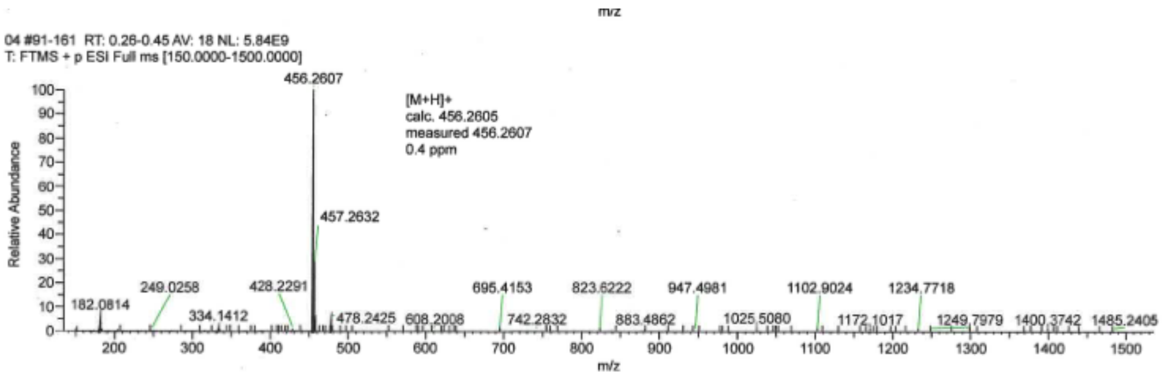


**Figure S95:** HR-ESI MS of **1**

**S2**

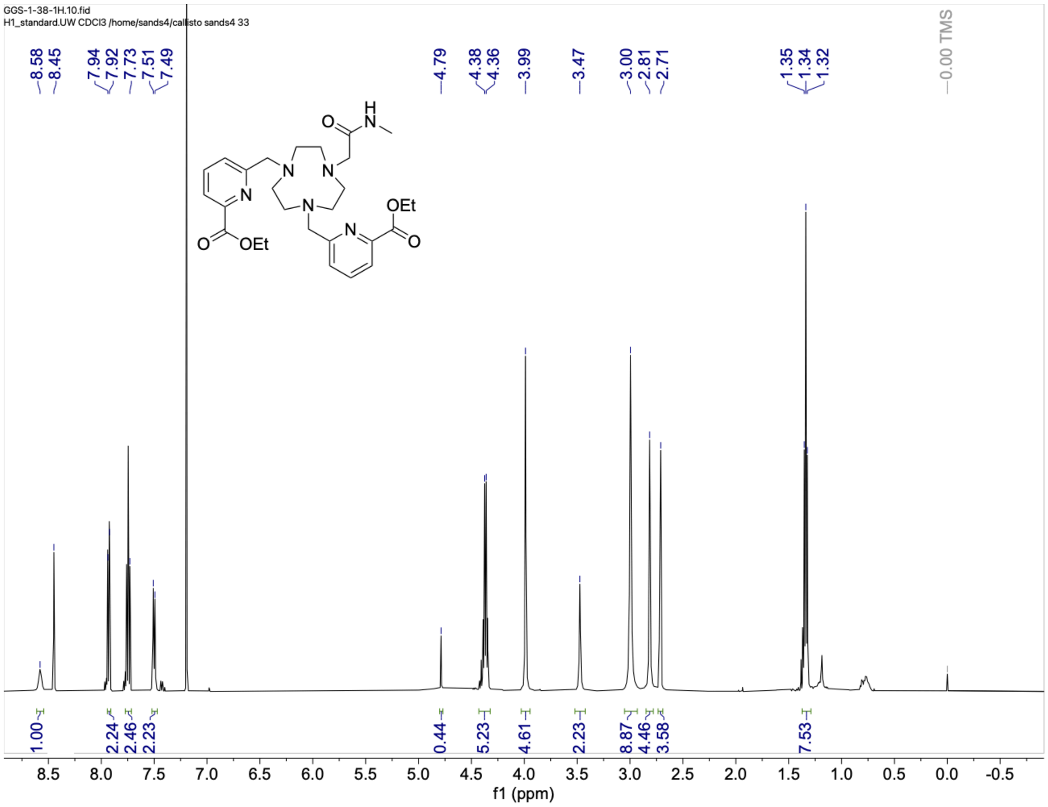


**Figure S96:** $^{1}$H NMR of **2** (peak at 8.45 is formic acid)

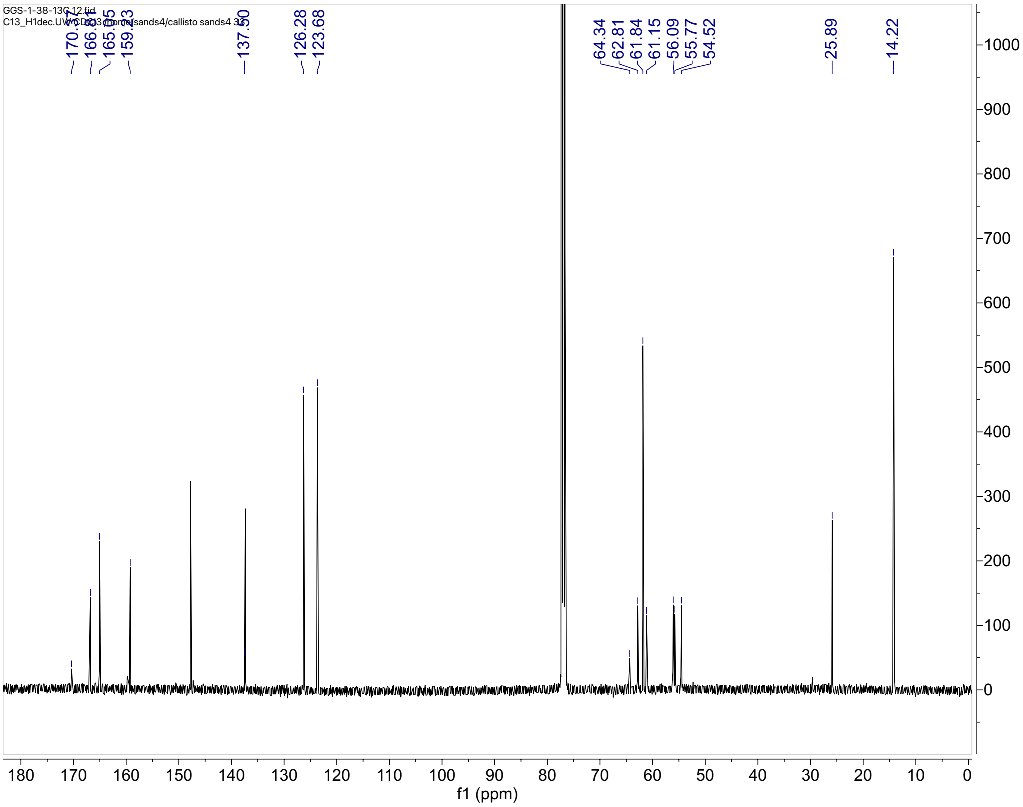


**Figure S97:** $^{13}$C NMR **2**

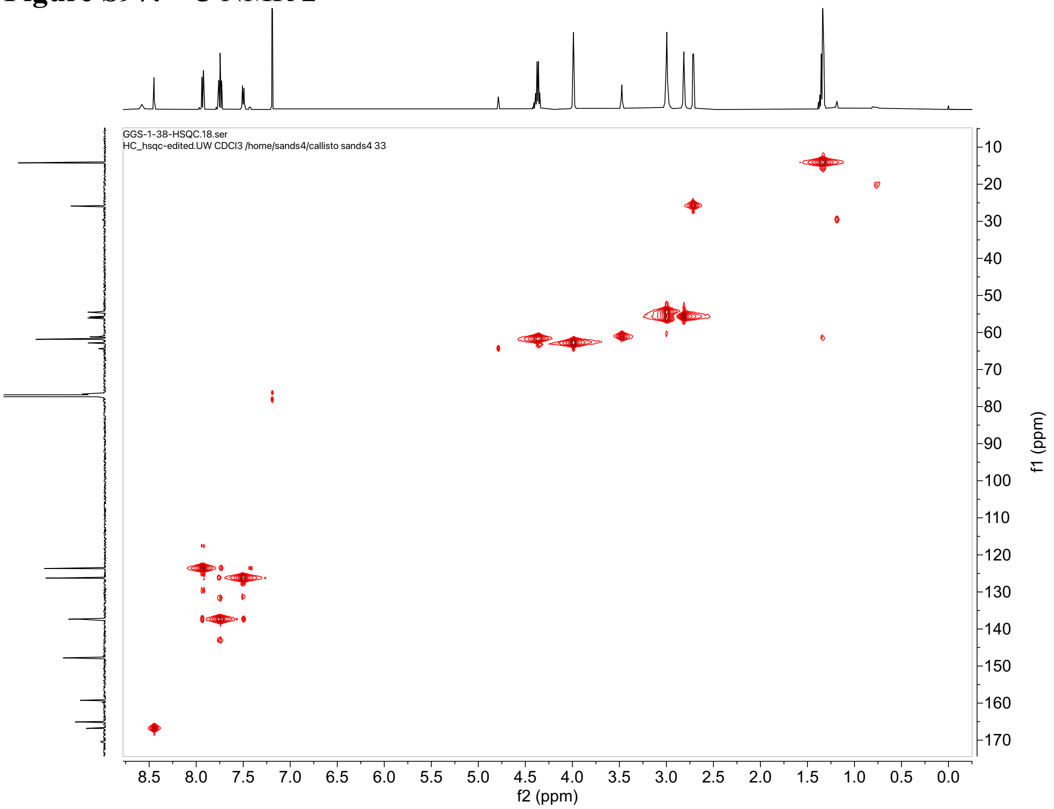


**Figure S98:** HSQC of **2**

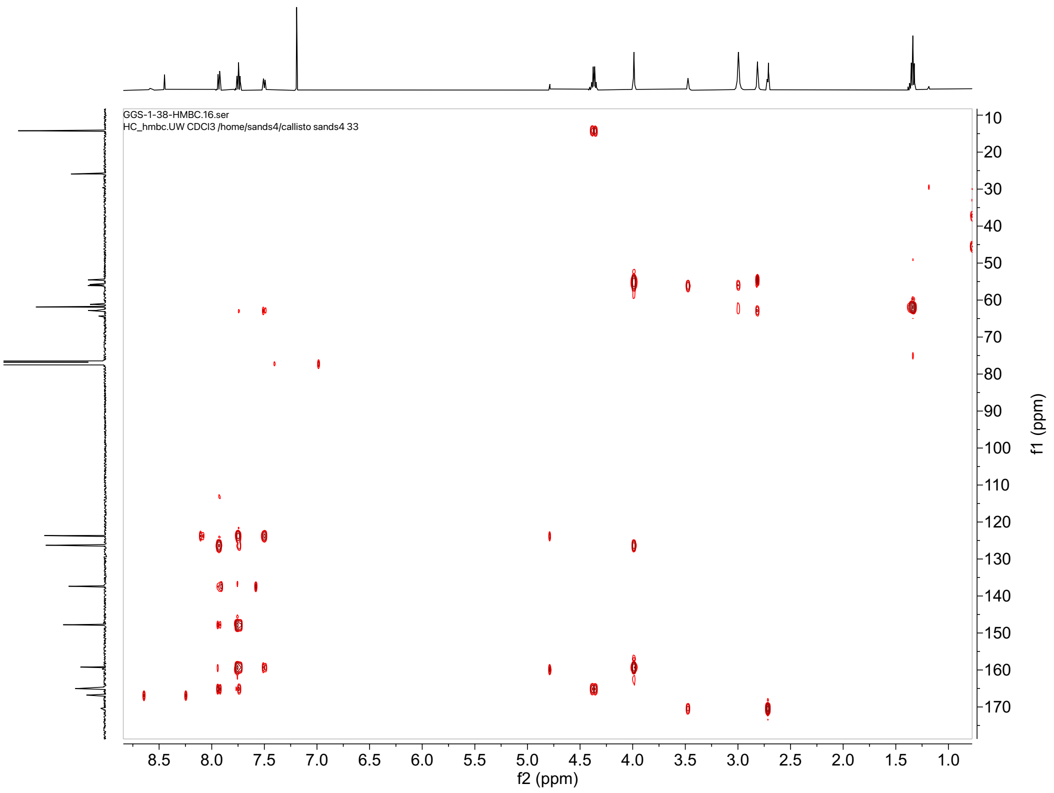


**Figure S99:** HMBC of **2**

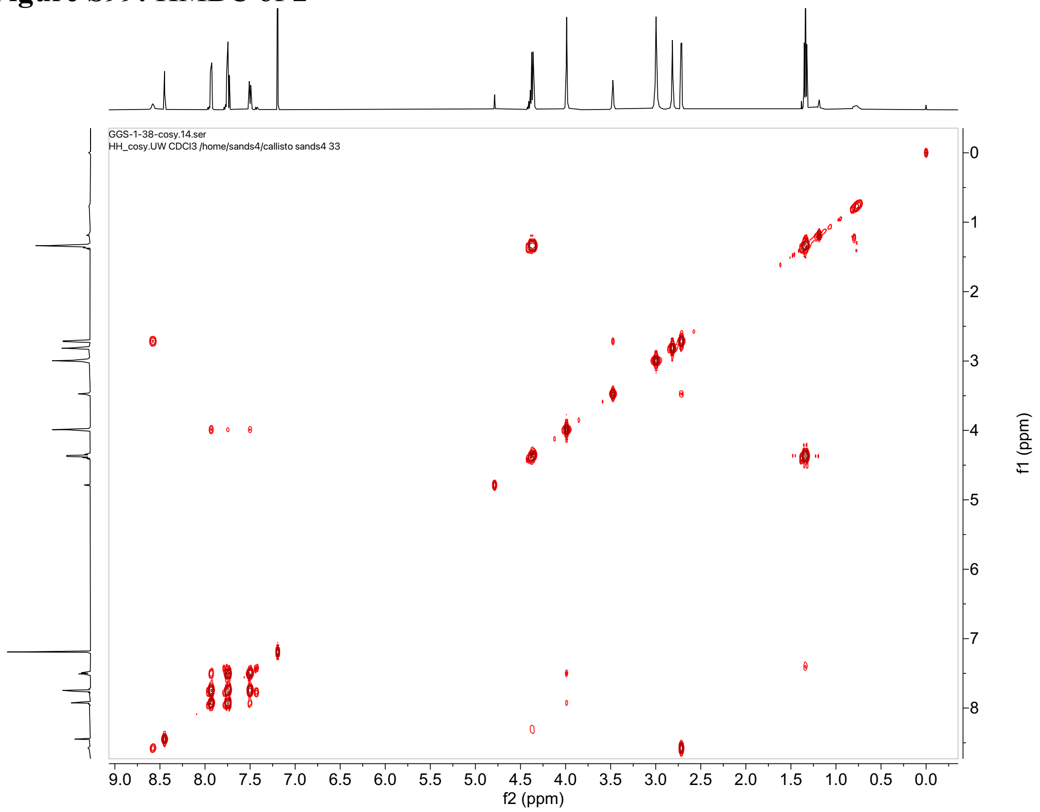


**Figure S100:** COSY of **2**

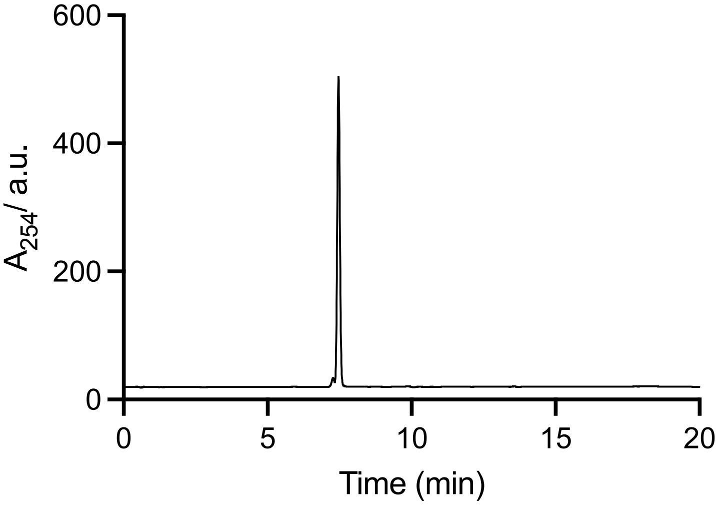


**Figure S101:** HPLC trace of **2** ($R_t$ =7.46 min) (Method C)

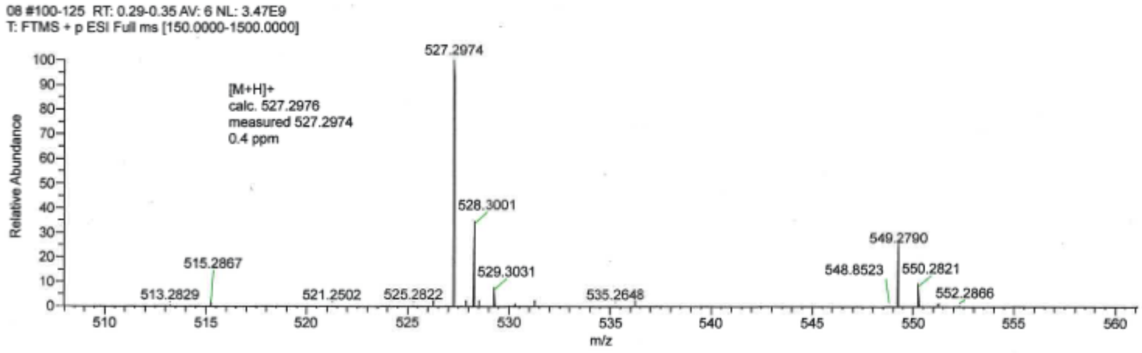


**Figure S102:** HR-ESI MS of **2**

**S3**

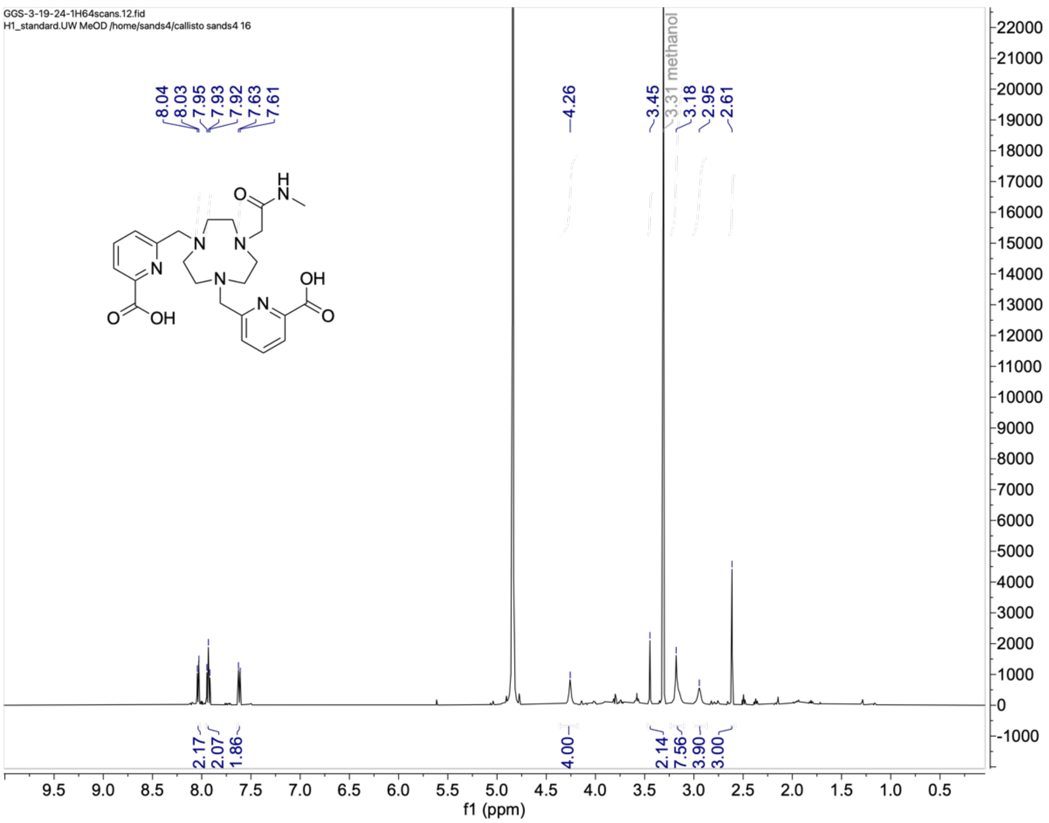


**Figure S103:** $^{1}$H NMR of **3**

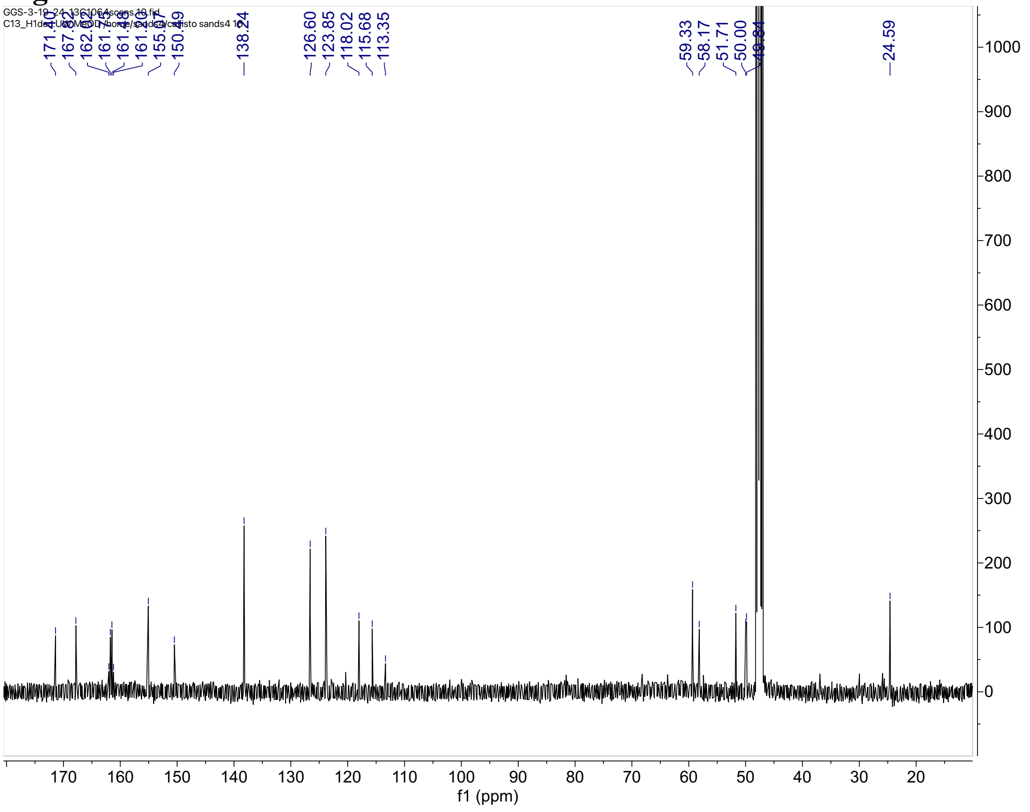

**Figure S104:** $^{13}$C NMR of **3**

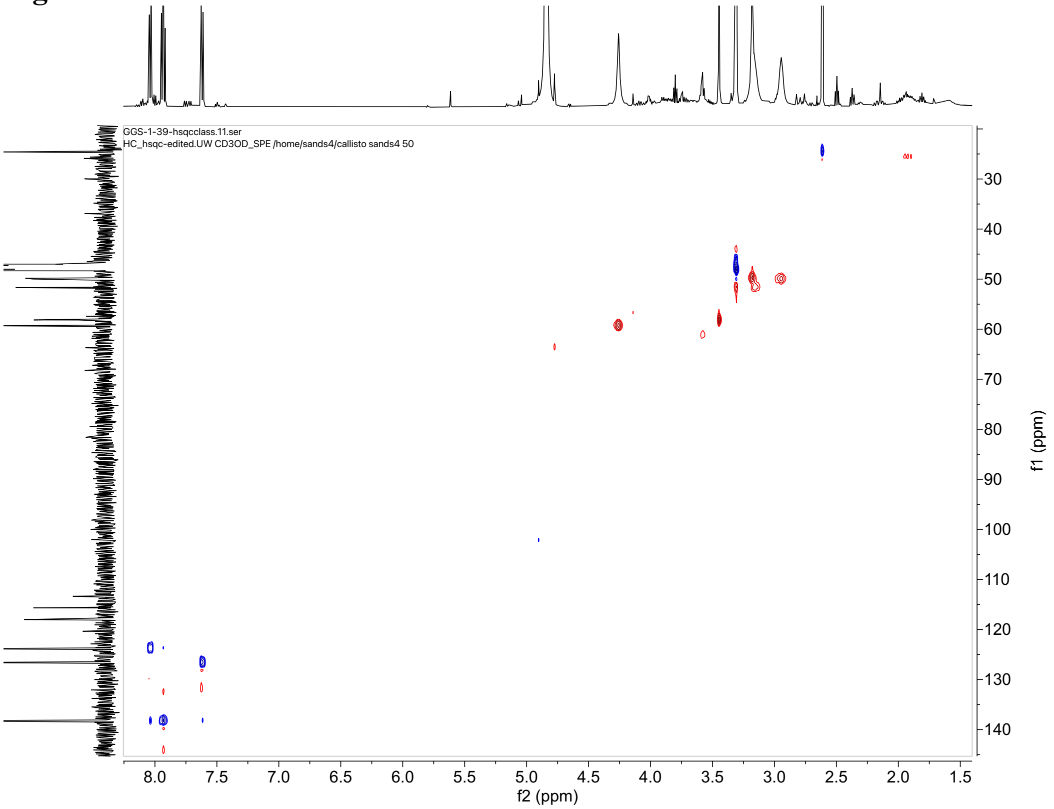


**Figure S105:** HSQC of **3**

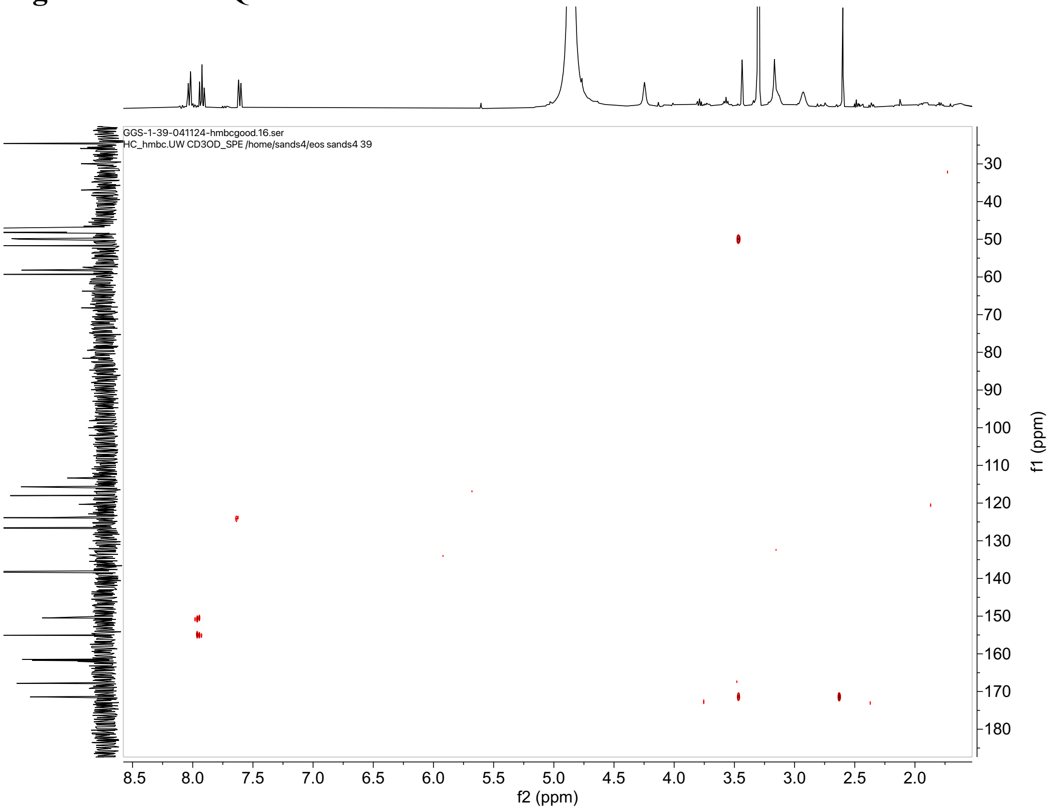


**Figure S106:** HMBC of **3**

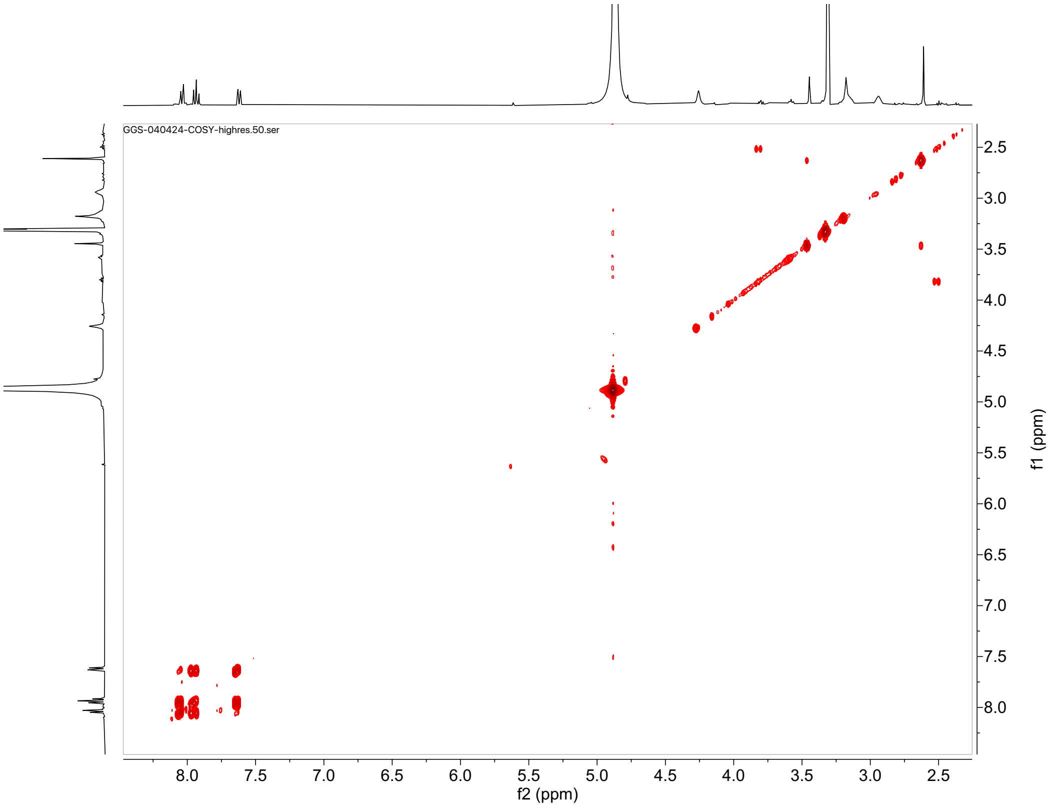


**Figure S107:** COSY of **3**

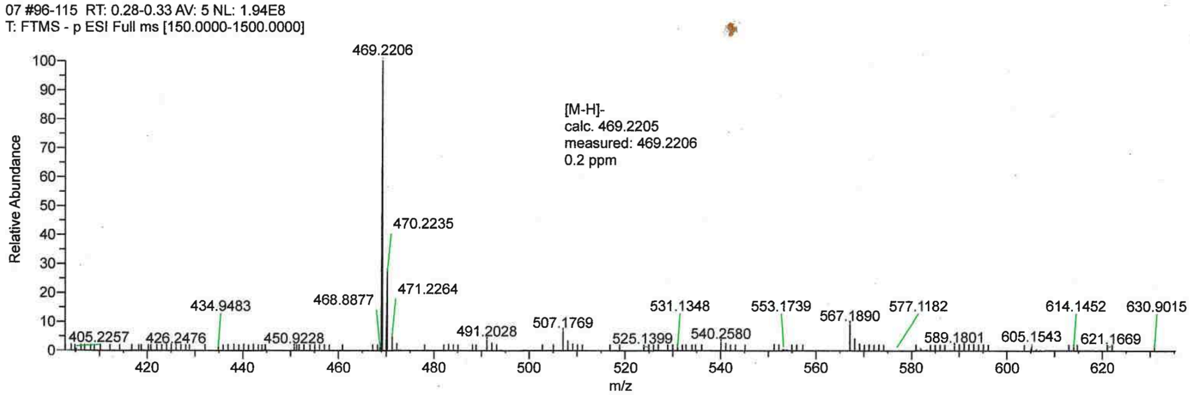


**Figure S108:** HR-ESI MS of **3**

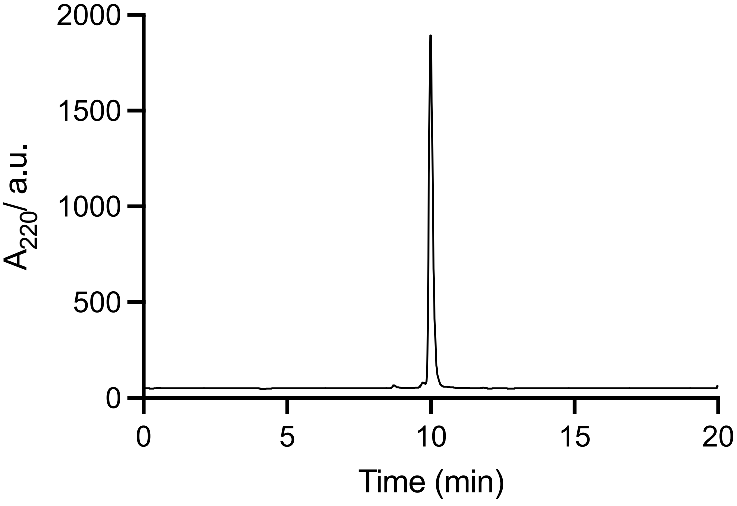


**Figure S109:** HPLC trace of **3** ($R_t$ = 10.01 mins) (method F)

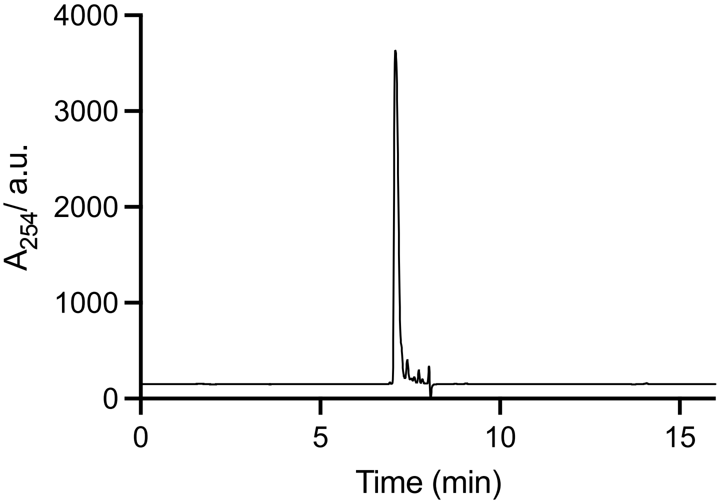


**Figure S110:** HPLC trace of **[Tb(bispic-amide)]$^+$** ($R_t$ = 7.13 mins) (method F)

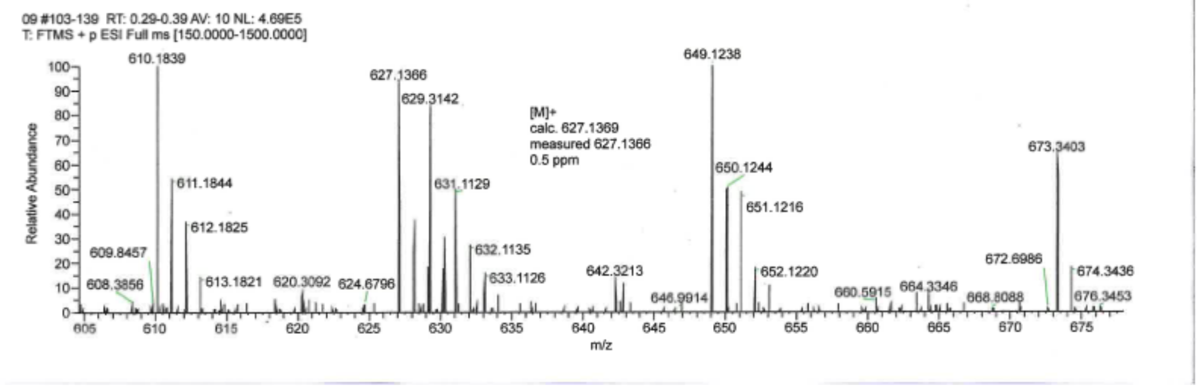


**Figure S111:** HR-ESI MS of **[Tb(bispic-amide]$^+$**

**Synthesis of [Ln(bispic-lysine)]$^{+}$**

**S4**

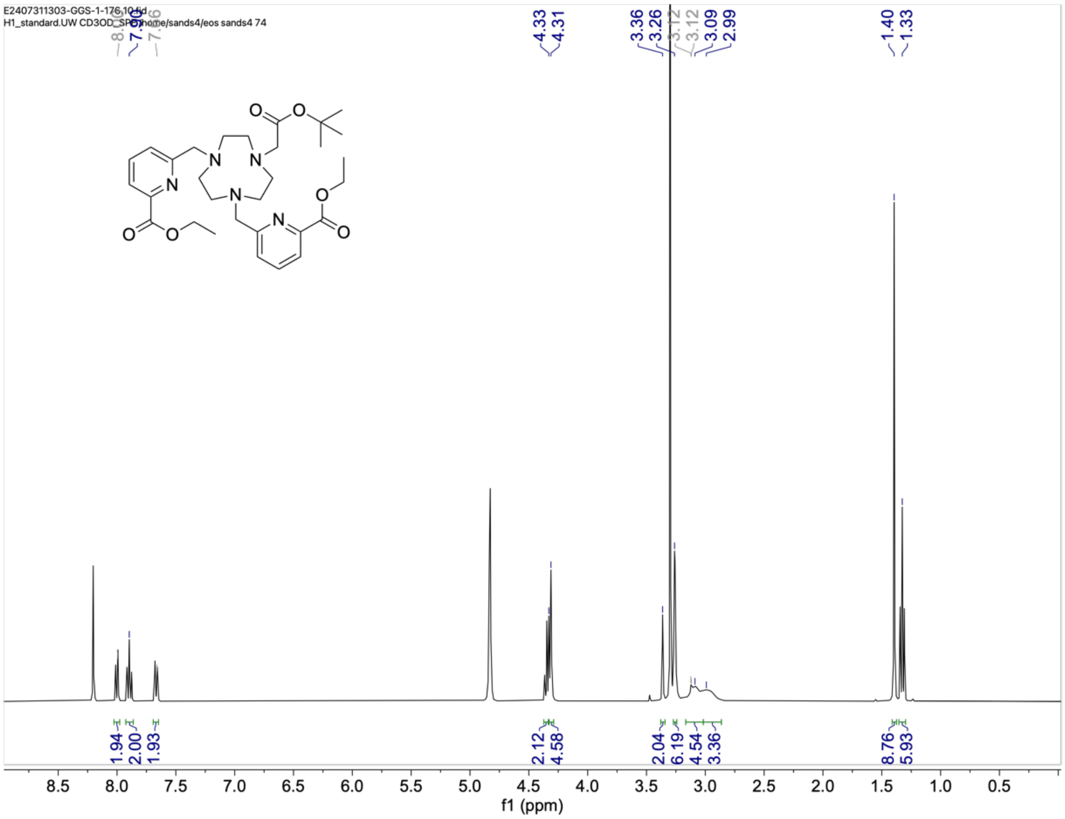


**Figure S112:** $^{1}$H NMR of **4** (peak at 8.35 is formic acid)

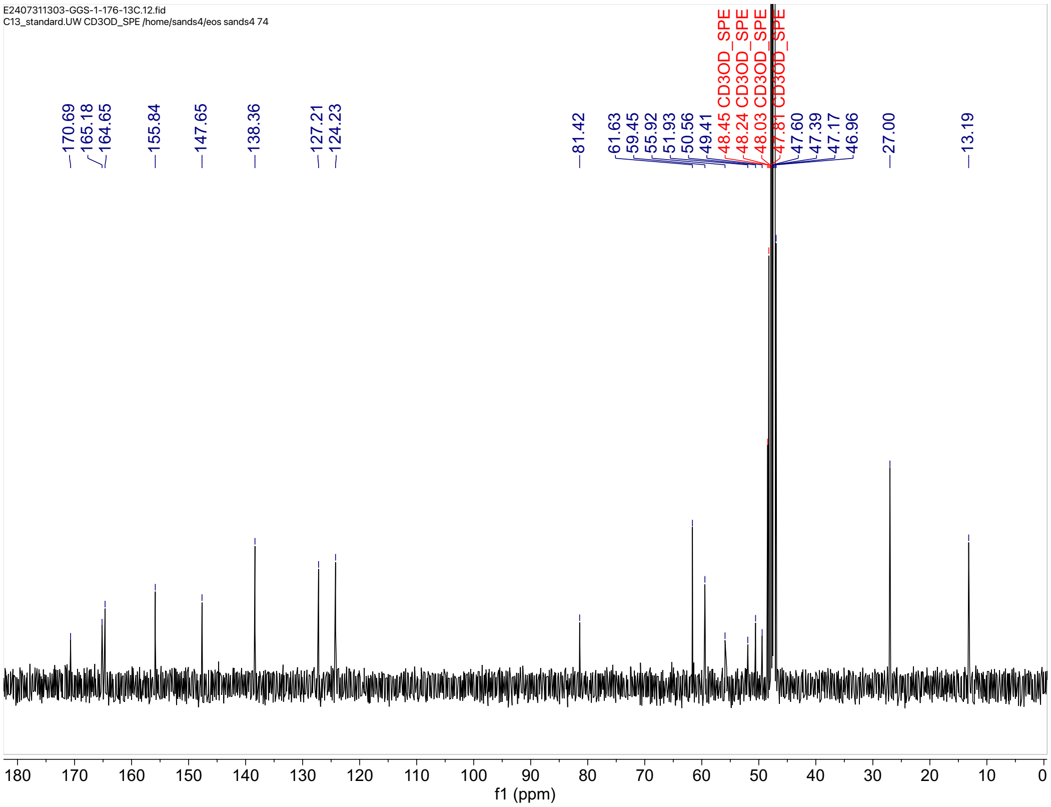


**Figure S113:** $^{13}C$ NMR of **4**

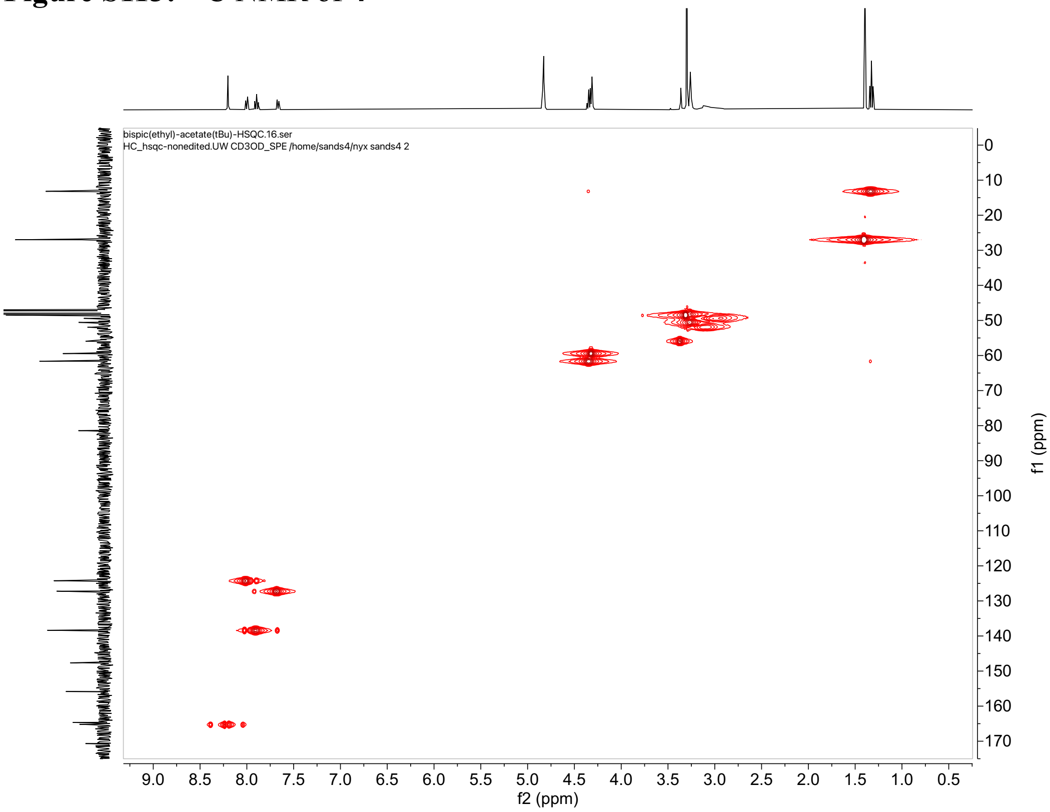


**Figure S114:** HSQC of **4**

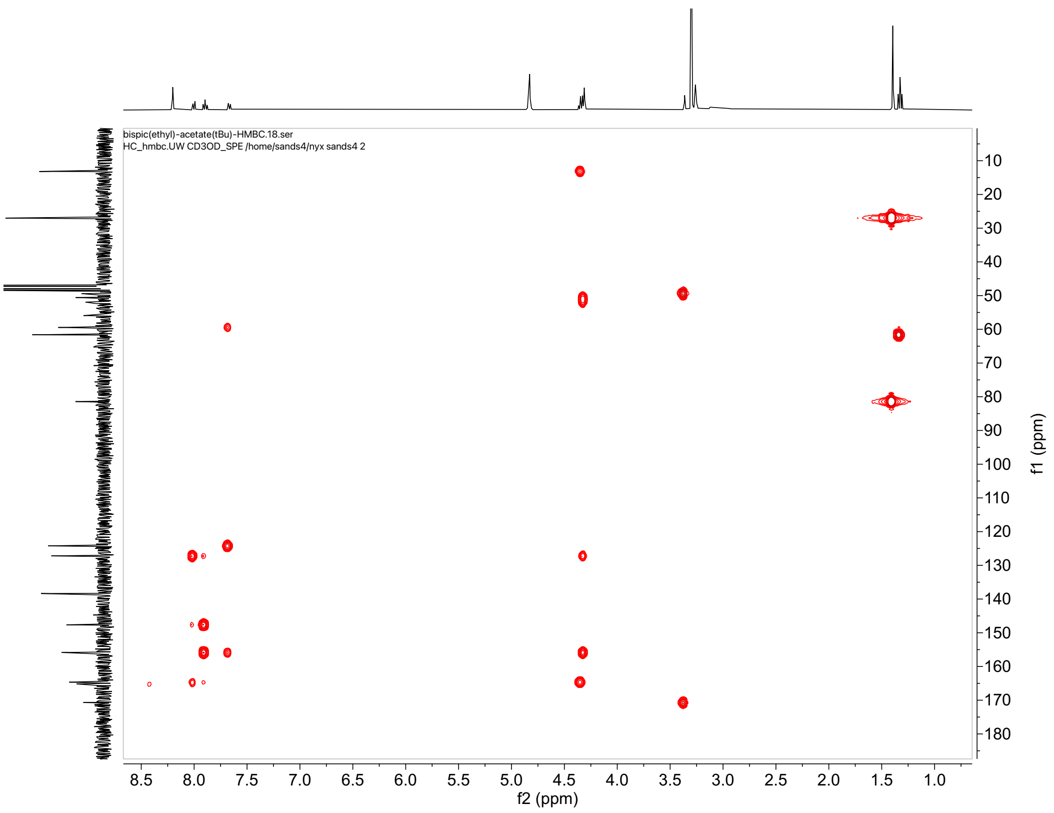


**Figure S115:** HMBC of **4**

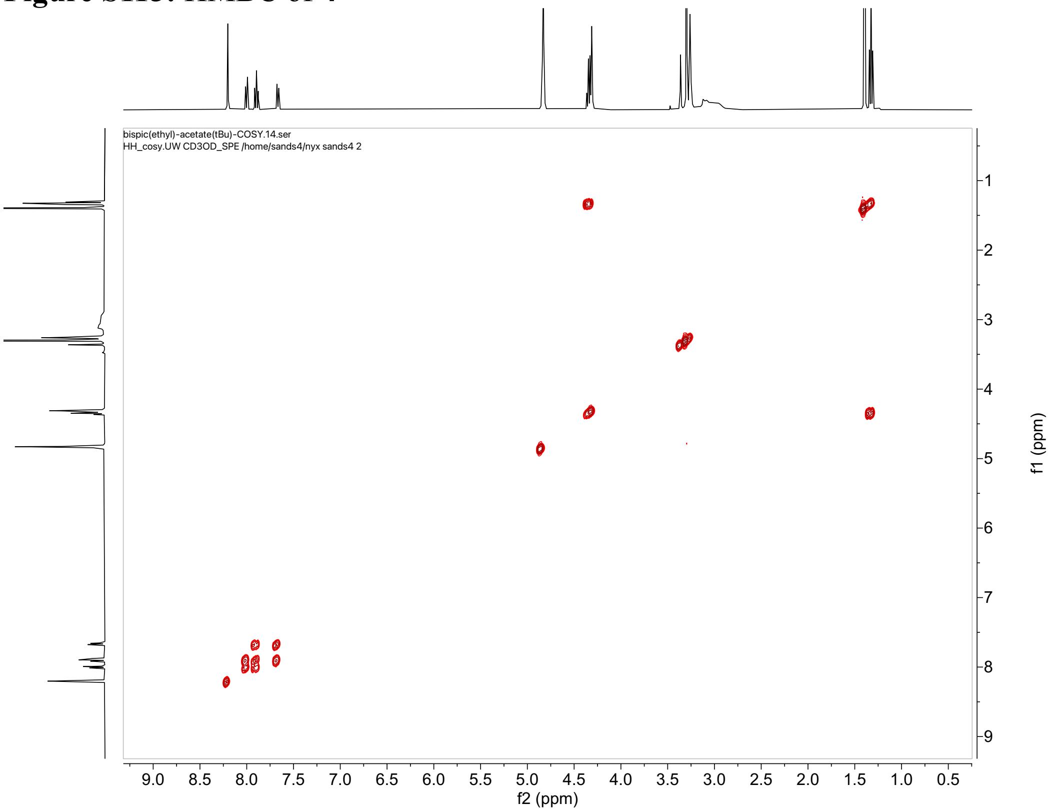


**Figure S116:** COSY of **4**

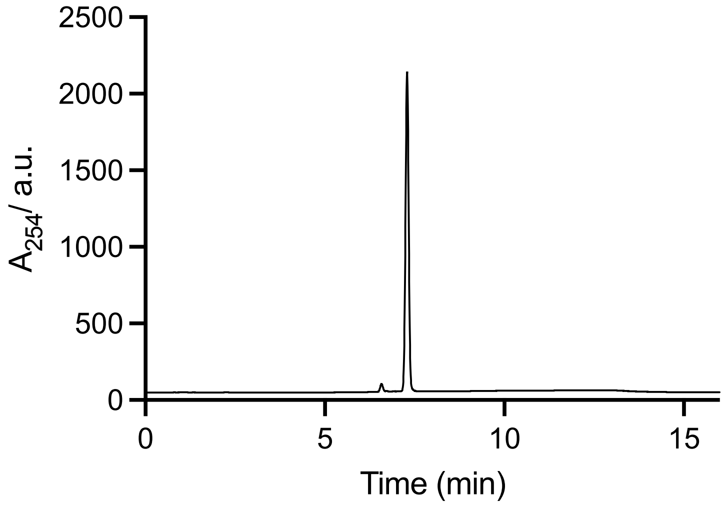


**Figure S117:** HPLC trace of **4** ($R_t$= 7.30 min) (Method E)

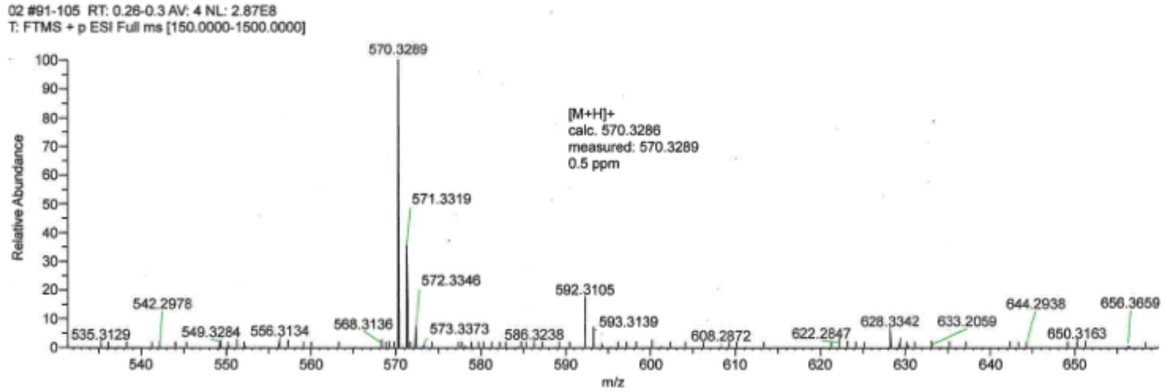


**Figure S118:** HR-ESI MS of **4**

## S5

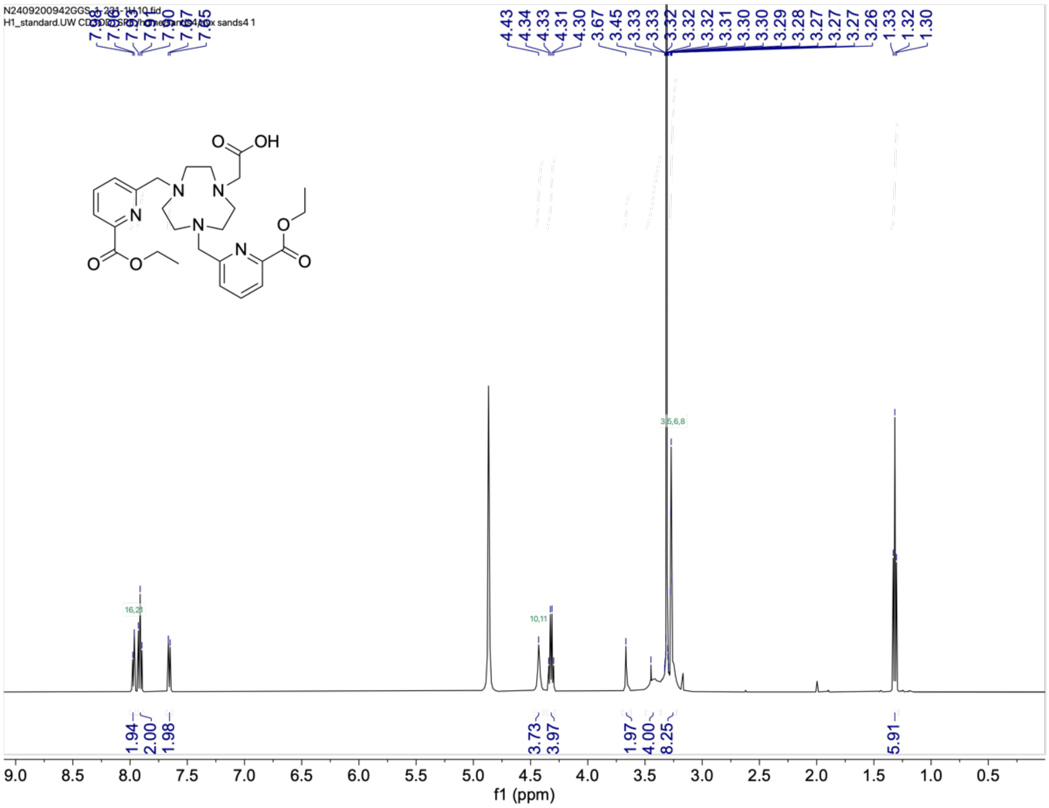


**Figure S119:** $^{1}$H NMR of **5**

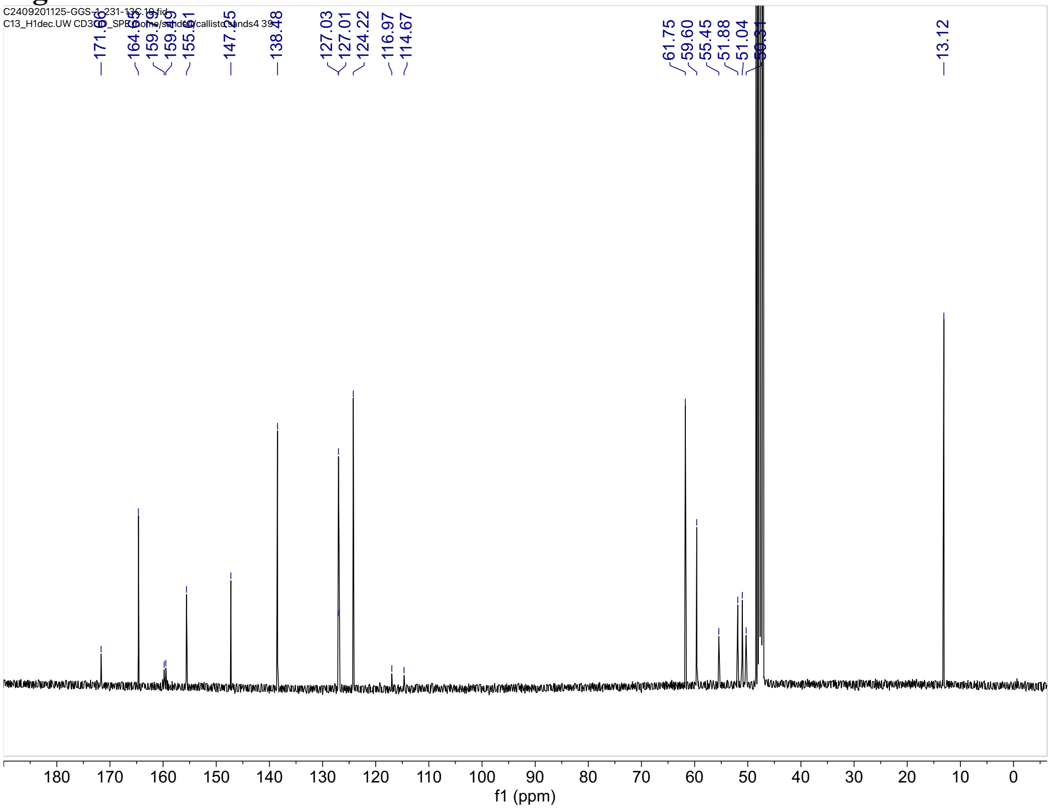


**Figure S120:** $^{13}$C NMR of **5**

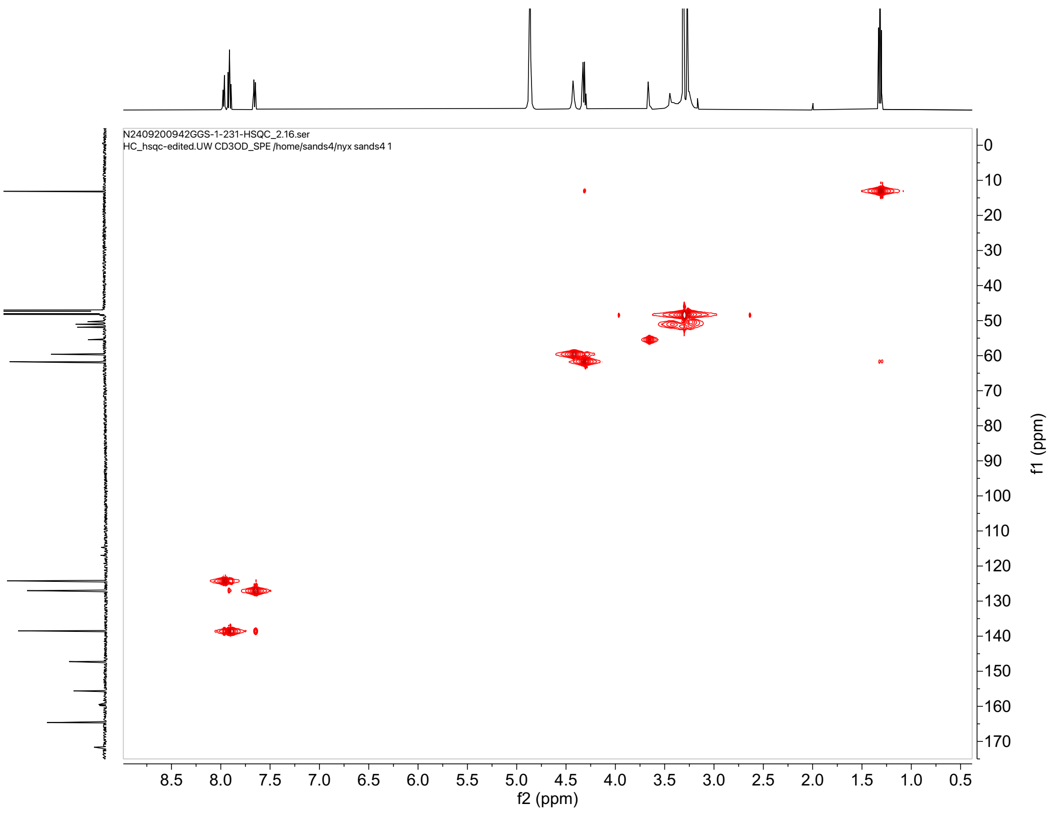


**Figure S121:** HSQC of **5**

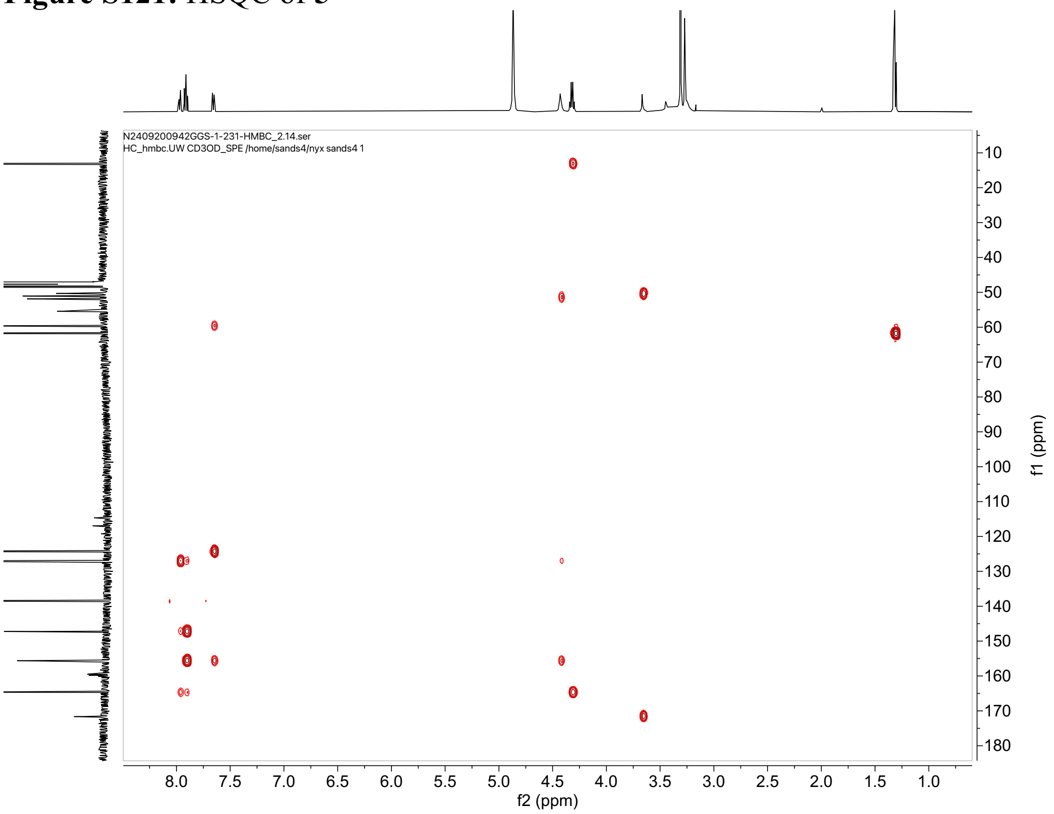


**Figure S122:** HMBC of **5**

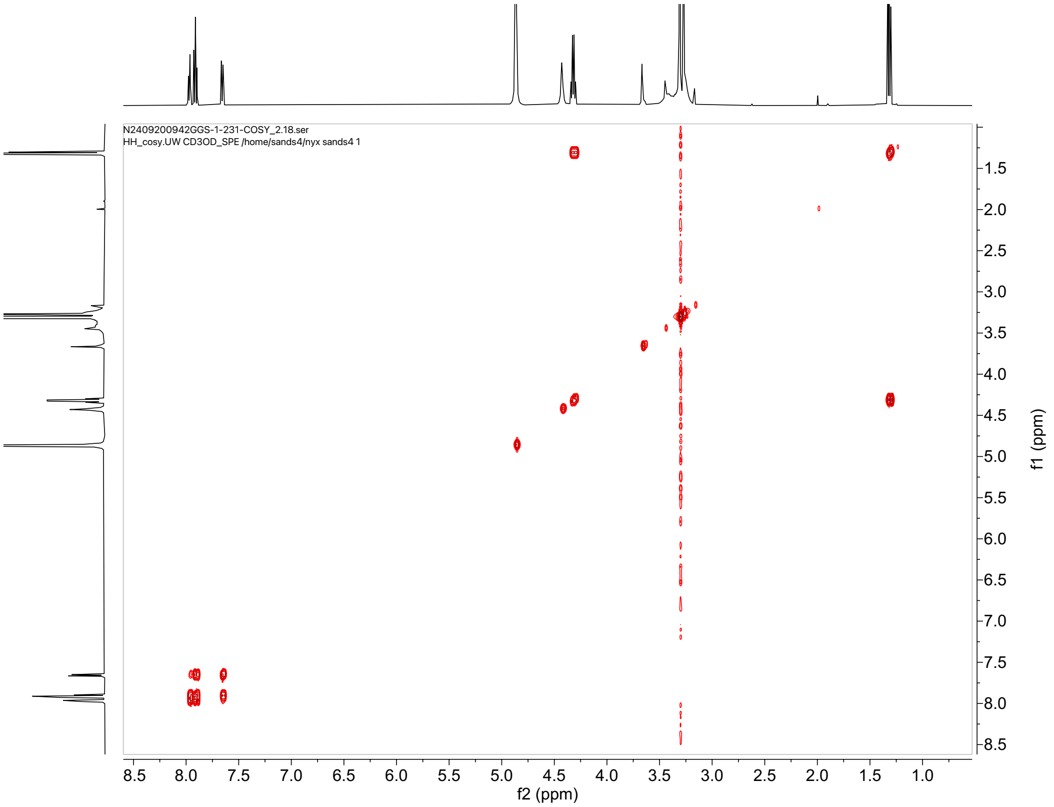


**Figure S123:** COSY of **5**

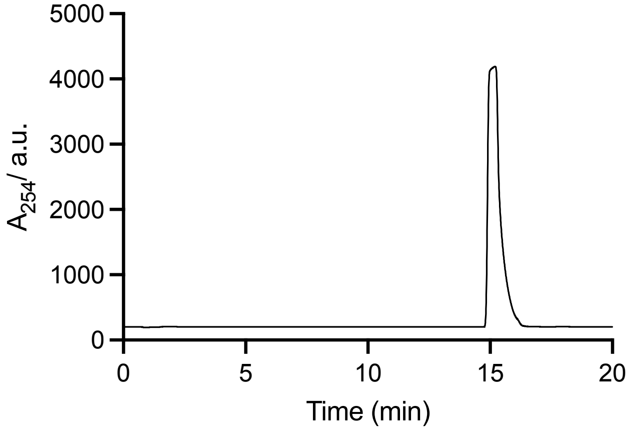


**Figure S124:** HPLC trace of **5** $R_t$ ($R_t$ = 15.25 min) (Method F)

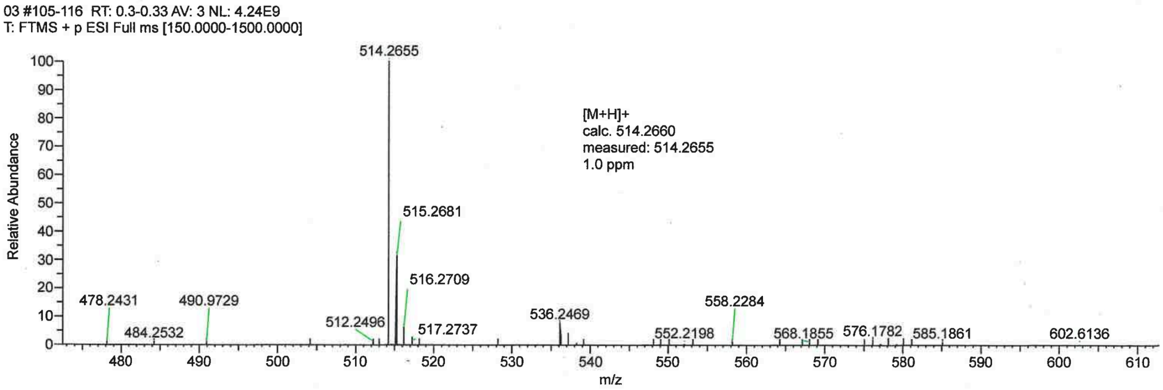


**Figure S125:** HR-ESI MS of **5**

**S7**

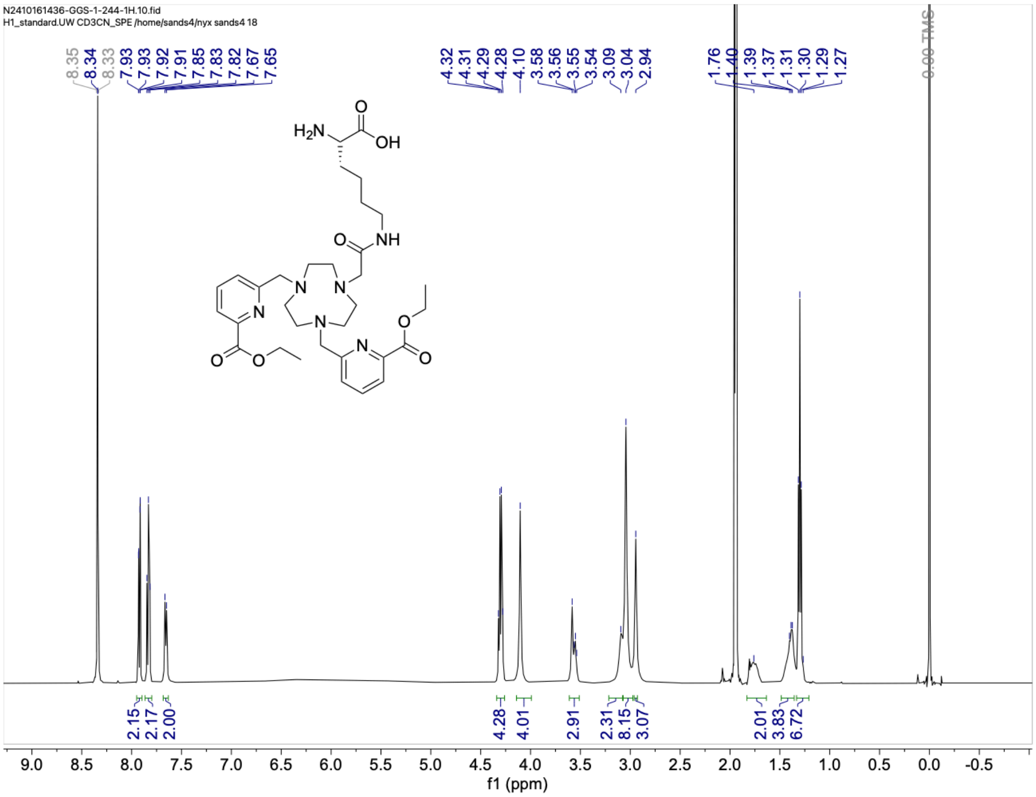


**Figure S126:** $^{1}$H NMR of **7** (peak at 8.34 is formic acid)

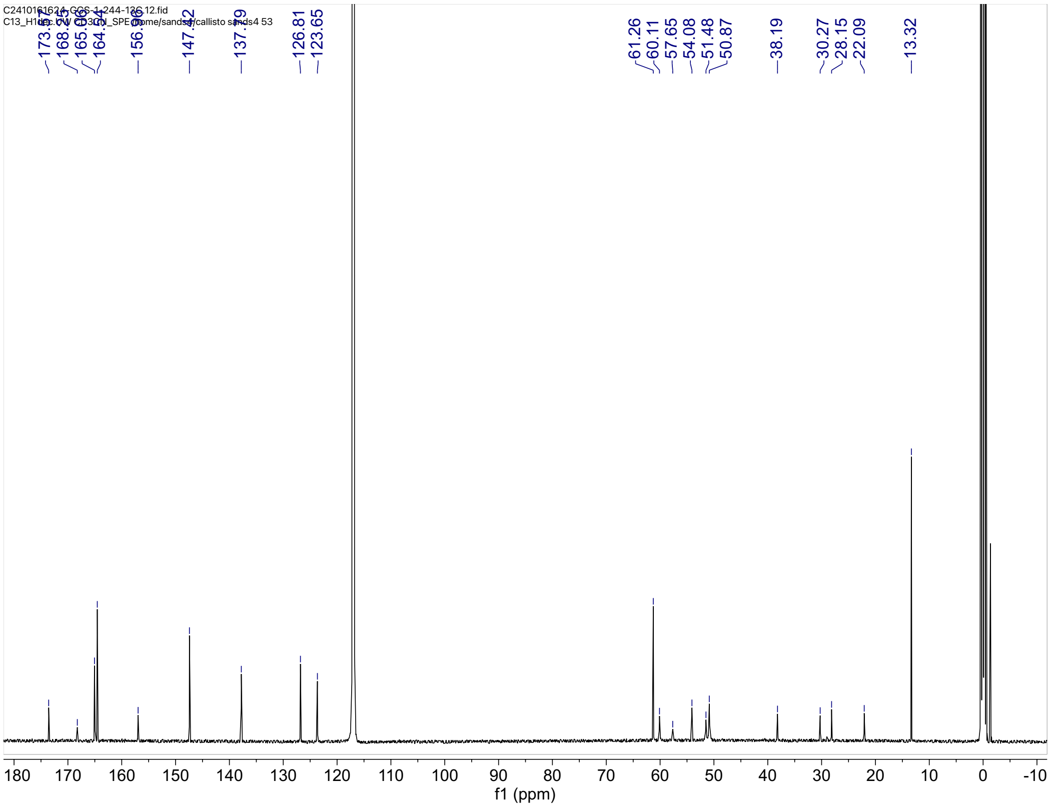


**Figure S127:** $^{13}$C NMR of **7**

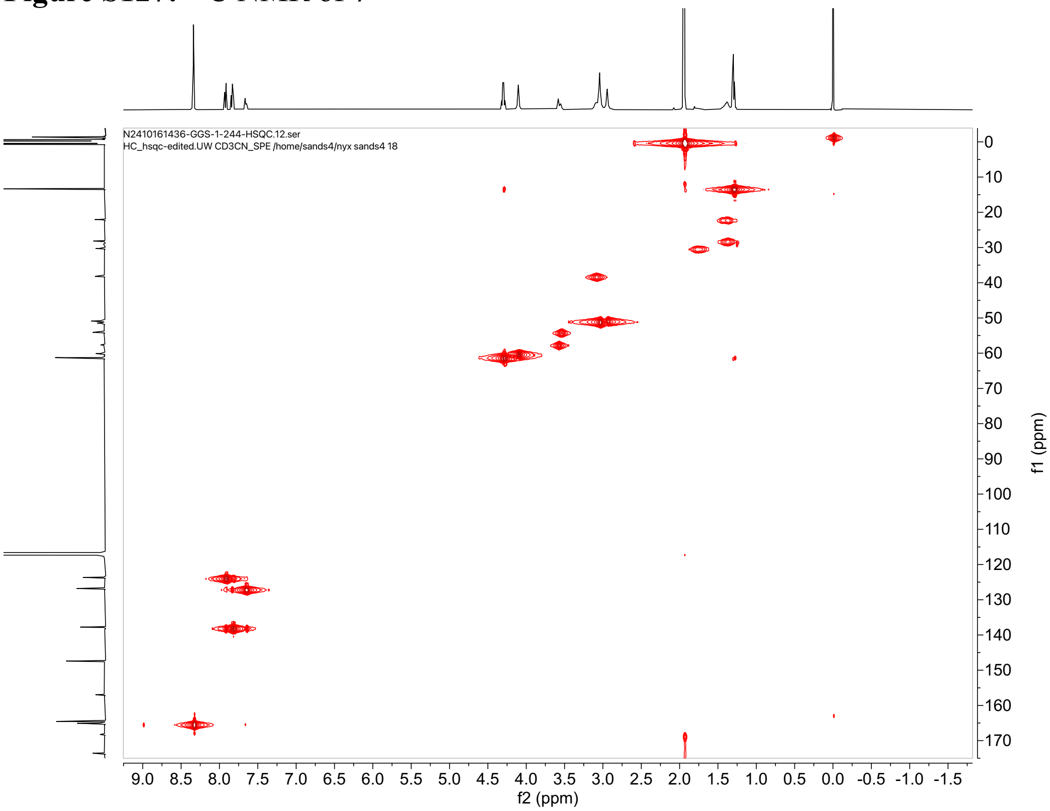


**Figure S128:** HSQC of **7**

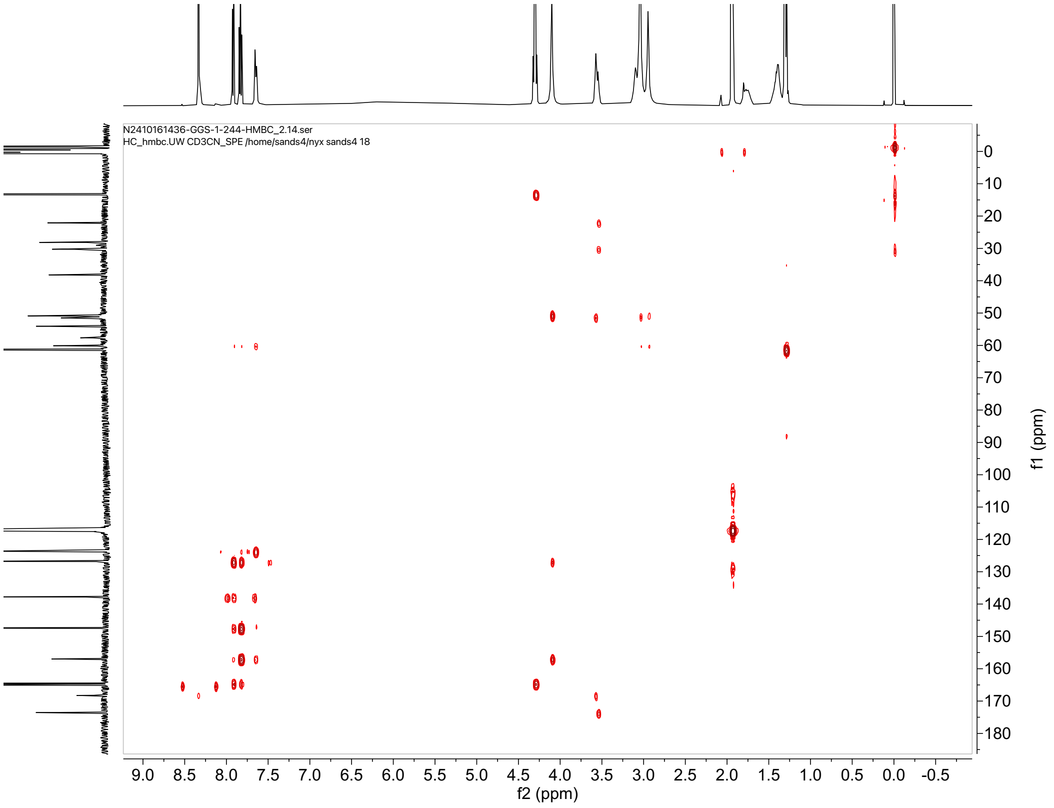


**Figure S129:** HMBC of **7**

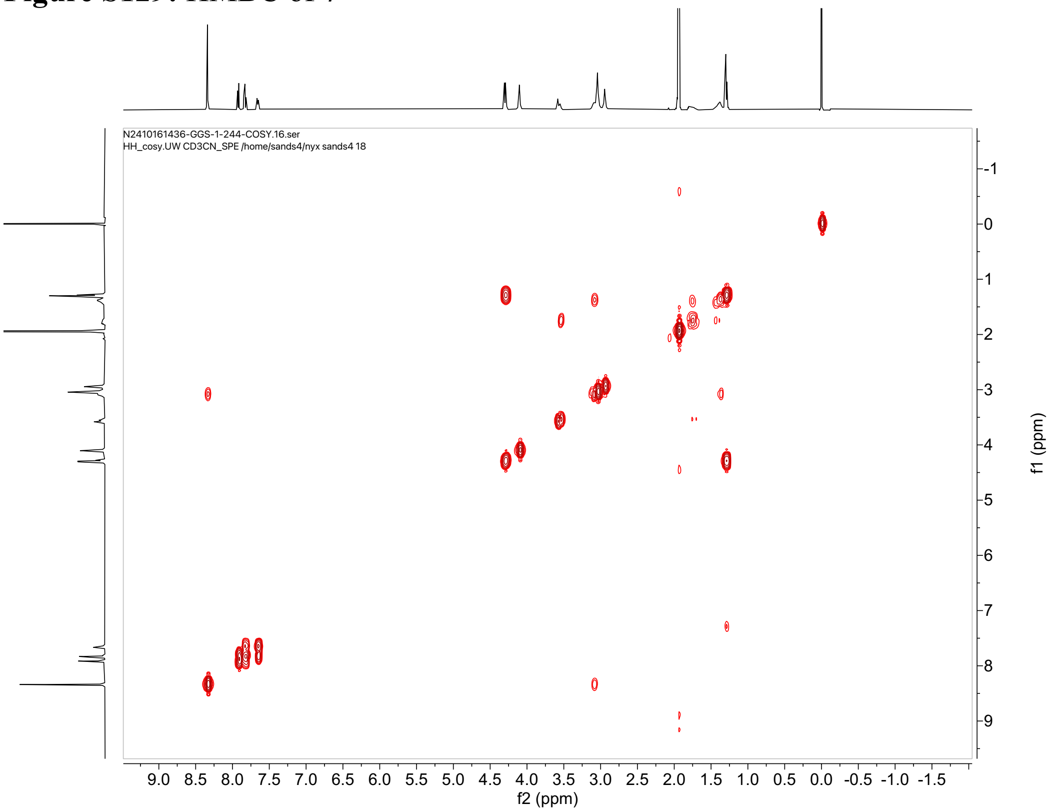


**Figure S130:** COSY of **7**

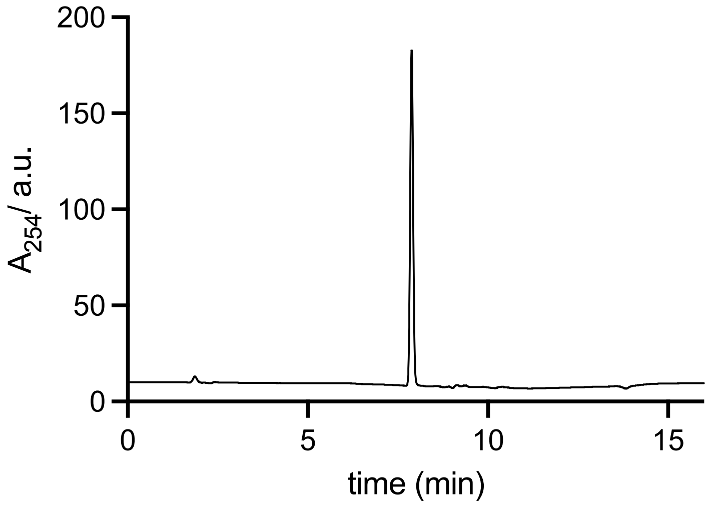


**Figure S131:** HPLC trace of **7** ($R_t$ = 7.89 min) (Method D).

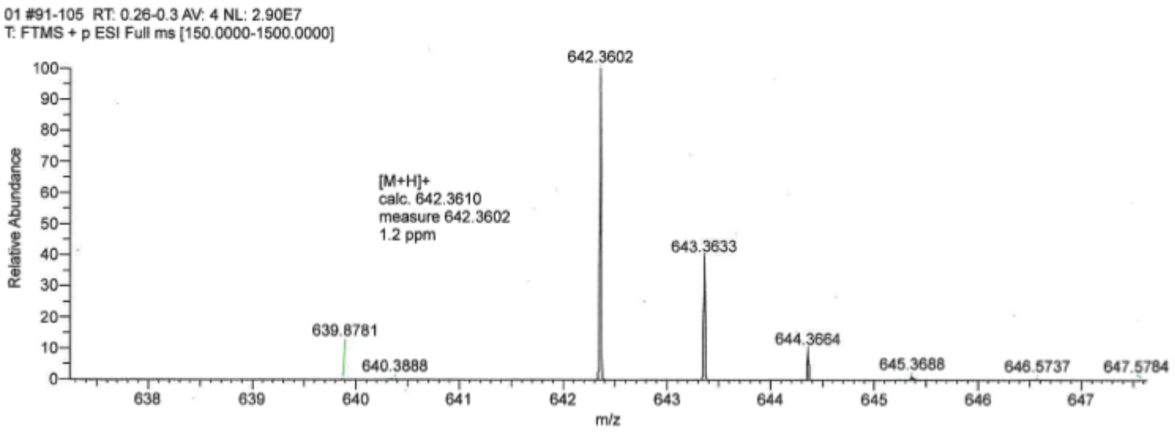


**Figure S132:** HR-ESI MS of **7**

**S8**

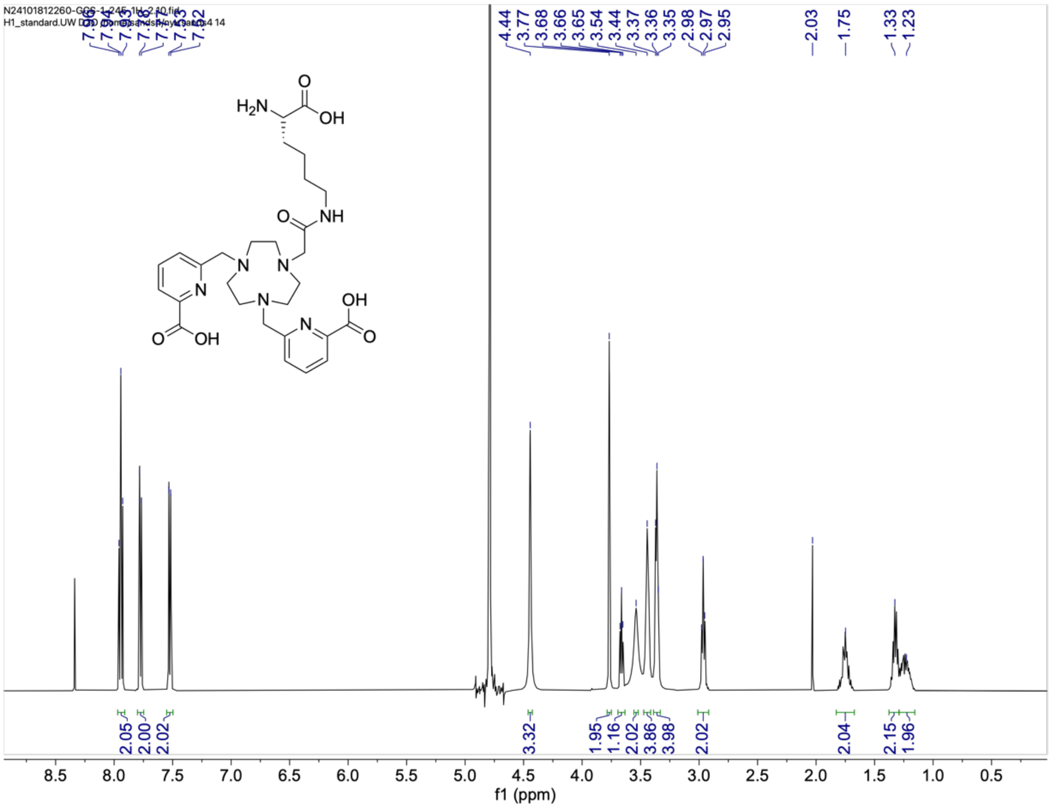


**Figure S133:** $^{1}$H NMR of **8** (peak at 8.35 is formic acid)

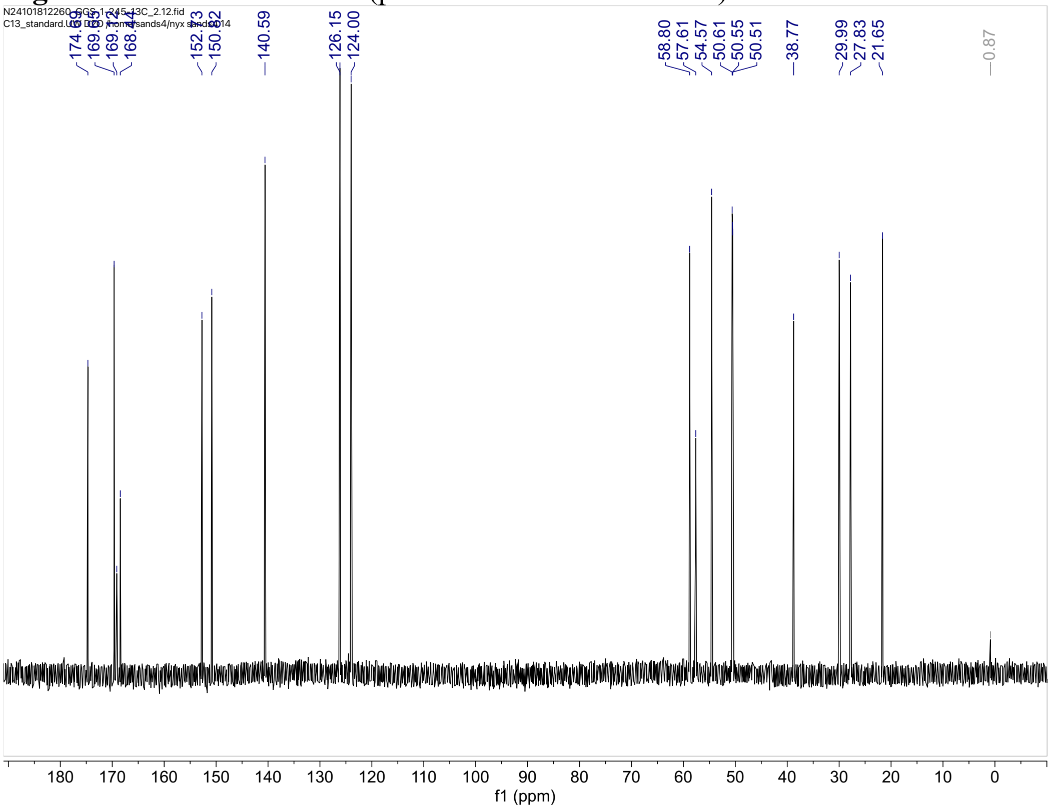


**Figure S134:** $^{13}$C NMR of **8**

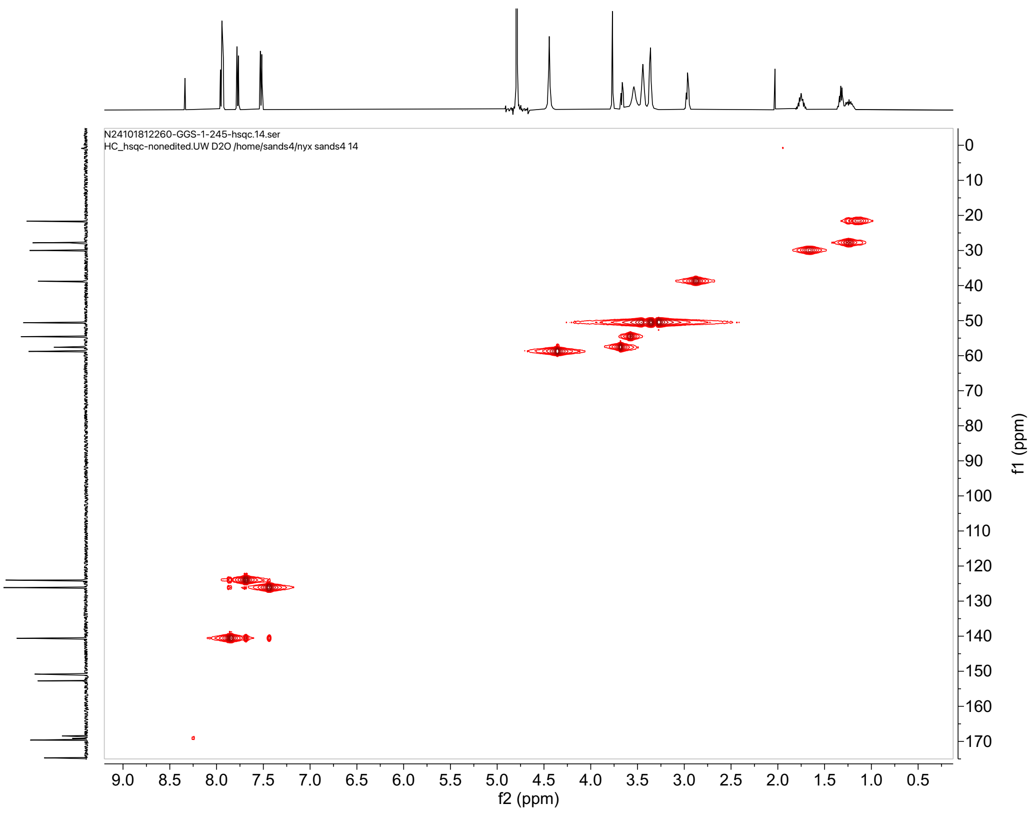


**Figure S135:** HSQC of **8**

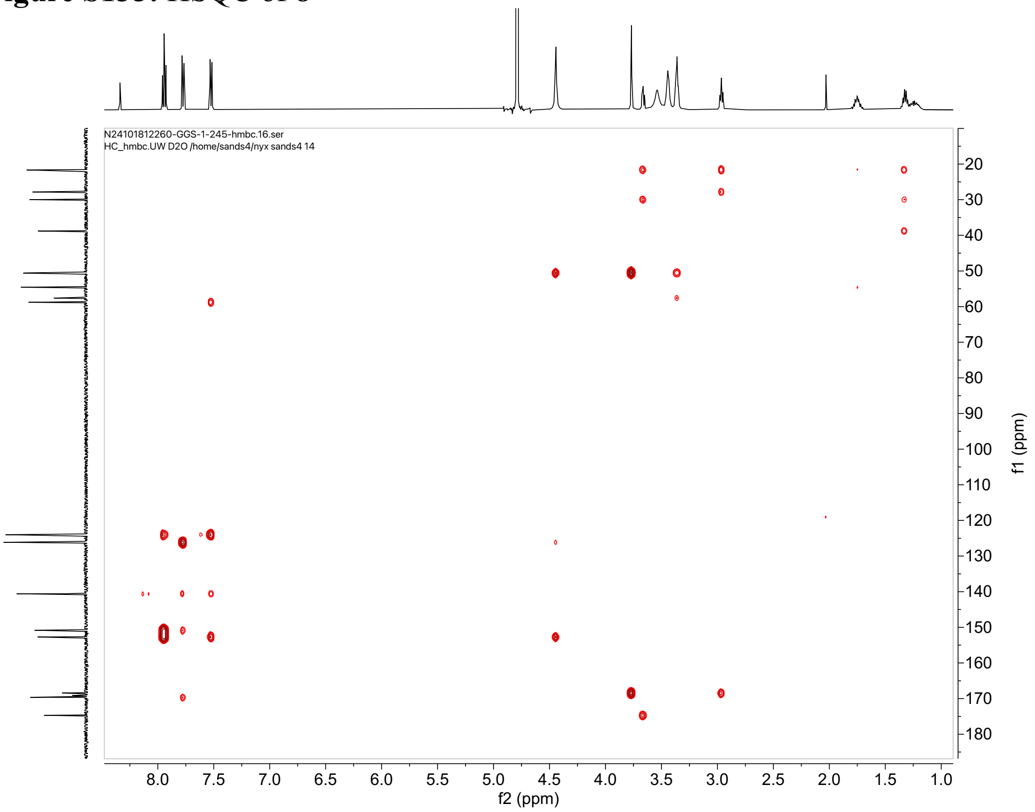


**Figure S136:** HMBC of **8**

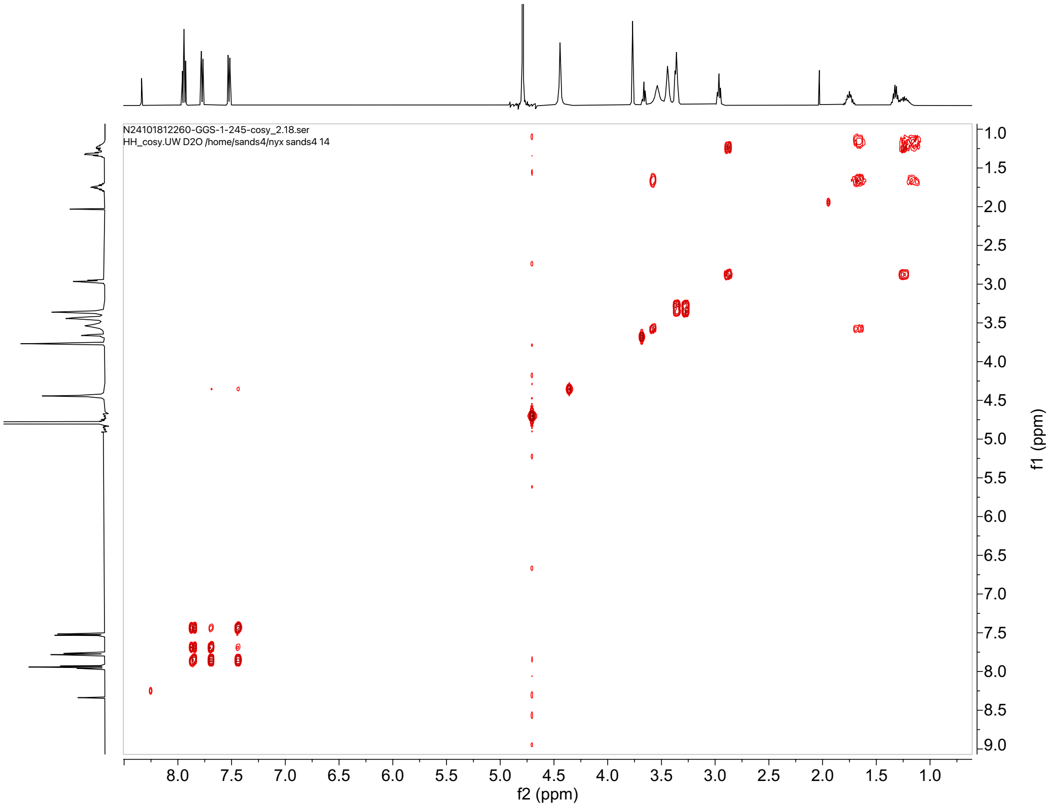


**Figure S137:** COSY of **8**

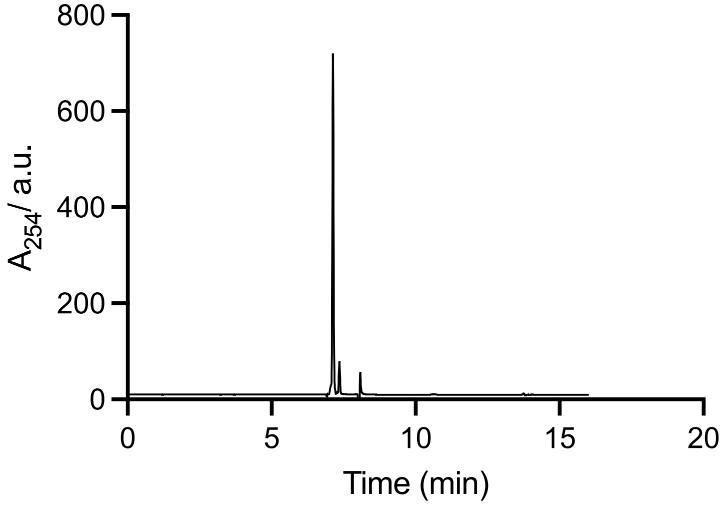


**Figure S138:** HPLC trace of **8** ($R_t$ = 7.13 mins) (Method F).

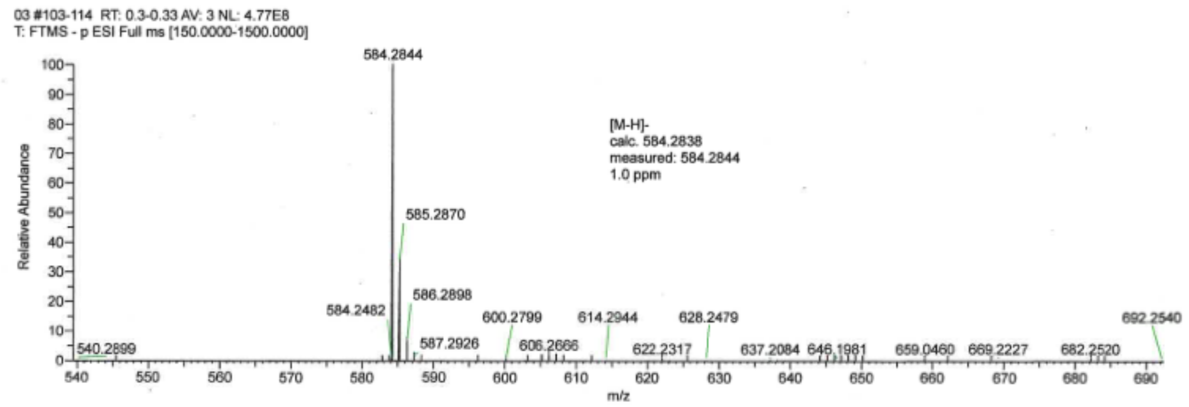


**Figure S139:** HR-ESI MS of **8**

**[Tb(bispic-lysine)]⁺**

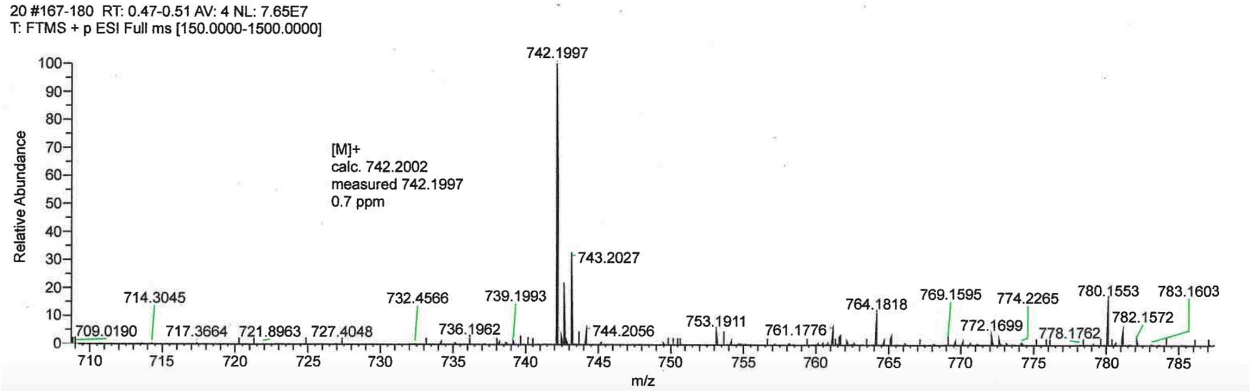


**Figure S140:** HR-ESI MS of **[Tb(bispic-lysine)]$^+$**

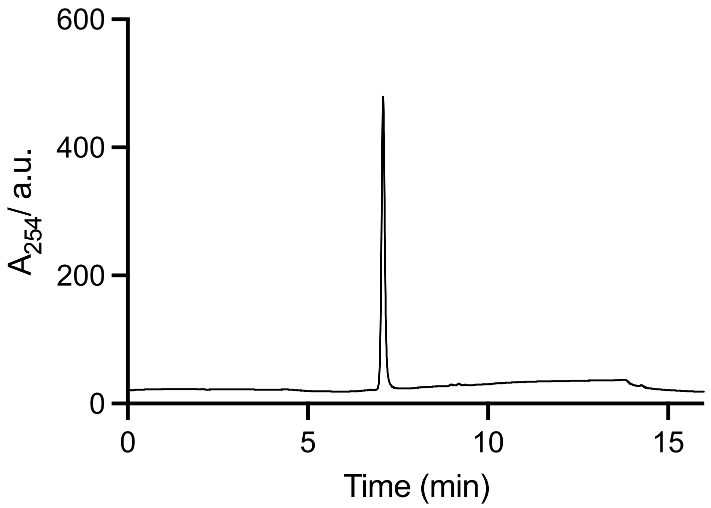


**Figure S141:** HPLC trace of **[Tb(bispic-lysine)]$^+$** ($R_t$= 7.10 min) (method F).

**[Eu(bispic-lysine)]$^+$**

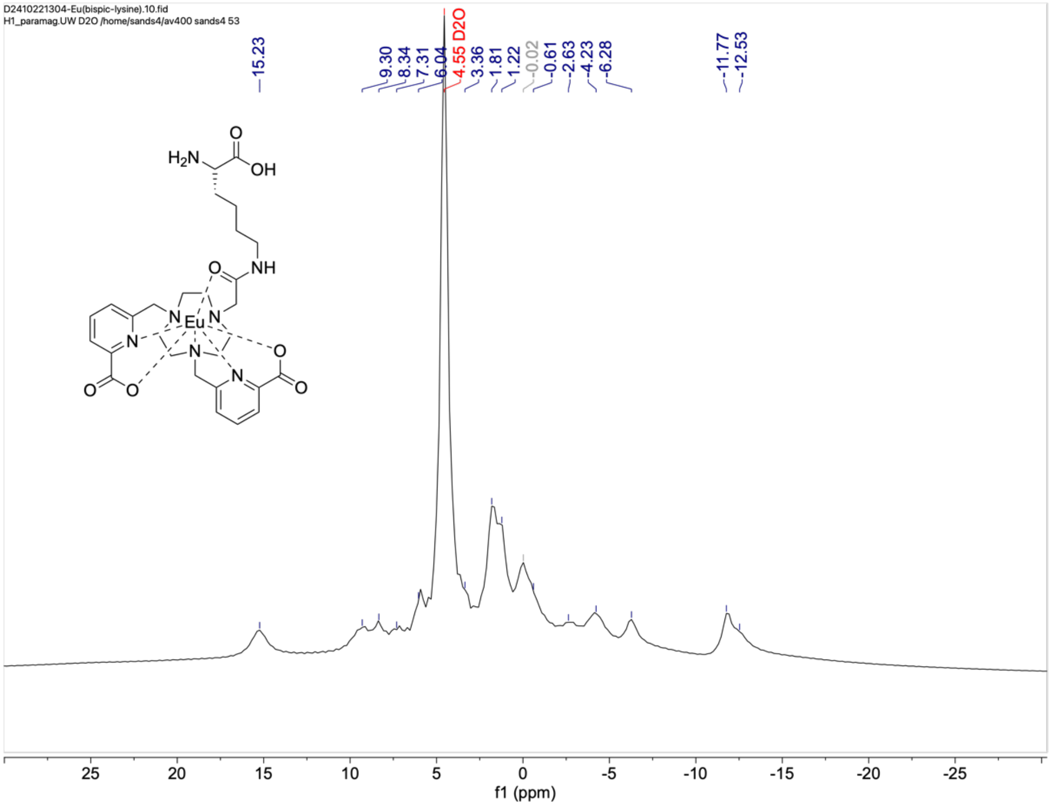


**Figure S142:** $^1$H NMR of **[Eu(bispic-lysine)]$^+$**

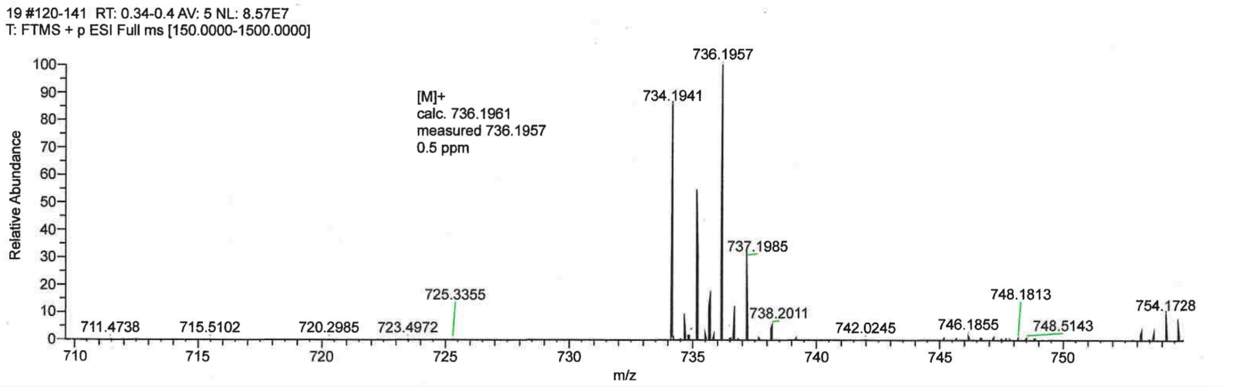


**Figure S143:** HR-ESI MS of **[Eu(bispic-lysine)]$^+$**

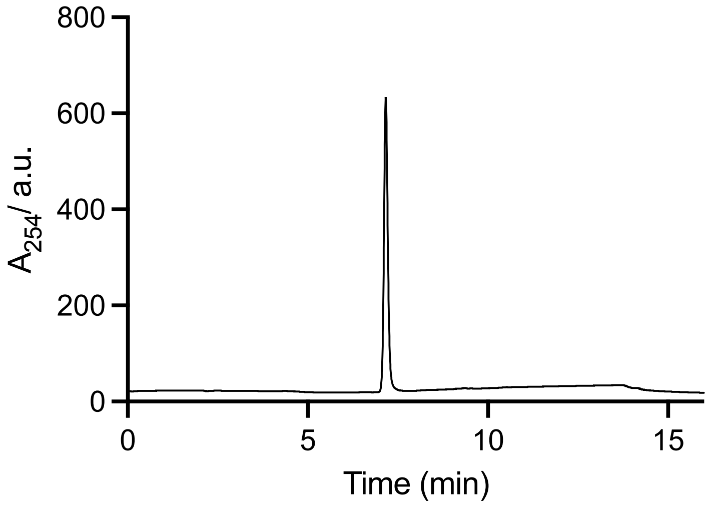


**Figure S144:** HPLC trace of **[Eu(bispic-lysine)]**$^{+}$ ($R_t$ = 7.17 min) (method F).

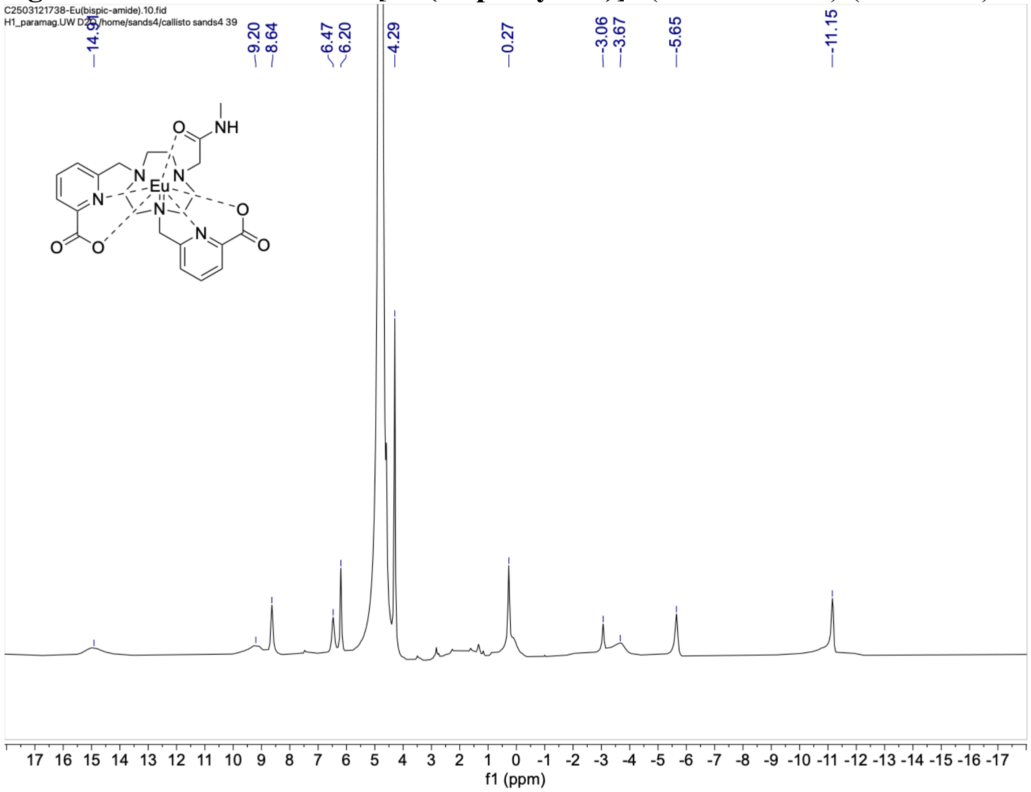


**Figure S145:** $^{1}$H NMR of **[Eu(bispic-amide)]**$^{+}$

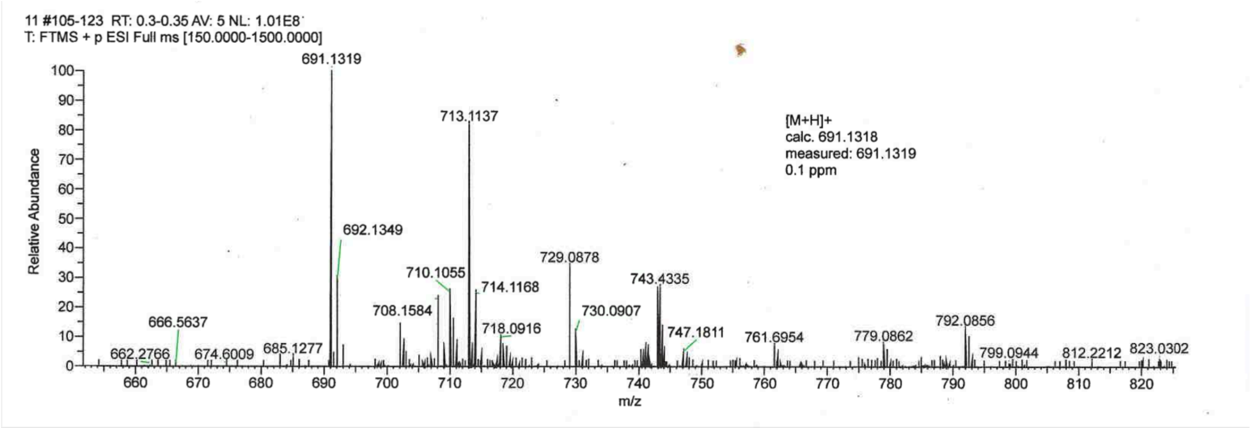


**Figure S146:** HR-ESI MS **[Tb(trispic)]**

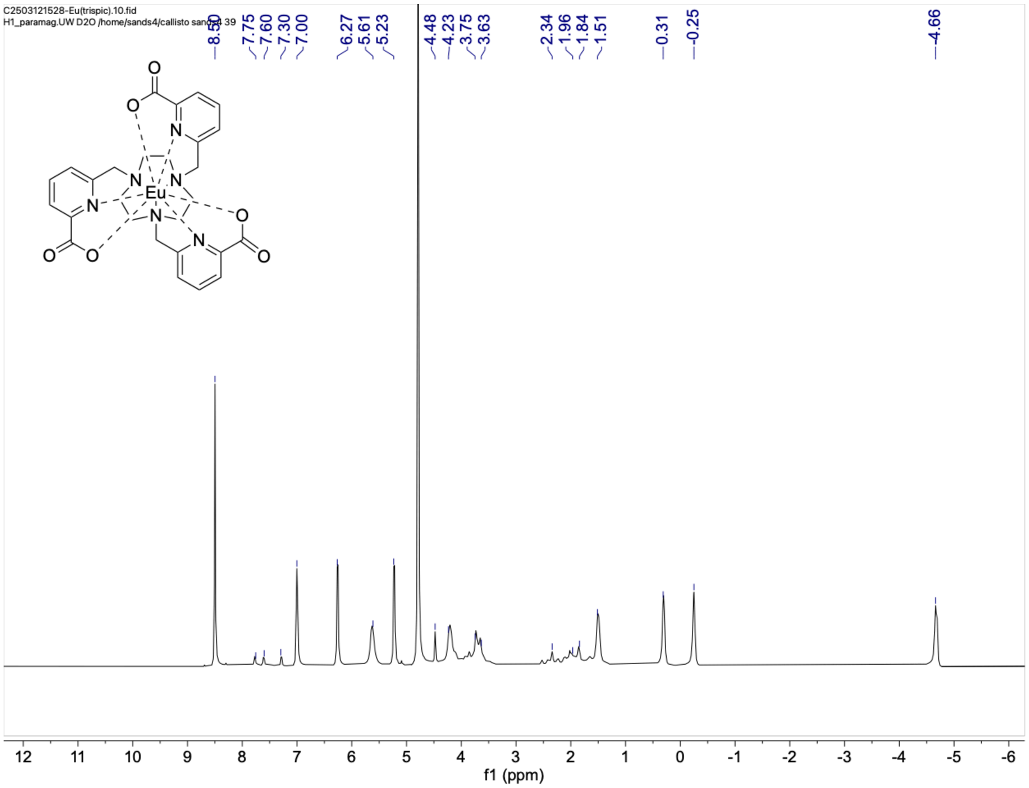


**Figure S147:** $^{1}$H NMR of **[Eu(trispic)]**

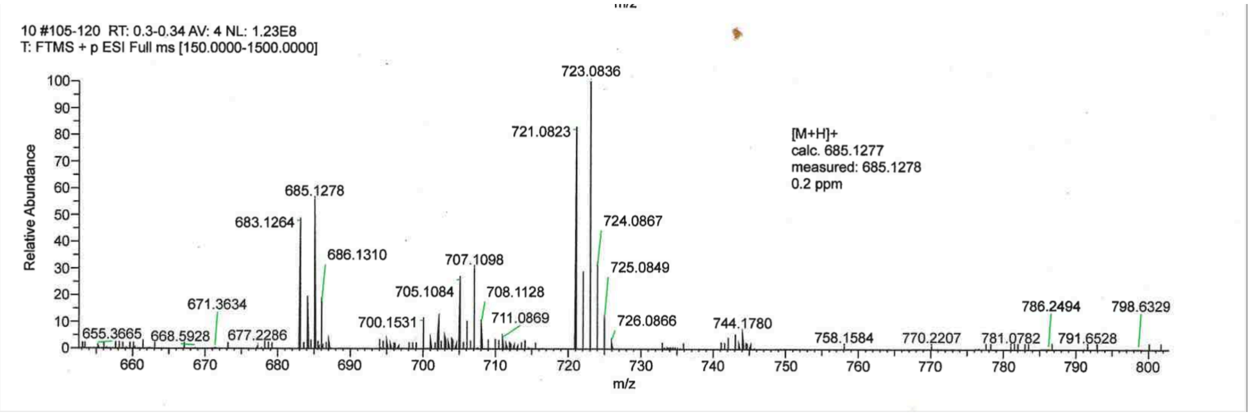


**Figure S148:** HR-ESI MS **[Eu(trispic)]**

## [Eu(bispic)]$^+$

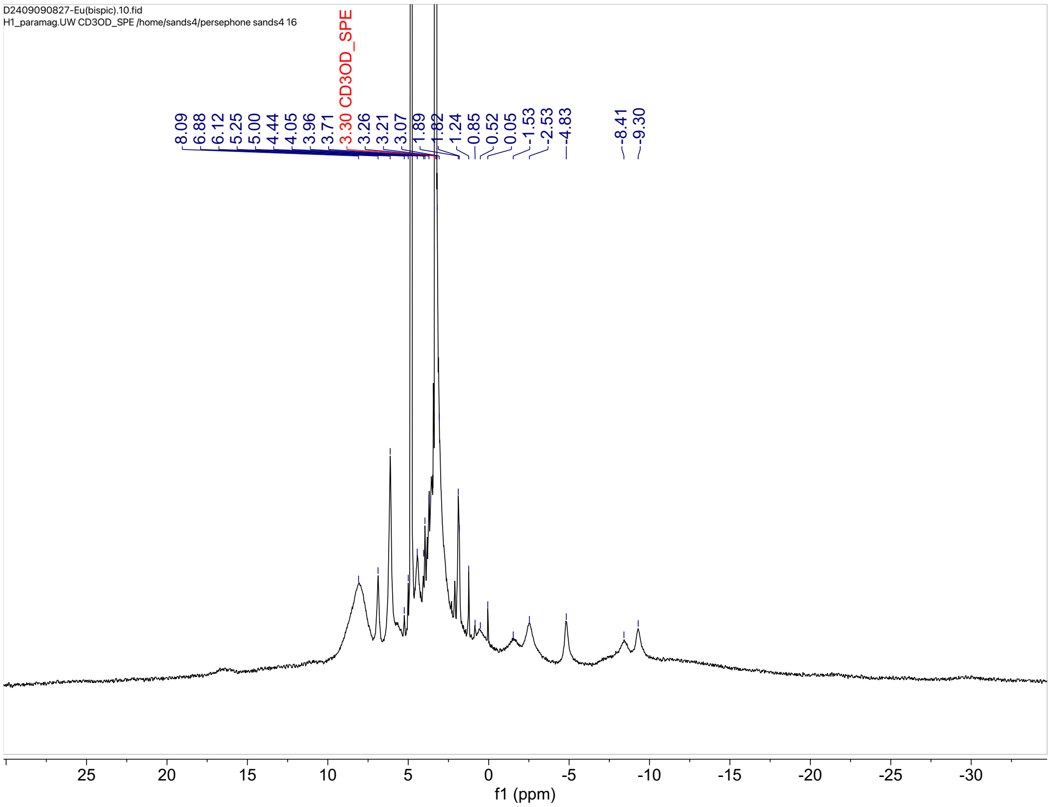


**Figure S149:** $^1$H NMR of **[Eu(bispic)]**$^+$

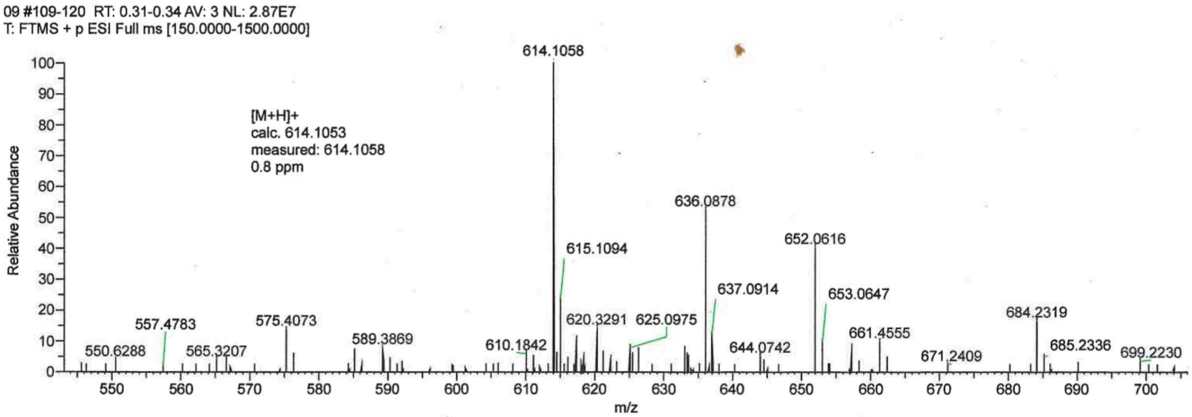


**Figure S150:** HR-ESI MS of **[Tb(bispic-acetate)]**

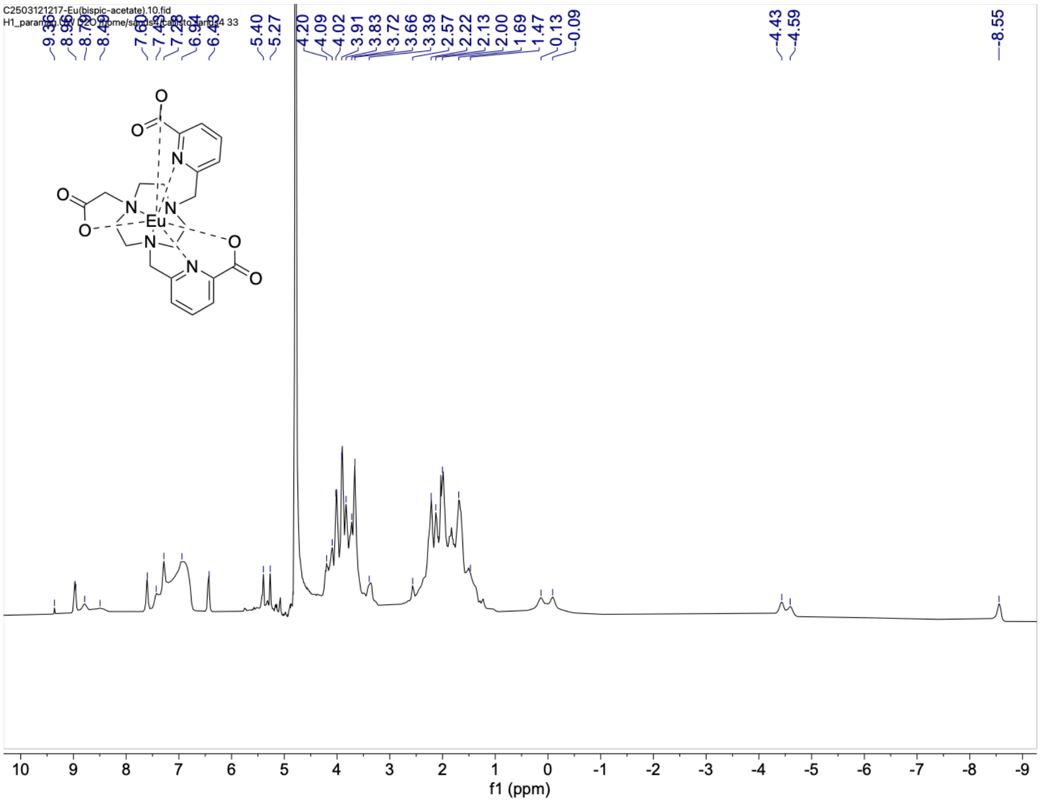


**Figure S151:** $^{1}$H NMR of **[Eu(bispic-acetate)]**

**Synthesis of $[Ln(bispic\text{-}Ser)]^+$**

S10

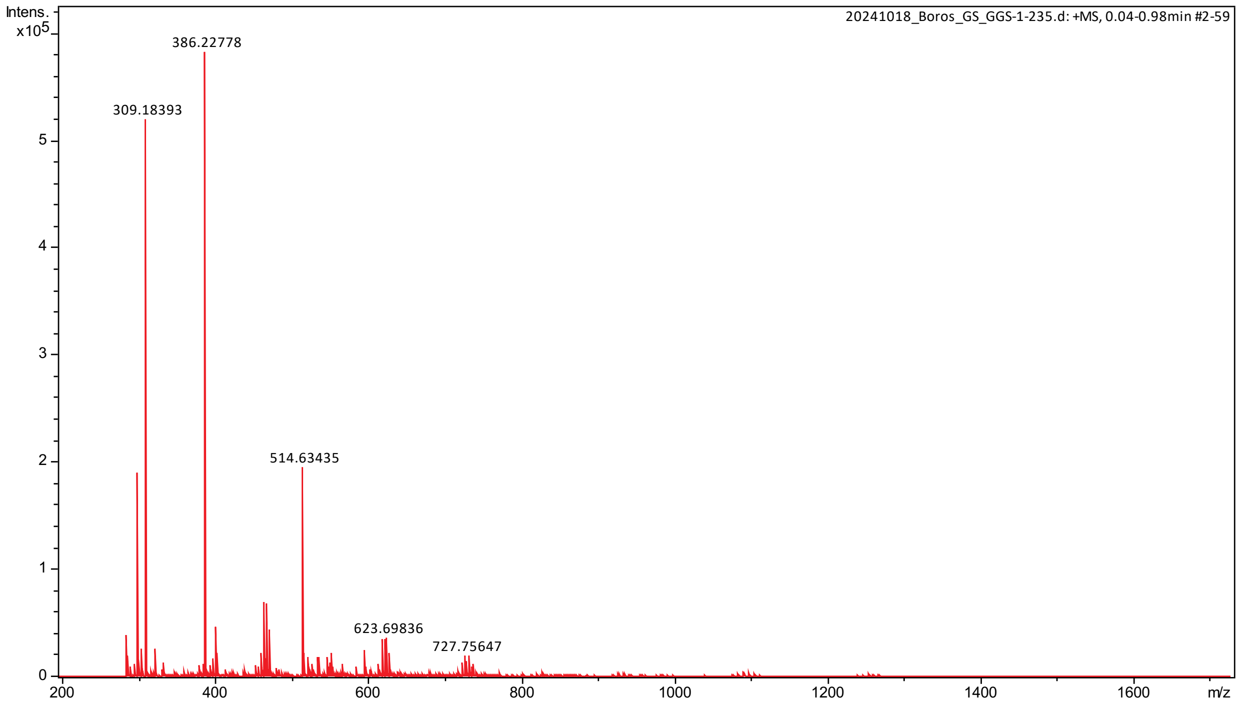


**Figure S152:** HR-ESI MS of **10**

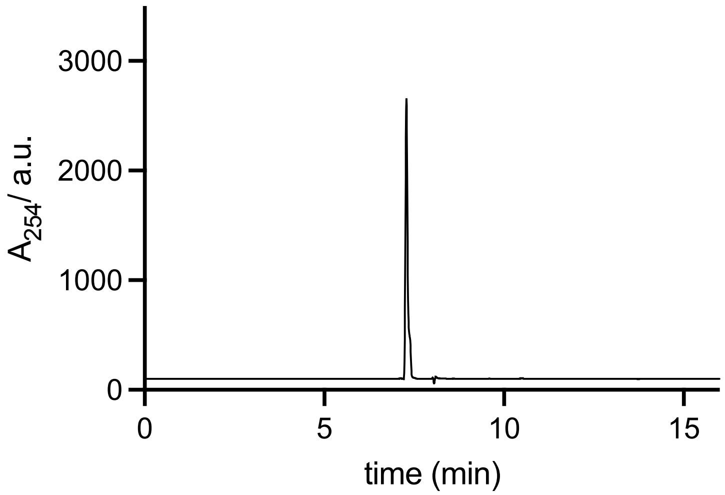


**Figure S153:** HPLC trace of **10** ($R_t$ = 7.38 min) (method F).

**S11**

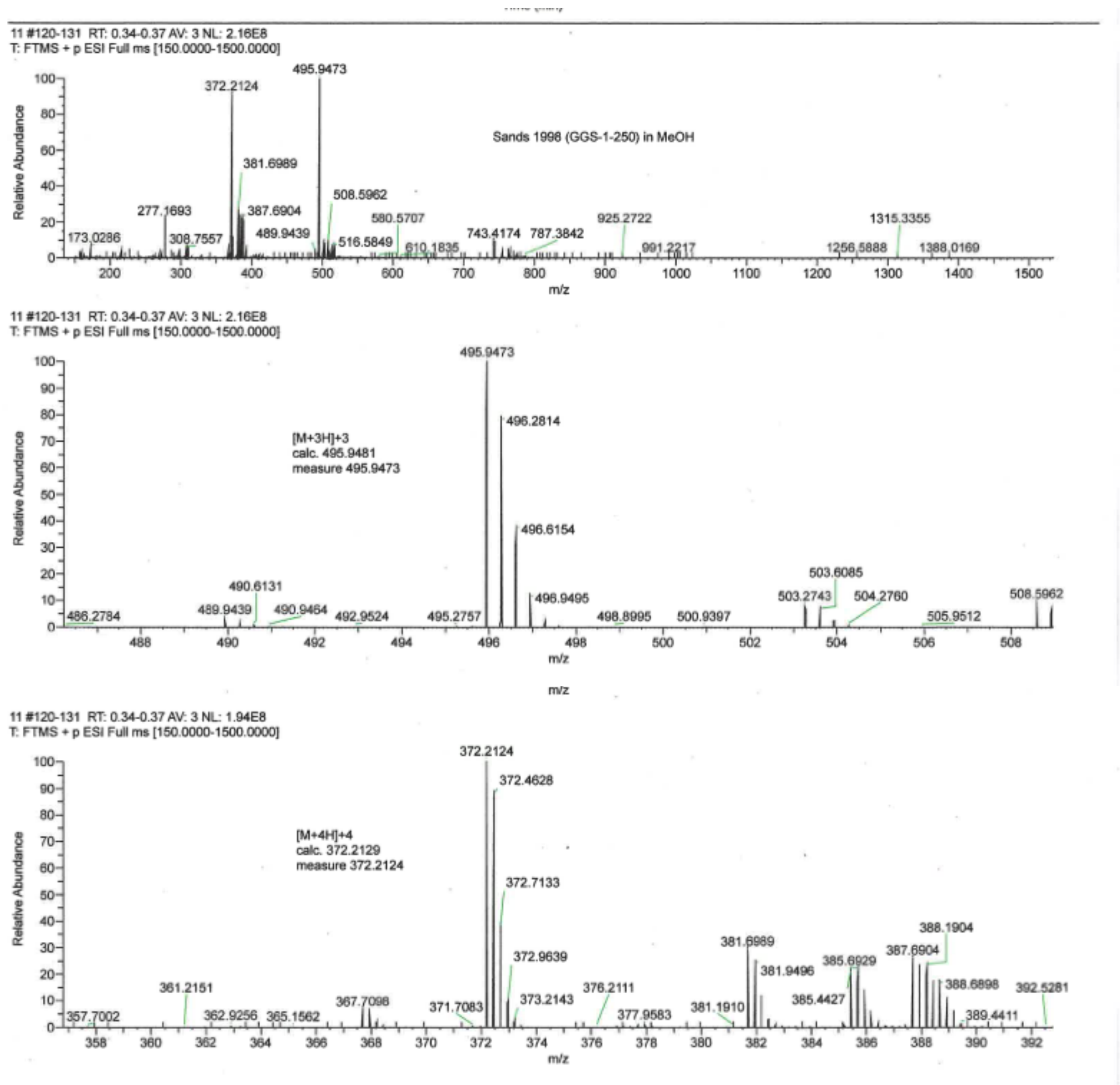


**Figure S154:** HR-ESI MS of **11**

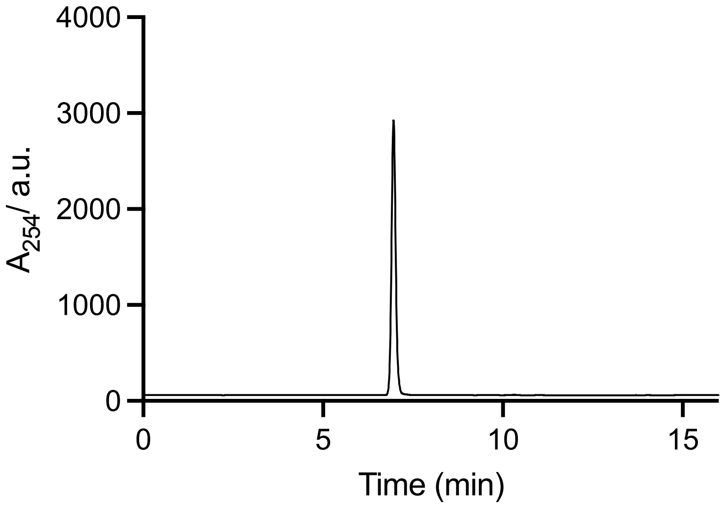


**Figure S155:** HPLC trace of **11** ($R_t$ =6.97 min) (method F).

**[Eu(bispic-Ser)]$^+$**

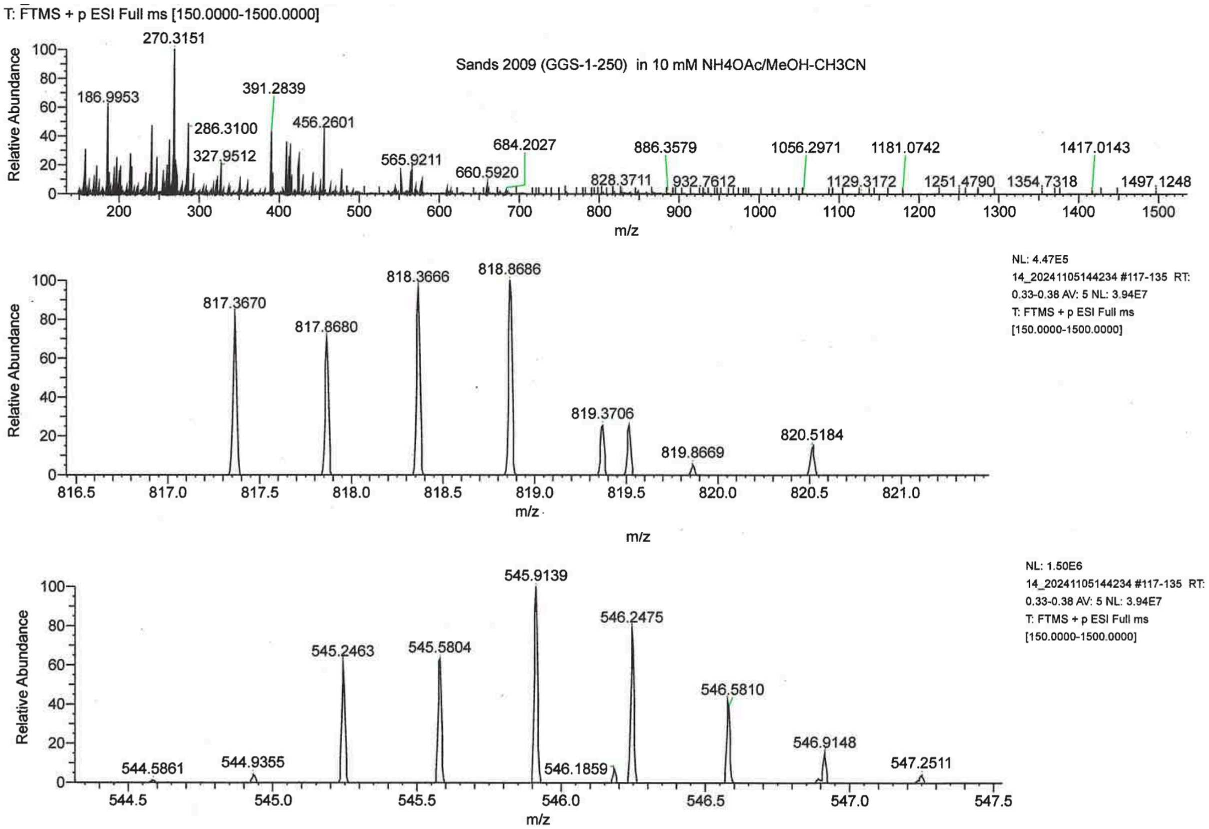


**Figure S156:** HR-ESI MS of [Eu(bispic-Ser)]$^+$

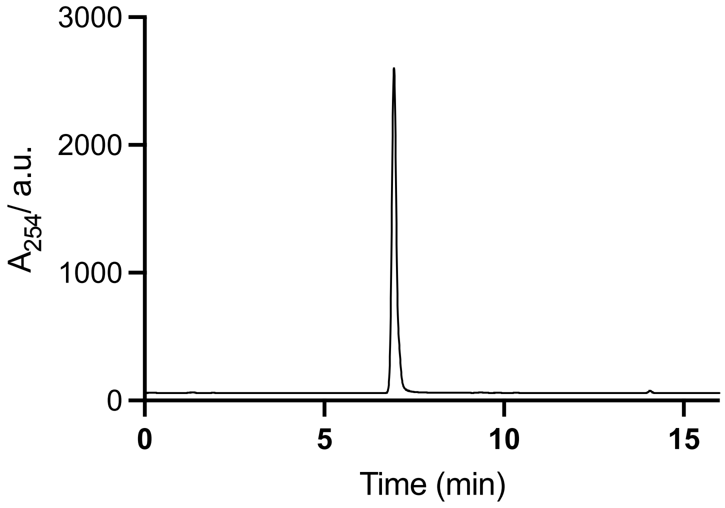


**Figure S157:** HPLC trace of $[Eu(bispic\text{-}Ser)]^{+}$ ($R_t = 6.93$ min) (method F)

**$[Tb(bispic\text{-}Ser)]^{+}$**

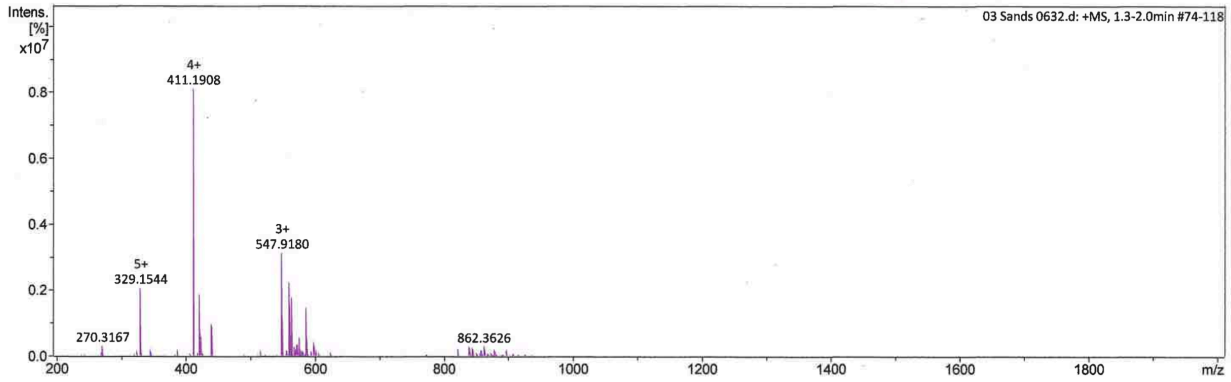


**Figure S158:** HR-ESI of $[Tb(bispic\text{-}Ser]^{+}$

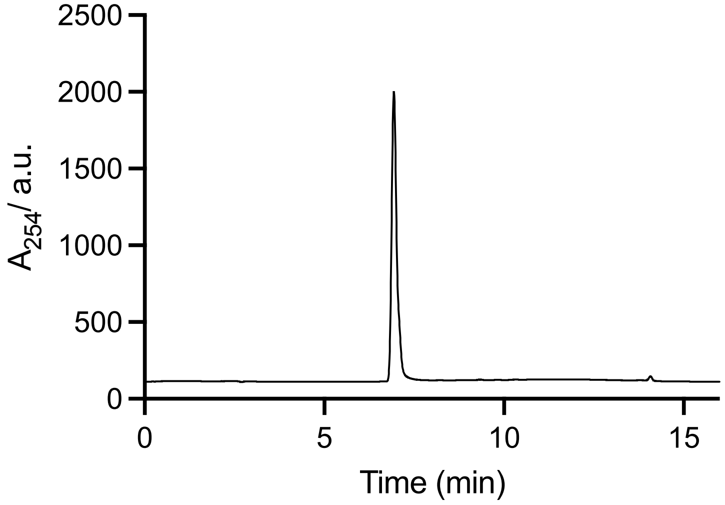


**Figure S159:** HPLC trace of $[Tb(bispic\text{-}Ser)]^{+}$ ($R_t = 6.93$ min) (method F)

**Synthesis of [Ln(bispic-Peptides)]⁻**

**S12**

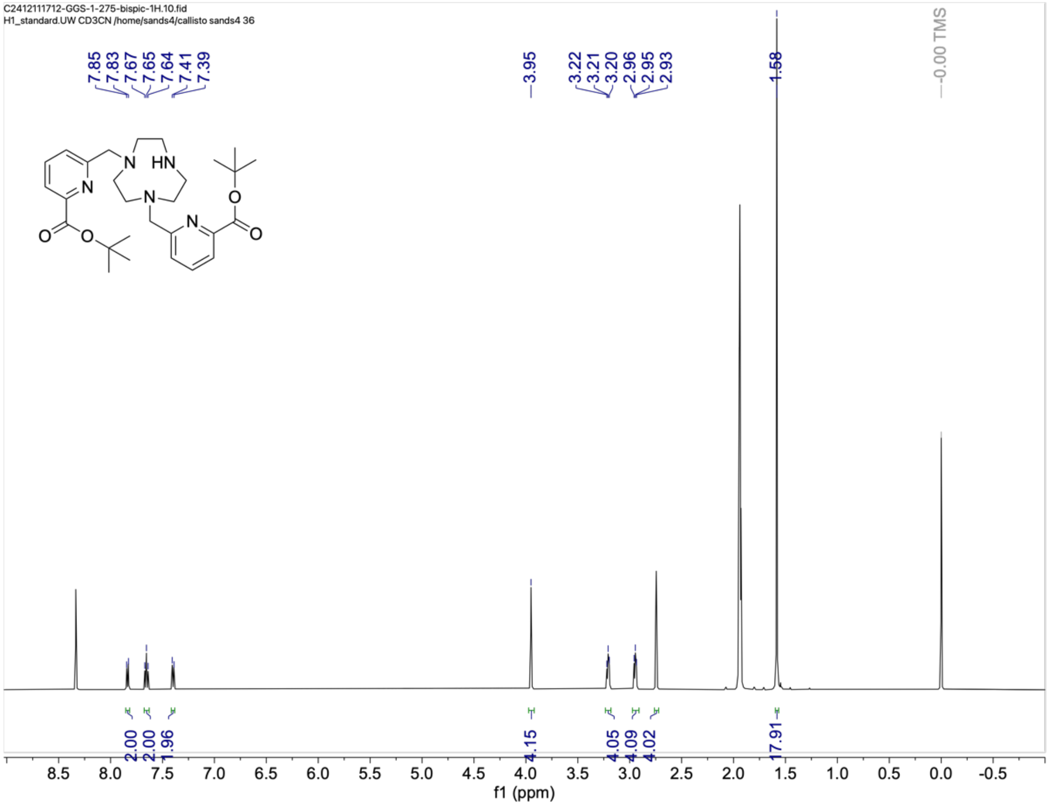


**Figure S160:** $^{1}$H NMR of **12** (peak at 8.35 is formic acid)

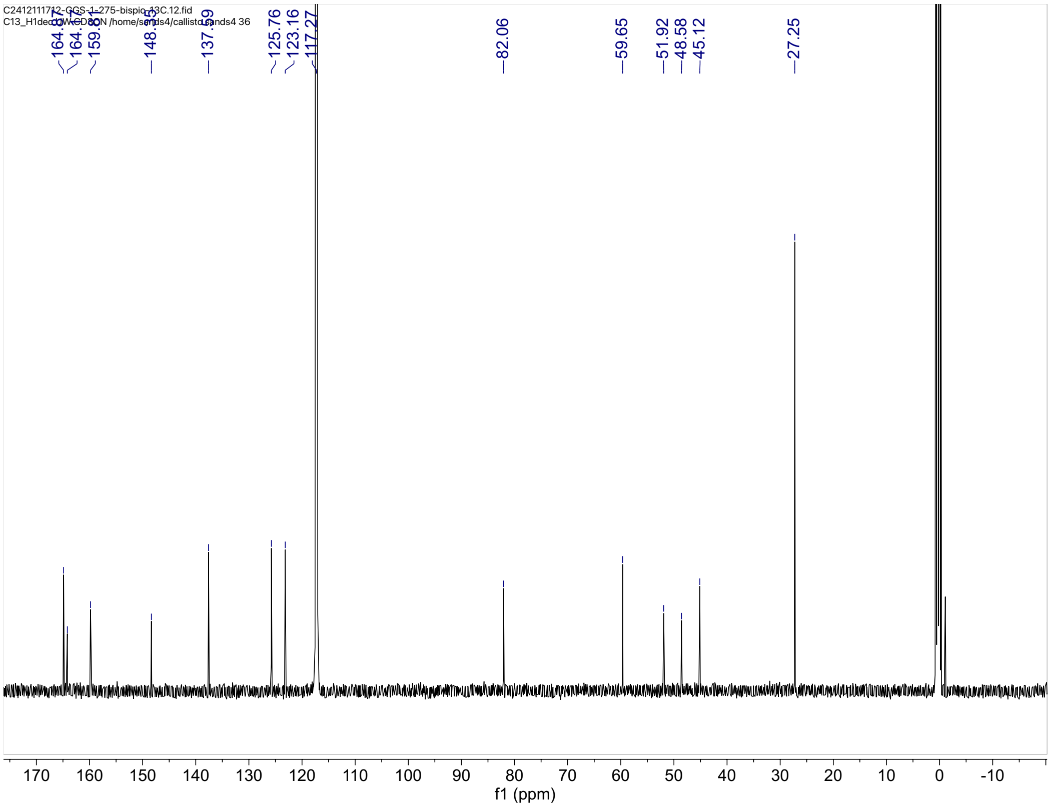


**Figure S161:** $^{13}$C NMR of **12**

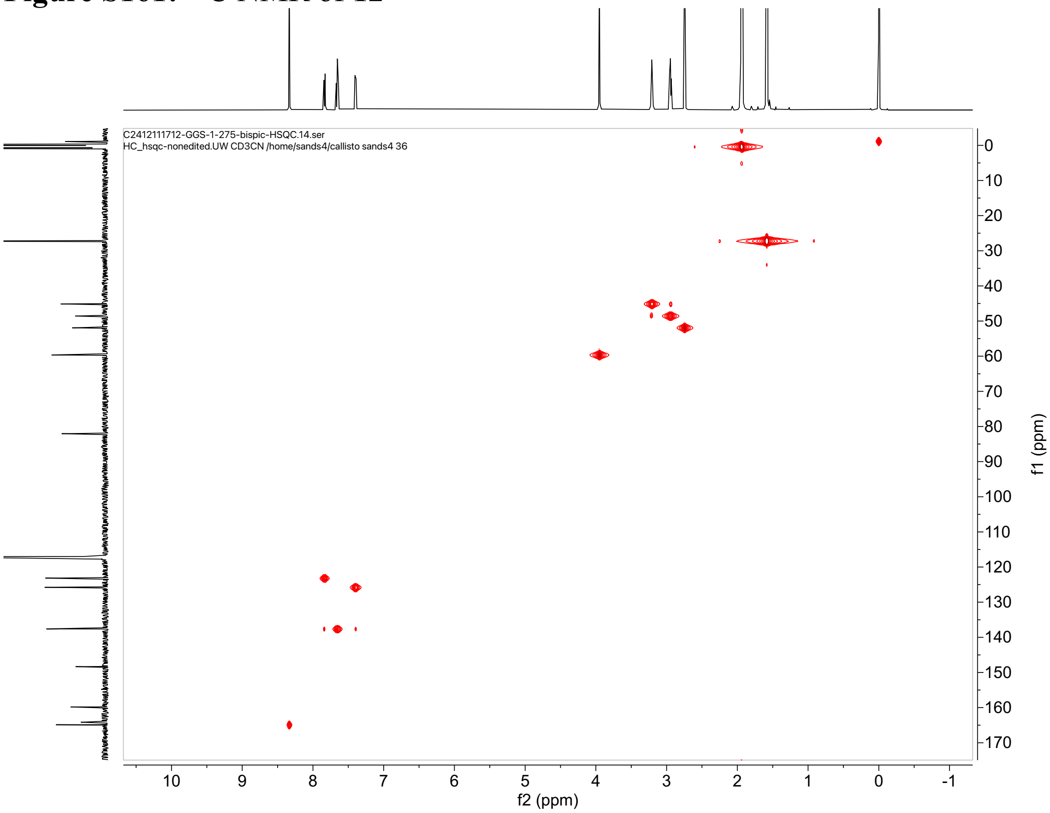


**Figure S162:** HSQC of **12**

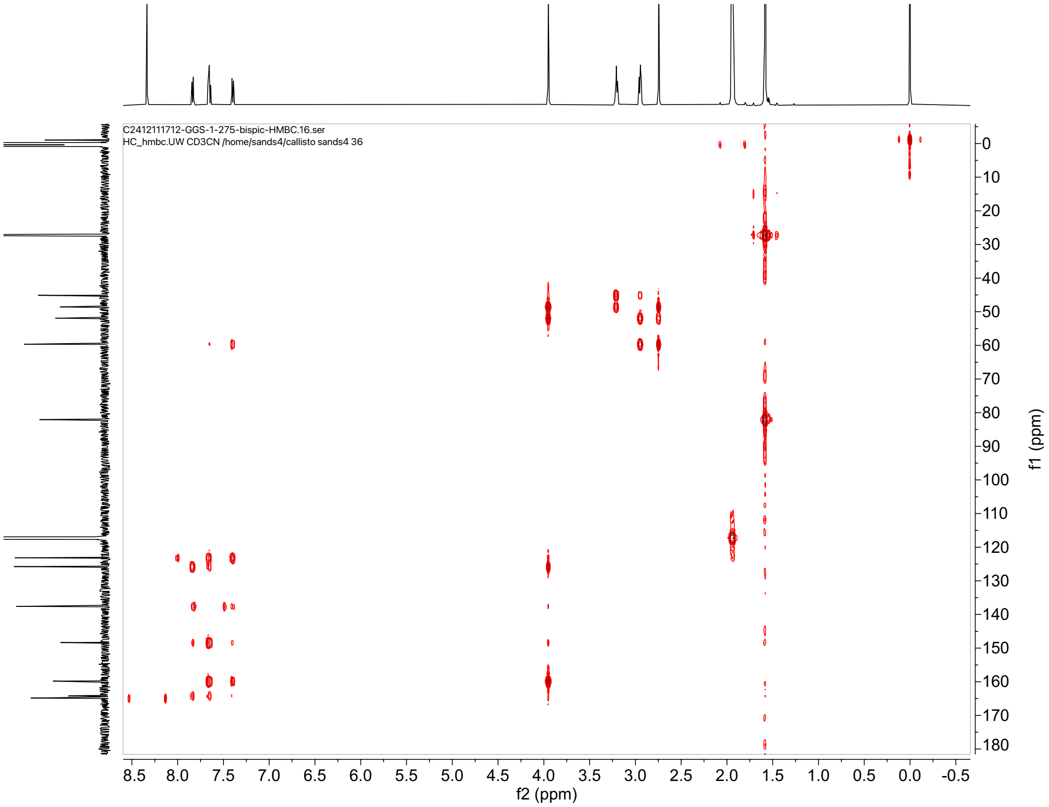


**Figure S163:** HMBC of **12**

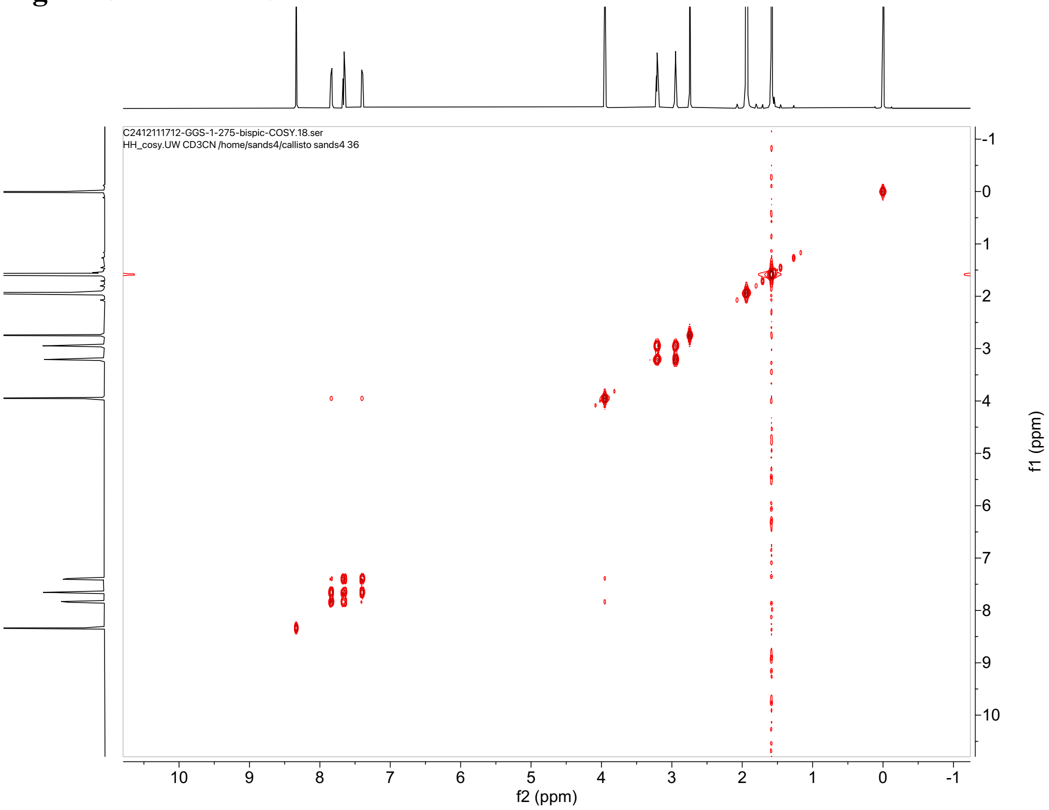


**Figure S164:** COSY of **12**

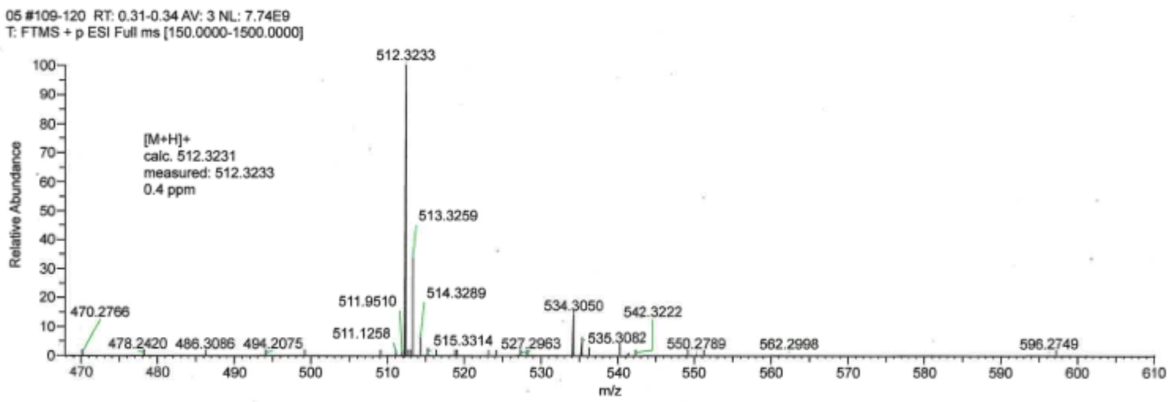


**Figure S165:** HR-ESI MS of **12**

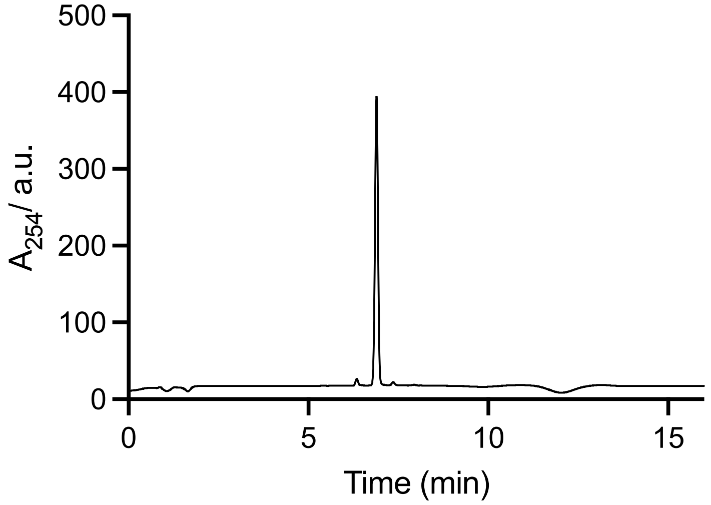


**Figure S166:** Analytical trace of **12** ($R_t$ = 6.90 min) (method E)

## S13

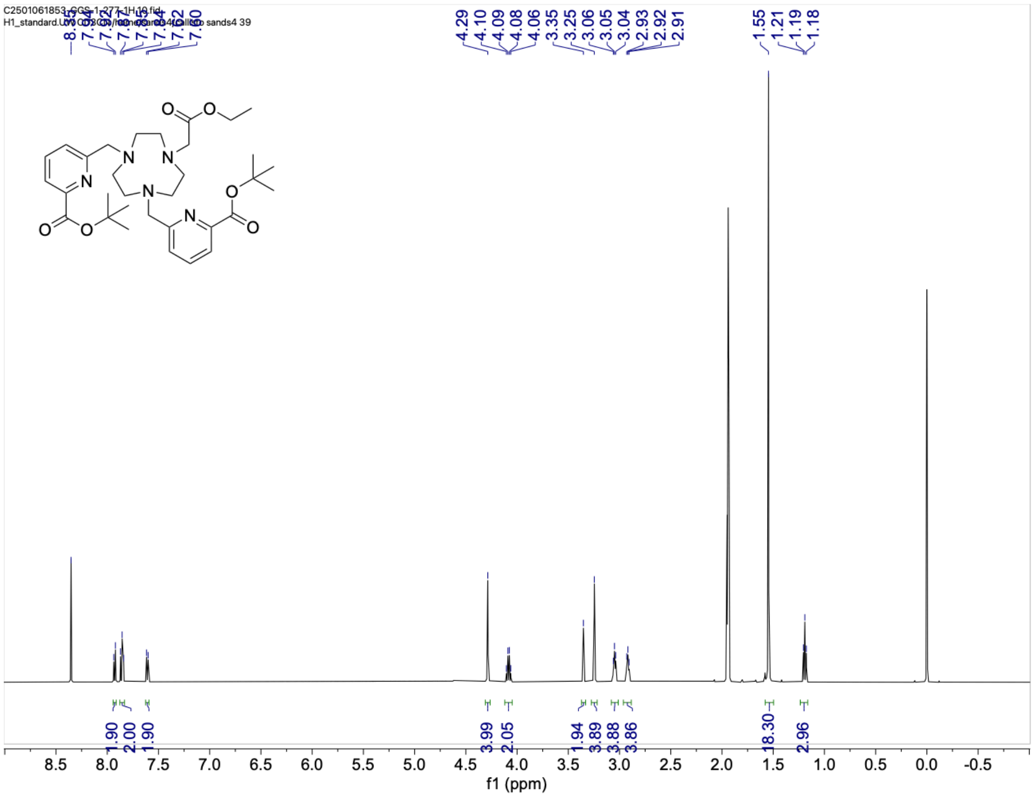


**Figure S167:** $^{1}$H NMR of **13** (peak at 8.35 is formic acid)

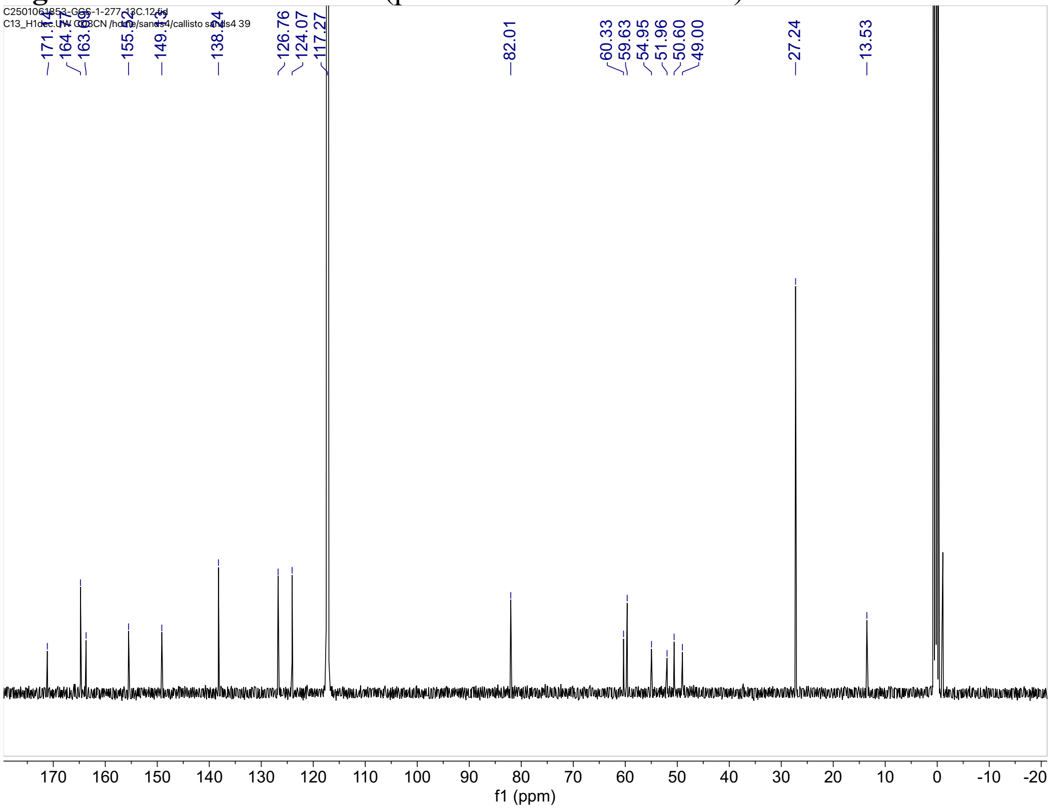


**Figure S168:** $^{13}$C NMR of **13**

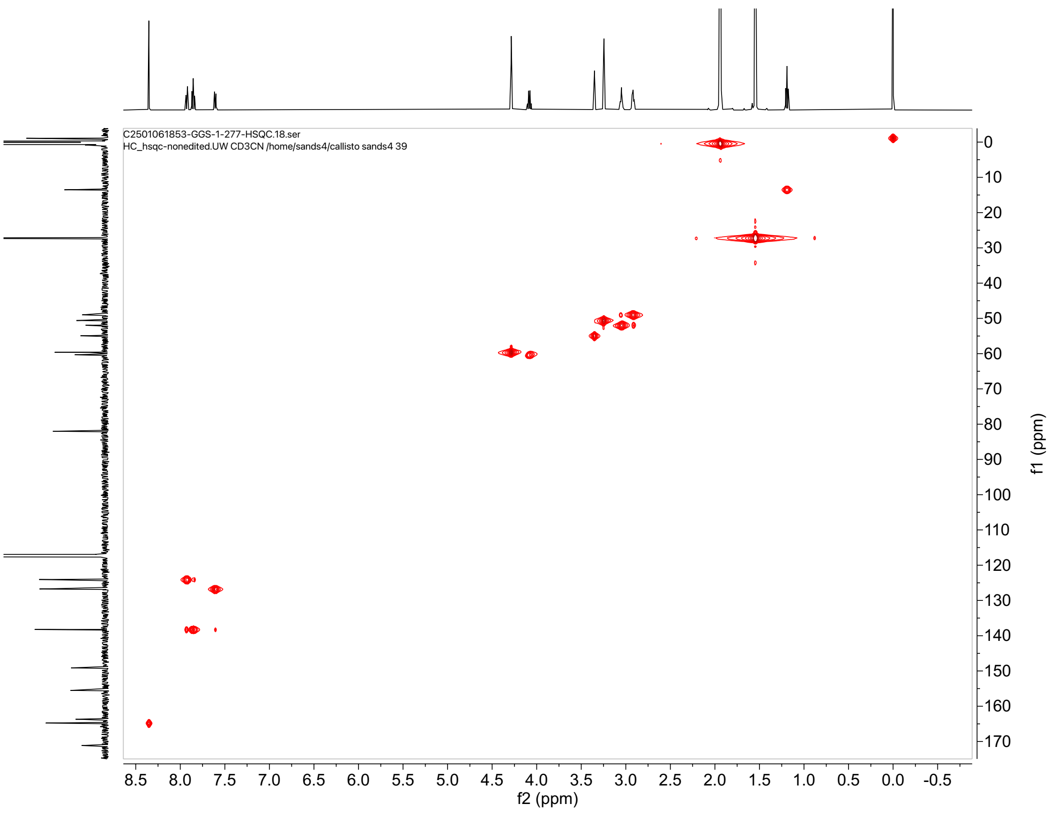


**Figure S169:** HSQC of **13**

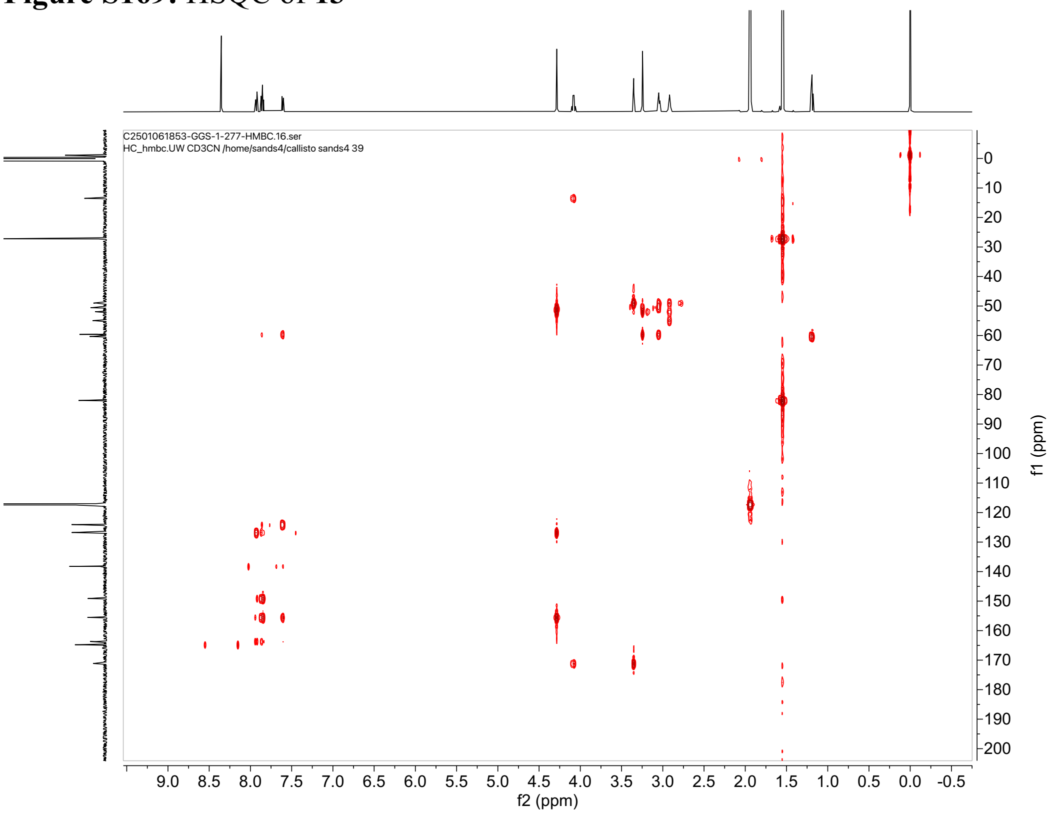


**Figure S170:** HMBC of **13**

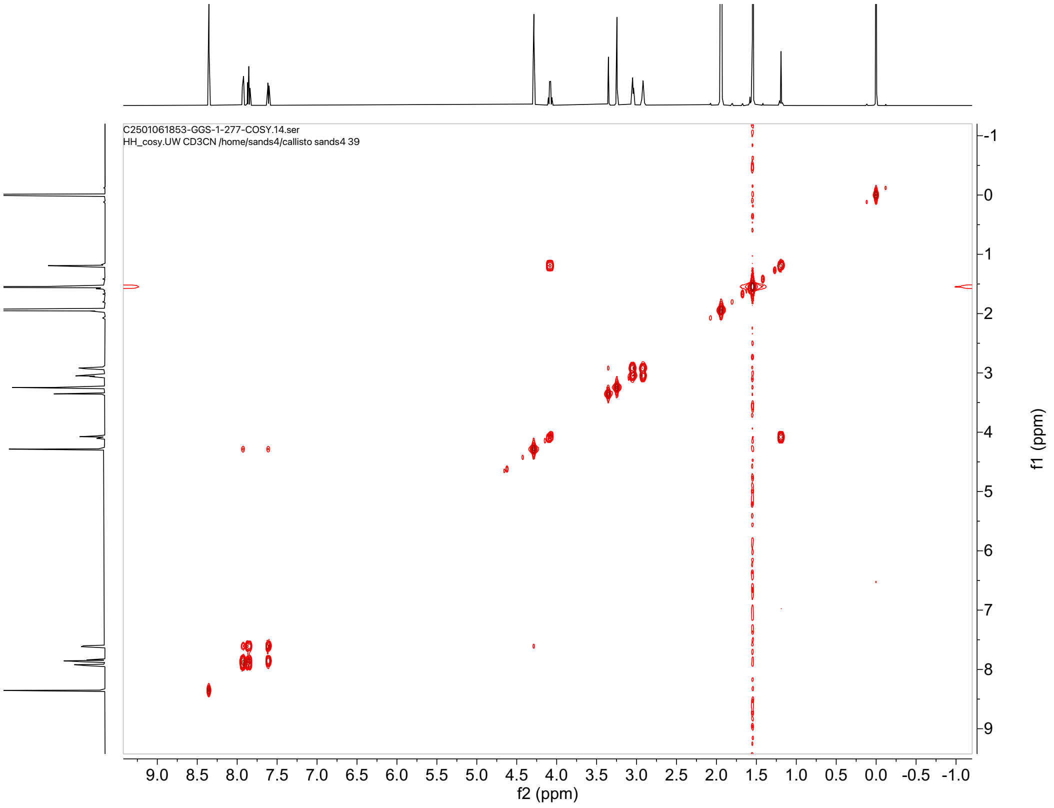


**Figure S171:** COSY of **13**

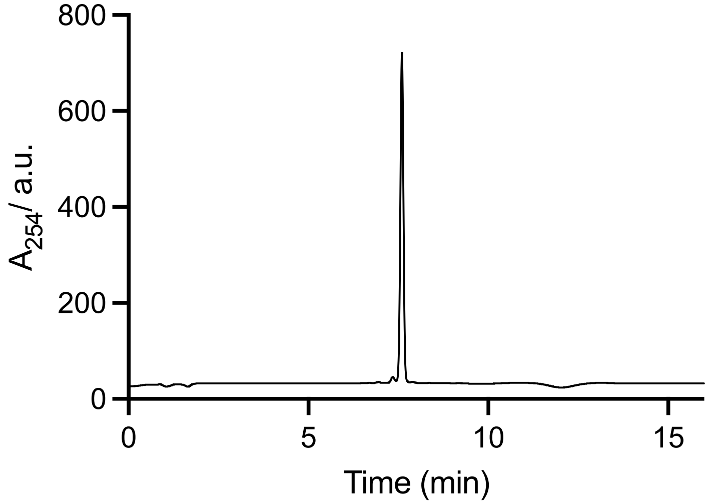


**Figure S172:** Analytical trace of **13** ($R_t$ = 7.61 min) (method E)

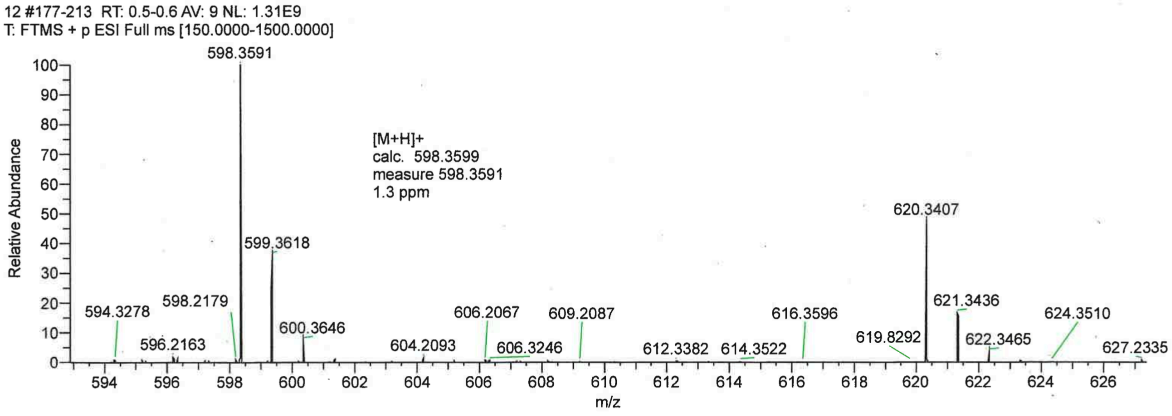


**Figure S173:** HR-ESI of **13**

## S14

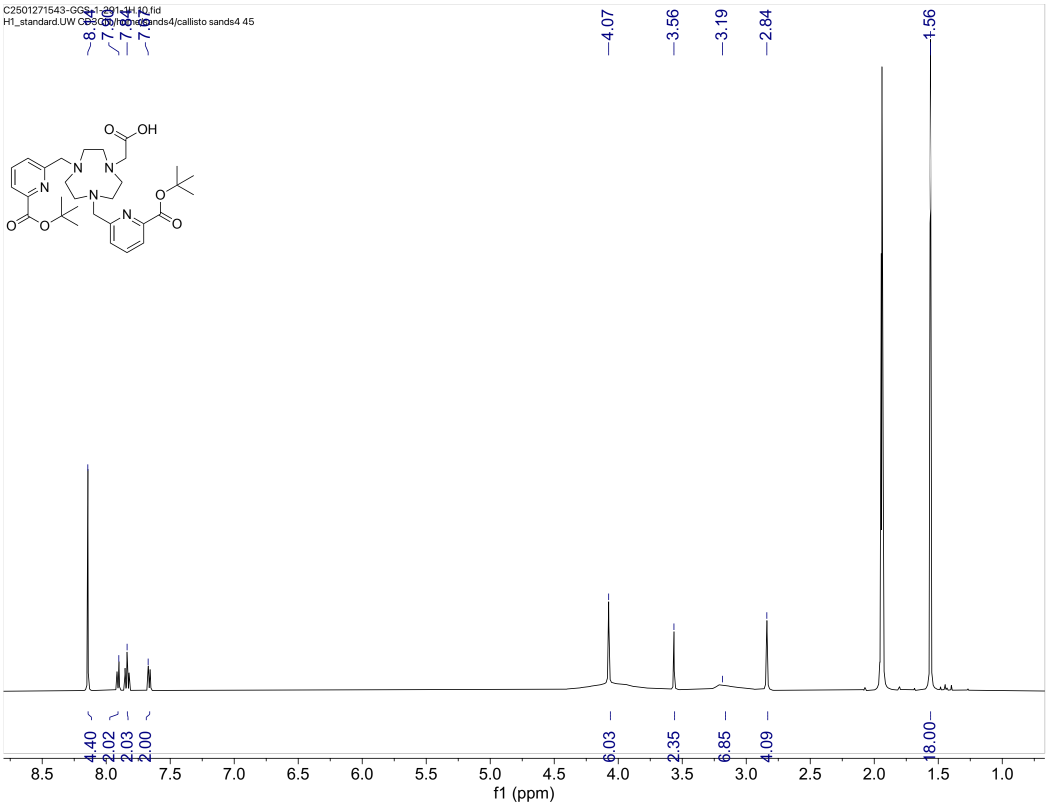


**Figure S174:** ${}^{1}$H NMR of **14** (peak at 8.34 is formic acid)

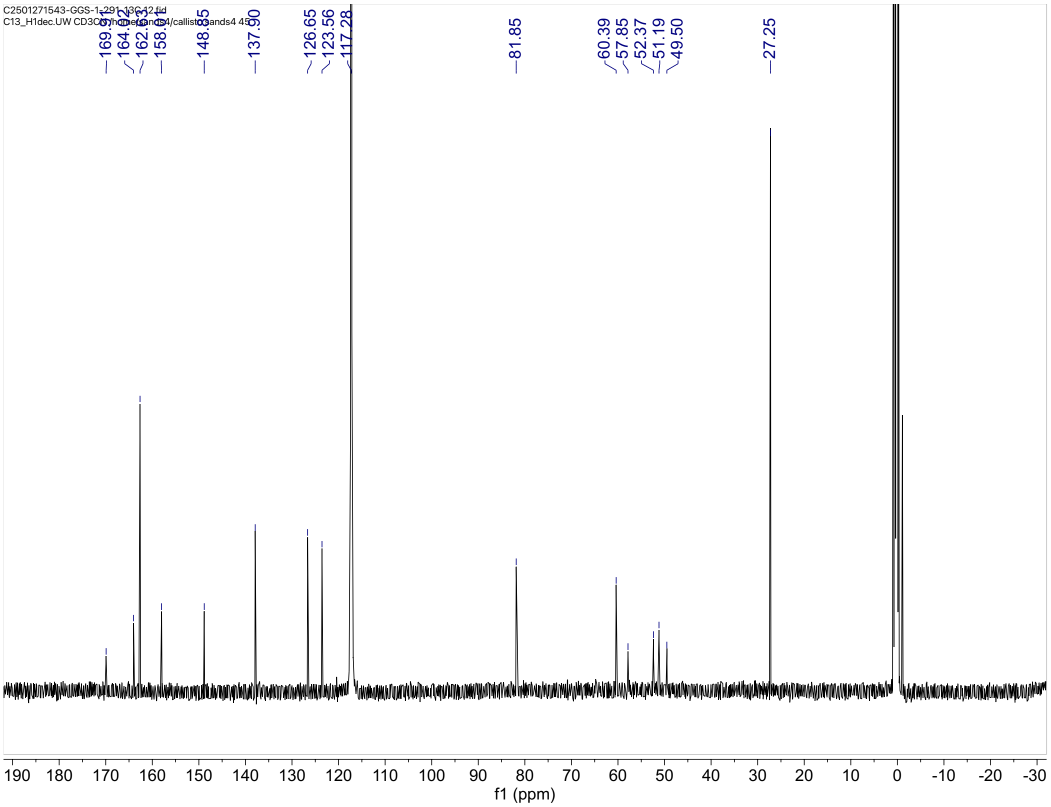


**Figure S175:** $^{13}$C NMR of **14**

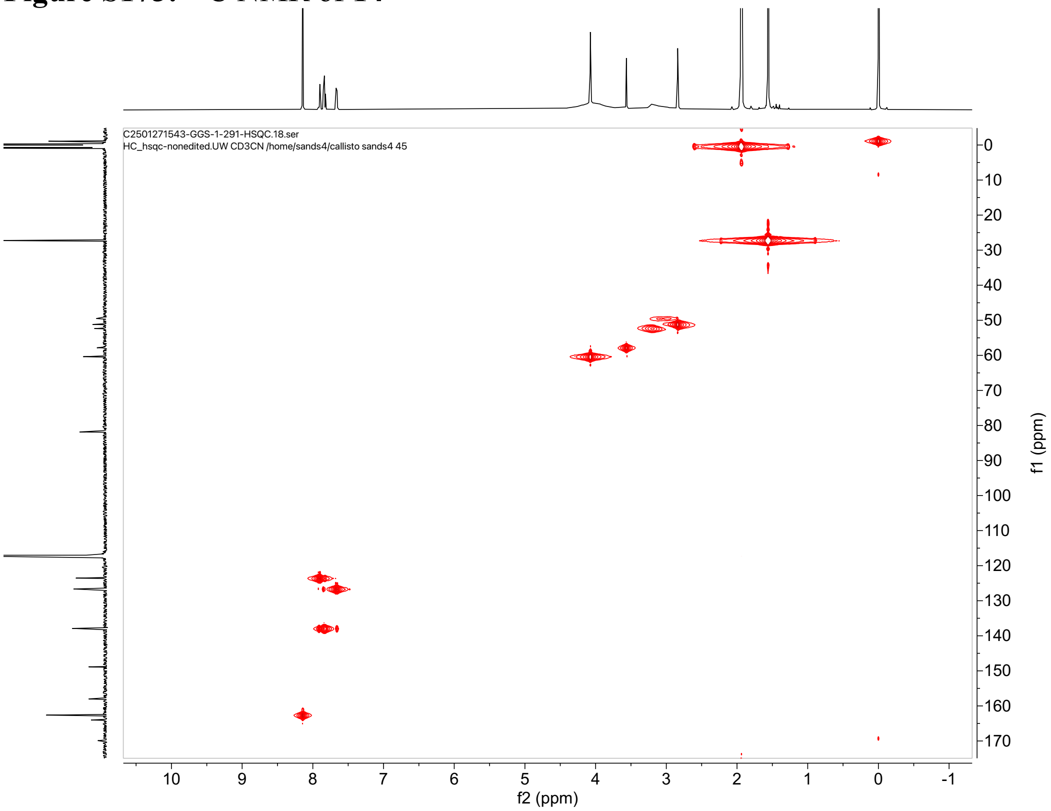


**Figure S176:** HSQC of **14**

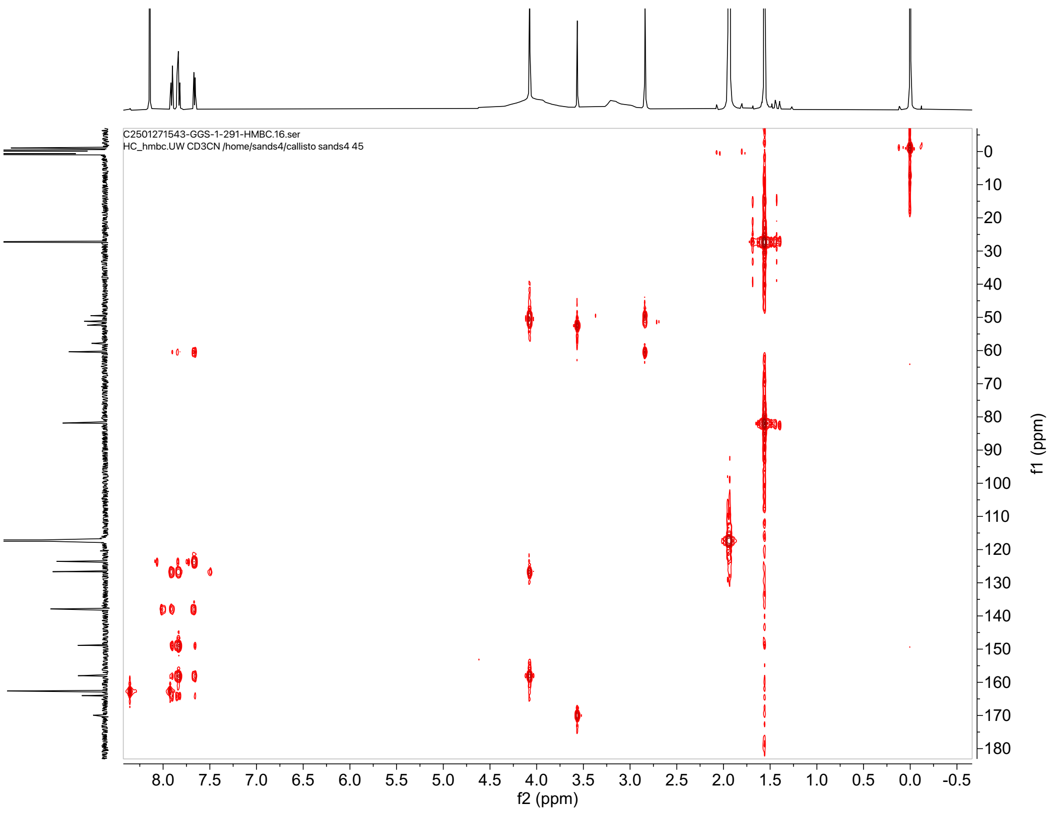


**Figure S177:** HMBC of **14**

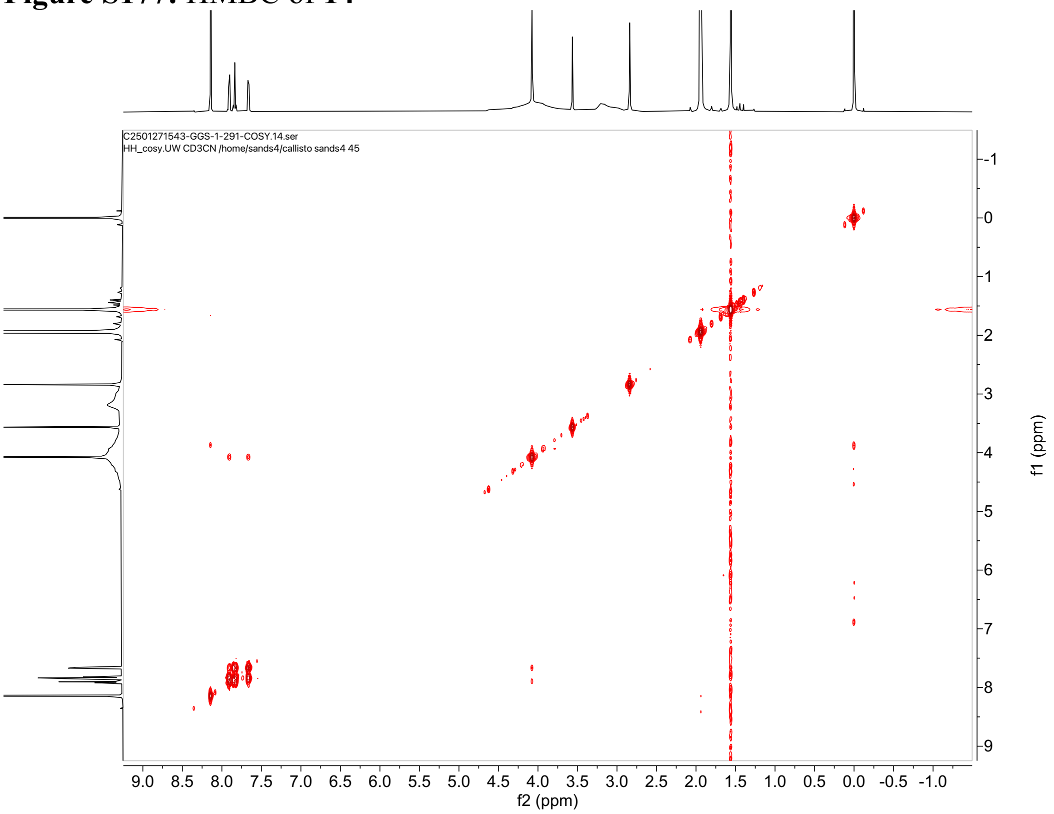


**Figure S178:** COSY of **14**

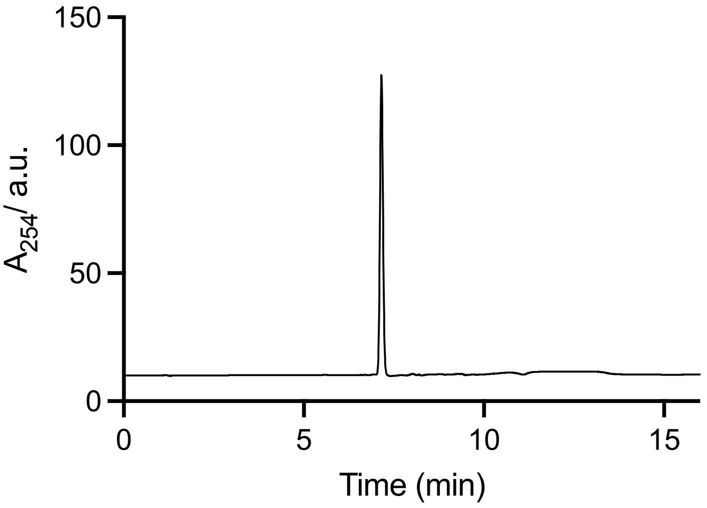


**Figure S179:** Analytical trace of **14** **(**$R_t$ = 7.16 min) (method E)

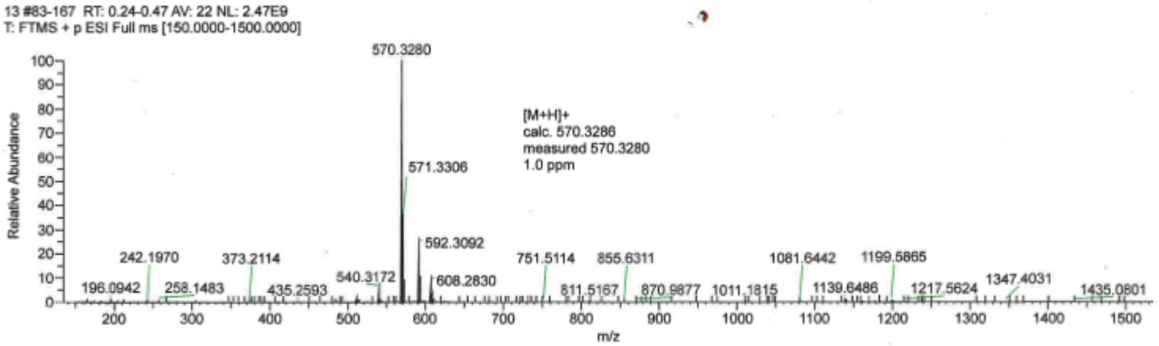


**Figure S180:** HR-ESI of **14**

## S16

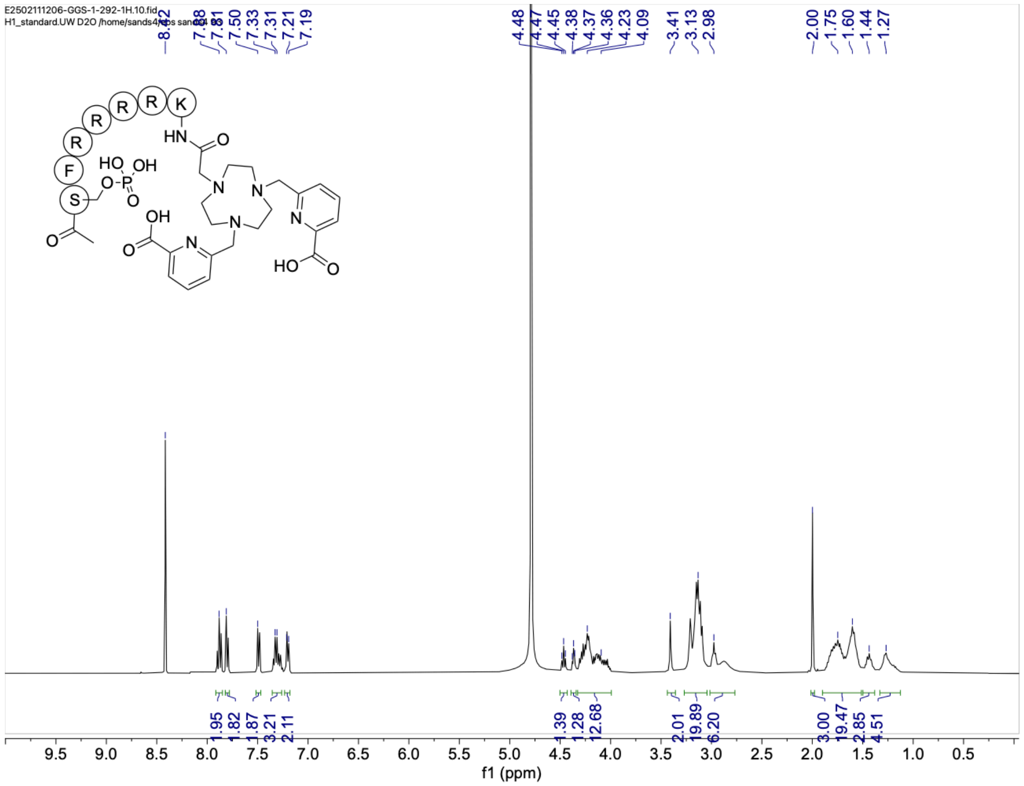


**Figure S181:** $^{1}H$ NMR of **16** (peak at 8.42 is formic acid)

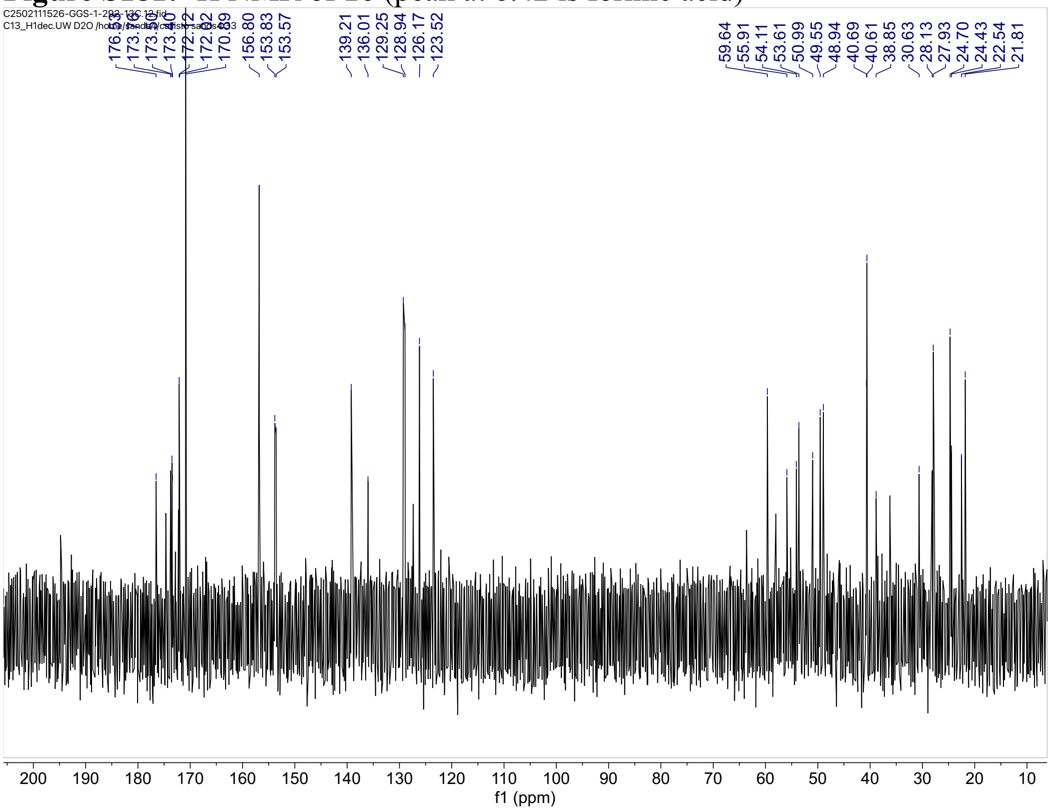


**Figure S182:** $^{13}C$ NMR of **16**

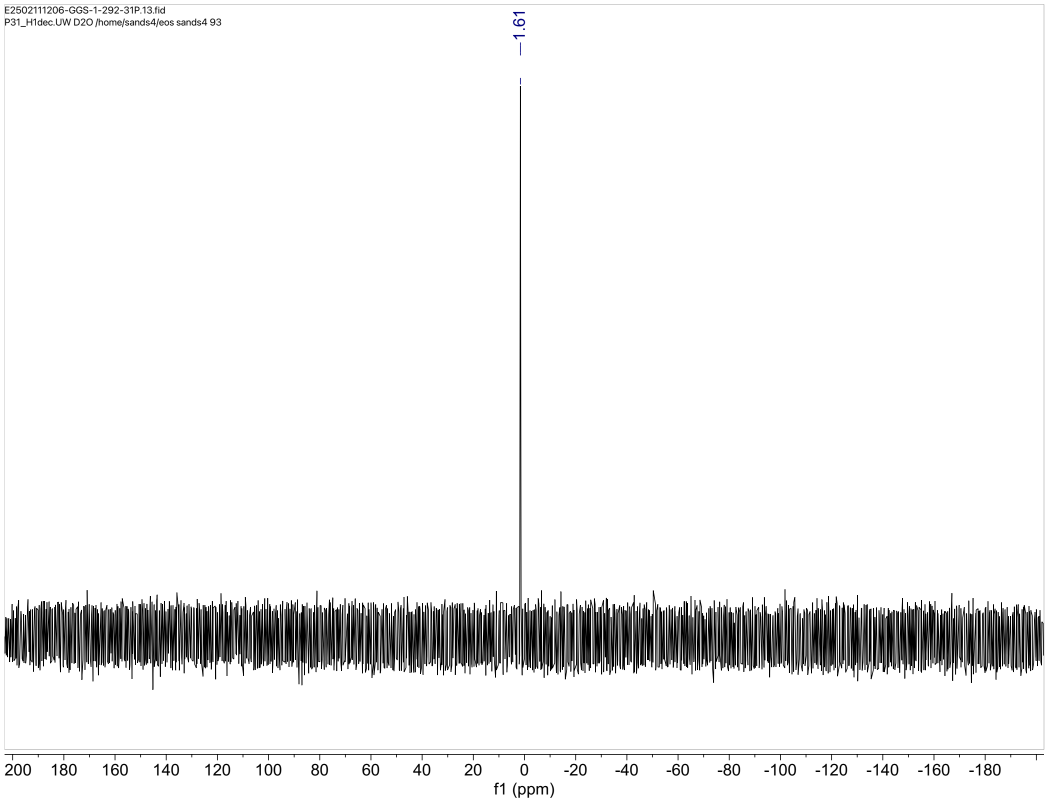


**Figure S183:** $^{31}$P NMR of **16**

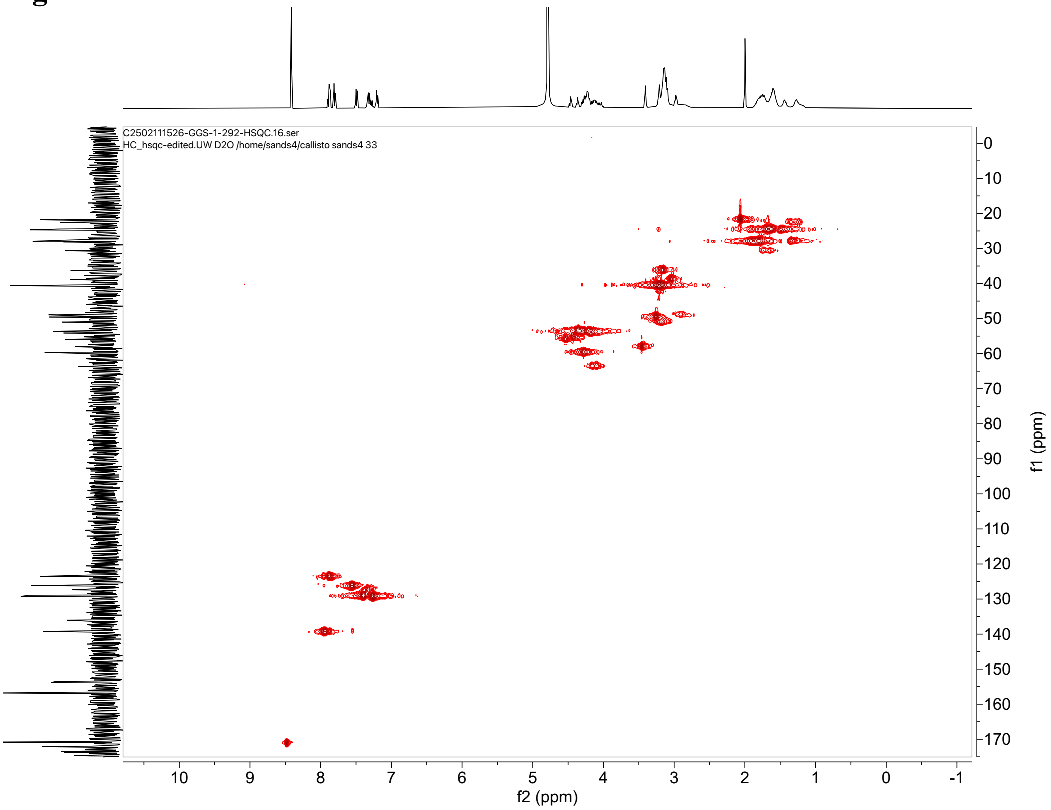

**Figure S184:** HSQC of **16**

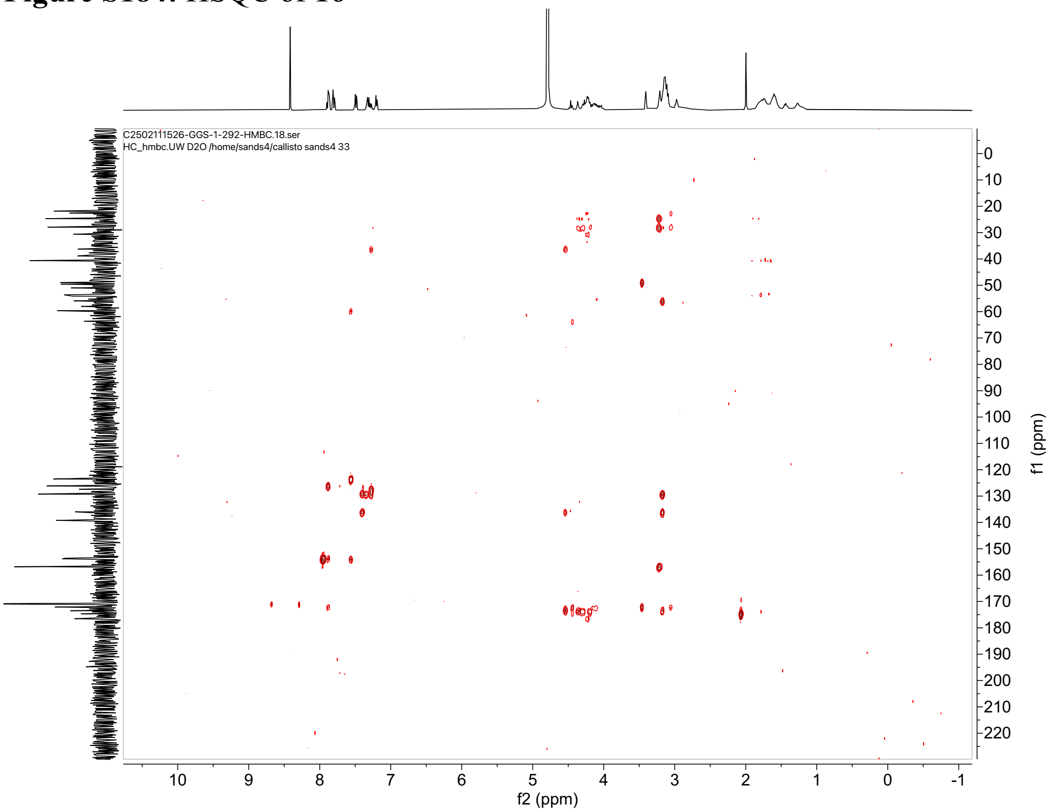


**Figure S185:** HMBC of **16**

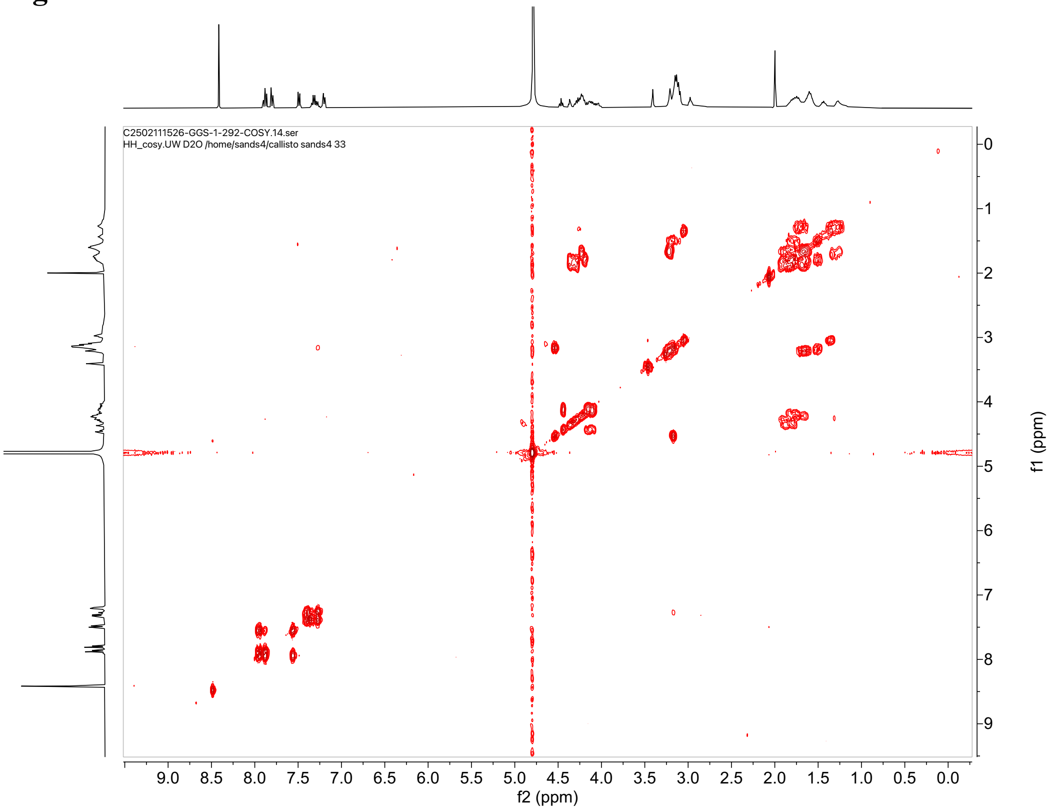


**Figure S186:** COSY of **16**

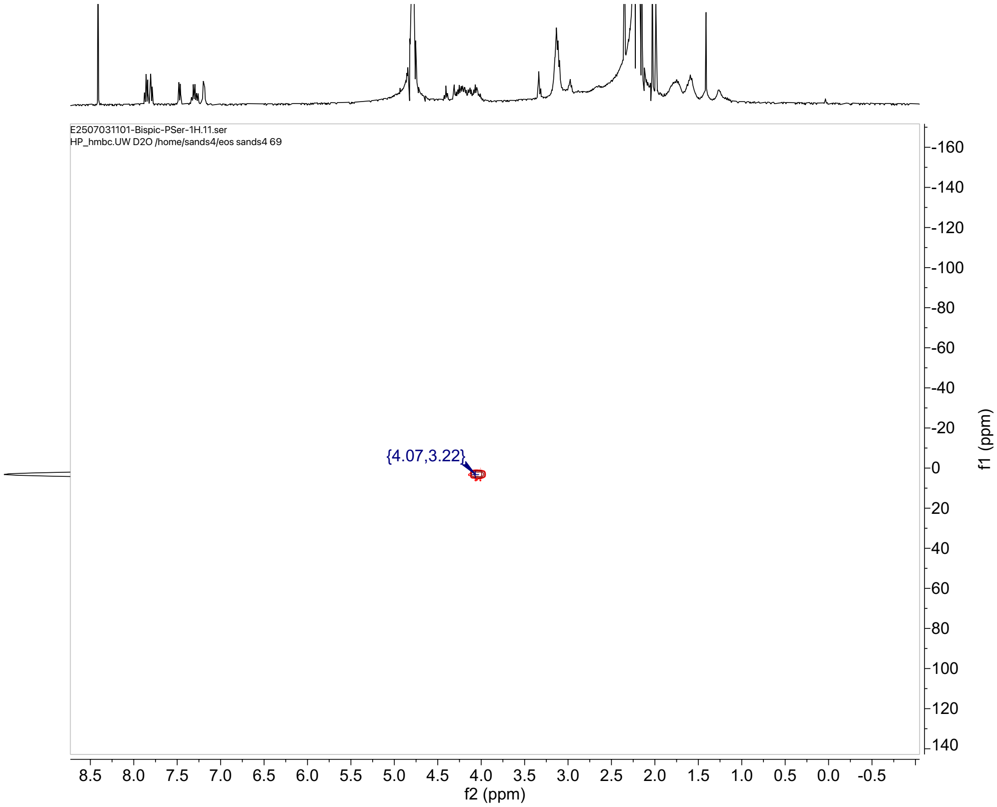


**Figure S187:** $^{1}H^{31}P$ HMBC of **bispic-PSer**

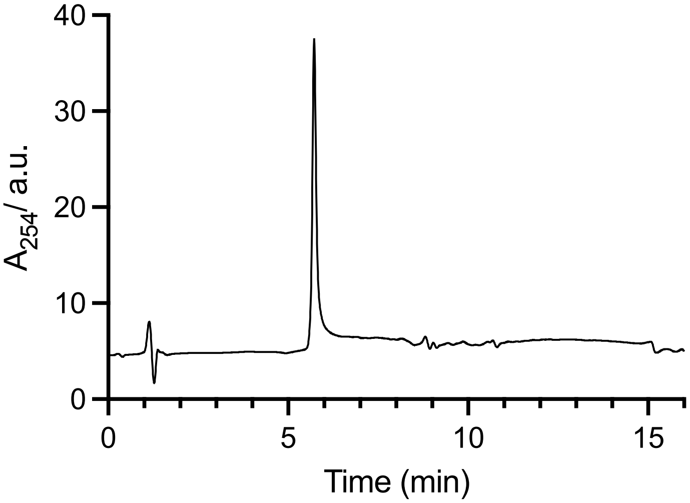


**Figure S188:** Analytical trace of **16** ($R_t$ = 5.73 min) (method E)

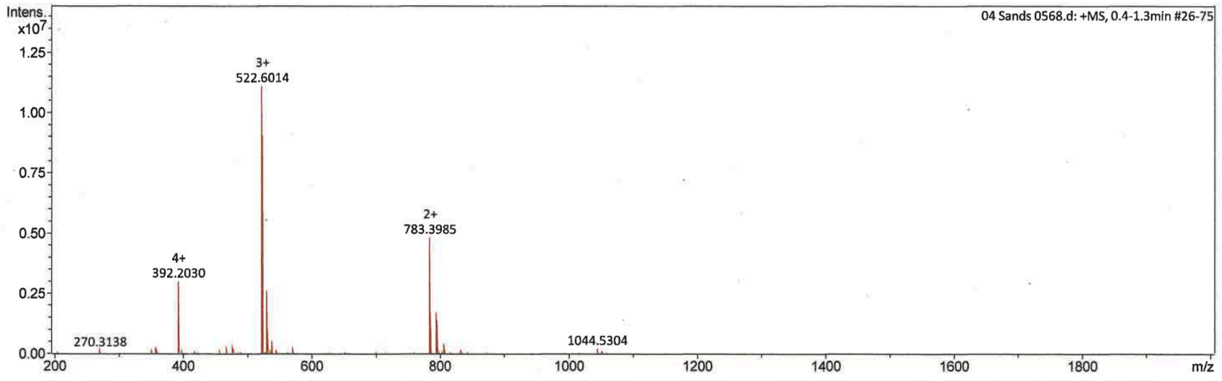


**Figure S189:** HR-ESI of **16**

S**17**

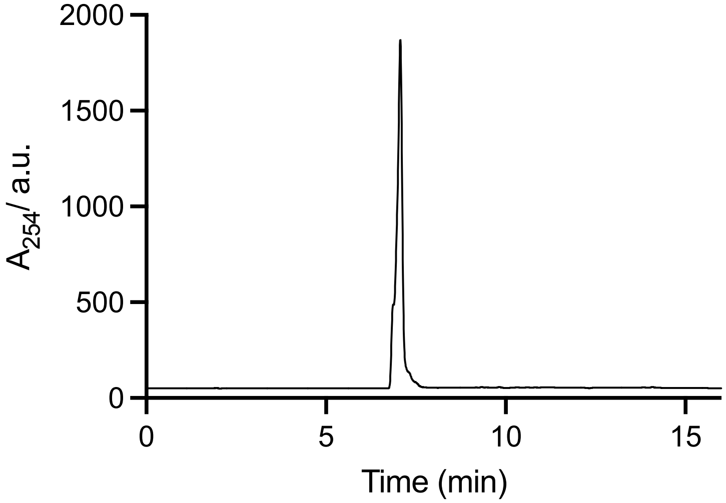


**Figure S190:** Analytical trace of **17** ($R_t$ = 7.08 min) (method D)

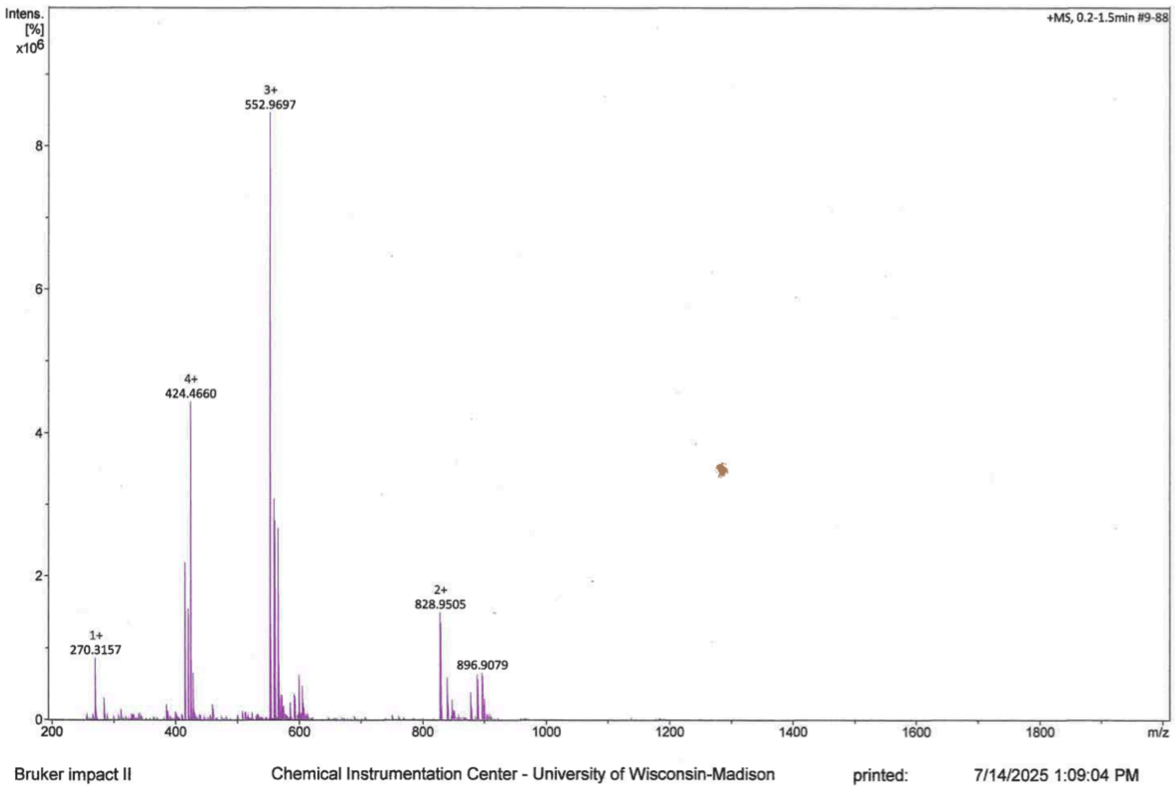


**Figure S191:** HR-ESI of **17**

Synthesis of [**Eu(bispic-PSer)**]$^{-}$

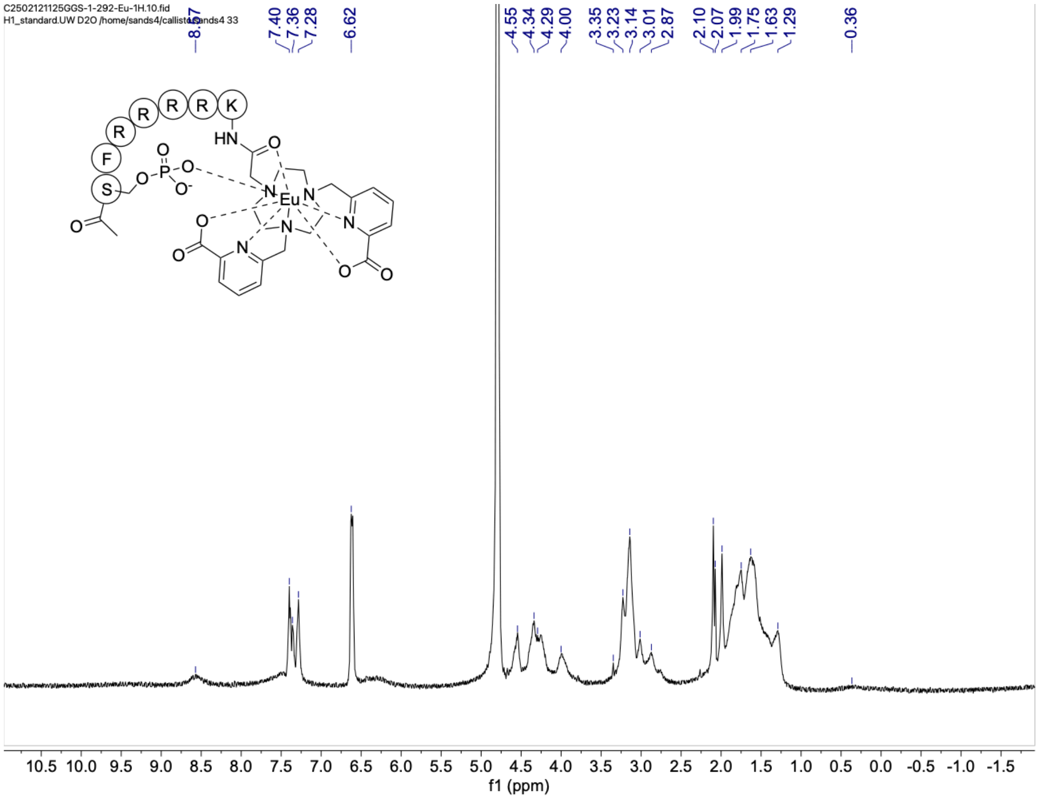

**Figure S192:** $^{1}$H NMR of **[Eu(bispic-PSer)]$^{-}$**

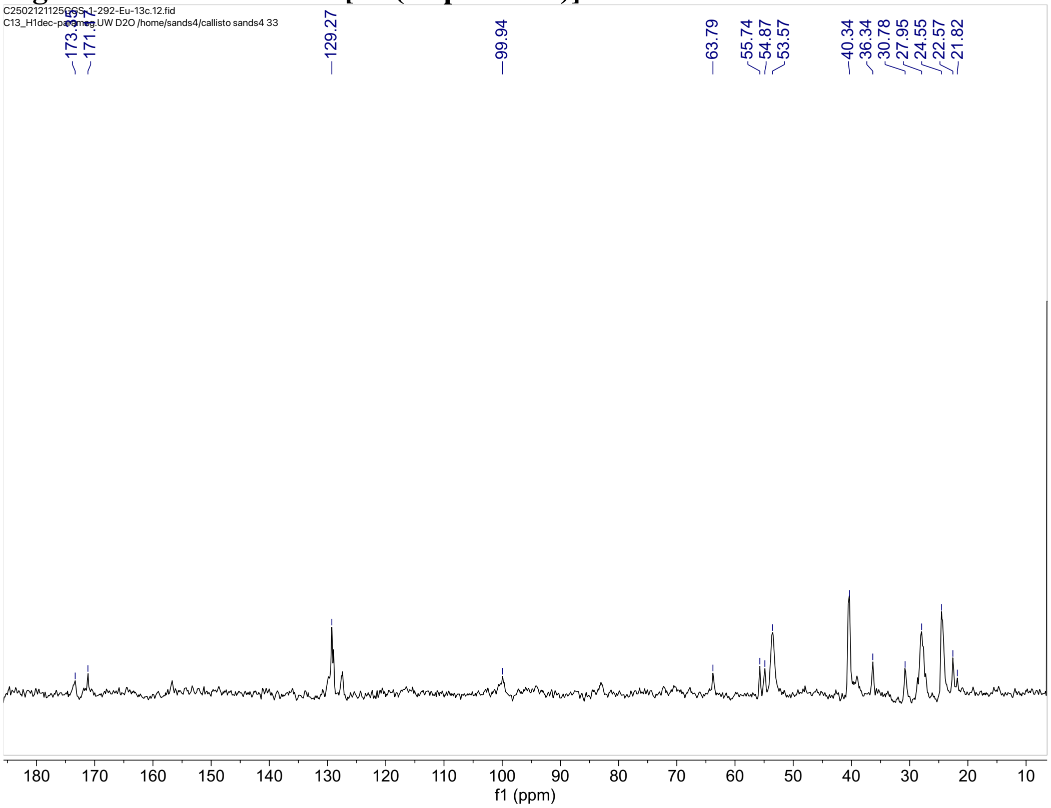

**Figure S193:** $^{13}$C NMR of **[Eu(bispic-PSer)]$^{-}$**

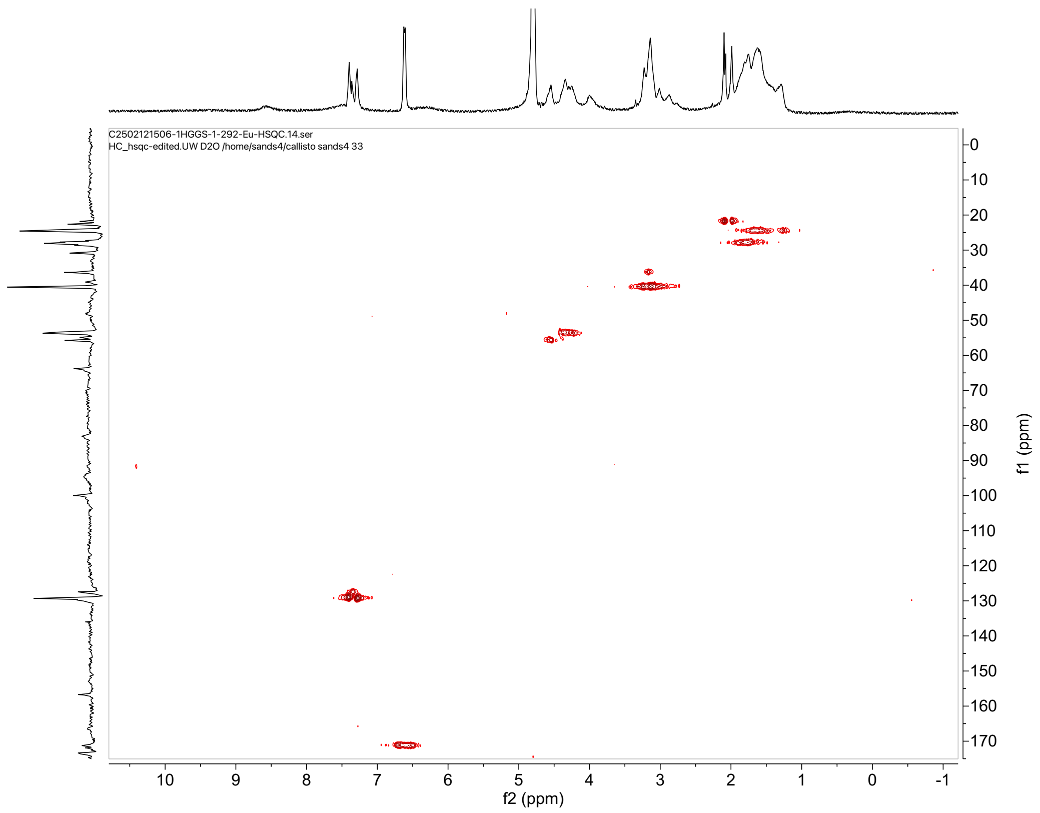


**Figure S194:** HSQC of **[Eu(bispic-PSer)]**$^-$

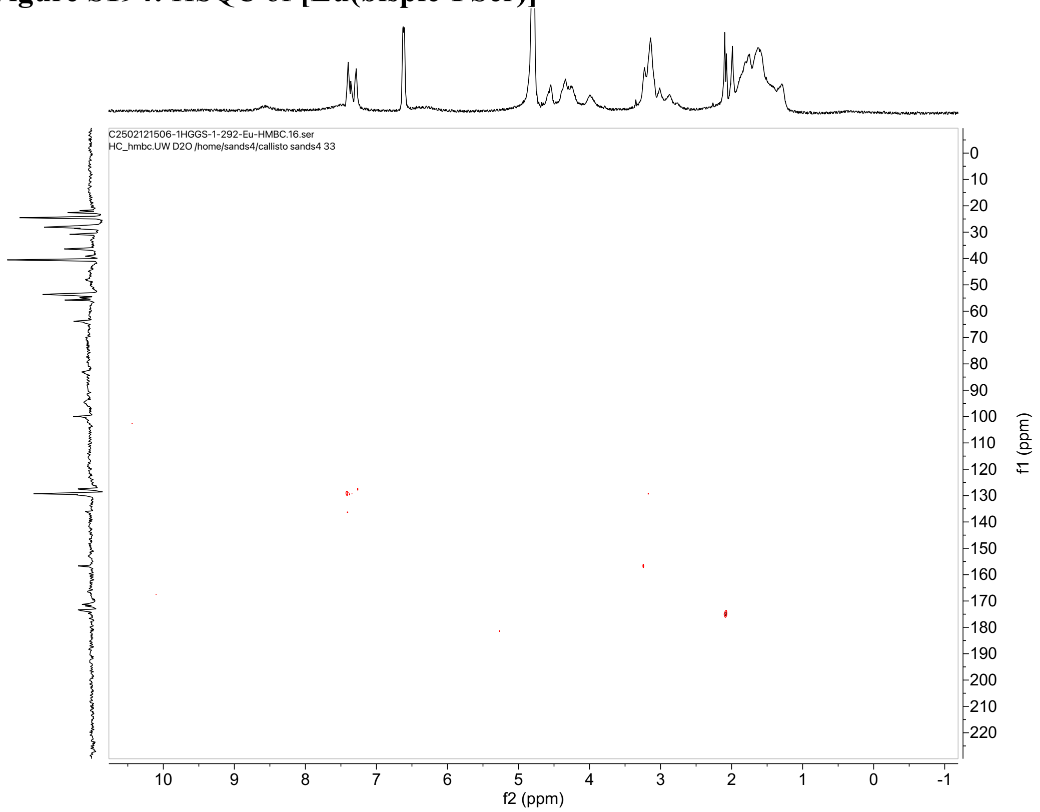


**Figure S195:** HMBC of **[Eu(bispic-PSer)]**$^-$

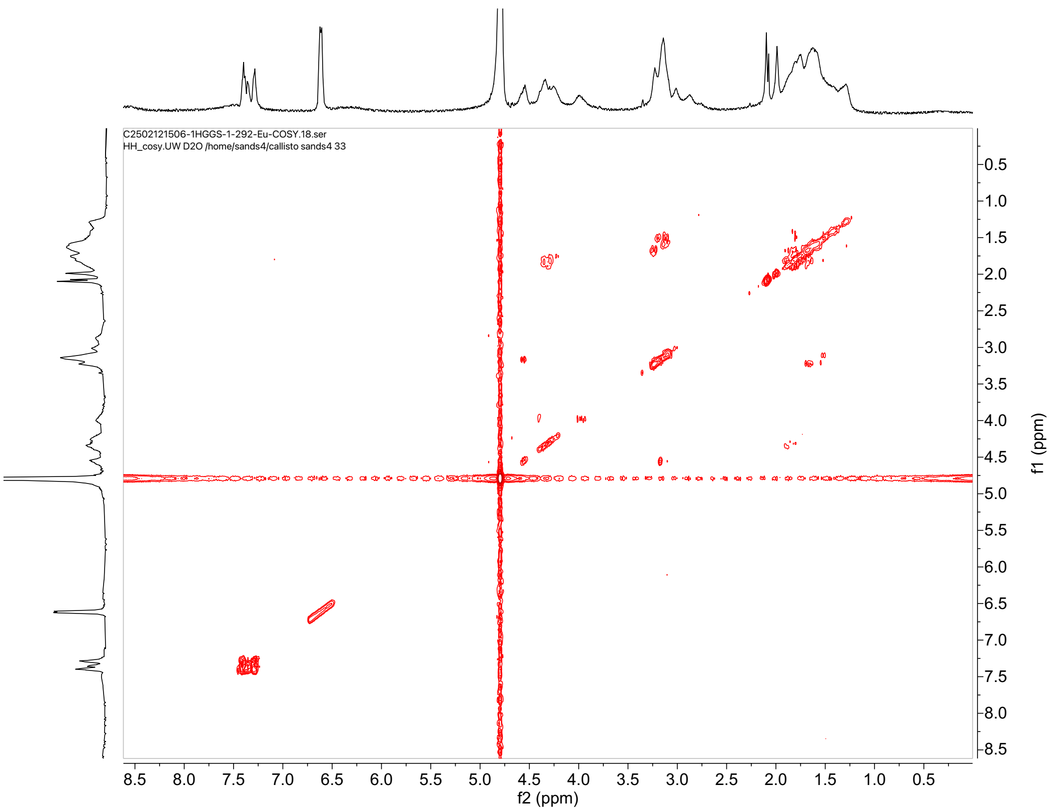


**Figure S196:** COSY of **[Eu(bispic-PSer)]⁻**

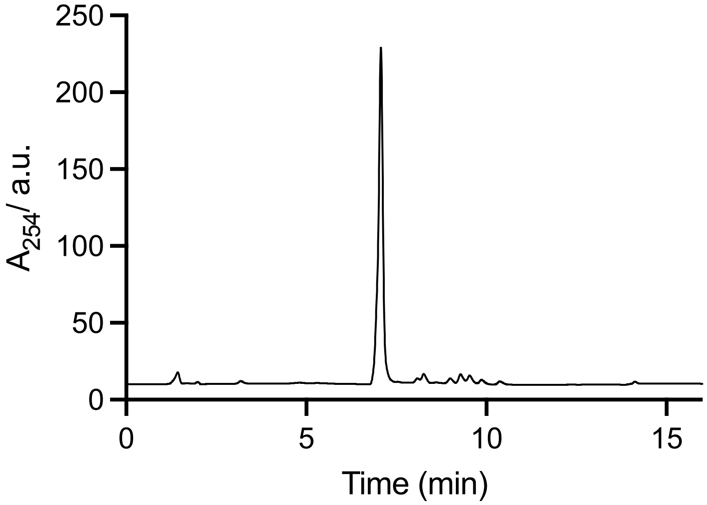


**Figure S197:** Analytical trace of **[Eu(bispic-PSer)]⁻** ($R_t$ = 6.96 min) (method F)

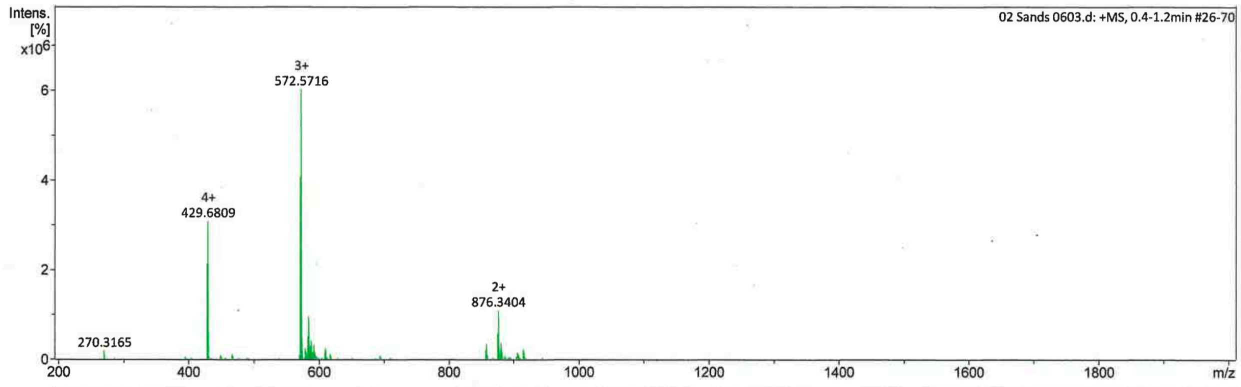


**Figure S198:** HR-ESI of **[Eu(bispic-PSer)]$^-$**

Synthesis of **[Tb(bispic-PSer)]$^-$**

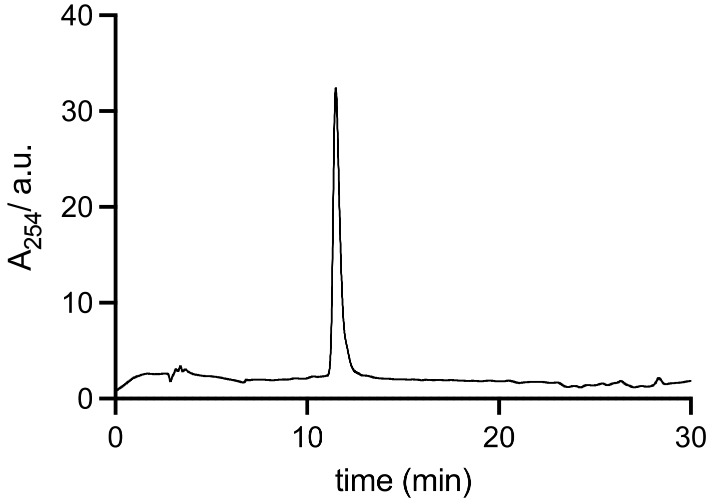


**Figure S199:** Analytical trace of **[Tb(bispic-PSer)]$^-$** (method I)

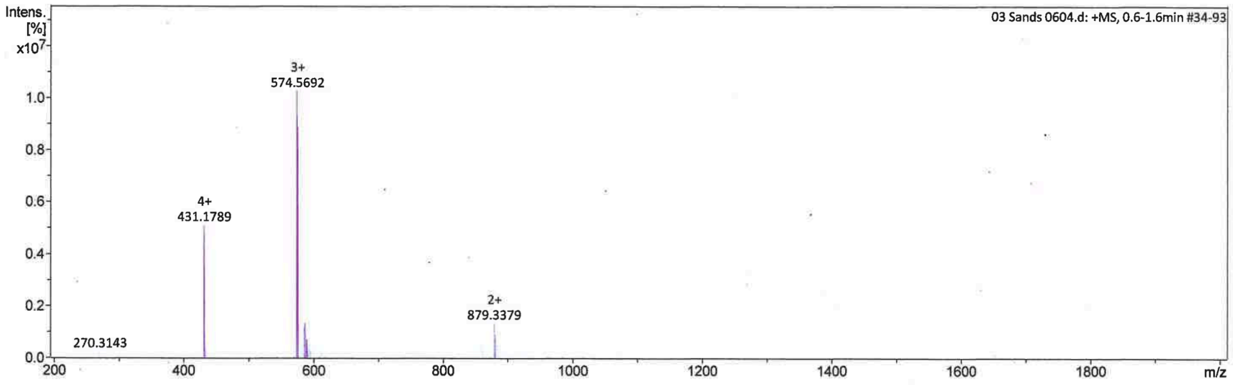


**Figure S200:** HR-ESI of **[Tb(bispic-PSer)]$^-$**

Synthesis of **[Lu(bispic-PSer]$^-$**

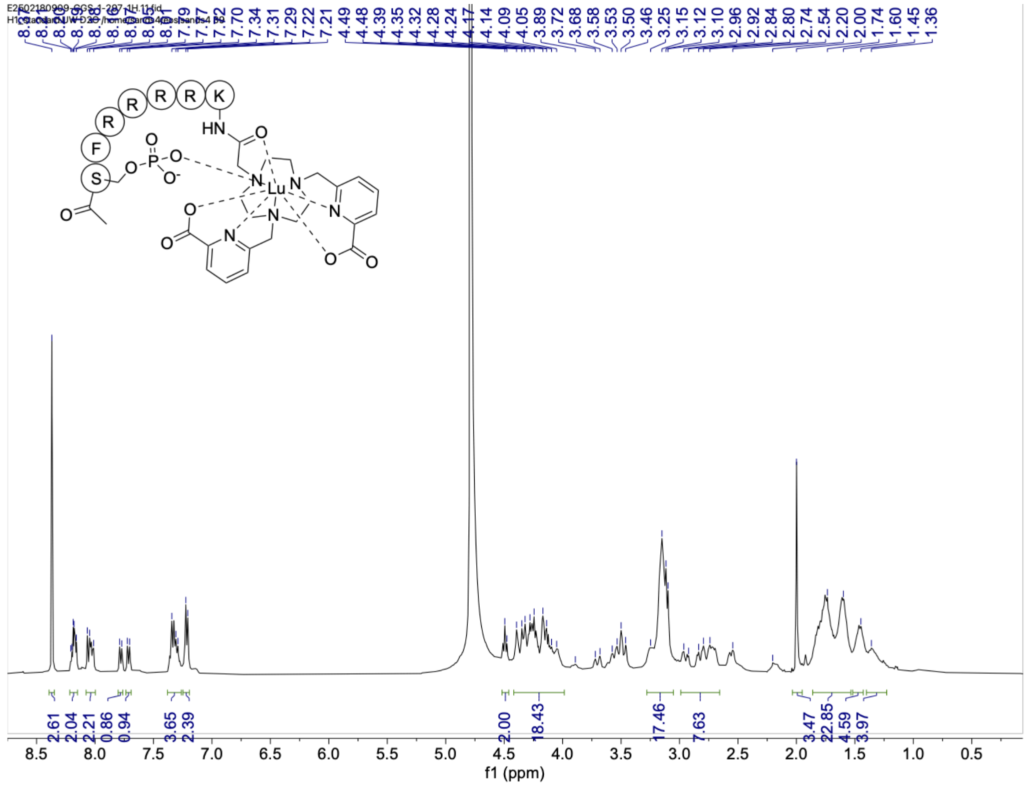

**Figure S201:** $^{1}$H NMR of **[Lu(bispic-PSer)]$^{-}$**

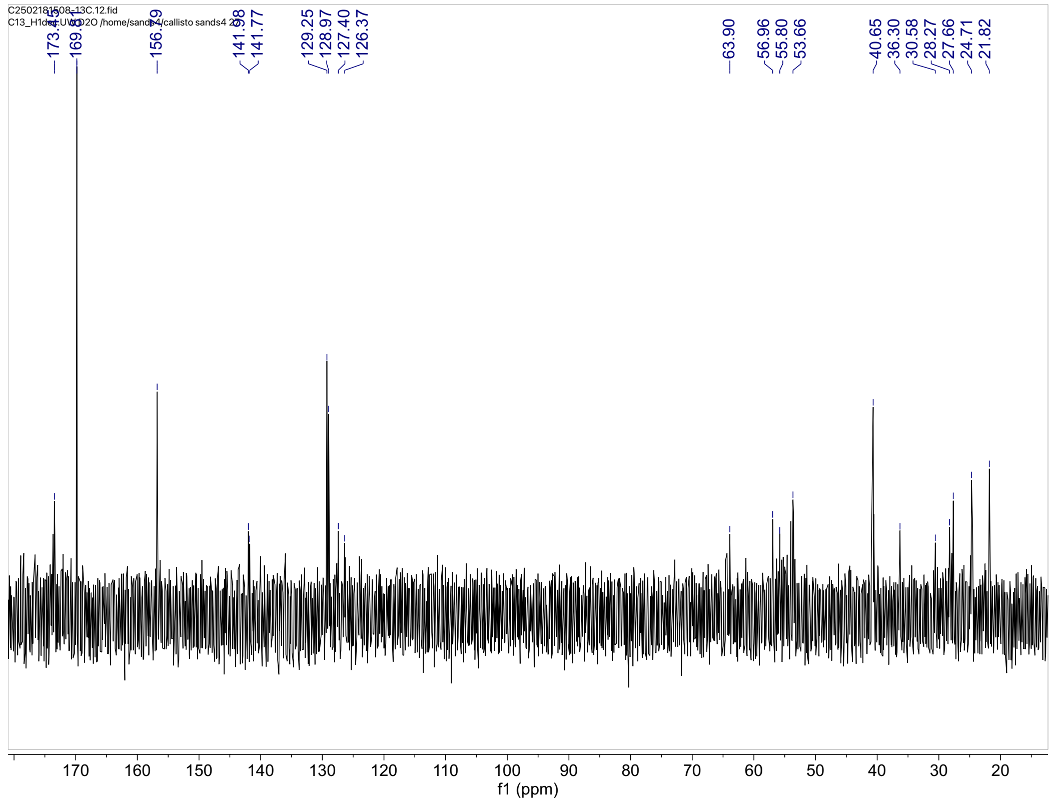

**Figure S202:** $^{13}$C NMR of **[Lu(bispic-PSer)]$^{-}$**

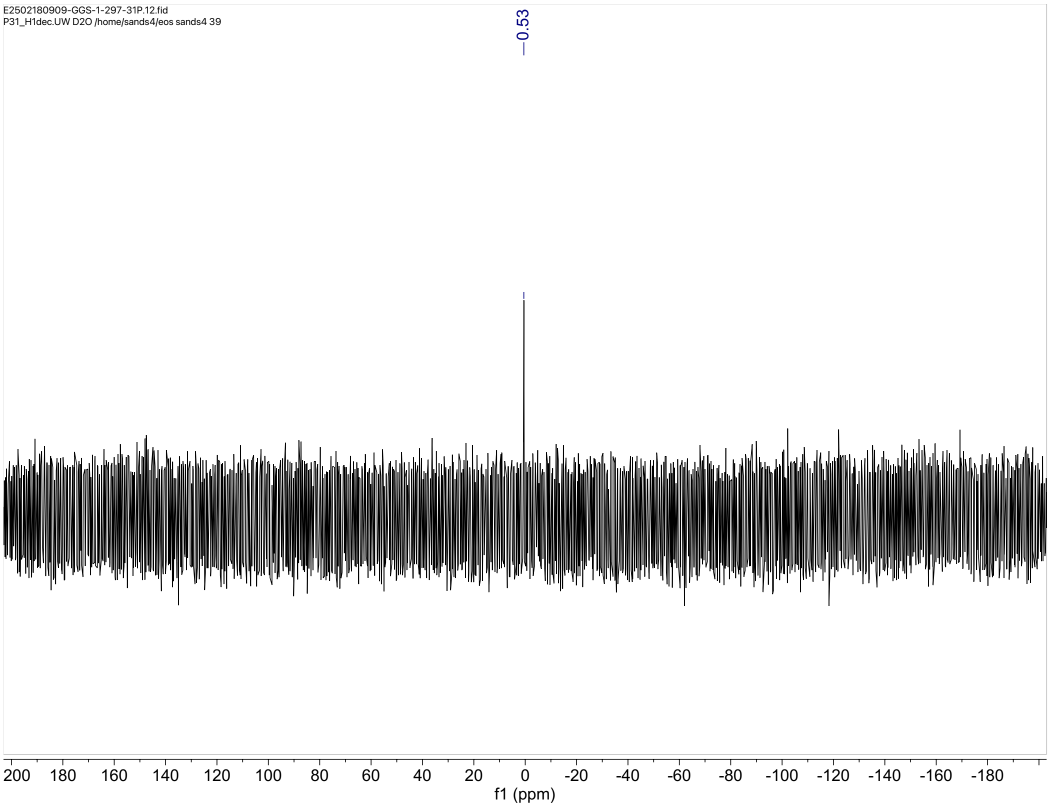


**Figure S203:** $^{31}$P NMR of **[Lu(bispic-PSer)]$^{-}$**

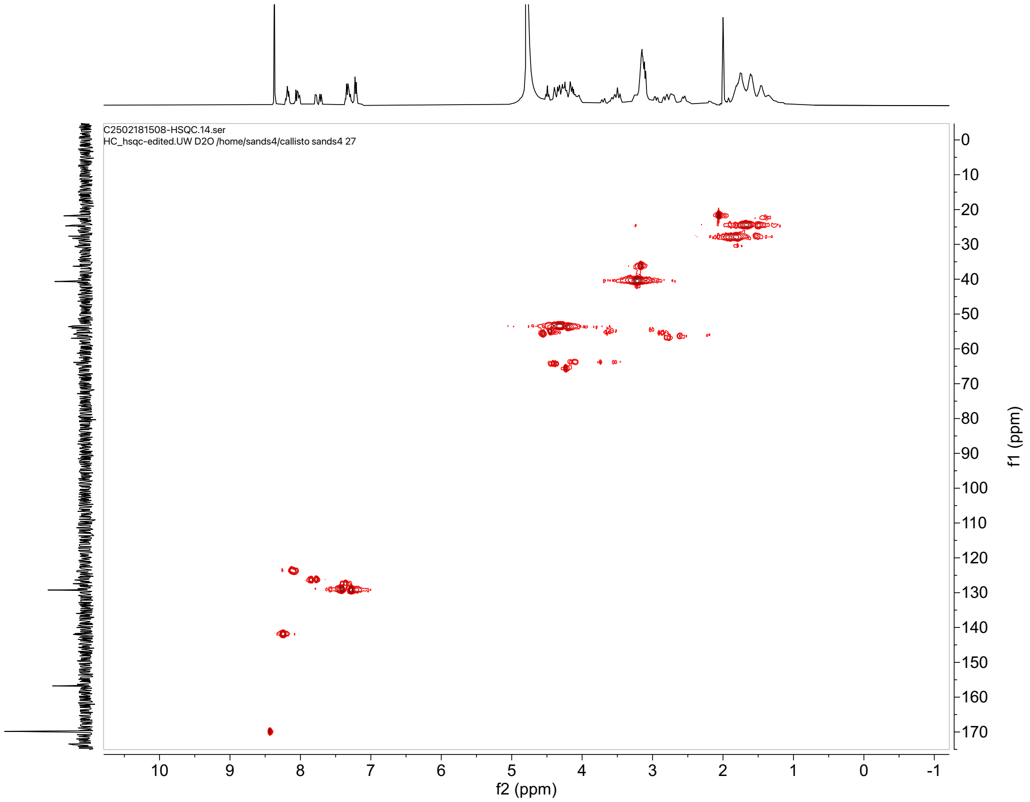


**Figure S204:** HSQC of **[Lu(bispic-PSer)]$^{-}$**

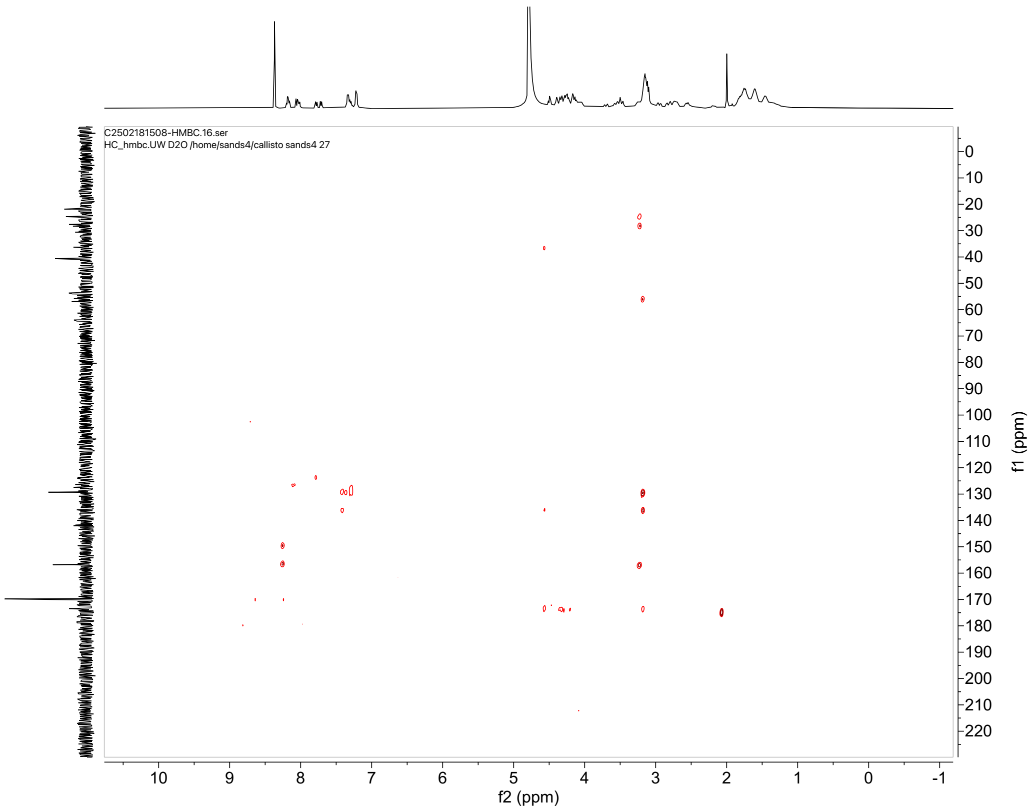


**Figure S205:** HMBC of **[Lu(bispic-PSer)]**$^-$

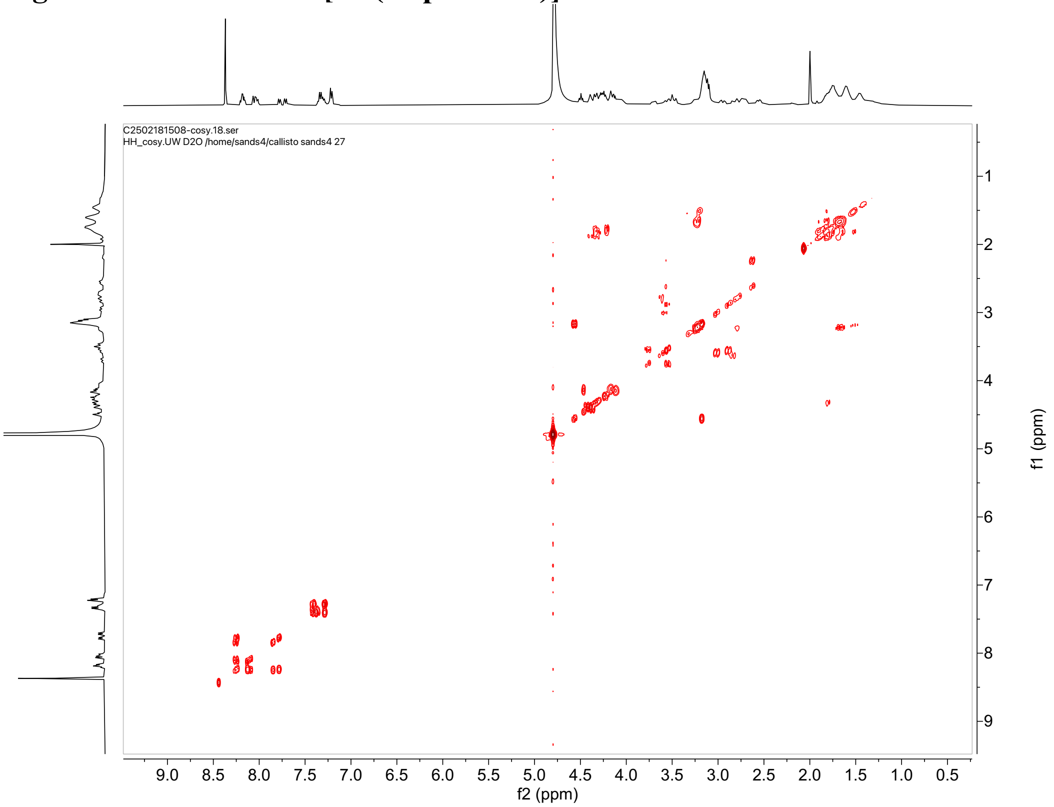


**Figure S206:** COSY of **[Lu(bispic-PSer)]**$^-$

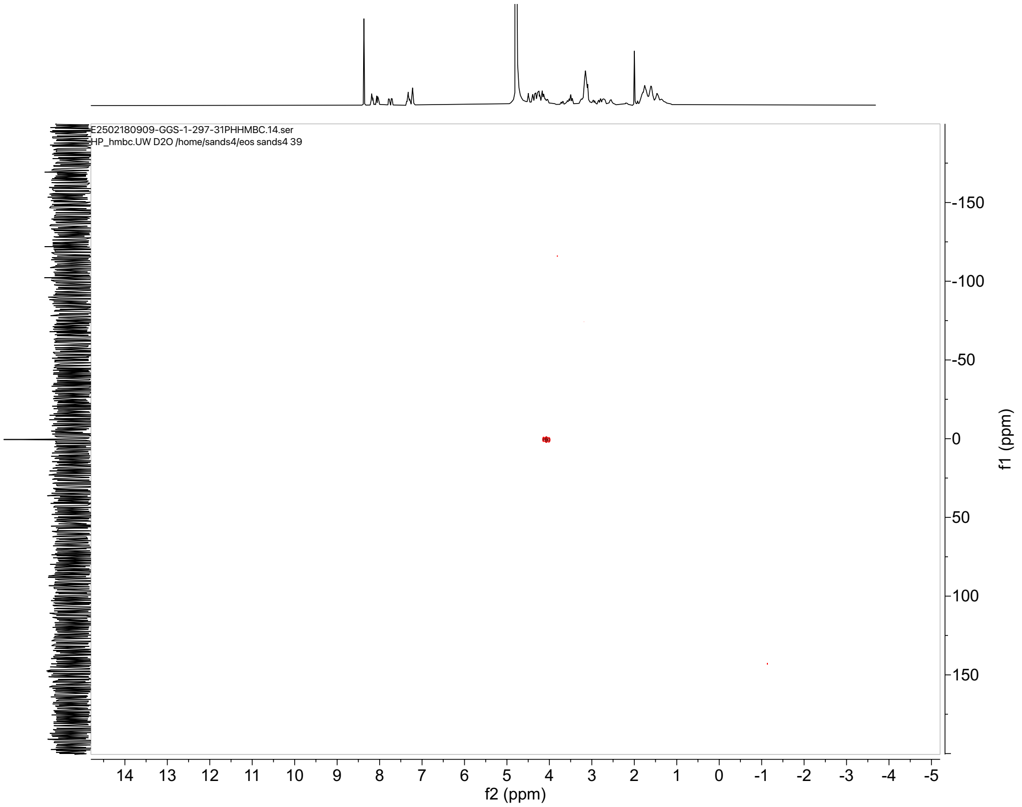


**Figure S207:** $^{1}H^{31}P$ HMBC of **[Lu(bispic-PSer)]⁻**

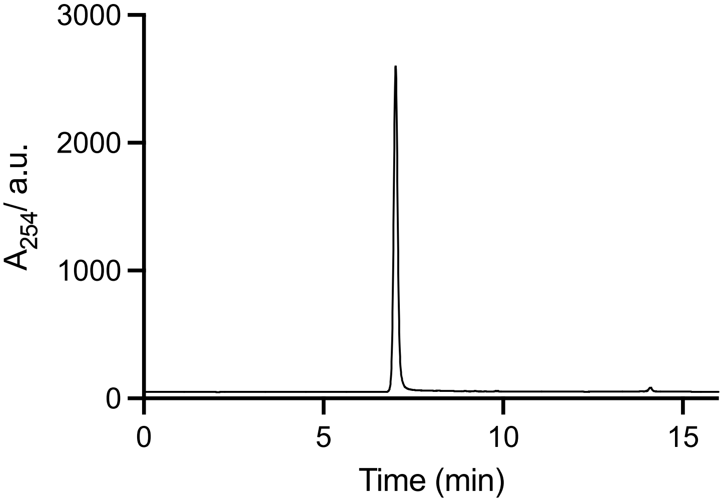


**Figure S208:** Analytical trace of **[Lu(bispic-PSer)]⁻** ($R_t$ = 7.02 min) (method D)

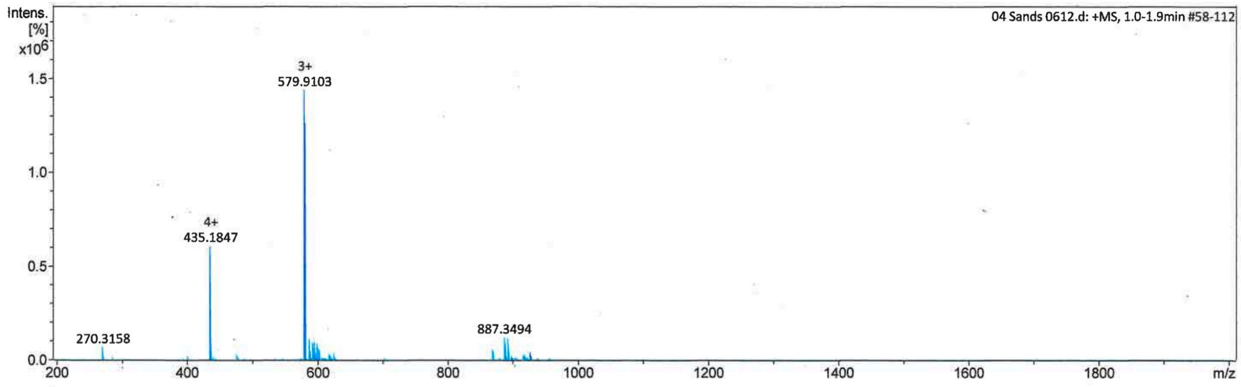


**Figure S209:** HR-ESI of **[Lu(bispic-PSer)]**⁻

## S18

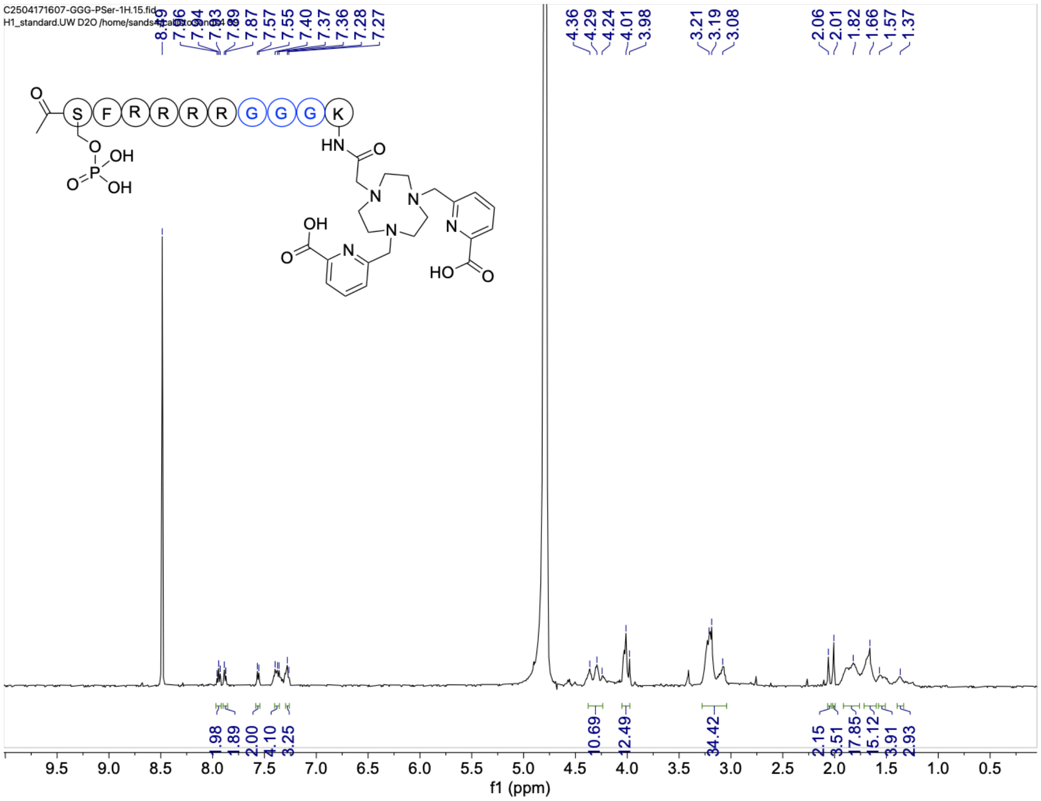


**Figure S210:** $^{1}$H NMR of **18** (peak at 8.49 is formic acid)

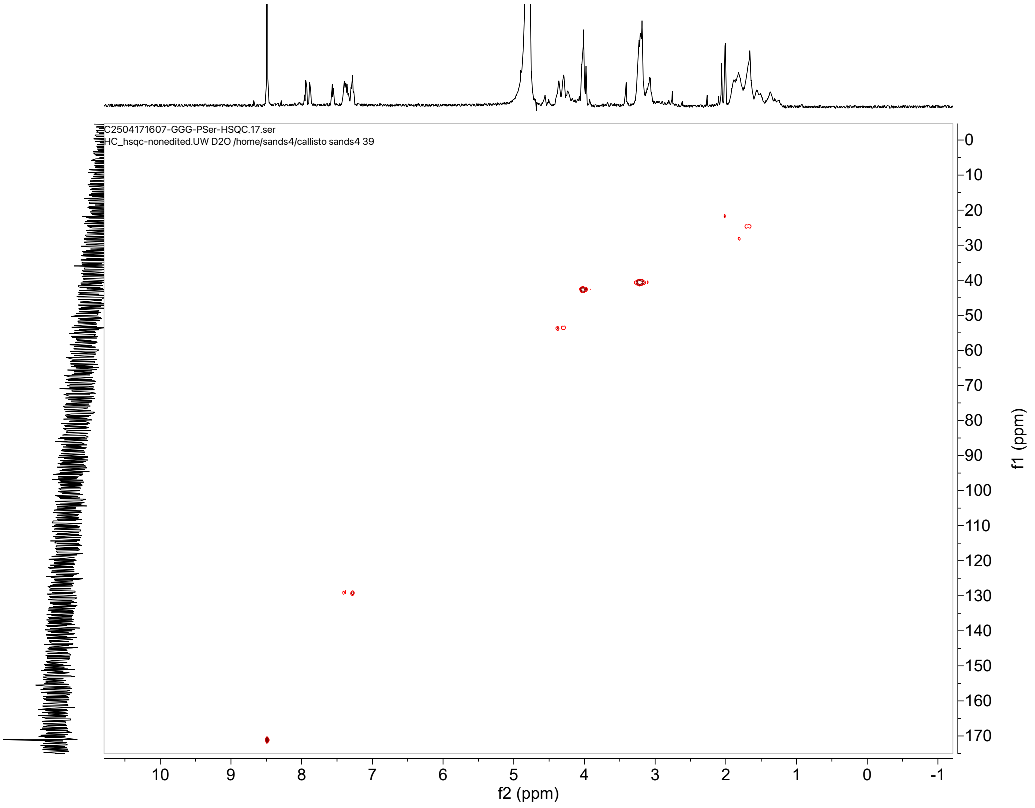


**Figure S211:** HSQC of **18**

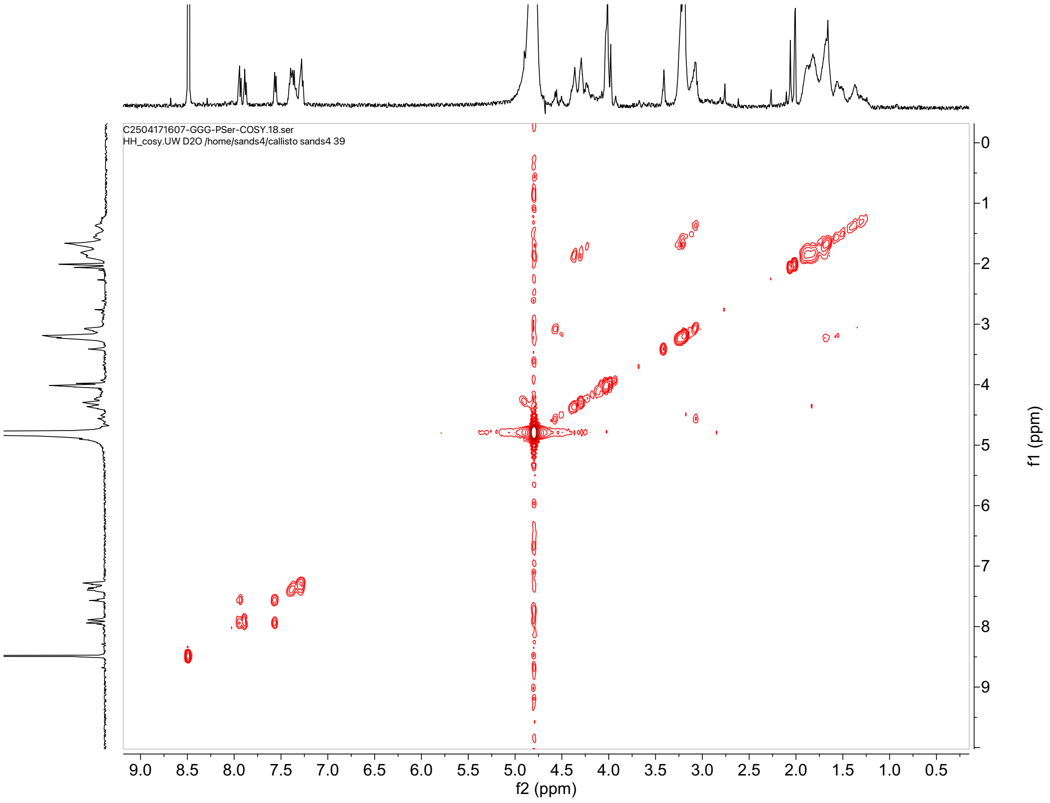


**Figure S212:** COSY of **18**

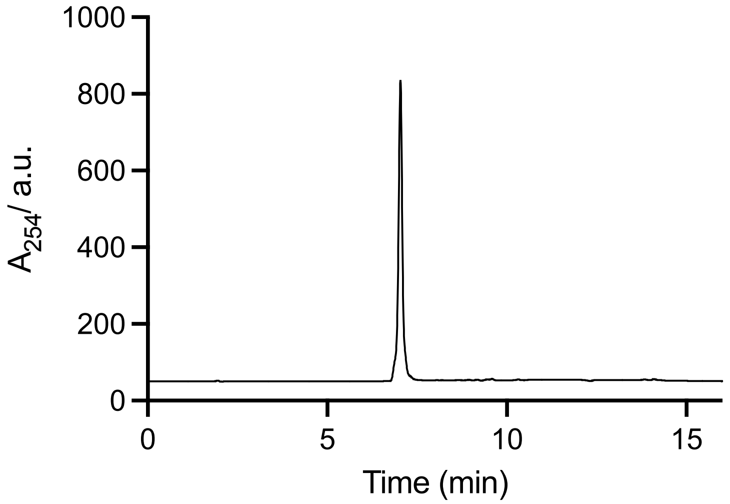


**Figure S213:** Analytical trace of **18** ($R_t$ = 7.04 min) (Method D)

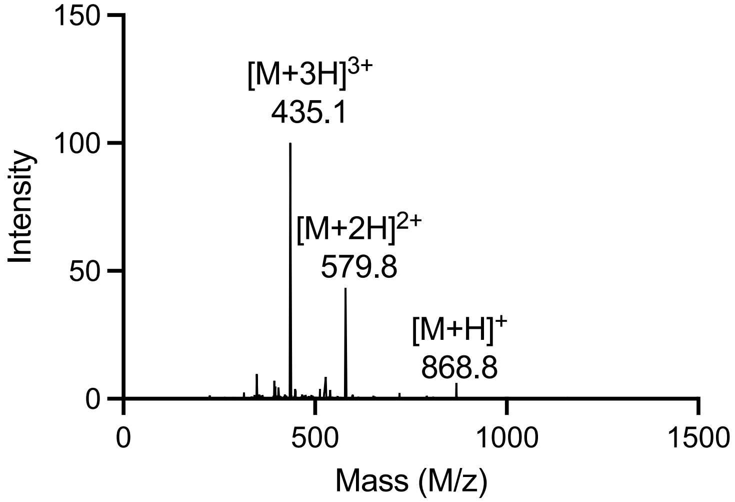


**Figure S214:** LR-ESI of **18**

S**19**

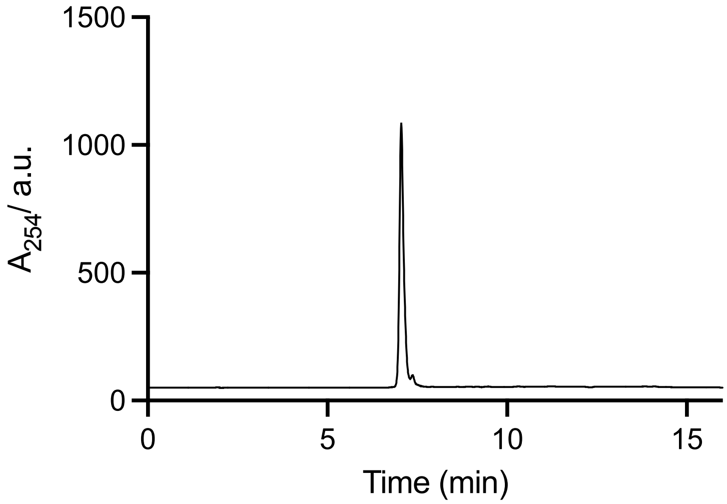


**Figure S215:** Analytical trace of **19** ($R_t$ = 7.06 min) (Method D)

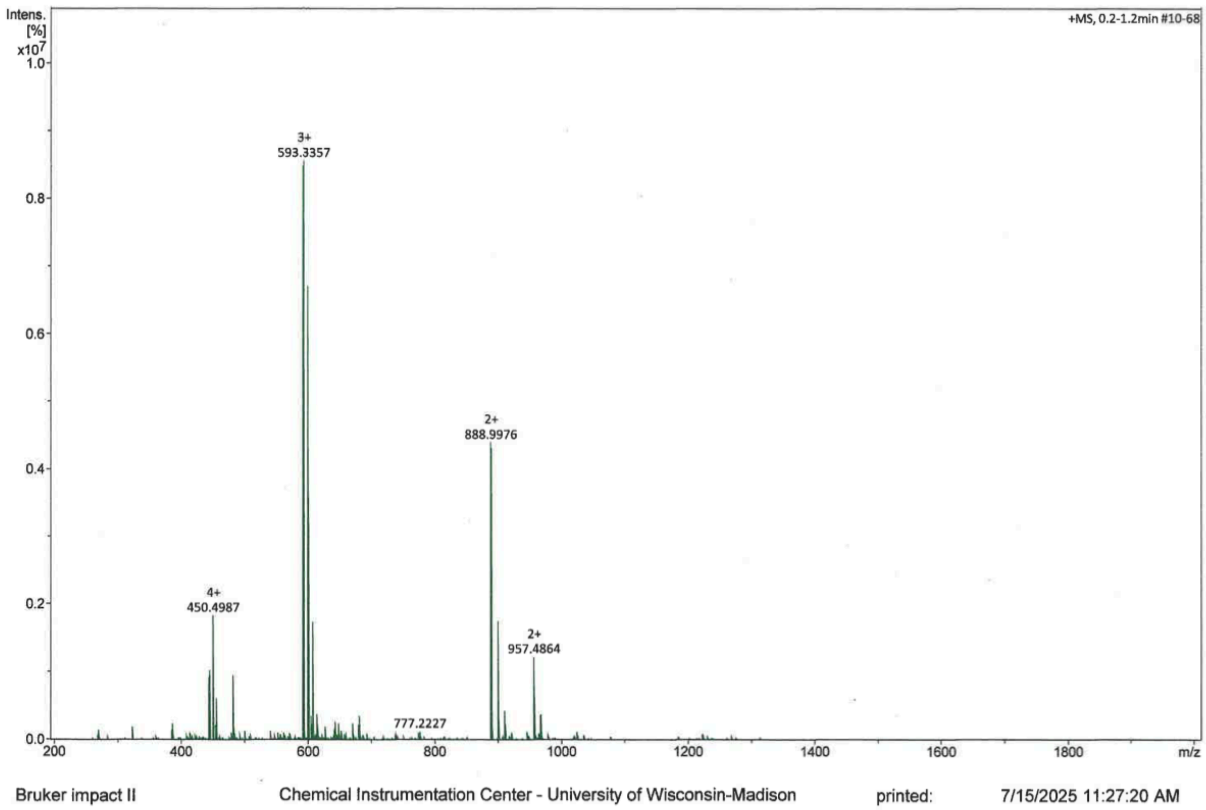


**Figure S216:** HR-ESI of **19**

S**20**

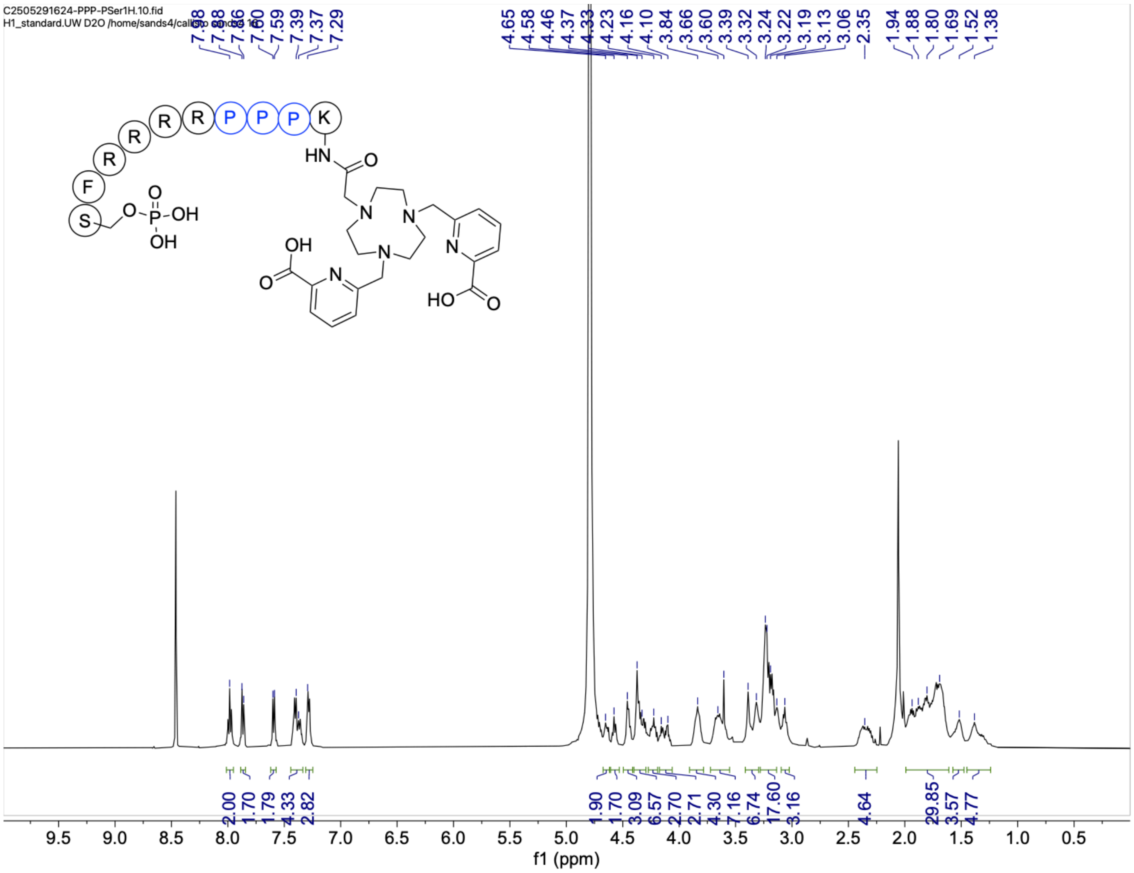


**Figure S217:** $^{1}$H NMR of **20**

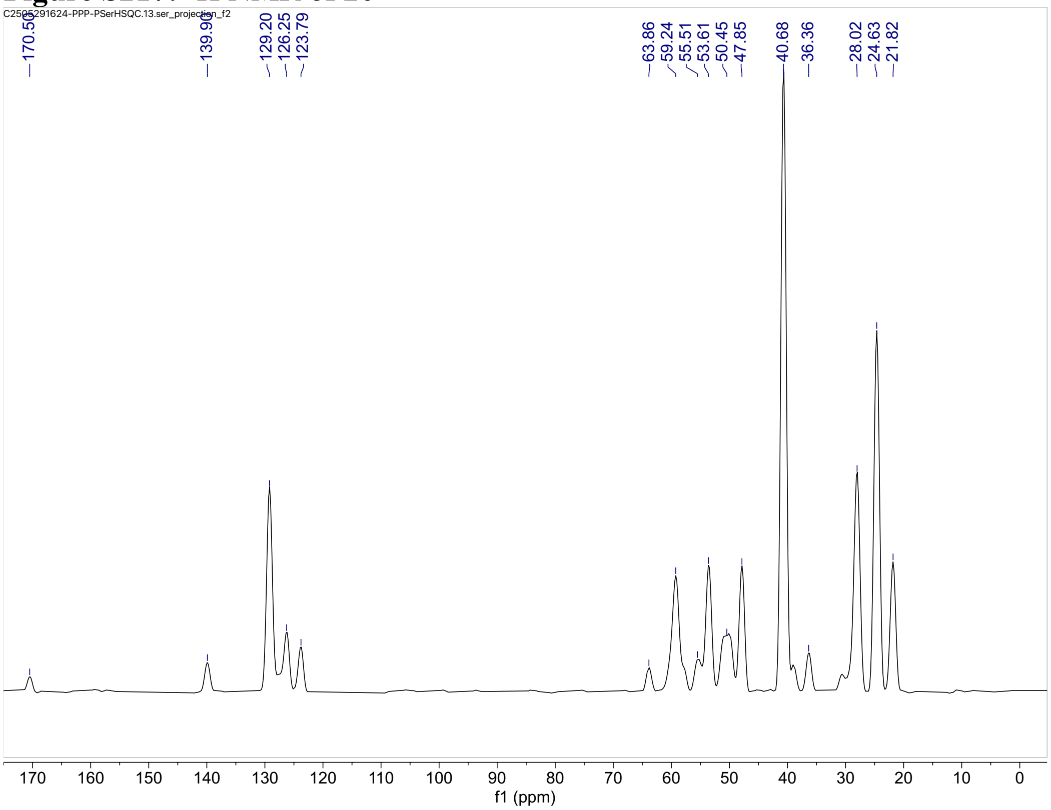

**Figure S218:** $^{13}$C NMR of **20**

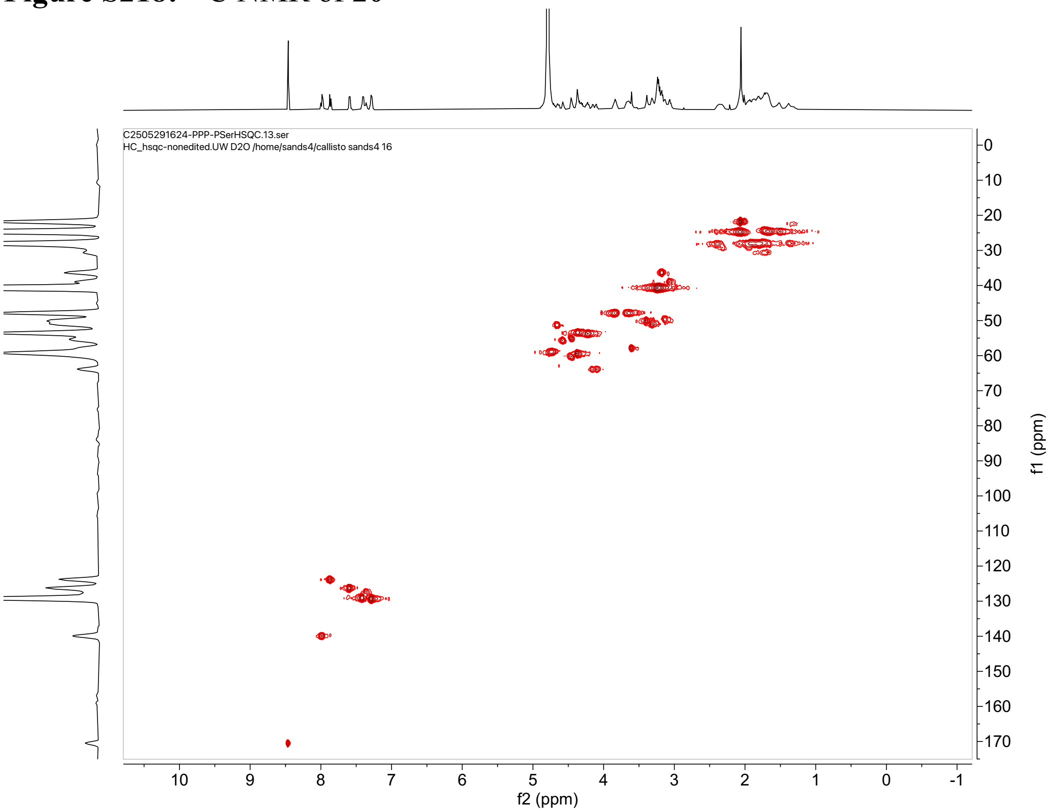


**Figure S219:** HSQC of **20**

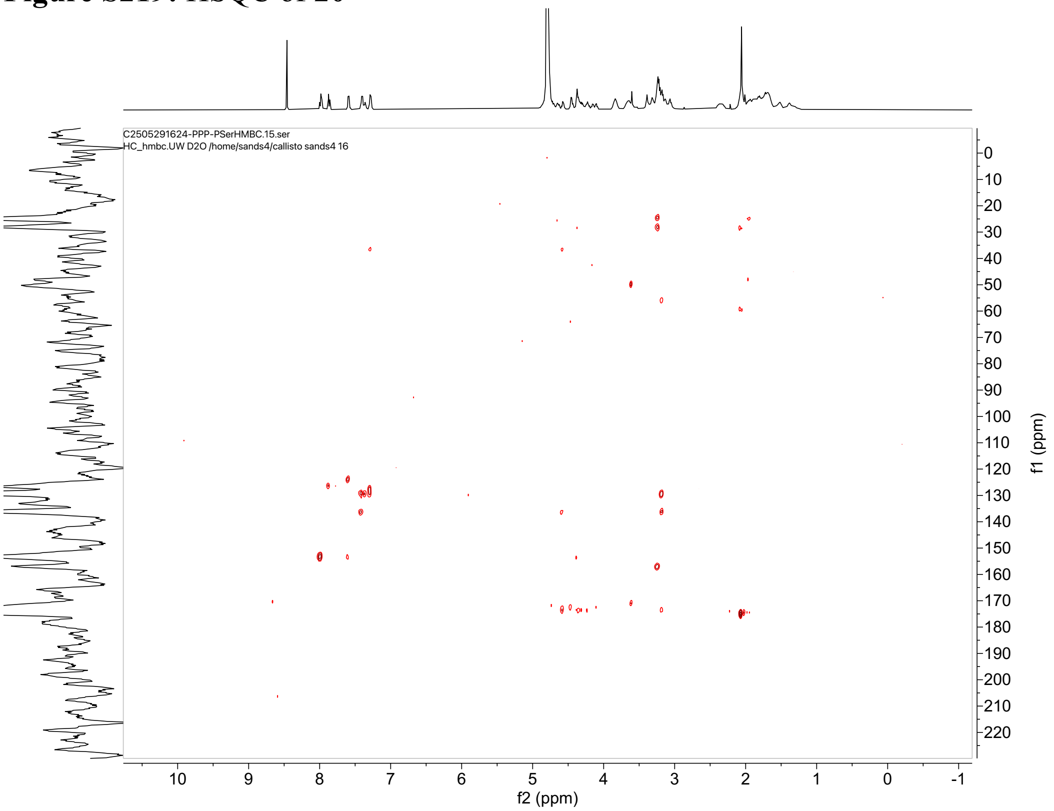

**Figure S220:** HMBC of **20**

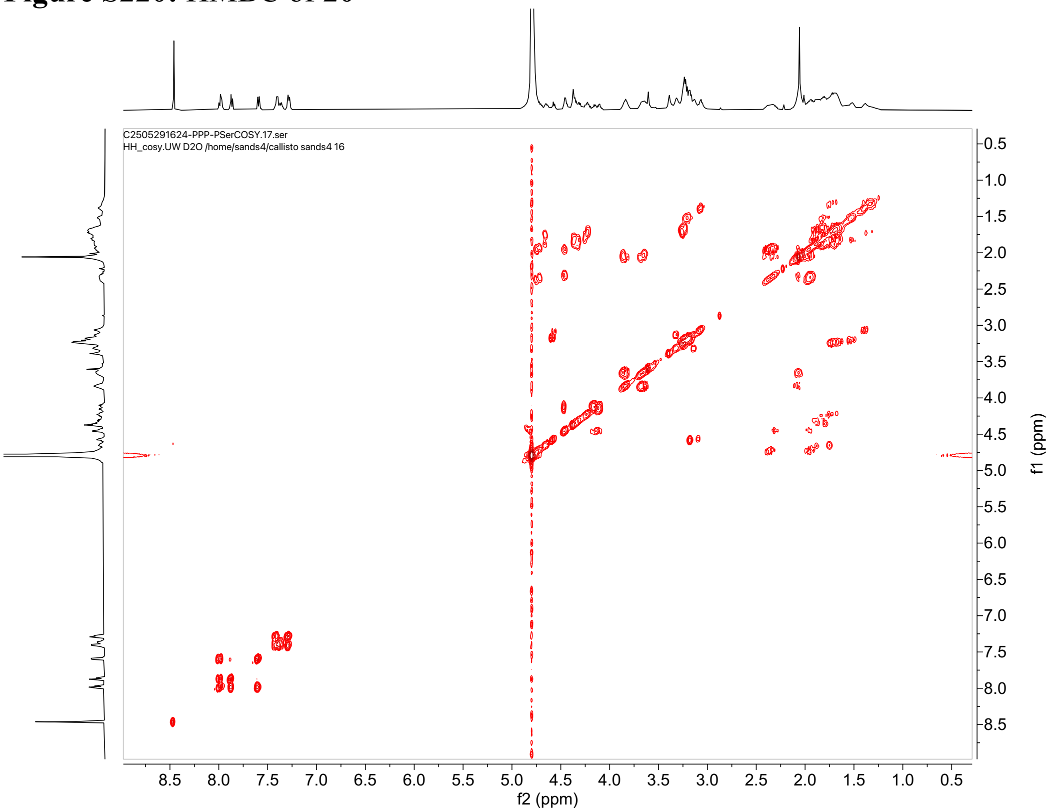


**Figure S221:** COSY of **20**

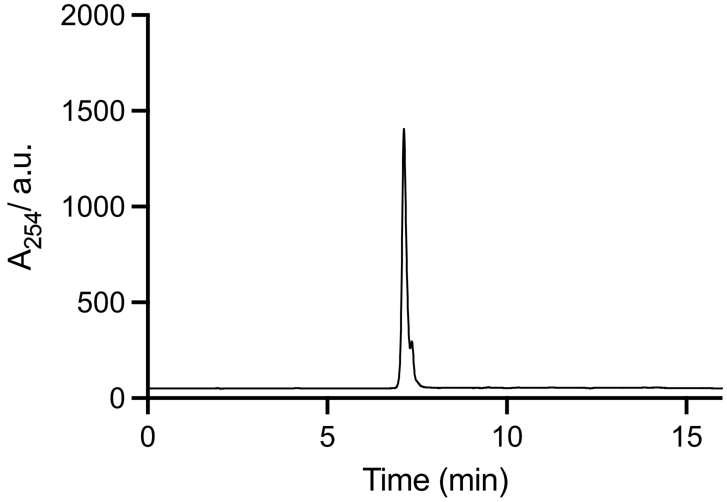


**Figure S222:** Analytical trace of **20** (**$R_t$** = 7.14 min) (method D)

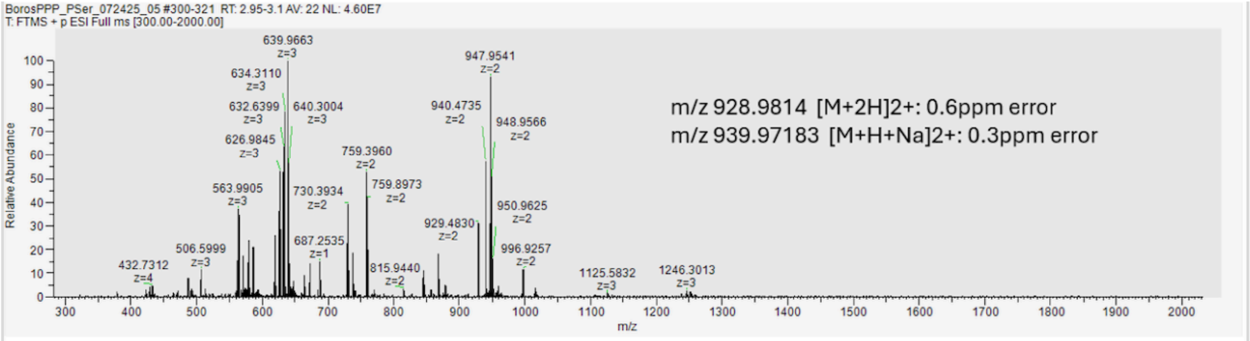

**Figure S223:** HR-ESI of **20**

Synthesis of **[Tb(bispic-GGG-Ser)]**$^+$

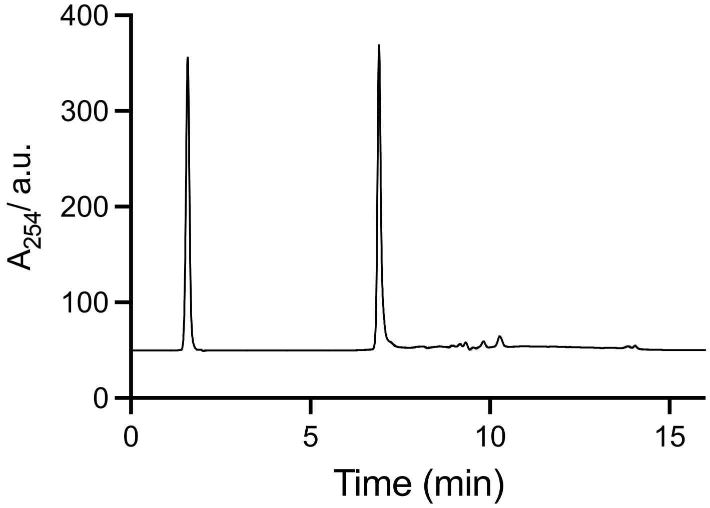

**Figure S224:** Analytical trace of **[Tb(bispic-GGG-Ser)]**$^+$ ($R_t$ = 6.9 min) (method D)

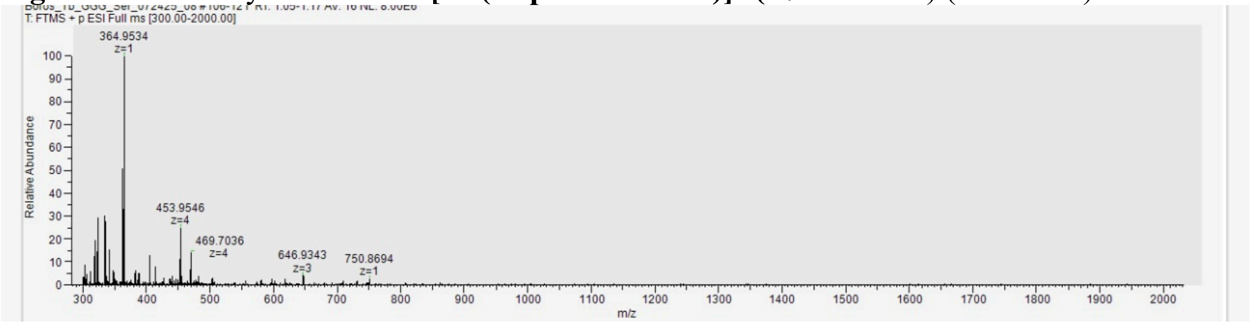

**Figure S225:** HR-ESI of **[Tb(bispic-GGG-Ser)]**$^+$

Synthesis of **[Eu(bispic-GGG-Ser)]**$^+$

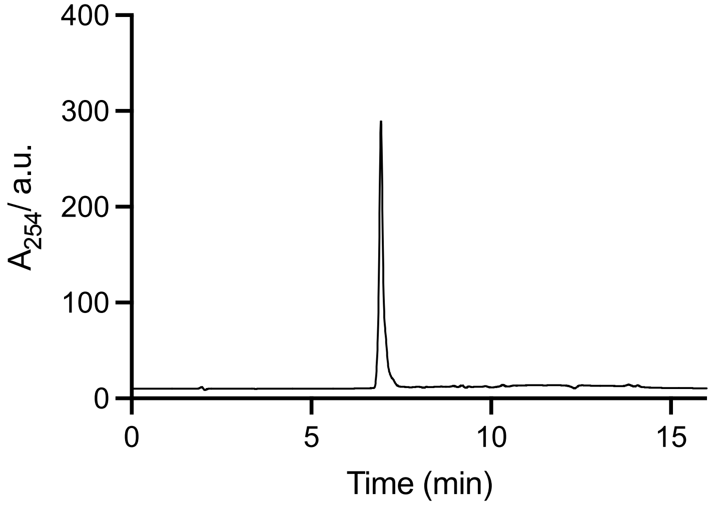


**Figure S226:** Analytical trace of **[Eu(bispic-GGG-Ser)]**$^{+}$ ($R_t$ = 6.96 min) (method D)

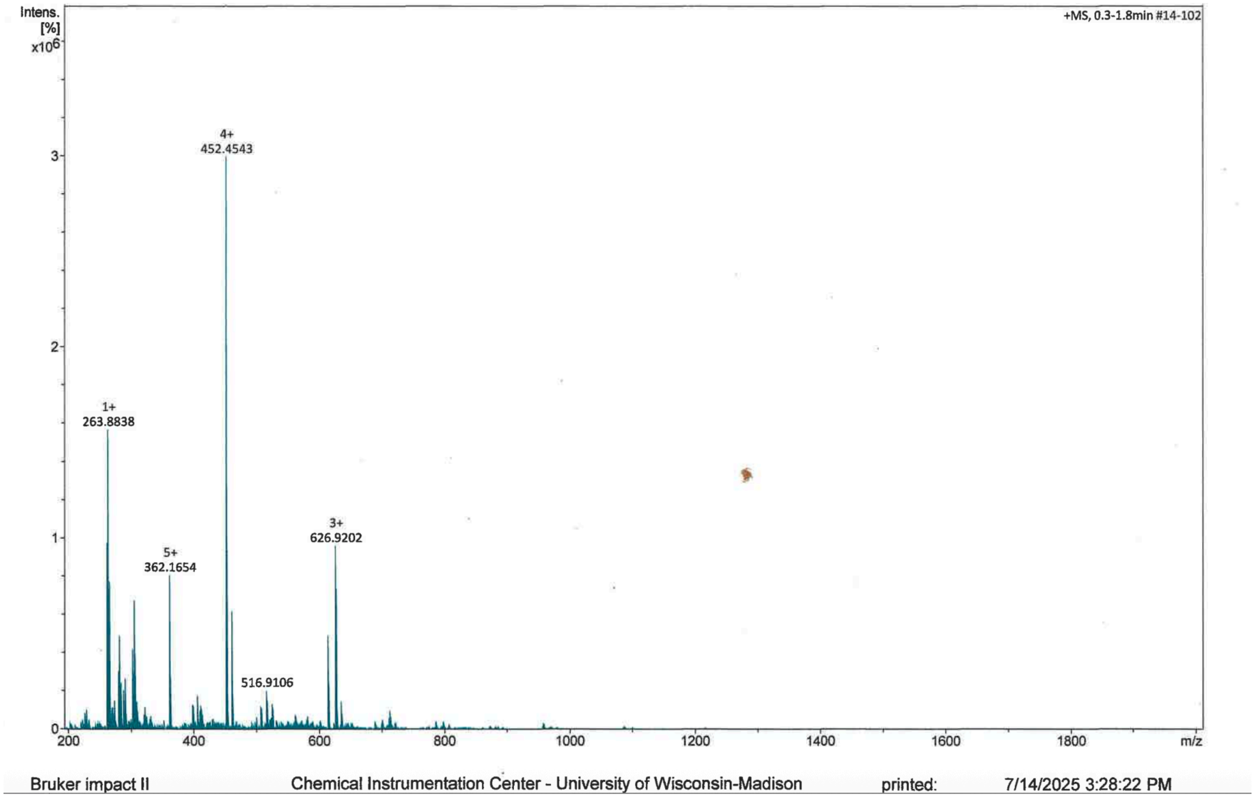


**Figure S227:** HR-ESI of **[Eu(bispic-GGG-Ser)]**$^{+}$

Synthesis of [**Tb(bispic-GGG-PSer)]**$^{-}$

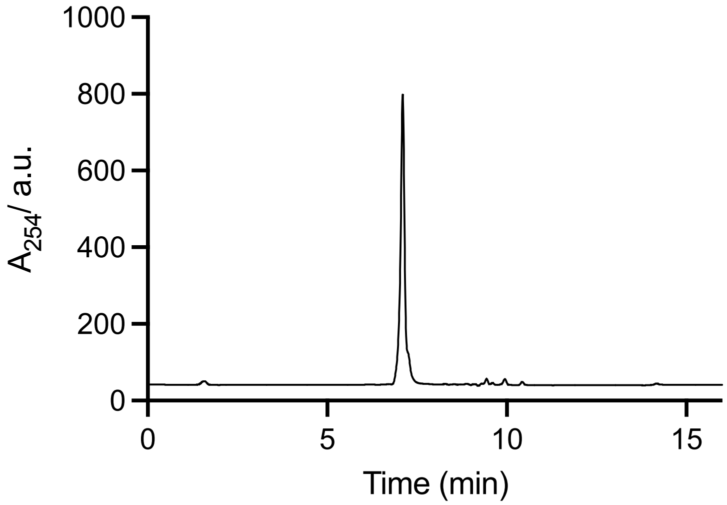


**Figure S228:** Analytical trace of **[Tb(bispic-GGG-PSer)]$^-$** ($R_t$ = 7.11 min) (method D)

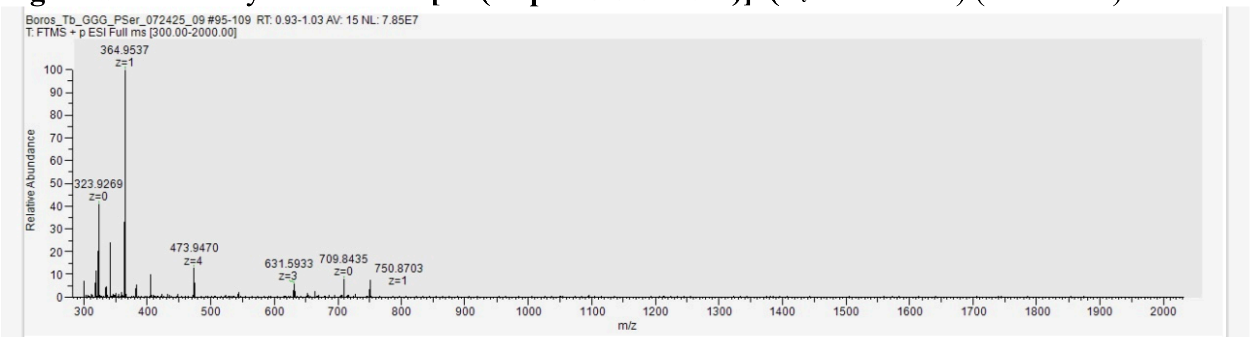


**Figure S229:** HR-ESI of **[Tb(bispic-GGG-PSer)]$^-$**

Synthesis of [**Eu(bispic-GGG-PSer)]$^-$**

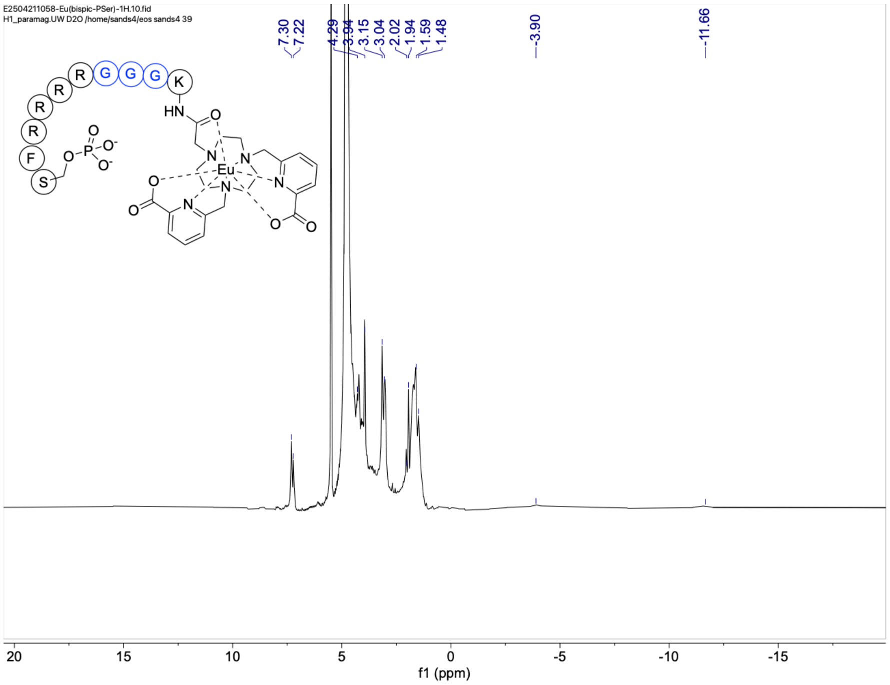

**Figure S230:** $^{1}$H NMR of [**Eu(bispic-GGG-PSer)**]$^{-}$

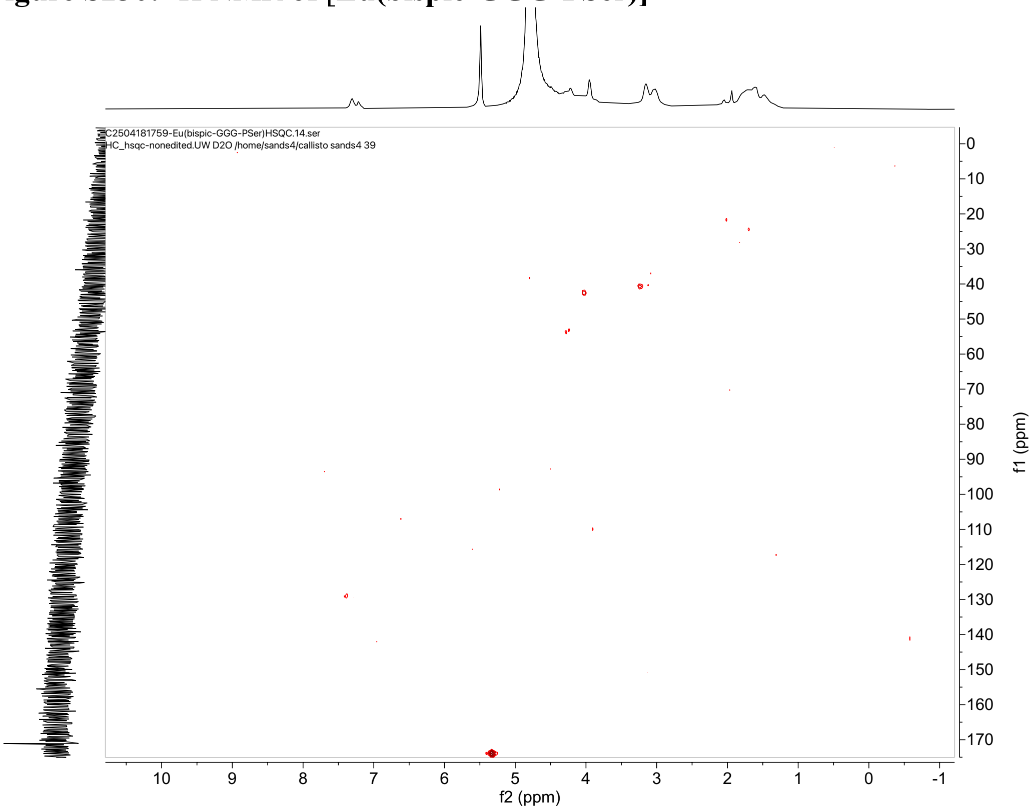

**Figure S231:** HSQC of [**Eu(bispic-GGG-PSer)**]$^{-}$

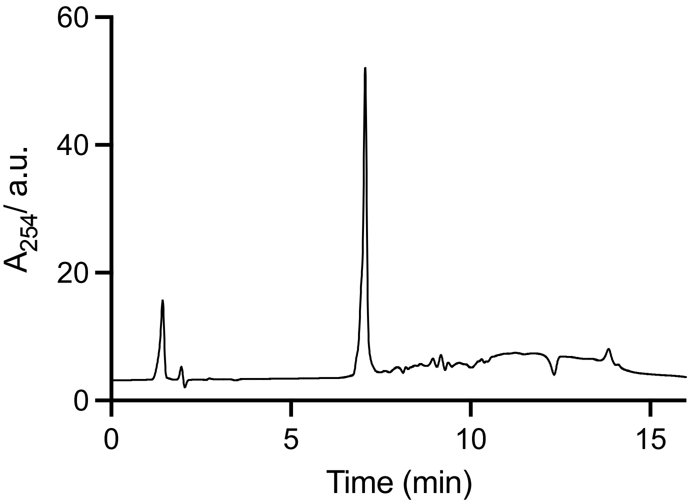


**Figure S232:** Analytical trace of **[Eu(bispic-GGG-PSer)]$^-$ (**$R_t$ = 7.08 min) (method D)

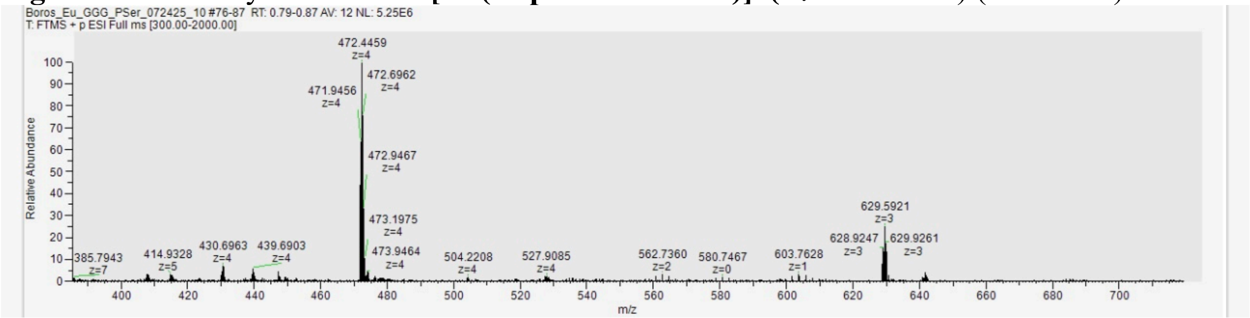


**Figure S233:** HR-ESI of [Eu(bispic-GGG-PSer)]$^-$

Synthesis of **[Tb(bispic-PPP-Ser)]$^+$**

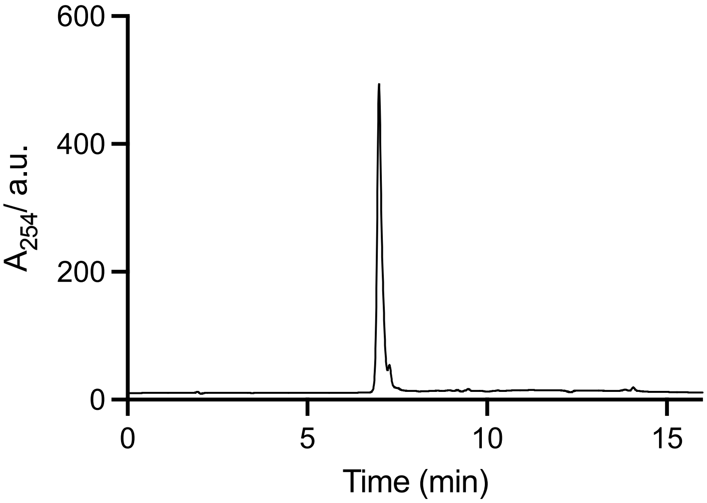


**Figure S234:** Analytical trace of **[Tb(bispic-PPP-Ser)]$^+$ (**$R_t$ = 7.00 min) (method D)

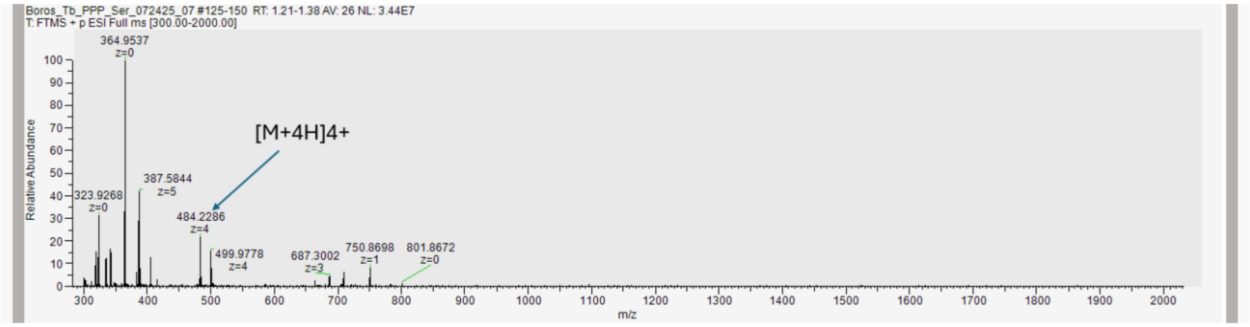


**Figure S235:** HR-ESI of **[Tb(bispic-PPP-Ser)]$^+$**

Synthesis of **[Eu(bispic-PPP-Ser)]$^+$**

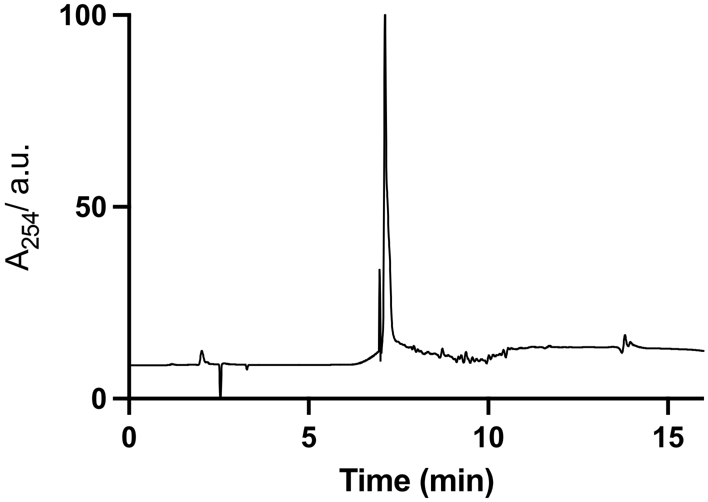


**Figure S236:** Analytical trace of **[Eu(bispic-PPP-Ser)]$^+$** (R$_t$ = 7.01 min) (method D)

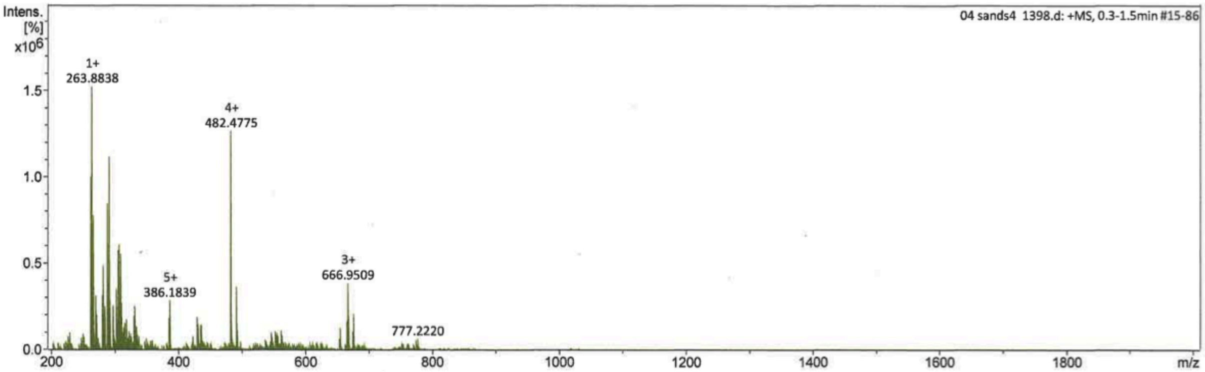


**Figure S237:** HR-ESI of **[Eu(bispic-PPP-Ser)]$^+$**

Synthesis of **[Tb(bispic-PPP-PSer)]$^-$**

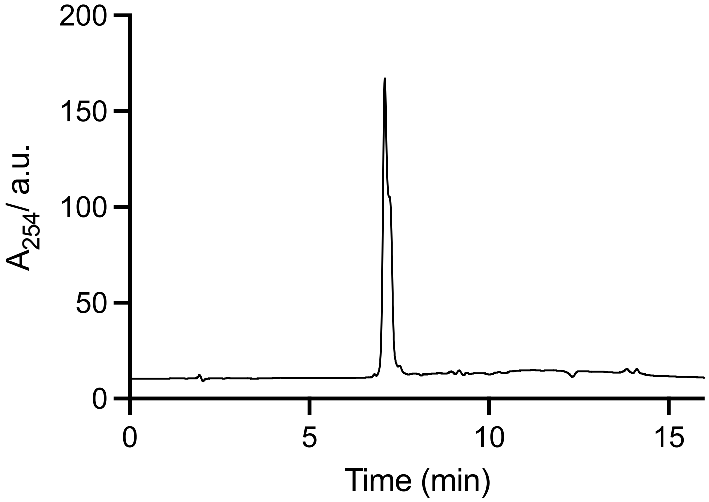


**Figure S238:** Analytical trace of **[Tb(bispic-PPP-PSer)]**$^{-}$ ($R_t$ = 7.11 min) (method D)

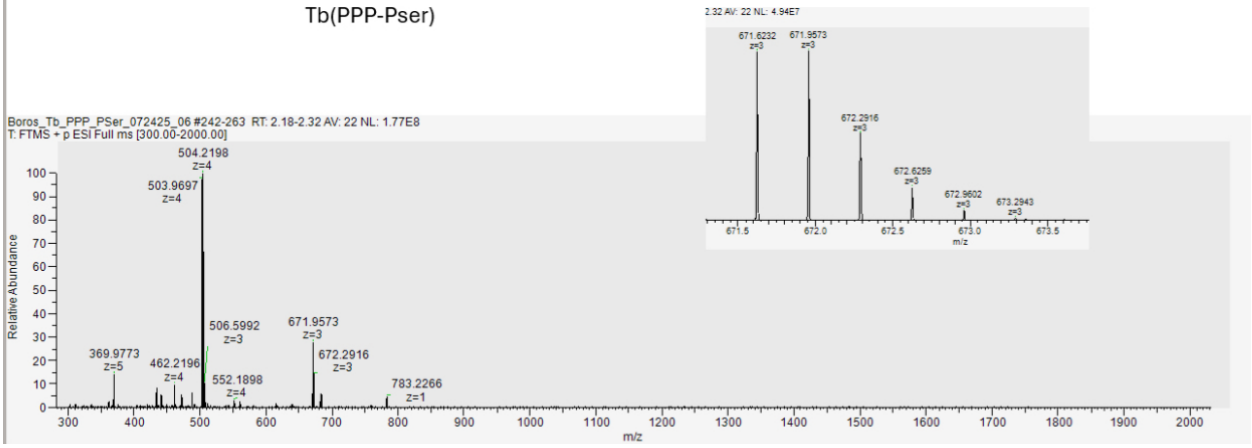


**Figure S239:** HR-ESI of **[Tb(bispic-PPP-PSer)]**$^{+}$

Synthesis of **[Eu(bispic-PPP-PSer)]**$^{-}$

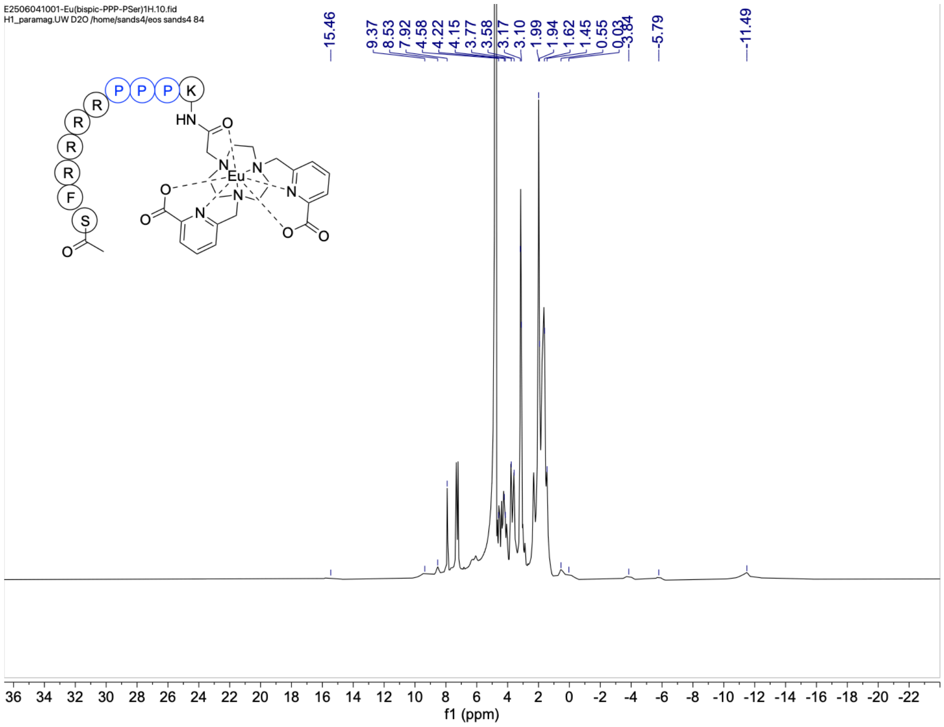

**Figure S240:** $^{1}$H NMR of [**Eu(bispic-PPP-PSer)**]$^{-}$

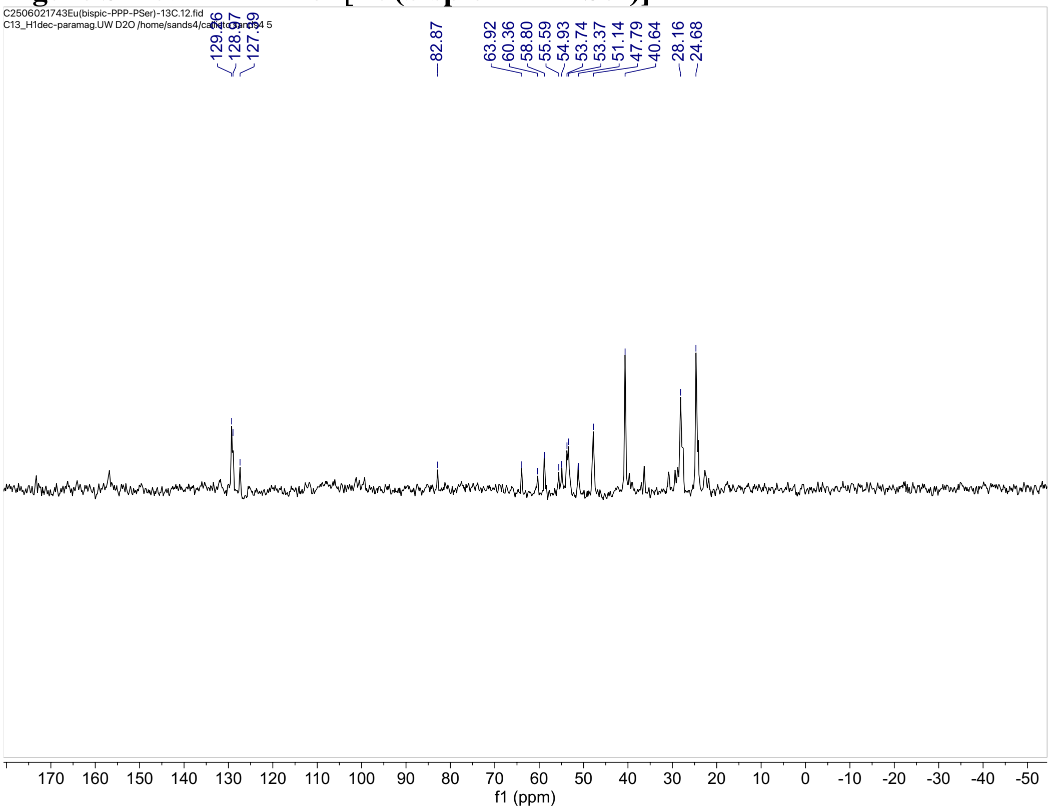

**Figure S241:** $^{13}$C NMR of [**Eu(bispic-PPP-PSer)**]$^{-}$

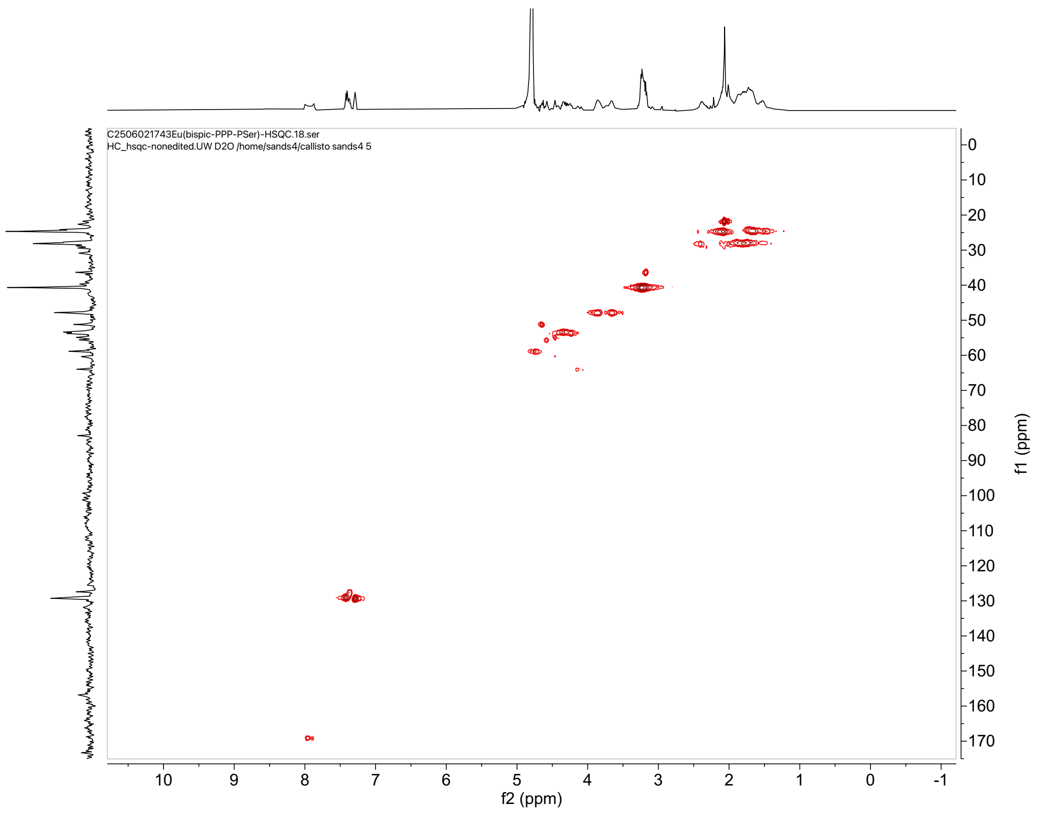


**Figure S242:** HSQC of [**Eu(bispic-PPP-PSer)**]$^{-}$

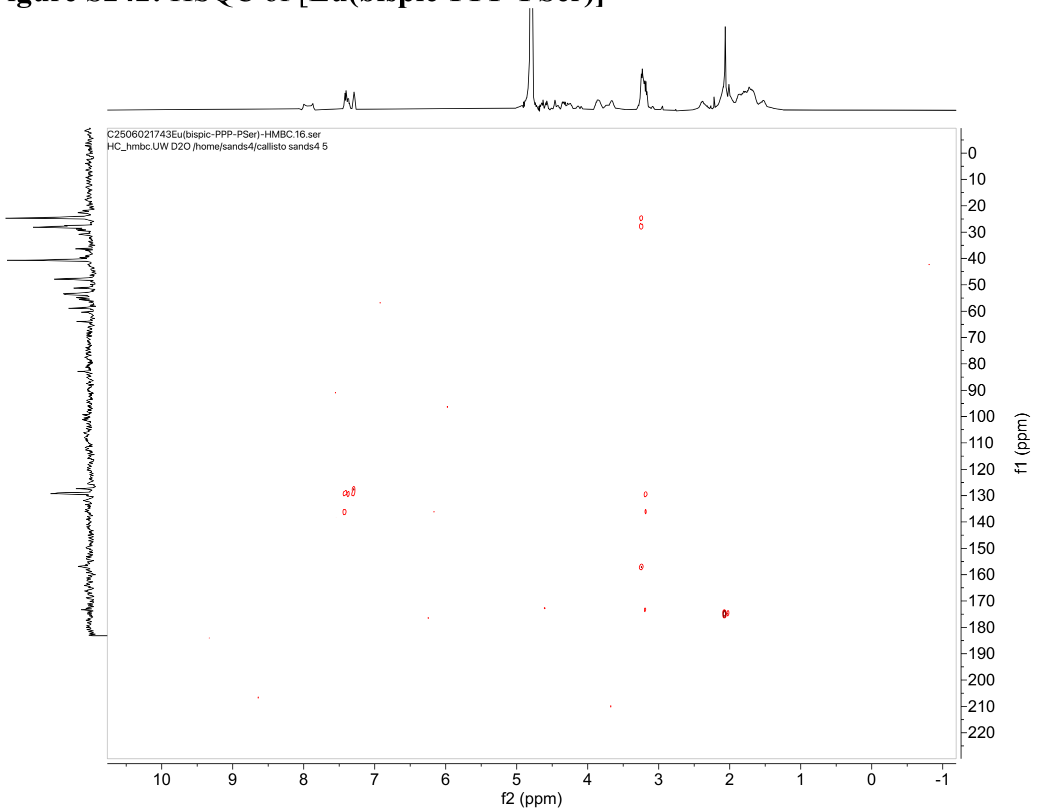


**Figure S243:** HMBC of [**Eu(bispic-PPP-PSer)**]$^{-}$

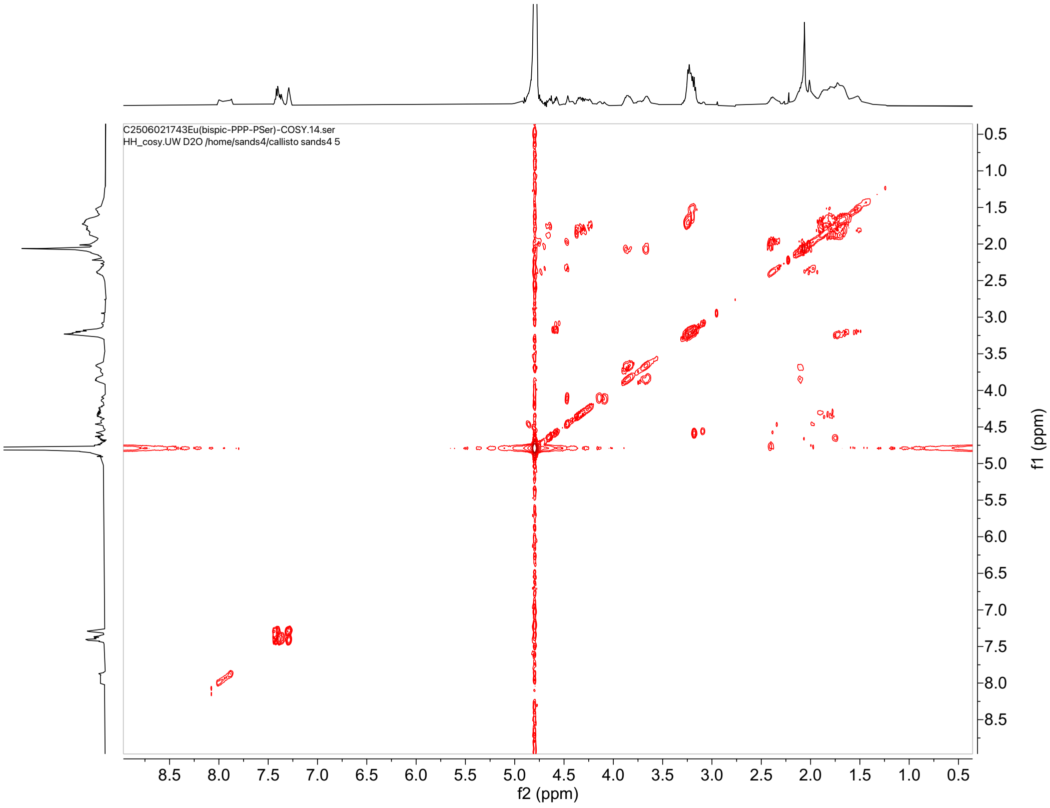


**Figure S244:** COSY of [**Eu(bispic-PPP-PSer)**]$^{-}$

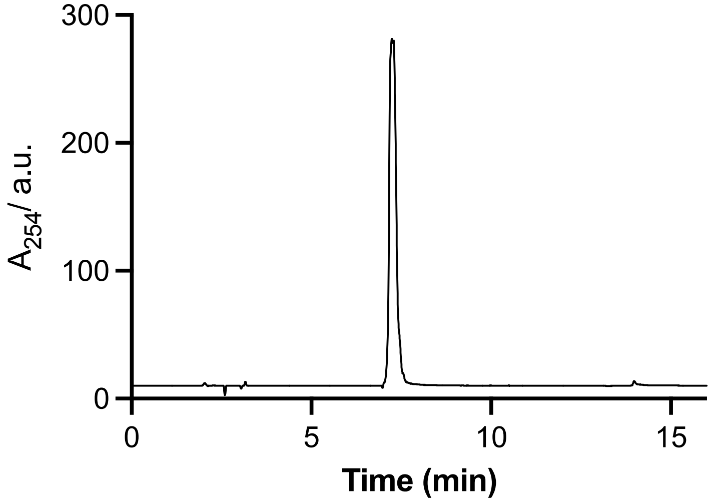


**Figure S245:** analytical trace of [**Eu(bispic-PPP-PSer)**]$^{-}$ **(**$R_t$ = 7.31 min) (method D)

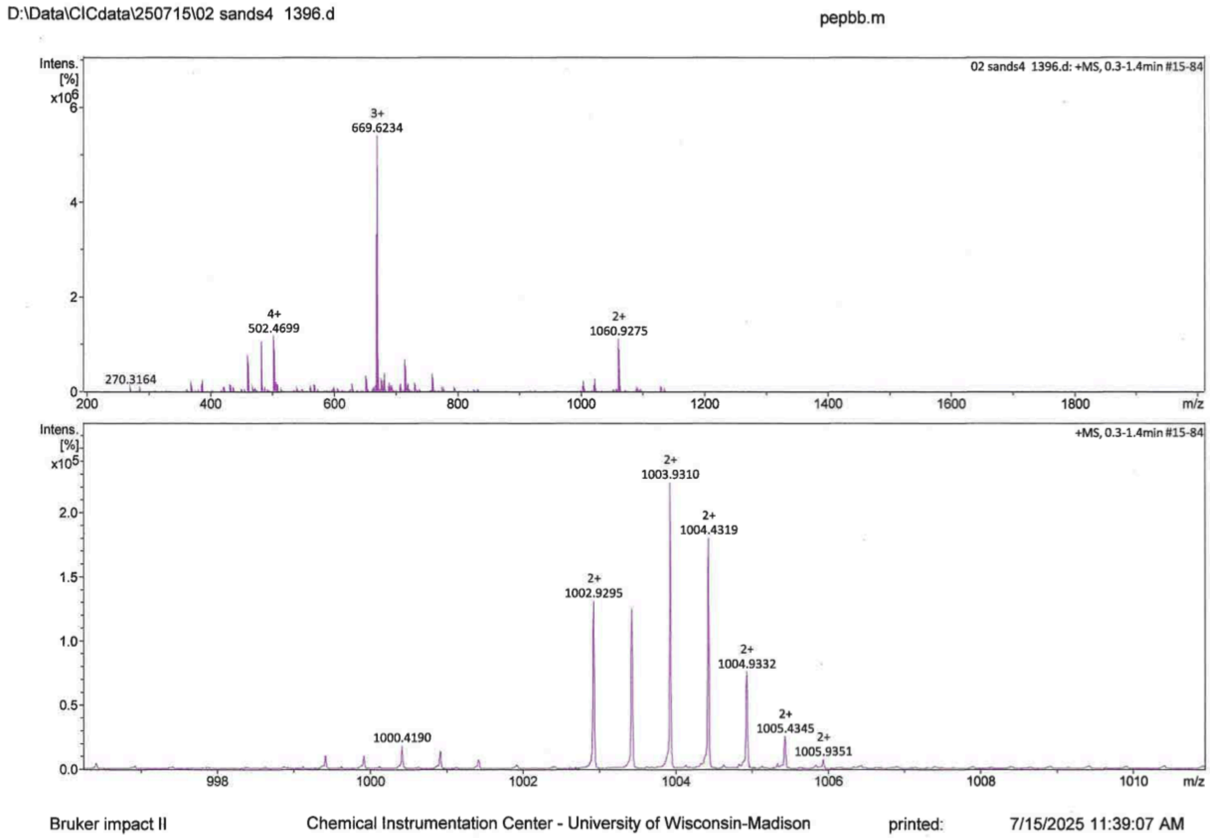


**Figure S246:** HR-ESI of [**Eu(bispic-PPP-PSer)]**$^-$

## References


(1) Brayshaw, P. A.; Buenzli, J.-C. G.; Froidevaux, P.; Harrowfield, J. M.; Kim, Y.; Sobolev, A. N. Synthetic, Structural, and Spectroscopic Studies on Solids Containing Tris(Dipicolinato) Rare Earth Anions and Transition or Main Group Metal Cations. *Inorg. Chem.* **1995**, *34* (8), 2068–2076. https://doi.org/10.1021/ic00112a019.

(2) Kofod, N.; Sørensen, T. J. $Tb^{3+}$ Photophysics: Mapping Excited State Dynamics of $[Tb(H_2O)_9]^{3+}$ Using Molecular Photophysics. *J. Phys. Chem. Lett.* **2022**, *13* (51), 11968–11973. https://doi.org/10.1021/acs.jpclett.2c03506.

(3) Martinon, T. L. M.; Ramakrishnam Raju, M. V.; Pierre, V. C. Kinetically Inert Macrocyclic Europium(III) Receptors for Phosphate. *Inorg. Chem.* **2023**, *62* (26), 10064–10076. https://doi.org/10.1021/acs.inorgchem.2c03833.

(4) Kelderman, C. A. A.; Glaser, O. M.; Whetter, J. N.; Aluicio-Sarduy, E.; Mixdorf, J. C.; Sanders, K. M.; Guzei, I. A.; Barnhart, T. E.; Engle, J. W.; Boros, E. Charting the Coordinative Landscape of the $^{18}$F–Sc/$^{44}$Sc/$^{177}$Lu Triad with the Tri-Aza-Cyclononane (Tacn) Scaffold. *Chem. Sci.* **2024**, *15* (43), 17927–17936. https://doi.org/10.1039/D4SC04735D.

(5) Nocton, G.; Nonat, A.; Gateau, C.; Mazzanti, M. Water Stability and Luminescence of Lanthanide Complexes of Tripodal Ligands Derived from 1,4,7-Triazacyclononane: Pyridinecarboxamide *versus* Pyridinecarboxylate Donors. *Helv. Chim. Acta* **2009**, *92* (11), 2257–2273. https://doi.org/10.1002/hlca.200900150.

(6) Nonat, A.; Gateau, C.; Fries, P. H.; Mazzanti, M. Lanthanide Complexes of a Picolinate Ligand Derived from 1,4,7-Triazacyclononane with Potential Application in Magnetic Resonance Imaging and Time-Resolved Luminescence Imaging. *Chem. – Eur. J.* **2006**, *12* (27), 7133–7150. https://doi.org/10.1002/chem.200501390.
(7) Roux, A.; Talon, R.; Alsalman, Z.; Engilberge, S.; D'Aléo, A.; Di Pietro, S.; Robin, A.; Bartocci, A.; Pilet, G.; Dumont, E.; Wagner, T.; Shima, S.; Riobé, F.; Girard, E.; Maury, O. Influence of Divalent Cations in the Protein Crystallization Process Assisted by Lanthanide-Based Additives. *Inorg. Chem.* **2021**, *60* (20), 15208–15214. https://doi.org/10.1021/acs.inorgchem.1c01635.
(8) Price, E. W.; Cawthray, J. F.; Adam, M. J.; Orvig, C. Modular Syntheses of $H_4$ Octapa and $H_2$ Dedpa, and Yttrium Coordination Chemistry Relevant to $^{86}Y/^{90}Y$ Radiopharmaceuticals. *Dalton Trans* **2014**, *43* (19), 7176–7190. https://doi.org/10.1039/C4DT00239C.
(9) Wang, J.; Wolf, R. M.; Caldwell, J. W.; Kollman, P. A.; Case, D. A. Development and Testing of a General Amber Force Field. *J. Comput. Chem.* **2004**, *25* (9), 1157–1174. https://doi.org/10.1002/jcc.20035.
(10)
(11) Lee, C.; Yang, W.; Parr, R. G. Development of the Colle-Salvetti Correlation-Energy Formula into a Functional of the Electron Density. *Phys. Rev. B* **1988**, *37* (2), 785–789. https://doi.org/10.1103/PhysRevB.37.785.
(12) Becke, A. D. Density-Functional Thermochemistry. III. The Role of Exact Exchange. *J. Chem. Phys.* **1993**, *98* (7), 5648–5652. https://doi.org/10.1063/1.464913.
(13) Grimme, S.; Ehrlich, S.; Goerigk, L. Effect of the Damping Function in Dispersion Corrected Density Functional Theory. *J. Comput. Chem.* **2011**, *32* (7), 1456–1465. https://doi.org/10.1002/jcc.21759.
(14) Dolg, M.; Stoll, H.; Preuss, H. Energy-Adjusted *a b i n i t i o* Pseudopotentials for the Rare Earth Elements. *J. Chem. Phys.* **1989**, *90* (3), 1730–1734. https://doi.org/10.1063/1.456066.
(15) Miertuš, S.; Scrocco, E.; Tomasi, J. Electrostatic Interaction of a Solute with a Continuum. A Direct Utilizaion of AB Initio Molecular Potentials for the Prevision of Solvent Effects. *Chem. Phys.* **1981**, *55* (1), 117–129. https://doi.org/10.1016/0301-0104(81)85090-2.
(16) Sobtop, Version 1.0. http://sobereva.com/soft/Sobtop.
(17) Li, P.; Song, L. F.; Merz, K. M. Parameterization of Highly Charged Metal Ions Using the 12-6-4 LJ-Type Nonbonded Model in Explicit Water. *J. Phys. Chem. B* **2015**, *119* (3), 883–895. https://doi.org/10.1021/jp505875v.
(18) The PyMOL Molecular Graphics System.
(19) Jorgensen, W. L.; Chandrasekhar, J.; Madura, J. D.; Impey, R. W.; Klein, M. L. Comparison of Simple Potential Functions for Simulating Liquid Water. *J. Chem. Phys.* **1983**, *79* (2), 926–935. https://doi.org/10.1063/1.445869.
(20) Pastor, R. W.; Brooks, B. R.; Szabo, A. An Analysis of the Accuracy of Langevin and Molecular Dynamics Algorithms. *Mol. Phys.* **1988**, *65* (6), 1409–1419. https://doi.org/10.1080/00268978800101881.
(21) Åqvist, J.; Wennerström, P.; Nervall, M.; Bjelic, S.; Brandsdal, B. O. Molecular Dynamics Simulations of Water and Biomolecules with a Monte Carlo Constant Pressure Algorithm. *Chem. Phys. Lett.* **2004**, *384* (4–6), 288–294. https://doi.org/10.1016/j.cplett.2003.12.039.

(22) Darden, T.; York, D.; Pedersen, L. Particle Mesh Ewald: An $N\cdot\log(N)$ Method for Ewald Sums in Large Systems. *J. Chem. Phys.* **1993**, *98* (12), 10089–10092. https://doi.org/10.1063/1.464397.
(23) Ryckaert, J.-P.; Ciccotti, G.; Berendsen, H. J. C. Numerical Integration of the Cartesian Equations of Motion of a System with Constraints: Molecular Dynamics of n-Alkanes. *J. Comput. Phys.* **1977**, *23* (3), 327–341. https://doi.org/10.1016/0021-9991(77)90098-5.
(24) Amber 2025.
(25) Maier, J. A.; Martinez, C.; Kasavajhala, K.; Wickstrom, L.; Hauser, K. E.; Simmerling, C. ff14SB: Improving the Accuracy of Protein Side Chain and Backbone Parameters from ff99SB. *J. Chem. Theory Comput.* **2015**, *11* (8), 3696–3713. https://doi.org/10.1021/acs.jctc.5b00255.
